# Adverse events of pharmacological interventions for insomnia disorder in adults: a systematic review and network meta-analysis

**DOI:** 10.3389/fpsyt.2025.1461166

**Published:** 2025-07-09

**Authors:** Changhong Lu, Yuanyuan Geng, Xiaoli Guan, Ying Meng, Mucheng Zhu, Yuan Zhao

**Affiliations:** ^1^ Department of General Medicine, The Second Hospital of Lanzhou University, Lanzhou, China; ^2^ Ophthalmology Department of the Second Hospital of Lanzhou University, Lanzhou, China; ^3^ Department of General Medicine, The Second Clinical College of Lanzhou University, Lanzhou, China

**Keywords:** adverse events, safety, insomnia drug, network meta-analysis, systematic review

## Abstract

**Background:**

The clinical decision-making to insomnia drugs should comprehensively weight its risks.

**Objective:**

To perform a systematic review and network meta-analysis of randomized controlled trials to compare the AEs associated with different insomnia drugs for adults with insomnia.

**Methods:**

We conducted Bayesian network meta-analyses and fixed-effects Mantel-Haenszel network meta-analyses to estimate the relative safety between treatments.

**Results:**

Compared with placebo, zolpidem (somnolence: relative risk [RR] 1.85; dizziness: RR 2.33; headache: RR 1.26), zopiclone (somnolence: RR 2.02; dizziness: RR 2.33; dysgeusia: RR 7.84), indiplon (somnolence: RR 3.46; dizziness: RR 2.30; headache: RR 1.63), gaboxadol (dizziness: RR 3.44), eszopiclone (somnolence: RR 2.00; dizziness: RR 3.18; dysgeusia: RR 10.54), estazolam (somnolence: RR 2.08), flunitrazepam (somnolence: RR 3.04), flurazepam (somnolence: RR 2.52), lemborexant (somnolence: RR 6.57), nitrazepam (somnolence: RR 3.80), Ramelteon (somnolence: RR 2.19), suvorexant (somnolence: RR 3.32), Temazepam (somnolence: RR 3.77), trazodone (somnolence: RR 2.86), triazolam (somnolence: RR 2.35), and esmirtazapine (somnolence: RR 4.63; dizziness: RR 2.87) had the most harmful profile in nervous system disorders. Additionally, compared to placebo, zolpidem was also found to be associated with dry mouth (RR 1.92) and anxiety (RR 3.32); gaboxadol was associated with nausea/vomiting (RR 3.49); and eszopiclone was associated with dry mouth (RR 4.39). Doxepin was associated with lower risk of headache and somnolence than placebo or/and most of other drugs, and had also a lower rate of AEs. We observed no associations between drugs and the risks of serious AEs including nasopharyngitis, respiratory problem, accidental injury, infection, upper respiratory tract infection, sinusitis, or hematuria.

**Conclusions:**

Most drugs were positive associated with nervous system disorders and gastrointestinal disorders. Data on some drugs like flurazepam, nitrazepam, triazolam, and zaleplon in some outcomes were mainly based on limited study with rare event and thus was highly uncertain and do not allow firm conclusions.

**Systematic Review Registration:**

https://www.crd.york.ac.uk/prospero/, identifier CRD42022344981.

## Introduction

Insomnia is the most commonly reported sleep problem, affecting 19% to 50% of adults (1–3). Insomnia is often associated with chronic physical and mental health problems (2–4). Evidence demonstrated that pharmacological treatments could be effective for the management of insomnia symptoms (5–7), and meanwhile, the number of prescriptions for insomnia have been skyrocketed from 5.3 million in 1999 to 20.8 million in 2010 (8). However, the risks of insomnia drugs should be comprehensively weighted in clinical decision-making because almost all insomnia drugs are sedating and thus may increase the risk of confusion and falls, especially in elderly patients (9). Previous studies (5–7) have documented that the acceptability and safety of some insomnia drugs may be poor, and patients with insomnia might be hesitant to initiate treatment with insomnia drugs because potential risks of adverse events (AEs) such as anterograde amnesia, somnolence, fatigue, and abnormal dreams. Clinical practice guidelines also suggested that the benefits of insomnia drugs could be likely to outweigh harms (10). Patients might consider specific adverse effects to be important when making decisions about whether to use or not use a drug, particularly in the face of considerable uncertainty regarding their desirable effects. Therefore, it is crucial to better understand the risks of AEs in order to determine the trade-off between the benefits and harms of insomnia drugs in patients with insomnia.

Evidence-based safety concerns and warnings are essential to inform treatment guidelines, clinical care, and to realize an individualized treatment decision (11). Our study aims to perform a systematic review and network meta-analysis of randomized controlled trials to compare the AEs associated with different insomnia drugs for adults with primary insomnia, and to provide evidence for shared decision-making between doctors and patients.

## Method

### Protocol registration

We registered our systematic review and network meta-analysis on PROSPERO: CRD42022344981. We performed this study in accordance with the Preferred Reporting Items for Systematic Reviews and Meta-Analyses extension statement for network meta-analyses (12) (**Appendix 1**).

### Search strategy and selection criteria

We searched PubMed, Embase, Cochrane Central Register of Controlled Trials (CENTRAL), and PsycINFO from database inception until Oct 9^th^ 2023. Detailed search strategy including search terms is shown in **Appendix 2**. We also reviewed the references of relevant reviews and ClinicalTrials.gov to additionally supplement the any potential trials. We made no restrictions on the year of publication, publication language, or publication status.

Randomized controlled trials (RCTs) that met all of the following criteria were eligible: a) they enrolled adults aged >18 years with primary insomnia; b) they compared one insomnia drug with or without placebo or an alternative active drug; c) at least one specific adverse event (i.e. the number of patients with one specific adverse event and the total number of patients) was reported. We made no restrictions on treatment duration. We excluded studies that reported patients with psychiatric, physical, or general medical conditions, cluster-randomized trials, and cross-over trials because of considerably potential sources of heterogeneity. We also excluded non-pharmacological, augmentation (e.g., drug A+ drug B versus drug A), and herbal medicine interventions.

After training and calibration exercises, teams of three reviewers using Rayyan (13) independently screened all titles and abstract according to a structured screening form (see **Appendix 3**), followed by evaluation of full texts of articles that were identified as potentially eligible. Any conflicts were resolved by discussion.

### Outcome measure

We extracted outcomes of interest at any time point during the study follow-up. The primary outcomes were the following common specific AEs that were reported by more than 2% of the participants in recent large trials (14–16): nervous system disorders (somnolence, dizziness, headache, amnesia, dysgeusia, difficulty concentrating, impaired coordination, nervousness, nightmare, and asthenia), gastrointestinal disorders (dyspepsia, diarrhea, dry mouth, nausea/vomiting, constipation, abdominal pain, and increased appetite), general disorders and administration site conditions (fatigue, and pain), respiratory, thoracic and mediastinal disorders (nasopharyngitis, and respiratory problem), psychiatric disorders (anxiety, confusional state, depression, and emotional lability), injury, poisoning and procedural complications (accidental injury), musculoskeletal and connective tissue disorders (arthralgia, back pain, and myalgia), eye disorders (eye pain), infections and infestations (infection, upper respiratory tract infection, urinary tract infection, and sinusitis). Secondary outcomes were nervous system disorders (abnormal dreams, abnormal vision, gait disturbance, hypnagogic hallucinations, tremor, and paresthesia), gastrointestinal disorders (gastroenteritis, decreased appetite, pain gastralgia), sleeping problem (insomnia exacerbated, and sleep paralysis), skin and subcutaneous tissue disorders (peripheral edema, pruritis, sweating, and skin diseases), general disorders and administration site conditions (influenza, common cold, and malaise), respiratory, thoracic and mediastinal disorders (cough), psychiatric disorders (suicidal ideation, hallucinations, and irritability), injury, poisoning and procedural complications (falls, and laceration), eye disorders (dry eyes), investigations (alanine aminotransferase increased, blood creatine phosphokinase increased, weight increased, **γ-**Glutamyl transferase increased), metabolism and nutrition disorders (hyperglycemia), reproductive system and breast disorders (dysmenorrhea), vascular disorders (hypertension), cardiac disorders (tachycardia), and renal and urinary disorders (hematuria). Adverse events could be assessed by monitoring, clinical and physical examinations, vital signs, routine laboratory parameters, and electrocardiograms. The AEs were classified according to the Common Terminology Criteria for Adverse Events (CTCAE) version 5.0 (17).

### Data extraction

After pilot testing of data extraction forms, the articles were divided to two pairs of investigators in Sep 4^th^ 2022. Both investigators independently extracted the following data using a standard data collection form: first author, year of publication, study design, single-center or multicenter study, country of study, inclusion criteria, exclusion criteria, study duration, total sample size, age, gender distribution, insomnia diagnosis, severity of illness, treatment setting, number of patients allocated to each arm, drug name, dose, route or administration, duration of the interventions, follow-up, and outcomes of interests.

### Risk of bias assessment

Teams of two reviewers the risk of bias for each included RCTs in duplicate using a modified version of the Cochrane risk of bias tool (18), which includes the following six items: was the allocation sequence adequately generated; was the allocation adequately concealed; blinding of patients and healthcare providers; blinding of outcome assessors; was loss to follow-up (missing outcome data) infrequent; and are reports of the study free of selective outcome reporting. We used response options of “definitely or probably yes” (assigned to be a low risk of bias) and “definitely or probably no” (assigned to be a high risk of bias). Disputes were resolved by discussion or through adjudication by a third reviewer. We classified individual studies as to be low risk of bias if all of six questions were low risk (definitely or probably yes), and otherwise, as having a high risk of bias.

### Data synthesis and statistical methods

We used Bayesian pairwise meta-analysis to obtain the pooled direct estimates and assessed heterogeneity using the *I2* statistic and visual inspection of forest plots (19). We calculated risk ratios (RRs) with 95% credible intervals (CrIs) for each outcome of interest.

We performed primary network meta-analysis using a Bayesian Markov-chain Monte-Carlo simulation method (20, 21). We simulated each model by using three chains with 100,000 sample iterations with an initial burn­in of 10,000 and a thinning of 10 for all analyses. We assessed the goodness of model fit by the posterior mean of the overall residual deviance (22) for both random effect and fixed effect models, and we chose a well-fitting model with residual deviance close to the number of data points included in the analysis as the final analysis model. We used vague priors for all variance parameter in the primary analysis, and specified priors as sensitivity analysis. We used node splitting method to assess the local inconsistency between direct and indirect estimates for each closed loop and to obtain the effect estimates of indirect evidence (23). We calculated the surface under the cumulative ranking curve (SUCRA) to evaluated the ranking probabilities for all treatments. Bayesian network meta-analysis was conducted using the *‘gemtc’* package under R software version 4.2.1 (R Core Team, Vienna, Austria). The presence of small-study effects bias was assessed by means of funnel plots when more than studies were available for a comparison.

Given the outcomes of interest are expected to be rare, we also performed sensitivity analyses by conducting fixed-effects Mantel-Haenszel network meta-analyses using the ‘*netmetabin*’ package (24) to assess the robustness of primary results. Although the effect estimates of Mantel-Haenszel network meta-analyses are given as odds ratios (OR), odds and risks are almost identical for rare events. We also compared the distributions of characteristics across study arms grouped by individual agents to assess the transitivity assumption of indirect comparisons by drawing a box plot. We used the function of *‘networkplot’* function of STATA version 15.1 (StataCorp, College Station, TX) to draw network plots to describe and present the geometry of different forms of pharmacologic treatment.

## Results

**Figure 1:**
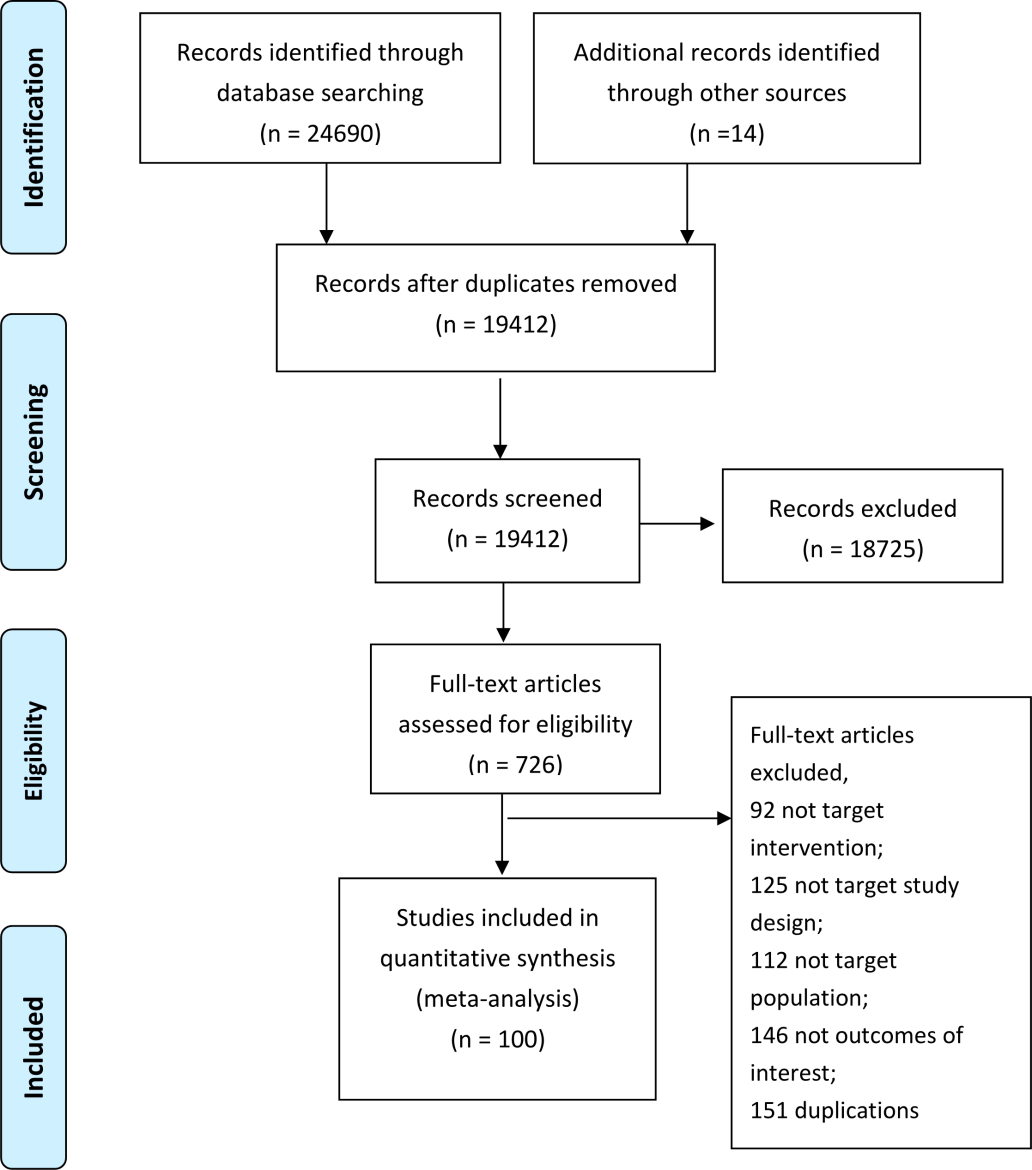
Study selection process.

We totally identified 24,704 unique citations through our initial search (**Figure 1**). After screening the titles and abstracts, we assessed 687 full-text articles, from which 102 trials reported in 100 articles comprising 35,700 participants and 33 insomnia drugs proved eligible (**Figure 1**). The 102 trials were published between 1977 and 2022, 51 trials (48.6%) were from USA (**Appendix 4**), with a median mean age of 47.4 years, a median proportion of women of 60.2%, a median treatment duration of 4 weeks (range from 4 days to 48 weeks). About a quarter (26.3) focused on patients aged older than 60 years old, 65.3% used DSM criteria for insomnia diagnosis.

**Figure 2:**
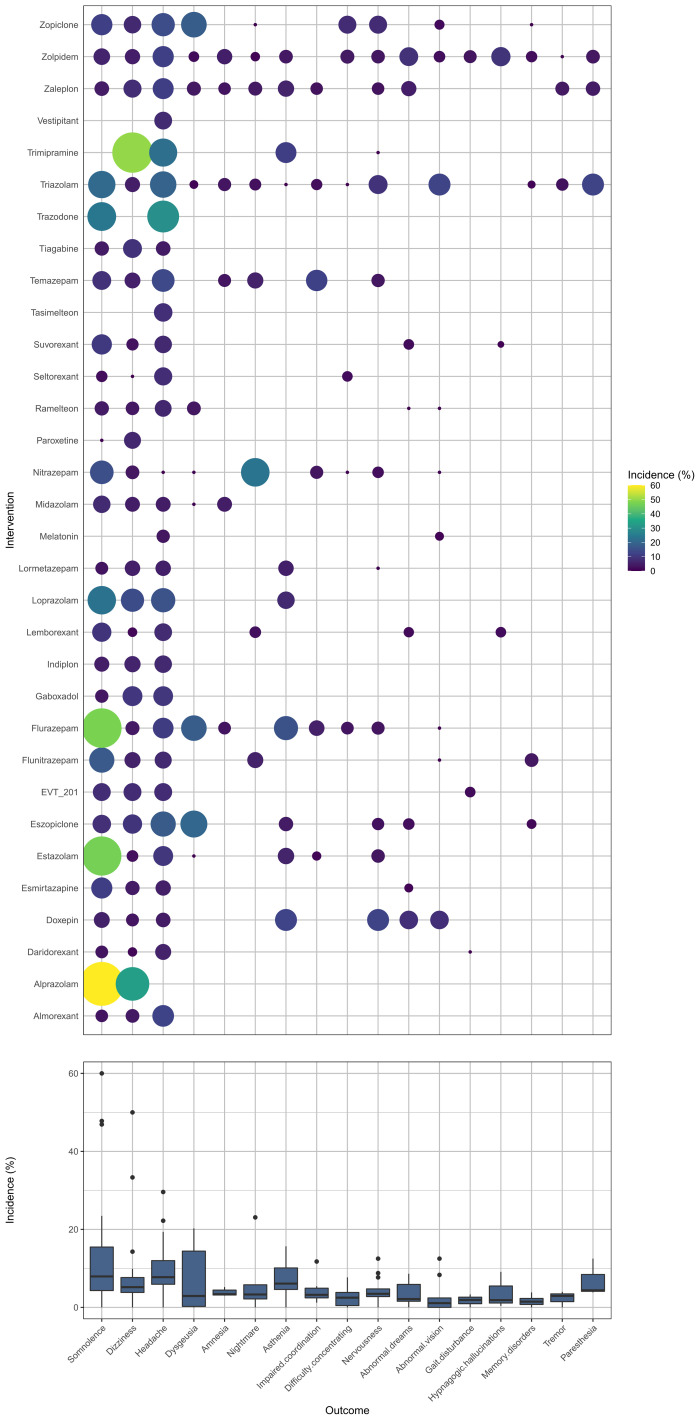
The incidence of nervous system disorders.

**Figure 3:**
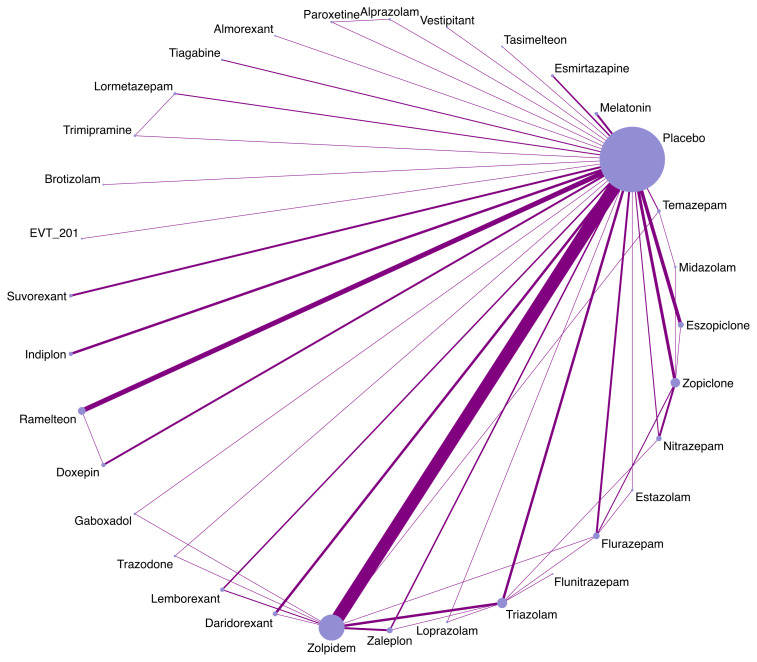
Network plots of available comparisons.

**Appendix 5** summarizes the details of the drugs. A total of 33 drugs forming 48 pairwise comparisons were included. The most common pairwise comparison was zolpidem versus placebo. The 102 trials reported on 69 AEs, in which the most commonly measured AEs was headache (74 trials), followed by somnolence and dizziness (62 trials). Among insomnia drugs, trazodone showed the highest incidence of headache, alprazolam showed the highest incidence of somnolence, and trimipramine showed the highest incidence of dizziness (**Figure 2**). The incidence of other AEs was presented in **Appendix 6**. The direct comparisons are presented in the network diagram (**Figure 3**).

**Figure 4:**
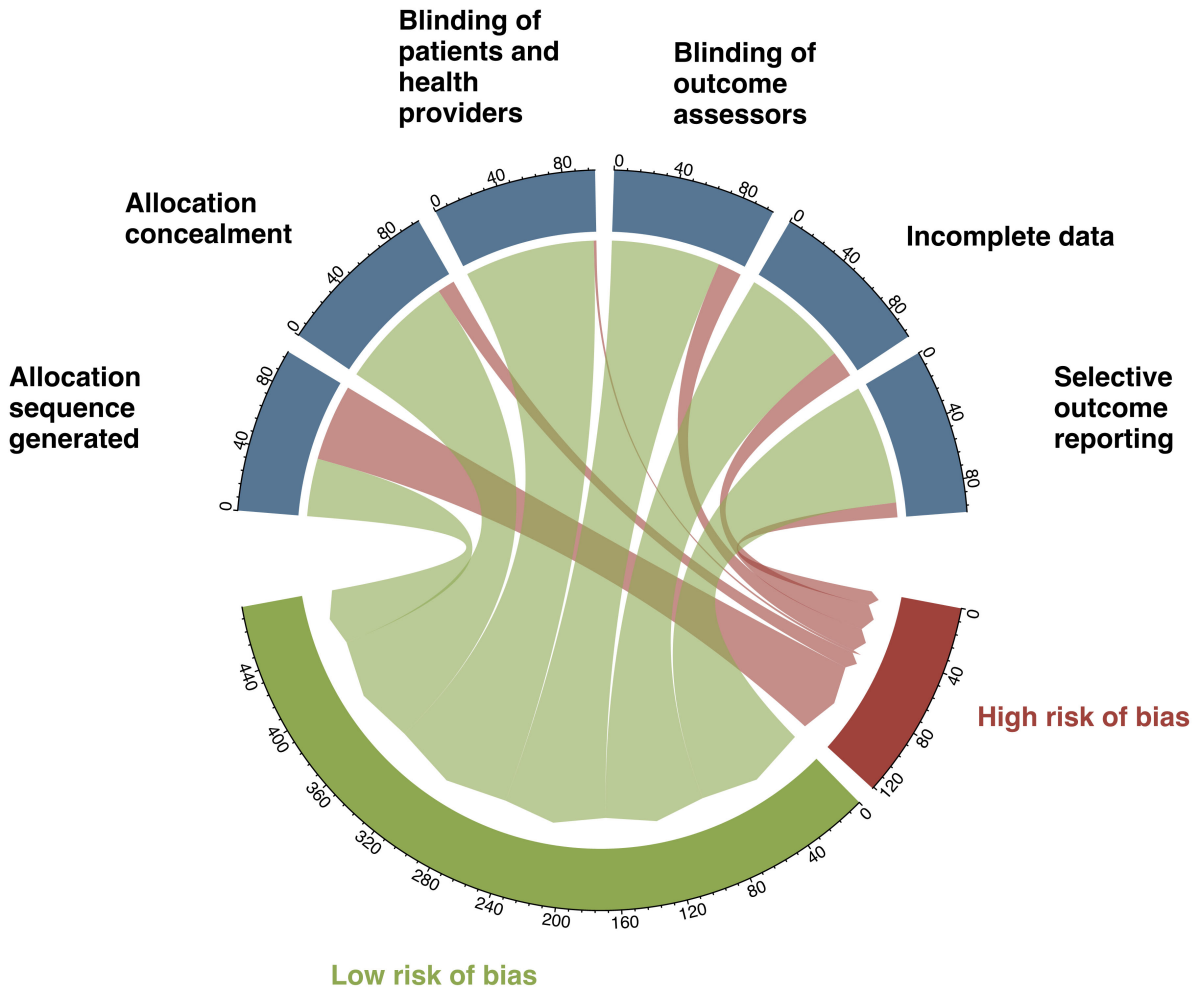
Results of risk of bias.

**Appendices 7**, **8** summarize the guidance for risk of bias assessment and the estimated risk of bias for each trial. The most common limitation was inadequate reporting on methods generated allocation sequence (56%; **Figure 4**).

The network evidence plots for each outcome are presented in **Appendix 9**. The results of the direct and the network meta-analyses are presented in **Appendices 10**, **11**. We also evaluated heterogeneity (see **Appendix 10**) and inconsistency (incoherence; see **Appendix 12**). The sensitivity analyses are presented in **Appendix 13**. **Appendix 14** presents the results of posterior mean of the overall residual deviance for all outcomes and **Appendix 15** shows the reference list of included studies. **Tables 1**–**4** present the summary results of the network estimates for all outcomes. **Appendix 16** presents the estimated small-study effect bias. Some drugs did not exhibit statistically significant adverse events in our analysis. The full list is available in **Tables 1**-**4**.

**Table 1:** Summary results of the network meta-analyses for the most common adverse events: nervous system disorders.

Individual drugs	Nervous system disorders									
	Somnolence	Dizziness	Headache	Amnesia	Difficulty concentrating	Nightmare	Impaired coordination	Dysgeusia	Nervousness	Asthenia
Almorexant	1.9 (0.68 to 5.36)	1.65 (0.6, 4.56)	0.94 (0.61, 1.45)	/	/	/	/	/	/	/
Alprazolam	***SV***	3.6 (0.88, 19.62)	/	/	/	/	/	/	/	/
Daridorexant	1.68 (0.9, 3.25)	1.13 (0.52, 2.47)	**1.47 (1.04, 2.15)**	/	/	/	/	/	/	/
Doxepin	0.96 (0.5, 1.9)	1.16 (0.26, 5.17)	**0.51 (0.28, 0.92)**	/	/	/	/	/	0.7 (0.07, 6.27)	1.55 (0.05, 50.73)
Esmirtazapine	**4.63 (2.38, 9.82)**	**2.87 (1.16, 8.49)**	0.72 (0.44, 1.15)	/	/	/	/	/	/	/
Estazolam	**2.08 (1.14, 3.86)**	1.13 (0.11, 7.58)	0.66 (0.27, 1.46)	/	/	/	9381.88 (0.24, 1985168036512689)	0 (0, 0.32)	0.38 (0.04, 2.88)	0.52 (0.02, 10.99)
Eszopiclone	**2 (1.26, 3.19)**	**3.18 (1.61, 6.5)**	1.02 (0.82, 1.26)	/	/	/	/	**10.54 (4.4, 38.85)**	1.86 (0.36, 10.74)	0.8 (0.04, 15.28)
EVT_201	4.52 (0.69, 109.55)	**SV**	**SV**	/	/	/	/	/	/	/
Flunitrazepam	**3.04 (1.18, 8.27)**	1.48 (0.47, 4.77)	0.88 (0.41, 1.87)	/	/	0.36 (0, 19.75)	/	/	/	/
Flurazepam	**2.52 (1.62, 3.97)**	1.84 (0.48, 6.63)	0.94 (0.51, 1.77)	1.91 (0.04, 60.26)	1.29 (0.02, 53.28)	/	**SV**	**6.81 (1.55, 50.97)**	0.5 (0.09, 2.4)	1.41 (0.07, 27.32)
Gaboxadol	2.16 (0.83, 6.27)	**3.44 (2.01, 6.26)**	1.04 (0.78, 1.38)	/	/	/	/	/	/	/
Indiplon	**3.46 (1.65, 8.59)**	**2.3 (1.12, 5.13)**	**1.63 (1.03, 2.6)**	/	/	/	/	/	/	/
Lemborexant	**6.57 (3.55, 13.43)**	0.57 (0.21, 1.53)	1.24 (0.86, 1.81)	/	/	**13.94 (1.43, 496.72)**	/		/	/
Loprazolam	2.09 (0.95, 4.8)	**SV**	0.93 (0.59, 1.47)	/	/	/	/		/	SV
Lormetazepam	**SV**	**SV**	SV	/	/	/	/		0 (0, 0.27)	0.38 (0, 15.9)
Melatonin	/	/	0.61 (0.25, 1.29)	/	/	/	/	/	/	/
Midazolam	4.91 (0.97, 27.65)	0.4 (0.03, 4.34)	0.61 (0.15, 2.62)	/	/	/	/	0 (0, 0.52)	/	/
Nitrazepam	**3.8 (1.27, 12.51)**	3.25 (0.56, 22.19)	0 (0, 0.36)	/	/	/	**SV**	0 (0, 0.27)	0.67 (0.01, 15.98)	/
Paroxetine	SV	0.61 (0.07, 4.88)	/	/	/	/	/	/	/	/
Ramelteon	**2.19 (1.43, 3.56)**	1.3 (0.76, 2.48)	0.97 (0.75, 1.26)	/	/	/	/	1.22 (0.09, 6.96)	/	/
Seltorexant	0.94 (0.25, 3.74)	0 (0, 0.06)	0.83 (0.41, 1.72)	/	0.33 (0.01, 5.91)	/	/	/	/	/
Suvorexant	**3.32 (2.11, 5.31)**	1.04 (0.54, 2.08)	1.23 (0.87, 1.8)	/	/	/	/	/	/	/
Tasimelteon	**/**	/	1.47 (0.63, 3.95)	/	/	/	/	/	/	/
Temazepam	**3.77 (1.39, 9.78)**	0.37 (0.04, 2.18)	1.04 (0.56, 1.82)	/	/	0.19 (0, 3.33)	0.64 (0.02, 18.95)	/	1.05 (0.13, 9.28)	/
Tiagabine	1.21 (0.26, 9.17)	**4.41 (1.16, 31.75)**	1.36 (0.45, 7.92)	/	/	/	/	/	/	/
Trazodone	**2.86 (1.38, 5.81)**	2.64 (0.98, 7.42)	1.53 (0.95, 2.44)	/	/	/	/	/	/	/
Triazolam	**2.35 (1.3, 4.45)**	**SV**	1.4 (0.95, 2.1)	1.78 (0.01, 364.83)	0 (0, 1.43)	0.24 (0, 6.18)	**SV**	0.29 (0, 10.83)	2.58 (0.74, 12.57)	0 (0, 0.08)
Trimipramine	/	/	2.2 (0.43, 17.89)	/	/	/	/	/	0 (0, 0.17)	0.99 (0.03, 33.88)
Vestipitant	/	/	0.9 (0.29, 2.65)	/	/	/	/	/	/	/
Zaleplon	1.36 (0.76, 2.49)	3.21 (0.99, 11.2)	0.99 (0.45, 2.11)	1.95 (0.25, 20.94)	/	SV	**SV**	1.65 (0.17, 17.19)	0.78 (0.06, 8.94)	0.84 (0.04, 11.57)
Zolpidem	**1.85 (1.36, 2.5)**	**2.33 (1.57, 3.54)**	**1.26 (1.04, 1.48)**	3.76 (0.65, 39.51)	0.94 (0.08, 5.29)	0.22 (0.01, 4.42)	/	0.39 (0.05, 2.14)	1.27 (0.3, 7.48)	0.26 (0.02, 1.78)
Zopiclone	**2.02 (1.11, 3.66)**	**2.33 (1.06, 5.6)**	0.95 (0.63, 1.42)	/	/	/	/	**7.84 (2.29, 55.11)**	SV	/

SV, spurious values. Bold value: Show significantly statistical difference.

**Table 2:** Summary results of network meta-analyses for the most common adverse events: gastrointestinal disorders and general disorders.

Individual drugs	Gastrointestinal disorders							General disorders and administration site conditions	
	Dyspepsia	Diarrhea	Dry mouth	Nausea/vomiting	Constipation	Abdominal pain	Increased appetite	Fatigue	Pain
Almorexant	/	4.33 (0.48, 57.5)	/	1.25 (0.44, 3.52)	/	/		2.46 (0.5, 14.32)	/
Alprazolam	/	/	**SV**	1.35 (0.44, 4.51)	**SV**	/		/	/
Daridorexant	/	1.19 (0.12, 15.66)	/	0.96 (0.42, 2.43)	/	0.27 (0.02, 2.77)		1.74 (0.57, 5.77)	/
Doxepin	0.25 (0.01, 3.13)	0.29 (0.05, 1.3)	1.24 (0.39, 3.01)	1.35 (0.47, 4.73)	0.62 (0.01, 33.1)	/	1.22 (0.07, 20.31)	/	/
Esmirtazapine	/	/	1.39 (0.51, 4.25)	0.98 (0.33, 3.51)	**SV**	/	17.08 (0.8, 967.94)	0.9 (0.16, 5.41)	/
Estazolam	/	/	/	0.75 (0.16, 3.31)	/	/	/	/	/
Eszopiclone	1.14 (0.46, 3.14)	1.08 (0.14, 8.57)	**4.39 (1.76, 12.9)**	1.92 (0.92, 4.26)	/	1.48 (0.28, 8.02)	/	/	1.33 (0.59, 3.42)
EVT_201	/	**SV**	/	/	/	/	/	/	/
Flunitrazepam	/	/	SV	1.28 (0.19, 8.22)	/	/	/	/	/
Flurazepam	/	/	1.15 (0.48, 4.37)	0.11 (0, 0.68)	/	/	/	0 (0, 0.48)	/
Gaboxadol	/	/	/	**3.49 (2, 6.5)**	/	/	/	/	/
Indiplon	/	/	/	**SV**	/	/	/	/	/
Lemborexant	/	/	/	2.15 (0.68, 8.36)	**SV**	**SV**	SV	**17.65 (1.66, 614.75)**	/
Loprazolam	/	/	/	0 (0, 0.4)	/	/	/	**SV**	/
Lormetazepam	/	0 (0, 0.24)	SV	SV	0 (0, 0.28)	/	/	**SV**	/
Melatonin	/	0.95 (0.02, 53.76)	/	/	/	/	/		/
Midazolam	/	/	2.96 (0.34, 34.64)	**11.32 (1.27, 575.35)**	/	/	/	SV	/
Nitrazepam	/	/	0.93 (0.1, 7.61)	0.63 (0.02, 8.99)	/	/	/	SV	/
Paroxetine	/	/	**SV**	0.47 (0.09, 1.99)	**SV**	/	/	/	/
Ramelteon	0 (0, 0.81)	1.58 (0.56, 5.2)	0 (0, 0.15)	1.21 (0.54, 2.83)	**SV**	2.39 (0.32, 29.4)	/	1.88 (0.59, 6.51)	/
Seltorexant	/	0.12 (0, 1.84)	/	0.4 (0.08, 1.88)	/	/	/	0.81 (0.16, 4.5)	/
Suvorexant	**SV**	1.18 (0.25, 5.74)	**2.18 (1.01, 5.22)**	1.14 (0.5, 2.66)	/	/	/	2.23 (0.89, 6.2)	/
Tasimelteon	/	/	/	/	/	/	/		/
Temazepam	0.98 (0.19, 6.15)	1.05 (0.1, 11.16)	/	0.77 (0.22, 2.21)	1.05 (0.01, 79.44)	/	/	7.41 (0.9, 86.9)	/
Tiagabine	/	/	**SV**	**SV**	/	/	/	0.09 (0, 1.74)	/
Trazodone	/	/	/	/	/	/	/	/	/
Triazolam	0.68 (0.17, 3.51)	**SV**	0.92 (0.08, 7.96)	1.82 (0.74, 4.32)	/	/	/	2.61 (0.19, 37.32)	SV
Trimipramine	/	0 (0, 0.22)	**SV**	**SV**	0 (0, 0.12)	/	/	/	/
Vestipitant	/	/	2.42 (0.16, 76.41)	/	/	/	/	1.77 (0.22, 15.86)	/
Zaleplon	2.24 (0.12, 90.71)	/	0 (0, 0.11)	0.18 (0, 1.41)	/	1.83 (0.52, 6.29)	/	/	**SV**
Zolpidem	1.28 (0.36, 6.59)	2.38 (0.67, 11.78)	2.03 (0.62, 7.05)	**1.92 (1.25, 2.99)**	**SV**	1.88 (0.63, 6.92)	SV	1.53 (0.67, 3.8)	0.35 (0.01, 5.31)
Zopiclone	1.31 (0.23, 8.22)	/	1.54 (0.79, 3.47)	1.46 (0.63, 3.38)	/	/	/	SV	/

SV, spurious values. Bold value: Show significantly statistical difference.

**Table 3:** Summary results of network meta-analyses for the most common adverse events: respiratory disorders, psychiatric disorders, and injury.

Individual drugs	Respiratory, thoracic and mediastinal disorders		Psychiatric disorders				Injury, poisoning and procedural complications
	Nasopharyngitis	Respiratory problem	Anxiety	Confusional state	Depression	Emotional lability	Accidental injury
Almorexant	/	/	/	/	/	/	/
Alprazolam	/	/	/	/	/	/	/
Daridorexant	0.35 (0.09, 1.22)	/	/	/	**0 (0, 0.23)**	/	1.85 (0.63, 6.81)
Doxepin	/	/	/	/	/	/	/
Esmirtazapine	1 (0.51, 2.27)	/	**SV**	/	/	/	/
Estazolam	/	/	**/**	/	/	/	/
Eszopiclone	1.5 (0.9, 2.48)	/	**SV**	/	/	**SV**	1.02 (0.52, 2.05)
EVT_201	/	/	/	/	/	/	/
Flunitrazepam	/	/	/	SV	SV	/	/
Flurazepam	1.32 (0.05, 16.49)	/	/	SV	/	/	/
Gaboxadol	1.09 (0.51, 2.12)	/	/	/	/	/	/
Indiplon	/	/	/	/	**SV**	/	/
Lemborexant	1.95 (0.49, 8.96)	/	/	/	/	SV	/
Loprazolam	/	/	/	**SV**	/	/	/
Lormetazepam	/	/	/	/	/	/	/
Melatonin	1.47 (0.35, 7.12)	/	0 (0, 1.06)	/	/	/	/
Midazolam	/	/	/	/	/	/	/
Nitrazepam	/	/	/	**SV**	SV	/	/
Paroxetine	/	/	/	/	/	/	/
Ramelteon	0.96 (0.59, 1.59)	/	/	/	3.81 (0.17, 89.7)	/	/
Seltorexant	/	/	/	/	/	/	/
Suvorexant	0.93 (0.49, 1.8)	/	/	/	/	/	/
Tasimelteon	0.87 (0.21, 4.21)	/	/	/	/	/	/
Temazepam	/	1.06 (0.09, 12.7)	/	**0 (0, 0.09)**	/	2.29 (0.25, 27.97)	/
Tiagabine	/	/	SV	SV	/	/	/
Trazodone	/	/	/	/	/	/	/
Triazolam	3.14 (0.8, 14.94)	0 (0, 8.38)	19.41 (1.09, 1444.1)	SV	SV	/	/
Trimipramine	/	/	/	/	/	/	/
Vestipitant	0 (0, 0.06)	/	/	/	/	/	/
Zaleplon	0.4 (0.14, 1.04)	/	0.5 (0.05, 3.98)	/	/	/	/
Zolpidem	1.08 (0.55, 2.07)	1.92 (0.41, 9.87)	**3.32 (1.15, 20.14)**	SV	0.85 (0.01, 70.39)	1.31 (0.35, 5.08)	/
Zopiclone	/	/	/	SV	SV	**SV**	/

SV, spurious values. Bold value: Show significantly statistical difference.

**Table 4:** Summary results of network meta-analyses for the most common adverse events: musculoskeletal disorders, eye disorders, and infections.

Individual drugs	Musculoskeletal and connective tissue disorders			Eye disorders	Infections and infestations			
	Arthralgia	Back pain	Myalgia	Eye pain	Infection	Upper respiratory tract infection	Urinary tract infection	Sinusitis
Almorexant	/	/	/	/	/	/	/	/
Alprazolam	/	/	/	/	/	/	/	/
Daridorexant	/	/	/	/	/	0.55 (0.19, 1.56)	/	/
Doxepin	/	/	/	/	/	1.11 (0.3, 4.56)	0.25 (0.02, 2.17)	2.01 (0.15, 74.65)
Esmirtazapine	2.43 (0.09, 76.92)	0.85 (0.09, 9.56)	/	/	/	0.82 (0.32, 2.26)	/	4.22 (0.37, 142.42)
Estazolam	/	/	/	/	/	/	/	/
Eszopiclone	3.81 (0.1, 294.62)	1.57 (0.6, 6.3)	2.19 (0.28, 17.28)	/	1.75 (0.83, 3.96)	/	/	0.77 (0.15, 4)
EVT_201	/	/	/	/	/	/	/	/
Flunitrazepam	/	/	/	/	/	/	/	/
Flurazepam	/	0 (0, 3.69)	0.86 (0.02, 14.37)	/	/	/	/	/
Gaboxadol	/	/	/	/	/	0.91 (0.47, 1.67)	/	/
Indiplon	/	/	/	/	/	/	1.18 (0.14, 10.29)	/
Lemborexant	0.98 (0.05, 21.42)	1.96 (0.44, 17.27)	1.89 (0.12, 64.04)	/	/	1.12 (0.5, 2.49)	1.43 (0.33, 7.56)	/
Loprazolam	/	/	/	/	/	/	/	/
Lormetazepam	/	/	/	/	/	/	/	/
Melatonin	/	/	/	/	/	SV	0 (0, 0.49)	/
Midazolam	/	/	/	/	/	/	/	/
Nitrazepam	/	/	/	/	/	/	/	/
Paroxetine	/	/	/	/	/	/	/	/
Ramelteon	/	5.78 (0.47, 225.33)	1.73 (0.16, 7.59)	1.83 (0.46, 9.4)	/	1.27 (0.71, 2.37)	1.06 (0.12, 9.49)	0 (0, 1.47)
Seltorexant	/	/	0.18 (0.01, 1.86)	/	/	/	0.29 (0.02, 4.31)	/
Suvorexant	/	0.53 (0.12, 2.32)	/	/	/	1.51 (0.56, 4.49)	0.49 (0.1, 2.45)	/
Tasimelteon	/	/	/	/	/	/	4.2 (0.26, 188.6)	/
Temazepam	0 (0, 0.14)	/	0.97 (0.16, 7)	/	/	0.89 (0.27, 2.86)	/	/
Tiagabine	/	/	/	/	/	/	/	/
Trazodone	/	/	/	/	/	/	/	/
Triazolam	1.76 (0.07, 48.24)	/	0.83 (0.13, 6)	/	/	0.23 (0.03, 1.03)	**0 (0, 0.6)**	/
Trimipramine	/	/	/	/	/	/	/	/
Vestipitant	/	/	/	/	/	/	/	/
Zaleplon	/	1.96 (0.27, 19.34)	1.43 (0.25, 10.02)	2.69 (0.5, 17.76)	1.15 (0.28, 5.48)	/	**SV**	/
Zolpidem	1.44 (0.05, 40.92)	3.29 (0.58, 32.93)	1.11 (0.44, 3.63)	2.51 (0.42, 17.96)	2.39 (0.55, 11.76)	0.71 (0.25, 1.86)	0.58 (0.13, 2.32)	4.32 (0.68, 32.77)
Zopiclone	/	/	/	/	/	/	/	/

SV, spurious values.

### Nervous system disorders

Eighty-seven trials including 30,107 participants and 32 drugs reported nervous system disorders, of which, the most commonly reported AEs were headache (74 trials), followed by somnolence and dizziness (62 trials). Overall, compared with placebo (**Table 1**), esmirtazapine, estazolam, eszopiclone, flunitrazepam, flurazepam, indiplon, nitrazepam, ramelteon, lemborexant, suvorexant, temazepam, trazodone, triazolam, zolpidem, and zopiclone were associated with an increased risk of somnolence; esmirtazapine, eszopiclone, gaboxadol, indiplon, tiagabine, zolpidem, and zopiclone were associated with an increased risk of dizziness; daridorexant, indiplon, and zolpidem were associated with an increased risk of headache; lemborexant was associated with an increased risk of nightmare; and eszopiclone, flurazepam, and zopiclone were associated with an increased risk of dysgeusia. In contrast, doxepin was associated with lower risk of headache and somnolence than placebo or/and most of other drugs like eszopiclone, gaboxadol, indiplon, lemborexant, ramelteon, suvorexant, tasimelteon, triazolam, and zolpidem. Although higher risk in some outcomes of nervous system disorders were observed for flurazepam, nitrazepam, triazolam, and zaleplon compared with placebo, those estimates were mainly based on one small study with rare events and thus had high uncertainty. Among comparisons across active drugs, risks of AEs were mostly observed in the comparison of a harmful drug with an unharmful drug (**Appendix 11**). Indiplon, lemborexant, ramelteon, suvorexant, and trazodone showed higher risks of both somnolence and headache than doxepin; eszopiclone and lemborexant showed a higher risk of somnolence than daridorexant; both esmirtazapine, eszopiclone, and gaboxadol showed a higher risk of somnolence than lemborexant and suvorexant. Compared with several other drugs that performed worse than placebo (**Appendix 11**), esmirtazapine and lemborexant had the highest risk of somnolence, and daridorexant the highest risk of headache. We found no associations between any drugs and other nervous system disorders including amnesia, difficulty concentrating, and nervousness (**Table 1**, **Appendix 11**).

For secondary outcomes (**Appendix 11**), the associations for abnormal dreams, abnormal vision, asthenia, gait disturbance, hypnagogic hallucinations, memory disorders, and tremor for any drug compared with placebo were highly uncertain.

### Gastrointestinal disorders

Sixty-eight trials including 24,595 participants and 32 drugs reported gastrointestinal disorders, of which, the most commonly reported AEs were nausea/vomiting (45 studies), followed by dry mouth (30 studies) and diarrhea (23 studies). When compared with placebo (**Table 2**), eszopiclone and suvorexant were associated with an increased risk of dry mouth; and gaboxadol, midazolam, and zolpidem were associated with an increased risk of nausea/vomiting. Other drugs like alprazolam, esmirtazapine, lemborexant, paroxetine, EVT 201, ramelteon, and lemborexant showed encouraging point estimates in some outcomes of gastrointestinal disorders but with low statistical power because of the rare event and the limited number of studies and participants included. Among comparisons across active drugs (**Appendix 11**), gaboxadol was associated with a higher risk of nausea/vomiting than ramelteon and suvorexant. We found no associations between drugs and increased appetite (**Table 2**, **Appendix 11**).

For secondary outcomes (**Appendix 11**), because of the limited number of studies with rare event the results for indiplon, doxepin, and triazolam in increased risks of decreased appetite and gastroenteritis were highly uncertain.

### General disorders and administration site conditions

Thirty-eight trials including 21,318 participants and 20 drugs reported on general disorders and administration site conditions, of which, the most commonly reported AE was fatigue (25 trials). When compared with placebo (**Table 2**), lemborexant, loprazolam, and lormetazepam were associated with an increased risk of fatigue, and zaleplon was associated with an increased risk of pain. However, these estimates were based on a limited number of studies that with rare events and small sample size, and thus are highly uncertain.

We found no associations between the drugs and the secondary outcomes belonging to general disorders and administration site conditions, such as common cold, influenza, and malaise.

### Respiratory, thoracic and mediastinal disorders

Thirty-one trials including 17,893 participants and 16 drugs reported respiratory, thoracic and mediastinal disorders, of which, the most commonly reported AE was nasopharyngitis (27 trials). We found no associations between any drugs and nasopharyngitis or respiratory problems (**Table 3**). Although the lowest risk compared with placebo and other drugs was observed for vestipitant in nasopharyngitis, the estimate was based on one small study with 80 participants and thus was highly uncertain.

For secondary outcomes (**Appendix 11**), the lowest risk and highest risk compared with placebo and other drugs were observed for ramelteon and EVT 201 respectively in cough, but these estimates were also based on one small study, respectively, and thus had high uncertainty.

### Psychiatric disorders

Thirty trials including 13,115 participants and 18 drugs reported psychiatric disorders, of which, the most commonly reported AE was anxiety (11 trials). When compared with placebo (**Table 3**), zolpidem was associated with an increased risk of anxiety. Although esmirtazapine, eszopiclone, indiplon, loprazolam, nitrazepam, and zopiclone were associated with higher risk of anxiety than placebo, these estimates were based on one small study respectively and thus was highly uncertain (**Table 3**).

For secondary outcomes, we found no associations between drugs and irritability (**Appendix 11**). The highest risk compared with placebo was observed for daridorexant in suicidal ideation, however the results were uncertain with wide CrIs. We did not find an association between daridorexant and hallucinations in the pairwise meta-analysis.

### Injury, poisoning and procedural complications

Ten trials including 7,442 participants and six drugs reported injury, poisoning and procedural complications. We found no associations between any drugs and accidental injury (**Table 3**). For secondary outcomes (**Appendix 11**), although doxepin was associated with a higher risk than placebo, suvorexant, and melatonin with doxepin in falls and laceration, that estimate was based on one small study with rare event and thus comes with high uncertainty.

### Musculoskeletal and connective tissue disorders

Eighteen trials including 13,912 participants and 12 drugs reported musculoskeletal and connective tissue disorders. The most commonly reported AE was back pain (13 trials). We found no association between the drugs and arthralgia, back pain, or myalgia (**Table 4**). Although temazepam showed the lowest risk compared to other drugs in arthralgia, this estimate was based on one small study with a sample size of 84, thus having high uncertainty.

### Eye disorders

Five trials including 1,770 participants and five drugs reported on eye disorders, of which, the most commonly reported AE was eye pain (three trials). We found no associations between drugs and eye pain or dry eyes (**Table 4**).

### Infections and infestations

Thirty-two trials including 23,366 participants and 16 drugs reported infections and infestations, of which, the most commonly reported AE was upper respiratory tract infection (22 trials). When compared with placebo (**Table 4**), there were no associations between drug and infection, upper respiratory tract infection, and sinusitis. Zaleplon and triazolam showed the highest and lowest risk, respectively, compared to placebo in urinary tract infection, but those results were mainly from one small study, respectively, and thus highly uncertain. Compared to triazolam, ramelteon and suvorexant could significantly increase the risk of upper respiratory tract infection (**Appendix 11**).

### Other disorders

For other secondary outcomes, we found no association between the drugs and the risk of insomnia exacerbated, dysmenorrhea, or tachycardia (**Appendix 11**). Hematuria was specific to melatonin, and paired meta-analysis showed that there was no association between melatonin and risk of hematuria when compared to placebo. Although some drugs showed higher risk of sleep paralysis, pruritis, skin diseases, sweating, peripheral edema, alanine aminotransferase increased, blood creatine phosphokinase increased, γ-Glutamyl transferase increased, hyperglycemia, and hypertension, these results were based on a limited number of studies with rare events and thus had high uncertainty (**Appendix 11**). Meanwhile, we found that esmirtazapine was associated with a weight increase (**Appendix 11**).

### Sensitivity analysis

The sensitivity analysis using Mantel-Haenszel network meta-analysis suggested that most results were similar to the primary analysis (**Appendix 13**). However, in the Mantel-Haenszel network meta-analysis, when compared with placebo, eszopiclone was associated with increased risks of nausea/vomiting, nasopharyngitis, and infection; esmirtazapine was associated with an increased risk of increased appetite; suvorexant and temazepam with an increased risk of fatigue; and ramelteon with an increased risk of depression (**Appendix 13**). Suvorexant and seltorexant showed lower risks of urinary tract infection and myalgia (**Appendix 13**) when compared with placebo.

## Discussion

This is the first largest systematic review with network analysis included 102 randomized trials involving 35,700 participants to examine the association between 33 insomnia drugs and the risks of adverse events in patients with primary insomnia. Zolpidem significantly increased the risks of nervous system disorders (somnolence, dizziness, and headache), gastrointestinal disorders (nausea/vomiting), and psychiatric disorders (anxiety), however, no increased risks of other AEs was found. Zopiclone was mainly associated with nervous system disorders such as somnolence, dizziness, and dysgeusia. Indiplon was also associated with elevated risk of somnolence, dizziness, and headache. Gaboxadol significantly increased the risks of nervous system disorders and gastrointestinal disorders (dizziness and nausea/vomiting). Eszopiclone was associated with nervous system disorders and gastrointestinal disorders (somnolence, dizziness, and dry mouth). Esmirtazapine could also increase the risk of nervous system disorders (somnolence and dizziness). Estazolam, flunitrazepam, flurazepam, nitrazepam, ramelteon, suvorexant, trazodone, and Triazolam were found to increase the risk of somnolence, tiagabine was also found to be associated with the increased risk of dizziness. Doxepin was associated with lower risk of headache and somnolence than placebo and most of other drugs. We observed no associations between any drugs and the risks of nasopharyngitis, respiratory problems, accidental injury, infection, upper respiratory tract infection, sinusitis, and hematuria. For outcomes for abnormal dreams, abnormal vision, asthenia, gait disturbance, hypnagogic hallucinations, memory disorders, decreased appetite, cough, and suicidal ideation, the risks were still highly uncertain. In terms of flurazepam, nitrazepam, triazolam, and zaleplon, although higher risks compared with placebo were observed for in some outcomes, those estimates were based on a limited number of studies with rare events. The data from the included trials also emphasized that certain adverse events might be specific to certain drugs (e.g., common cold to doxepin and zolpidem; hallucinations to daridorexant; laceration to doxepin and melatonin; and hematuria to melatonin).

### Strengths and limitations of this study

This systematic review had several strengths. This is to our knowledge the first network meta-analysis to comprehensively investigate the safety of a large number of drugs for patients with primary insomnia. We used a sensitive and systematic search strategy to identify eligible trials, and conducted the assessment of risk of bias and data extraction in duplicate. We conducted this systematic review and meta-analysis according to the PRISMA Extension Statement for Reporting of Systematic Reviews Incorporating Network Meta-analyses of Health Care Interventions (12).

Despite the above strengths, this review also had some limitations. First, the results regarding some drugs were underpowered because of the limited sample sizes and generally low incidence of AEs in included studies. Second, the primary purpose for most of reviews and all of included trials was to assess the efficacy of insomnia drugs, and data on AEs was not always well-documented. Third, the results had a serious risk of bias because of inadequate information about randomization. Fourth, one notable limitation of our study is the insufficient phenotyping of patients in the included trials. Many studies did not provide detailed information regarding insomnia subtype, duration, severity, or relevant demographic and clinical characteristics. These factors could influence adverse event profiles and treatment responses. Future research should prioritize more comprehensive patient characterization to enhance the clinical applicability of safety assessments. Finally, although our review systematically evaluates adverse events associated with pharmacological treatments for insomnia, its immediate applicability to clinical practice may be influenced by real-world prescribing and patient experiences. For instance, in everyday clinical settings, the most common reason for discontinuing Zopiclone or Eszopiclone is the presence of an unpleasant bitter taste, which was not a primary focus in the included studies. This highlights the need for future studies to consider patient-reported experiences and real-world medication adherence factors.

### Comparison with other studies

Previously published systematic reviews and meta-analyses have generally focused on the efficacy as their primary outcome as well as tolerability and overall risk of AEs (7, 25). Individual AEs have usually not been assessed in the currently available systematic reviews. To our knowledge, this systematic review is the first to compare all reported specific AEs on 33 individual drugs for primary insomnia. In contrast with previous systematic reviews, this study also indirect in addition to direct evidence, which can increase the amount evidence regarding the safety of primary insomnia drugs. In addition, this review included substantially more trials - including very recently published trials and unpublished trials - and patients than previous meta-analyses. Previous systematic reviews found that ramelteon, lemborexant, eszopiclone, and suvorexant were associated with increased risks of somnolence (8, 25, 26), which was similar with our findings. Xue and collogues (6) conducted a network meta-analysis to compare the efficacy and safety of dual orexin receptor antagonists in primary insomnia, they found that dual orexin receptor antagonists (include suvorexant, almorexant, filorexant, Lemborexant, and daridorexant) were associated with higher risks of somnolence, abnormal dreams, fatigue, and dry mouth when compared to placebo. However, our network meta-analysis only supported the association between lemborexant and suvorexant and the risk of somnolence; suvorexant and the risk of dry mouth; and lemborexant and the risk of fatigue. We did not find any associations between dual orexin receptor antagonists and the risk of abnormal dreams. The AASM guideline 10 concluded that the side effects of benzodiazepines outweigh the treatment effects. Results from meta-analyses also showed that benzodiazepine treatment had a higher risk of AEs (27, 28), and the side effects of benzodiazepines were more common relative to benzodiazepines receptor agonistic modulators (BzRAs) (28). However, a recent network meta-analysis (7) found that eszopiclone, zolpidem, and zopiclone had more side-effects reported. These findings were consistent with ours, according to which zolpidem, zopiclone, and eszopiclone significantly increased the risks of nervous system disorders, gastrointestinal disorders, and psychiatric disorders.

## Conclusion

Overall, zolpidem, zopiclone, indiplon, gaboxadol, eszopiclone, and esmirtazapine were associated with a higher risk of adverse events, especially in somnolence, dizziness, headache, dysgeusia, and nausea/vomiting. The number of studies was limited for some drugs like flurazepam, nitrazepam, triazolam, and zaleplon in some outcomes and thus did not allow to draw firm conclusions. Doxepin was associated with a lower risk of headache and somnolence than placebo or/and most of other drugs. We observed no associations between drugs and the risk of serious AEs including nasopharyngitis, respiratory problem, accidental injury, infection, upper respiratory tract infection, sinusitis, and hematuria. In terms of outcomes for abnormal dreams, abnormal vision, asthenia, gait disturbance, hypnagogic hallucinations, memory disorders, decreased appetite, cough, and suicidal ideation, the risk association was still highly uncertain. In conclusion, the findings of this network meta-analysis can present the best evidence basis that is currently available to determine the trade-off between the benefits and harms of hypnotic medications in patients with primary insomnia, and to inform treatment guidelines and clinical care. Ultimately, while meta-analyses provide essential insights into the safety profile of pharmacological interventions, clinical decision-making must remain individualized. Clinicians should carefully balance the therapeutic benefits of insomnia medications with potential adverse effects, tailoring treatment decisions based on patient-specific factors and real-world tolerability.

## Author’s note

The manuscript’s guarantors affirm that the manuscript is an honest, accurate, and transparent account of the study being reported; that no important aspects of the study have been omitted; and that any discrepancies from the study as planned (and, if relevant, registered) have been explained.

## Data Availability

The original contributions presented in the study are included in the article/**Supplementary Material**. Further inquiries can be directed to the corresponding author.

## References

[1] Mukherjee S Patel SR Kales SN Ayas NT Strohl KP Gozal D . An official American Thoracic Society statement: the importance of healthy sleep. Recommendations and future priorities. Am J Respir Crit Care Med. (2015) 191:1450–8. doi: 10.1164/rccm.201504-0767ST, PMID: 26075423 PMC5442970

[2] Walsh JK Coulouvrat C Hajak G Lakoma MD Petukhova M Roth T . Nighttime insomnia symptoms and perceived health in the America Insomnia Survey (AIS). Sleep. (2011) 34:997–1011. doi: 10.5665/SLEEP.1150, PMID: 21804662 PMC3138174

[3] Maust DT Solway E Clark SJ Kirch M Singer DC Malani P . Prescription and nonprescription sleep product use among older adults in the United States. Am J Geriatr Psychiatry. (2019) 27:32–41. doi: 10.1016/j.jagp.2018.09.004, PMID: 30409547

[4] Abraham O Pu J Schleiden LJ Albert SM . Factors contributing to poor satisfaction with sleep and healthcare seeking behavior in older adults. Sleep Health. (2017) 3:43–8. doi: 10.1016/j.sleh.2016.11.004, PMID: 28346150 PMC10124133

[5] Ford ES Wheaton AG Cunningham TJ Giles WH Chapman DP Croft JB . Trends in outpatient visits for insomnia, sleep apnea, and prescriptions for sleep medications among US adults: findings from the National Ambulatory Medical Care survey 1999-2010. Sleep. (2014) 37:1283–93. doi: 10.5665/sleep.3914, PMID: 25083008 PMC4096197

[6] Xue T Wu X Chen S Yang Y Yan Z Song Z . The efficacy and safety of dual orexin receptor antagonists in primary insomnia: A systematic review and network meta-analysis. Sleep Med Rev. (2022) 61:101573. doi: 10.1016/j.smrv.2021.101573, PMID: 34902823

[7] De Crescenzo F D'Alò GL Ostinelli EG Ciabattini M Di Franco V Watanabe N . Comparative effects of pharmacological interventions for the acute and long-term management of insomnia disorder in adults: a systematic review and network meta-analysis. Lancet. (2022) 400:170–84. doi: 10.1016/S0140-6736(22)00878-9, PMID: 35843245

[8] Kuriyama A Honda M Hayashino Y . Ramelteon for the treatment of insomnia in adults: a systematic review and meta-analysis. Sleep Med. (2014) 15:385–92. doi: 10.1016/j.sleep.2013.11.788, PMID: 24656909

[9] Glass J Lanctôt KL Herrmann N Sproule BA Busto UE . Sedative hypnotics in older people with insomnia: meta-analysis of risks and benefits. BMJ. (2005) 331:1169. doi: 10.1136/bmj.38623.768588.47, PMID: 16284208 PMC1285093

[10] Sateia MJ Buysse DJ Krystal AD Neubauer DN Heald JL . Clinical practice guideline for the pharmacologic treatment of chronic insomnia in adults: an American academy of sleep medicine clinical practice guideline. J Clin Sleep Med. (2017) 13:307–49. doi: 10.5664/jcsm.6470, PMID: 27998379 PMC5263087

[11] Solmi M Fornaro M Ostinelli EG Zangani C Croatto G Monaco F . Safety of 80 antidepressants, antipsychotics, anti-attention-deficit/hyperactivity medications and mood stabilizers in children and adolescents with psychiatric disorders: a large scale systematic meta-review of 78 adverse effects. World Psychiatry. (2020) 19:214–32. doi: 10.1002/wps.20765, PMID: 32394557 PMC7215080

[12] Hutton B Salanti G Caldwell DM Chaimani A Schmid CH Cameron C . The PRISMA extension statement for reporting of systematic reviews incorporating network meta-analyses of health care interventions: checklist and explanations. Ann Intern Med. (2015) 162:777–84. doi: 10.7326/M14-2385, PMID: 26030634

[13] Ouzzani M Hammady H Fedorowicz Z Elmagarmid A . Rayyan-a web and mobile app for systematic reviews. Syst Rev. (2016) 5:210. doi: 10.1186/s13643-016-0384-4, PMID: 27919275 PMC5139140

[14] Lankford A Rogowski R Essink B Ludington E Heith Durrence H Roth T . Efficacy and safety of doxepin 6 mg in a four-week outpatient trial of elderly adults with chronic primary insomnia. Sleep Med. (2012) 13:133–8. doi: 10.1016/j.sleep.2011.09.006, PMID: 22197474

[15] Ivgy-May N Hajak G van Osta G Braat S Chang Q Roth T . Efficacy and safety of esmirtazapine in adult outpatients with chronic primary insomnia: a randomized, double-blind placebo-controlled study and open-label extension. J Clin Sleep Med. (2020) 16:1455–67. doi: 10.5664/jcsm.8526, PMID: 32351205 PMC7970588

[16] Kärppä M Yardley J Pinner K Filippov G Zammit G Moline M . Long-term efficacy and tolerability of lemborexant compared with placebo in adults with insomnia disorder: results from the phase 3 randomized clinical trial SUNRISE 2. Sleep. (2020) 43:zsaa123. doi: 10.1093/sleep/zsaa123, PMID: 32585700 PMC7487867

[17] National Institutes of Health . Available online at: https://ctep.cancer.gov/protocoldevelopment/electronic_applications/docs/CTCAE_v5_Quick_Reference_8.5x11.pdf (Accessed March 09, 2023).

[18] Evidence Partners . Methodological resources (2011). Available online at: https://www.evidencepartners.com/resources/methodological-resources/. (Accessed March 12, 2023).

[19] Higgins JPT Thomas J Chandler J Cumpston M Li T Page MJ . Cochrane handbook for systematic reviews of interventions version 5.1. 0. The Cochrane Collaboration (2011). (Accessed March 2011).

[20] Ades AE Sculpher M Sutton A Abrams K Cooper N Welton N . Bayesian methods for evidence synthesis in cost-effectiveness analysis. Pharmacoeconomics. (2006) 24:1–19. doi: 10.2165/00019053-200624010-00001, PMID: 16445299

[21] Lumley T . Network meta-analysis for indirect treatment comparisons. Stat Med. (2002) 21:2313–24. doi: 10.1002/sim.v21:16 12210616

[22] Dias S Sutton AJ Welton NJ Ades AE . Evidence synthesis for decision making 3: heterogeneity-subgroups, meta-regression, bias, and bias-adjustment. Med Decis Making. (2013) 33:618–40. doi: 10.1177/0272989X13485157, PMID: 23804507 PMC3704206

[23] van Valkenhoef G Dias S Ades AE Welton NJ . Automated generation of node-splitting models for assessment of inconsistency in network meta-analysis. Res Synth Methods. (2016) 7:80–93. doi: 10.1002/jrsm.1167 26461181 PMC5057346

[24] Efthimiou O Rücker G Schwarzer G Higgins JPT Egger M Salanti G . Network meta-analysis of rare events using the Mantel-Haenszel method. Stat Med. (2019) 38:2992–3012. doi: 10.1002/sim.v38.16, PMID: 30997687

[25] Kishi T Nomura I Matsuda Y Sakuma K Okuya M Ikuta T . Lemborexant vs suvorexant for insomnia: A systematic review and network meta-analysis. J Psychiatr Res. (2020) 128:68–74. doi: 10.1016/j.jpsychires.2020.05.025, PMID: 32531478

[26] Rösner S Englbrecht C Wehrle R Hajak G Soyka M . Eszopiclone for insomnia. Cochrane Database Syst Rev. (2018) 10:CD010703. doi: 10.1002/14651858.CD010703.pub2, PMID: 30303519 PMC6492503

[27] Holbrook AM Crowther R Lotter A Cheng C King D . Meta-analysis of benzodiazepine use in the treatment of insomnia. CMAJ. (2000) 162:225–33. PMC1232276 10674059

[28] Buscemi N Vandermeer B Friesen C Bialy L Tubman M Ospina M . The efficacy and safety of drug treatments for chronic insomnia in adults: a meta-analysis of RCTs. J Gen Intern Med. (2007) 22:1335–50. doi: 10.1007/s11606-007-0251-z, PMID: 17619935 PMC2219774

