# Supplementary material for: Adverse events of pharmacological interventions for insomnia disorder in adults: a systematic review and network meta-analysis
Source: Front Psychiatry. 2025 Jul 9;16:1461166. doi: 10.3389/fpsyt.2025.1461166 (PMC12283787; doi:10.3389/fpsyt.2025.1461166)

Association between insomnia drugs and adverse events in adults: a systematic review and network meta-analysis

Appendix 1 PRISMA NMA Checklist of Items to Include When Reporting A Systematic Review Involving a Network Meta-analysis ... 4

Appendix 2 Search strategy ... 8

Appendix 3 Study eligibility form ... 12

Appendix 4 Bassline characteristics of included trails ... 13

Appendix 5 Detail drug information ... 20

Appendix 6 The incidence of different adverse events ... 26

The incidence of gastrointestinal disorders ... 26

The incidence of general disorders and administration site conditions ... 27

The incidence of respiratory, thoracic and mediastinal disorders ... 28

The incidence of psychiatric disorders ... 29

The incidence of injury, poisoning and procedural complications ... 30

The incidence of musculoskeletal and connective tissue disorders ... 31

The incidence of eye disorders ... 32

The incidence of infections and infestations ... 33

The incidence of sleeping problem ... 34

The incidence of investigations ... 35

The incidence of skin and subcutaneous tissue disorders ... 37

The incidence of other adverse events ... 38

Appendix 7 Principle for risk of bias assessment ... 39

Appendix 8 Results of risk of bias assessment ... 42

Appendix 9 The network evidence plots for each outcome ... 46

The network evidence plots for primary outcomes 46

The network evidence plots for secondary outcomes 63

Appendix 10 Results of pairwise meta-analysis 79

Results of pairwise meta-analysis for primary outcomes 79

Results of pairwise meta-analysis for secondary outcomes 102

Appendix 11 Results of network meta-analysis 111

Results of network meta-analysis for primary outcomes 111

Results of network meta-analysis for secondary outcomes 131

Appendix 12 Results of inconsistency between direct and indirect comparisons 147

Appendix 13 Results of sensitivity analysis 156

Results of Mantel-Haenszel network meta-analyses 156

Appendix 14 Results of posterior mean of the overall residual deviance for all outcomes 182

Appendix 15 Reference list of included studies 184

Appendix 16 The results of small-study effects bias 192

Appendix 1 PRISMA NMA Checklist of Items to Include When Reporting A Systematic Review Involving a Network Meta-analysis

Section/Topic	Item #	Checklist Item	Reported on Page #
**TITLE**			
Title	1	Identify the report as a systematic review *incorporating a network meta-analysis (or related form of meta-analysis).*	***1***
**ABSTRACT**			
Structured summary	2	Provide a structured summary including, as applicable:<br>**Background:** main objectives<br>**Methods:** data sources; study eligibility criteria, participants, and interventions; study appraisal; and *synthesis methods, such as network meta-analysis.*<br>**Results:** number of studies and participants identified; summary estimates with corresponding confidence/credible intervals; *treatment rankings may also be discussed. Authors may choose to summarize pairwise comparisons against a chosen treatment included in their analyses for brevity.*<br>**Discussion/Conclusions:** limitations; conclusions and implications of findings.<br>**Other:** primary source of funding; systematic review registration number with registry name.	2
**INTRODUCTION**			
Rationale	3	Describe the rationale for the review in the context of what is already known, *including mention of why a network meta-analysis has been conducted.*	***5***
Objectives	4	Provide an explicit statement of questions being addressed, with reference to participants, interventions, comparisons, outcomes, and study design (PICOS).	5
**METHODS**			
Protocol and registration	5	Indicate whether a review protocol exists and if and where it can be accessed (e.g., Web address); and, if available, provide registration information, including registration number.	6
Eligibility criteria	6	Specify study characteristics (e.g., PICOS, length of follow-up) and report characteristics (e.g., years considered, language, publication status) used as criteria for eligibility, giving rationale. *Clearly describe eligible treatments included in the treatment network, and note whether any have been clustered or merged into the same node (with*	***6***

		justification).	
Information sources	7	Describe all information sources (e.g., databases with dates of coverage, contact with study authors to identify additional studies) in the search and date last searched.	6
Search	8	Present full electronic search strategy for at least one database, including any limits used, such that it could be repeated.	6
Study selection	9	State the process for selecting studies (i.e., screening, eligibility, included in systematic review, and, if applicable, included in the meta-analysis).	7
Data collection process	10	Describe method of data extraction from reports (e.g., piloted forms, independently, in duplicate) and any processes for obtaining and confirming data from investigators.	7
Data items	11	List and define all variables for which data were sought (e.g., PICOS, funding sources) and any assumptions and simplifications made.	7
**Geometry of the network**	**S1**	Describe methods used to explore the geometry of the treatment network under study and potential biases related to it. This should include how the evidence base has been graphically summarized for presentation, and what characteristics were compiled and used to describe the evidence base to readers.	***7***
Risk of bias within individual studies	12	Describe methods used for assessing risk of bias of individual studies (including specification of whether this was done at the study or outcome level), and how this information is to be used in any data synthesis.	7
Summary measures	13	State the principal summary measures (e.g., risk ratio, difference in means). *Also describe the use of additional summary measures assessed, such as treatment rankings and surface under the cumulative ranking curve (SUCRA) values, as well as modified approaches used to present summary findings from meta-analyses.*	7-8
Planned methods of analysis	14	Describe the methods of handling data and combining results of studies for each network meta-analysis. This should include, but not be limited to:<br>• *Handling of multi-arm trials;*<br>• *Selection of variance structure;*<br>• *Selection of prior distributions in Bayesian analyses; and*<br>• *Assessment of model fit.*	7-8
**Assessment of Inconsistency**	**S2**	Describe the statistical methods used to evaluate the agreement of direct and indirect evidence in the treatment network(s) studied. Describe efforts taken to address its presence when found.	7-8
Risk of bias across studies	15	Specify any assessment of risk of bias that may affect the cumulative evidence (e.g., publication bias, selective reporting within studies).	7-8

Additional analyses	16	Describe methods of additional analyses if done, indicating which were pre-specified. This may include, but not be limited to, the following:<br>• Sensitivity or subgroup analyses;<br>• Meta-regression analyses;<br>• *Alternative formulations of the treatment network; and*<br>• *Use of alternative prior distributions for Bayesian analyses (if applicable).*	7-8
**RESULTS†**			
Study selection	17	Give numbers of studies screened, assessed for eligibility, and included in the review, with reasons for exclusions at each stage, ideally with a flow diagram.	9
**Presentation of network structure**	**S3**	Provide a network graph of the included studies to enable visualization of the geometry of the treatment network.	***9***
**Summary of network geometry**	**S4**	Provide a brief overview of characteristics of the treatment network. This may include commentary on the abundance of trials and randomized patients for the different interventions and pairwise comparisons in the network, gaps of evidence in the treatment network, and potential biases reflected by the network structure.	***9***
Study characteristics	18	For each study, present characteristics for which data were extracted (e.g., study size, PICOS, follow-up period) and provide the citations.	9
Risk of bias within studies	19	Present data on risk of bias of each study and, if available, any outcome level assessment.	9
Results of individual studies	20	For all outcomes considered (benefits or harms), present, for each study: 1) simple summary data for each intervention group, and 2) effect estimates and confidence intervals. *Modified approaches may be needed to deal with information from larger networks.*	***10-12***
Synthesis of results	21	Present results of each meta-analysis done, including confidence/credible intervals. *In larger networks, authors may focus on comparisons versus a particular comparator (e.g. placebo or standard care), with full findings presented in an appendix. League tables and forest plots may be considered to summarize pairwise comparisons.* If additional summary measures were explored (such as treatment rankings), these should also be presented.	***10-12***
**Exploration for inconsistency**	**S5**	Describe results from investigations of inconsistency. This may include such information as measures of model fit to compare consistency	***10-12***

		and inconsistency models, *P* values from statistical tests, or summary of inconsistency estimates from different parts of the treatment network.	
Risk of bias across studies	22	Present results of any assessment of risk of bias across studies for the evidence base being studied.	***10-12***
Results of additional analyses	23	Give results of additional analyses, if done (e.g., sensitivity or subgroup analyses, meta-regression analyses, *alternative network geometries studied, alternative choice of prior distributions for Bayesian analyses,* and so forth).	***10-12***
**DISCUSSION**			
Summary of evidence	24	Summarize the main findings, including the strength of evidence for each main outcome; consider their relevance to key groups (e.g., healthcare providers, users, and policy-makers).	12
Limitations	25	Discuss limitations at study and outcome level (e.g., risk of bias), and at review level (e.g., incomplete retrieval of identified research, reporting bias). *Comment on the validity of the assumptions, such as transitivity and consistency. Comment on any concerns regarding network geometry (e.g., avoidance of certain comparisons).*	13
Conclusions	26	Provide a general interpretation of the results in the context of other evidence, and implications for future research.	15
**FUNDING**			
Funding	27	Describe sources of funding for the systematic review and other support (e.g., supply of data); role of funders for the systematic review. This should also include information regarding whether funding has been received from manufacturers of treatments in the network and/or whether some of the authors are content experts with professional conflicts of interest that could affect use of treatments in the network.	***15***

PICOS = population, intervention, comparators, outcomes, study design.

* Text in italics indicateS wording specific to reporting of network meta-analyses that has been added to guidance from the PRISMA statement.

† Authors may wish to plan for use of appendices to present all relevant information in full detail for items in this section.

Appendix 2 Search strategy

Database: *PubMed*

Search Strategy:

#	Searches
#1	"Dyssomnias"[Mesh] OR "Wakefulness"[Mesh] OR "Sleep Initiation and Maintenance Disorders"[Mesh] OR "Sleep Wake Disorders"[Mesh]) OR "Sleep Deprivation"[Mesh]) OR insomn*[Title/Abstract]
#2	"Randomized Controlled Trial" [Publication Type] OR "Controlled Clinical Trial"[Publication Type] OR randomized [Title/Abstract] OR randomised [Title/Abstract] OR randomly [Title/Abstract] OR random* [Title/Abstract]
#3	"Hypnotics and Sedatives"[Mesh] OR "Benzodiazepines"[Mesh]) OR "Antidepressive Agents"[Mesh]
#4	antidepress*[Title/Abstract] OR “anti depress*”[Title/Abstract] OR anti-depress*[Title/Abstract] OR doxepin[Title/Abstract] OR mirtazapine[Title/Abstract] OR trazodone[Title/Abstract] OR amitriptyline[Title/Abstract]
#5	Diphenhydramine [Title/Abstract] OR doxylamine [Title/Abstract] OR gabapentin [Title/Abstract] OR pregabalin
#6	orexin[Title/Abstract] OR suvorexant[Title/Abstract]
#7	antipsycho* [Title/Abstract] OR anti-psycho* [Title/Abstract] OR olanzapine [Title/Abstract] OR quetiapine [Title/Abstract]
#8	Melatonin [Title/Abstract] OR N-acetyl-5-methoxytryptamine [Title/Abstract] OR Ramelteon [Title/Abstract] OR Rozerem [Title/Abstract] OR melaton* [Title/Abstract]
#9	Benzodiazepine [Title/Abstract] OR “benzodiazepine tranquillizers” [Title/Abstract]) OR clonazepam [Title/Abstract] OR triazolam [Title/Abstract] OR temazepam [Title/Abstract] OR estazolam [Title/Abstract] OR quazepam [Title/Abstract] OR flurazepam [Title/Abstract] OR alprazolam [Title/Abstract] OR lorazepam [Title/Abstract] OR clonazepam [Title/Abstract] OR benzodiaz* [Title/Abstract] OR brotizolam [Title/Abstract] OR diazepam [Title/Abstract] OR flunitrazepam [Title/Abstract] OR haloxazolam [Title/Abstract] OR loprazolam [Title/Abstract] OR lormetazepam [Title/Abstract] OR midazolam [Title/Abstract] OR nimetazepam [Title/Abstract] OR nitrazepam [Title/Abstract] OR oxazepam [Title/Abstract] OR rilmazafone [Title/Abstract] OR temazepam [Title/Abstract] OR triazolam [Title/Abstract]
#10	nonbenzodiazepin* [Title/Abstract] OR “non benzodiazepin*” [Title/Abstract] OR non-benzodiazepin* [Title/Abstract] OR “Z drug*” [Title/Abstract] OR eszopiclone [Title/Abstract] OR zaleplon [Title/Abstract] OR zolpidem [Title/Abstract] OR imidazopyridin* [Title/Abstract] OR cyclopyrrolon* [Title/Abstract] OR eszopiclon* [Title/Abstract] OR zopiclon* [Title/Abstract]
#11	hypnotic*[Title/Abstract] OR drug*[Title/Abstract] OR “Drug Therapy”[Title/Abstract] OR pharmacotherapy[Title/Abstract] OR "Drug Therapy"[Mesh]
#12	OR #3 OR #4 OR #5 OR #6 OR #7 OR #8 OR #9 OR #10 OR #11
#13	#1 AND #2 AND #13

Database: *Cochrane Library* < April 9th 2019> 10785

Search Strategy:

#	**Searches**
#1	MeSH descriptor: [Drug Therapy] explode all trees
#2	(pharmacotherapy):ti,ab,kw
#3	(“Drug Therapy”):ti,ab,kw
#4	(drug*):ti,ab,kw
#5	(hypnotic*):ti,ab,kw
#6	MeSH descriptor: [Hypnotics and Sedatives] explode all trees
#7	#1 OR #2 OR #3 OR #4 OR #5 OR #6
#8	(zopiclon*):ti,ab,kw
#9	(eszopiclon*):ti,ab,kw
#10	(cyclopyrrolon*):ti,ab,kw
#11	(imidazopyridin*):ti,ab,kw
#12	(zolpidem):ti,ab,kw
#13	(zaleplon):ti,ab,kw
#14	(eszopiclone):ti,ab,kw
#15	(“Z drug*”):ti,ab,kw
#16	(non-benzodiazepin*):ti,ab,kw
#17	(“non benzodiazepin*”):ti,ab,kw
#18	(nonbenzodiazepin*):ti,ab,kw
#19	#8 OR #9 OR #10 OR #11 OR #12 OR #13 OR #14 OR #15 OR #16 OR #17 OR #18
#20	(triazolam OR temazepam OR rilmazafone OR oxazepam OR nitrazepam OR nimetazepam OR midazolam OR lormetazepam OR loprazolam OR haloxazolam OR flunitrazepam OR diazepam OR brotizolam OR benzodiaz* OR clonazepam OR lorazepam OR alprazolam OR flurazepam OR quazepam OR estazolam OR temazepam OR triazolam OR clonazepam):ti,ab,kw
#21	(“benzodiazepine tranquillizers”):ti,ab,kw
#22	(benzodiazepine):ti,ab,kw
#23	#20 OR #21 OR #22
#24	(melaton* OR Rozerem OR Ramelteon OR Melatoni):ti,ab,kw
#25	(quetiapine OR olanzapine OR antipsycho*):ti,ab,kw
#26	#24 OR #25
#27	(orexin OR suvorexant):ti,ab,kw
#28	(pregabalin OR gabapentin OR doxylamine OR diphenhydramine):ti,ab,kw
#29	#27 OR #28
#30	(antidepress* OR “anti depress*” OR doxepin OR mirtazapine OR trazodone OR amitriptyline):ti,ab,kw
#31	MeSH descriptor: [Antidepressive Agents] explode all trees
#32	# 30 OR #31
#33	#7 OR #19 OR #23 OR #26 OR #29 OR #32
#34	MeSH descriptor: [Sleep Initiation and Maintenance Disorders] explode all trees
#35	MeSH descriptor: [Sleep Wake Disorders] explode all trees
#36	MeSH descriptor: [Sleep Deprivation] this term only
#37	(insomnia*):ti,ab,kw
#38	#34 OR #35 OR #36 OR #37
#39	#33 AND #38

Database: *Embase*

Search Strategy:

#	Searches
#1	'insomnia'/exp
#2	'insomnia'/dd_dt
#3	(insomni* OR dyssomni* OR wake* OR awake*) :ti,ab,kw
#4	#1 OR #2 OR #3 162745
#5	(randomized OR randomised OR randomly OR random*):ti,ab,kw
#6	'randomized controlled trial':de 8
#7	randomization:de
#8	'controlled clinical trial':de
#9	#5 OR #6 OR #7 OR #8
#10	'hypnotic agent'/exp
#11	'antidepressant agent'/exp
#12	'drug therapy'/exp
#13	#10 OR #11 OR #12
#14	'diphenhydramine'/exp OR diphenhydramine OR 'doxylamine'/exp OR doxylamine OR 'gabapentin'/exp OR gabapentin OR 'pregabalin'/exp OR pregabalin
#15	'orexin'/exp OR orexin OR 'suvorexant'/exp OR suvorexant
#16	antipsycho* OR 'anti psycho*' OR 'olanzapine'/exp OR olanzapine OR 'quetiapine'/exp OR quetiapine
#17	'melatonin'/exp OR melatonin OR 'n acetyl 5 methoxytryptamine'/exp OR 'n acetyl 5 methoxytryptamine' OR 'ramelteon'/exp OR ramelteon OR 'rozerem'/exp OR rozerem OR melaton*
#18	'benzodiazepine'/exp OR benzodiazepine OR 'benzodiazepine tranquillizers' OR 'estazolam'/exp OR estazolam OR 'quazepam'/exp OR quazepam OR 'flurazepam'/exp OR flurazepam OR 'alprazolam'/exp OR alprazolam OR 'lorazepam'/exp OR lorazepam OR 'clonazepam'/exp OR clonazepam OR benzodiaz* OR 'brotizolam'/exp OR brotizolam OR 'diazepam'/exp OR diazepam OR 'flunitrazepam'/exp OR flunitrazepam OR 'haloxazolam'/exp OR haloxazolam OR 'loprazolam'/exp OR loprazolam OR 'lormetazepam'/exp OR lormetazepam OR 'midazolam'/exp OR midazolam OR 'nimetazepam'/exp OR nimetazepam OR 'nitrazepam'/exp OR nitrazepam OR 'oxazepam'/exp OR oxazepam OR 'rilmazafone'/exp OR rilmazafone OR 'temazepam'/exp OR temazepam OR 'triazolam'/exp OR triazolam
#19	nonbenzodiazepin* OR 'non benzodiazepin*' OR 'z drug*' OR 'eszopiclone'/exp OR eszopiclone OR 'zaleplon'/exp OR zaleplon OR 'zolpidem'/exp OR zolpidem OR imidazopyridin* OR cyclopyrrolon* OR eszopiclon* OR zopiclon*
#20	#14 OR #15 OR #16 OR #17 OR #18 OR #19
#21	#13 OR #20
#22	#4 AND #9 AND #21

Database: PsycInfo

#	Searches
#1	mainsubject(Dyssomnias) OR mainsubject(Wakefulness) OR mainsubject(Sleep Initiation AND Maintenance Disorders) OR mainsubject(Sleep Wake Disorders) OR mainsubject(Sleep Wake Disorders) OR ti(insomn*) OR ab(insomn*)
#2	mainsubject(Randomized Controlled Trial) OR mainsubject(Controlled Clinical Trial) OR ti(randomized) OR ab(randomized) OR ti(randomised) OR ab(randomised) OR ti(randomly) OR ab(randomly) OR ti(random*) OR ab(random*)
#3	mainsubject("Hypnotics and Sedatives") OR mainsubject("Benzodiazepines") OR mainsubject("Antidepressive Agents")
#4	ti(antidepress*) OR ab(antidepress*) OR ti("anti depress*) OR ab("anti depress*) OR ti(anti-depress*) OR ab(anti-depress*) OR ti(doxepin) OR ab(doxepin) OR ti(mirtazapine) OR ab(mirtazapine) OR ti(trazodone) OR ab(trazodone) OR ti(amitriptyline) OR ab(amitriptyline)
#5	ti(Diphenhydramine) OR ab(Diphenhydramine) OR ti(doxylamine) OR ab(doxylamine) OR ti(gabapentin) OR ab(gabapentin) OR ti(pregabalin) OR ab(pregabalin)
#6	ti(orexin) OR ab(orexin) OR ti(suvorexant) OR ab(suvorexant)
#7	ti(antipsycho*) OR ab(antipsycho*) OR ti(olanzapine) OR ab(olanzapine) OR ti(quetiapine) OR ab(quetiapine) OR ti(olanzapine) OR ab(olanzapine)
#8	ti(Melatonin) OR ab(Melatonin) OR ti(N-acetyl-5-methoxytryptamine) OR ab(N-acetyl-5-methoxytryptamine) OR ti(Ramelteon) OR ab(Ramelteon) OR ti(Rozerem) OR ab(Rozerem) OR ti(melaton*) OR ab(melaton*)
#9	ti(Benzodiazepine) OR ab(Benzodiazepine) OR ti("benzodiazepine tranquillizers") OR ab("benzodiazepine tranquillizers") OR ti(clonazepam) OR ab(clonazepam) OR ti(triazolam) OR ab(triazolam) OR ti(temazepam) OR ab(temazepam) OR ti(estazolam) OR ab(estazolam) OR ti(quazepam) OR ab(quazepam) OR ti(flurazepam) OR ab(flurazepam) OR ti(alprazolam) OR ab(alprazolam) OR ti(lorazepam) OR ab(lorazepam) OR ti(clonazepam) OR ab(clonazepam) OR ti(benzodiaz*) OR ab(benzodiaz*) OR ti(brotizolam) OR ab(brotizolam) OR ti(diazepam) OR ab(diazepam) OR ti(flunitrazepam) OR ab(flunitrazepam) OR ti(haloxazolam) OR ab(haloxazolam) OR ti(loprazolam) OR ab(loprazolam) OR ti(lormetazepam) OR ab(lormetazepam) OR ti(midazolam) OR ab(midazolam) OR ti(nimetazepam) OR ab(nimetazepam) OR ti(nitrazepam) OR ab(nitrazepam) OR ti(oxazepam) OR ab(oxazepam) OR ti(rilmazafone) OR ab(rilmazafone) OR ti(temazepam) OR ab(temazepam) OR ti(triazolam) OR ab(triazolam)
#10	ti(nonbenzodiazepin*) OR ab(nonbenzodiazepin*) OR ti(("non benzodiazepine")) OR ab(("non benzodiazepine")) OR ti(non-benzodiazepin*) OR ab(non-benzodiazepin*) OR ti("Z drug*") OR ab("Z drug*") OR ti(eszopiclone) OR ab(eszopiclone) OR ti(zaleplon) OR ab(zaleplon) OR ti(zolpidem) OR ab(zolpidem) OR ti(imidazopyridin*) OR ab(imidazopyridin*) OR ti(cyclopyrrolon*) OR ab(cyclopyrrolon*) OR ti(eszopiclon*) OR ab(eszopiclon*) OR ti(zopiclon*) OR ab(zopiclon*)
#11	ti(hypnotic*) OR ab(hypnotic*) OR ti(drug*) OR ab(drug*) OR ti("Drug Therapy") OR ab("Drug Therapy") OR ti(pharmacotherapy) OR ab(pharmacotherapy) OR mainsubject("Drug Therapy")
#12	#4 OR #6 OR #7 OR #8 OR #10 OR #11 OR #12 OR #13 OR #14
#13	#1 AND #2 AND #12

Appendix 3 Study eligibility form

**Population:**		
• Adults aged 18 or older with primary insomnia	**YES**	**NO**

**Intervention:**		
• pharmacological interventions	**YES**	**NO**

**Control:**		
• Placebo and/or another agent	**YES**	**NO**

**Type of article:**		
• Randomized controlled trails (RCTs)	**YES**	**NO**

**Type of outcomes:**		
• Specific adverse events	**YES**	**NO**

**Study inclusion:**	
• All the answers are YES	**INCLUDE**
• Any answer is NO	**EXCLUDE**
• If you are unsure of the answer, include for full text screening	**INCLUDE**

Instructions:

1. For the type of articles, cluster-randomized trials and cross-over trials should be excluded, in order to avoid possible sources of heterogeneity.
2. We only include studies on primary insomnia and exclude those considering patients with insomnia due to psychiatric or physical comorbidity.
3. On occasion, some of the above criteria will be unclear. If any response to the above questions is UNCLEAR, mark YES.
4. Considered NO, if treatments are as follows: (1) combination of non-pharmacological treatments (e.g. drug+ non-pharmacological treatments vs drug/ non-pharmacological treatments); (2) all non-pharmacological treatments

Appendix 4 Bassline characteristics of included trails

Author, year	Country	Sample Size	Duration of Treatment	Women %	Age mean±SD	Patient type	Insomnia diagnosis	Funding
Black,2017	USA	709	16 days	61.64	45.4	NR	DSM-IV-TR	Pharmaceutical Inc
Pinto, 2016	Brazil	262	4 weeks	56.87	47.37±11.36	NR	DSM-IV	Pharmaceutical Inc
Michelson, 2014	Americas, Australia, Europe, and South Africa	781	12 weeks	55.83	61.53±14.53	NR	DSM-IV-TR	Pharmaceutical Inc
Roth, 2006a	USA	212	3 weeks	58.02	44.3±3.0	NR	DSM-IV	NR
Roth, 2006b	USA	207	12 days	54.59	71.27±4.88	NR	DSM-IV-TR	Pharmaceutical Inc
Roth, 2006c	USA	829	35 night	58.87	72.4±5.95	outpatient	DSM-IV-TR	Pharmaceutical Inc
Roth, 2007	USA	264	4 weeks	70.45	45.77±11	outpatient	DSM-IV	Pharmaceutical Inc
Roth, 2010a	USA	605	48 weeks	58.35	45.15±11.74	outpatient	DSM-IV	Pharmaceutical Inc
Roth, 2010b	USA	928	12 weeks	64.44	43.07±11.75	outpatient	DSM-IV	Pharmaceutical Inc
Roth, 2013	USA	300	28 nights	67.00	42.84±11.35	outpatient	DSM-IV-TR	Pharmaceutical Inc
Huang, 2011	China	48	28 days	68.75	39.9±16.95	NR	DSM-IV	Pharmaceutical Inc
Uchiyama, 2010b	Japan	987	2 weeks	62.92	38.8 ± 13.76	outpatient	DSM-IV	Pharmaceutical Inc
Lankford, 2012	USA	255	4 weeks	40.00	72.45±5.94	outpatient	DSM-IV-TR	Pharmaceutical Inc
Krystal, 2003	USA	788	24 weeks	63.20	43.47±11.18	outpatient	DSM-IV	Pharmaceutical Inc
Krystal, 2008	USA	1025	24 weeks	60.78	45.7±11.0	outpatient	DSM-IV-TR	Pharmaceutical Inc
Krystal, 2010	USA	240	12 weeks	65.00	71.4 ±5.2	outpatient	DSM-IV-TR	Pharmaceutical Inc
Krystal, 2011	USA	229	5 weeks	70.31	44.5±11.3	NR	DSM-IV-TR	Pharmaceutical Inc
Wade, 2007	UK	354	2 weeks	56.78	65.7±6.4	Primary care	sleep history	Pharmaceutical Inc

							questionnaire (SHQ)	
Walsh, 1998a	USA	132	2 weeks	58.33	40.25±10.26	NR	DSM-IIIR	Pharmaceutical Inc
Walsh, 1998b	USA	306	2 weeks	NR	21-65	NR	DSMIII-R	Pharmaceutical Inc
Walsh, 2006	USA	232	NR	68.97	44.35±12.29	NR	DSM-IV-TR	Pharmaceutical Inc
Walsh, 2007a	USA	358	12 weeks	54.75	70.88±0.4	NR	DSM-IV	Pharmaceutical Inc
Walsh, 2007b	USA	830	24 weeks	60.84	45.56±11.8	NR	DSM-IV	Pharmaceutical Inc
Walsh, 2008	USA	205	3 weeks	57.07	70.2±4.5	NR	DSM-IV	Pharmaceutical Inc
Walsh, 2010	USA	149	1 week	64.43	71.3±4.9	NR	DSM-IV	Pharmaceutical Inc
Mayer, 2009	Germany	451	24 weeks	NR	46.2±14.80	NR	NR	Pharmaceutical Inc
Zammit, 2004	USA	308	46 nights	64.61	39.8±11.7	NR	DSM-IV	Pharmaceutical Inc
Zammit, 2007	USA	405	5 weeks	67.16	39.28±11.98	NR	DSM-IV-TR	Pharmaceutical Inc
Scharf, 1990	USA	244	1 week	NR	21-65	outpatient	NR	NR
Scharf, 1994	USA	75	5 weeks	36.00	38	outpatient	NR	NR
Scharf, 2006	USA	231	2 weeks	57.58	72.3±3.33	outpatient	DSM-IV	Pharmaceutical Inc
Scharf, 2007	USA	702	12 weeks	39.03	45.6±11.25	outpatient	DSM-IV	Pharmaceutical Inc
McCall, 2006	USA	270	2 weeks	65.93	70.11±5.06	outpatient	DSM-IV	Pharmaceutical Inc
Fry, 2000	USA	595	9 weeks	57.48	41.8±11.64	outpatient	DSM-III-R	Pharmaceutical Inc
Elie, 1999	Canada	615	28 nights	60.16	42.84±12.42	outpatient	DSM-III-R	Pharmaceutical Inc
Lahmeyer, 1997	USA	145	6 weeks	55.86	44.9±11.6	outpatient	NR	Pharmaceutical Inc
Ancoli-Israel, 2010	USA	388	12 weeks	62.63	72±5.11	outpatient	DSM-IV	Pharmaceutical Inc
Riemann, 2002	Germany	55	7 weeks	41.82	47.03±11.02	outpatient	DSM-III-R	Pharmaceutical Inc
Luthringer, 2010	France	40	3 weeks	40.00	60.75±4.08	outpatient	DSM-IV	Pharmaceutical Inc
Zhou, 2002	China	40	12 weeks	37.50	68±12.49	outpatient	CCMD-2-R	NR

Hajak, 2001	Germany	47	6 weeks	76.60	47.4 ± 16.8	NR	DSM-IV	Pharmaceutical Inc
Hajak, 2009	Germany	742	4 weeks	63.48	48.14±11.29	outpatient	DSM-IV	Pharmaceutical Inc
Lydiard, 2006	USA	229	4 weeks	59.83	71.29±4.70	NR	DSM-IV	Pharmaceutical Inc
Fan, 2017	China	120	24 weeks	40.00	51±12	outpatient	DSM-IV-TR	NR
Allain, 2001	France	245	5 weeks	76.73	46.13±10.57	NR	DSM-IV	Pharmaceutical Inc
Allain, 1998	France	84	28 days	67.86	54.3±11.0	outpatient	NR	NR
Herrmann, 1993	Germany	21	28 days	42.86	25-65	outpatient	DSM-III-R	NR
Sivertsen, 2006	Norway	48	6 weeks	45.83	60.8±5.4	outpatient	DSM-IV	government
Morin, 2003	USA	60	8 weeks	56.67	64.75±6.95	outpatient	DSM-III-R	government
Herring, 2016a	NR	1022	12 weeks	62.33	55.75±15.3	NR	DSM-IV-TR	Pharmaceutical Inc
Herring, 2016b	NR	1021	12 weeks	65.72	56.75±15.3	NR	DSM-IV-TR	Pharmaceutical Inc
Leppik, 1997	USA	335	4 weeks	62.99	69 (range 59–85)	NR	DSM-III-R	Pharmaceutical Inc
Dockhorn, 1996	USA	139	1 week	56.83	32.7(range 20-55)	NR	DSM-III-R	Pharmaceutical Inc
Tamminen, 1987	Finland	130	6 weeks	55.38	47	outpatient	NR	NR
Jovanovic, 1983	France	10	2 weeks	50.00	30.1	NR	NR	NR
Ivgy-May, 2015a	USA and Canada	419	6 weeks	63.25	44.96±11.1	outpatient	DSM-IV	Pharmaceutical Inc
Ivgy-May, 2015b	USA and Canada	526	2 weeks	64.45	45.28±11.98	outpatient	DSM-IV	Pharmaceutical Inc
Ivgy-May, 2020	Asia, Canada, Europe, Latin	460	24 weeks	61.09	47.8±11.3	outpatient	DSM-IV-TR	Pharmaceutical Inc

	America, and the United States							
NCT00548340	USA	322	5 weeks	61.18	41.8±10.9	NR	DSM-IV	Pharmaceutical Inc
NCT01463098	USA	58	NR	46.55	NR	NR	DSM-IV-TR	NR
Kärppä, 2020	Finland. A total of 119 sites in North America (45), Europe (34), Asia (35), and Oceania (5)	971	48 weeks	66.63	54.5±13.8	NR	DSM-IV	Pharmaceutical Inc
Dauvilliers, 2020	Germany, Hungary,Israel, Spain, Sweden, USA	1005	4 weeks	64.07	44.7±11.3	outpatient	DSM-IV	Pharmaceutical Inc
Soares, 2006	NR	410	4 weeks	0.00	49.1	NR	DSM-IV	NR
Murphy,2017	USA	291	15 days	62.54	48.3	NR	DSM-IV	Pharmaceutical Inc
Ratti, 2013	Germany	161	6 weeks	54.66	45±7.5	outpatient	DSM-IV	Pharmaceutical Inc
Lemoine, 2007	France	170	3 weeks	65.88	68.5±8.31	NR	DSM-IV	Pharmaceutical Inc
Fleming, 1995	USA	144	4 nights	48	33-37	NR	NR	NR
Tsutsui, 2001	Japan	479	3 weeks	58.04	42.17±12.68	inpatient	NR	NR
Zhang, 2004	China	47	2 weeks	57.45	46.49±15.6	outpatient	CCMD-2-R	NR
Uchimura, 2011	Japan	1145	1 week	62.36	48.8±17.2	outpatient	DSM-IV-TR	Pharmaceutical Inc
Dehlin, 1995	Sweden	107	4 weeks	71.11	79(range 60–95)	NR	NR	Pharmaceutical Inc
Klimm, 1987	France	74	1 week	79.73	73.2±1.54	outpatient	NR	NR
Takeda, 2007	USA	135	10 weeks	32.59	49.2±13.35	outpatient	NR	government

Katz, 2011	USA	137	4 weeks	29.20	49.6±14.8	NR	NR	NR
Cordingley, 1984	UK	269	2 weeks	71.47	range 35-65	NR	NR	NR
Dahl, 1982	Sweden	58	1 weeks	65	54.35	outpatient	NA	NR
Elie, 1990	Canada	36	4 weeks	66.67	37.6±11.04	outpatient	NA	NR
Fabre, 1977	USA	110	1 weeks	48.03	41.99	outpatient	NA	NR
Allen, 1987	USA	141	4 weeks	50	37.3(range 18-65)	NR	NA	NR
Anderson, 1987	UK	94	2 weeks	NR	Range 20-69	NR	NA	NR
Bayer, 1986	UK	89	5 days	67.42	77.84(range 65-over 65)	inpatient	NA	NR
Dominguez, 1985	USA	59	3 weeks	over 60	49(range 20-60)	outpatient	NA	NR
Goldenberg, 1994	Belgium, Finland, France, The Netherlands and Eire	458	2 weeks	NR	range 25-60	NR	NA	NR
Mignot, 2022a	Australia, Canada, Denmark, Germany, Italy, Poland, Serbia, Spain, Switzerland, and the USA	930	3 months	67	55.47±15.34	NR	DSM-5	Pharmaceutical Inc
Mignot, 2022b	Belgium, Bulgaria, Canada, Czech Republic, Finland, France, Germany, Hungary, South Korea, Sweden, and the USA	924	3 months	69	56.7±14.18	NR	DSM-5	Pharmaceutical Inc
Monti, 1994	Uruguay	24	27 nights	87.5	47.33±14.03	NR	NA	NR
Richardson, 2009	USA	66	6 months	56.56	34.3	NR	DSM-IV-TR	Pharmaceutical Inc

Wang-Weigand, 2011	USA	441	3 weeks	64.7	43.2±12.5	outpatient	NA	Pharmaceutical Inc
Heidrich, 1981	Geramany	60	2 weeks	67	45.3(range 21-64)	outpatient	NA	NR
Begg, 1992	New Zealand	51	1 weeks	NR	range 18-over 18	NR	NA	Pharmaceutical Inc
Moon, 1985	UK	1791	1 weeks	65.61	49.66(range 18-94)	NR	NA	NR
Roger, 1993	France and Belgium	205	3 weeks	74.21	81.08±7.5	inpatient	NA	NR
NCT00156533	USA	20	12 weeks	70	range 25-55	NR	ICSD	University, Pharmaceutical Inc
NCT00177216	USA	69	100 days	55.1	36.67±9.05	NR	DSM-IV	University
NCT00383643	USA	48	12 weeks	66.7	53.2±12.3	NR	ICSD	University
NCT00755495	NR	472	5 weeks	NR	range 18-80	NR	DSM-IV-TR	Pharmaceutical Inc
NCT00756002	Europe and Russia	259	5 weeks	41.7	42±12.05	NR	NA	Pharmaceutical Inc
NCT03375203	Belgium, France, Germany, Japan, Poland, United States	364	2 weeks	67.6	57.8±12.4	NR	DSM-5	Pharmaceutical Inc
NCT03545191	Australia, Canada, Denmark, Germany, Italy, Poland, Serbia, Spain, Switzerland, United States	930	3 months	67.1	55.4±15.3	NR	DSM-5	Pharmaceutical Inc
NCT03575104	Belgium, Bulgaria, Canada, Czechia, Finland, France,	924	3 months	69	56.7±14.2	NR	DSM-5	Pharmaceutical Inc

	Germany, Hungary, Korea, Republic of, Sweden, United States							
Rosenberg, 1994	Denmark	178	2 weeks	53.93	51.07±12.63	NR	NR	Pharmaceutical Inc
Rosenberg, 2019	North America and Europe	1006	4 weeks	86.38	median63 (55-88)	NR	DSM-V	Pharmaceutical Inc

Appendix 5 Detail drug information

Author, year	Treatment Group			Control Group					
	Drug	Drug classess	Dose	Drug	Drug classess	Dose	Drug	Drug classess	Dose
Black,2017	Almorexant	Orexin receptor antagonists	100, 200mg/d	Placebo	NA	NA	NA	NA	NA
Pinto, 2016	Eszopiclone	Non-benzodiazepines	3mg/d	Zopiclone	Non-benzodiazepines	7.5mg/d	NA	NA	NA
Michelson, 2014	Suvorexant	Orexin receptor antagonists	30mg/d for elderly patients<br>40mg/d for non-elderly patients	Placebo	NA	NA	NA	NA	NA
Roth, 2006a	Zolpidem	Non-benzodiazepines	12.5mg/d	Placebo	NA	NA	NA	NA	NA
Roth, 2006b	Tiagabine	Antiepileptic	2, 4, 6, 8mg/d	Placebo	NA	NA	NA	NA	NA
Roth, 2006c	Ramelteon	Melatonin receptor agonists	4, 8mg/d	Placebo	NA	NA	NA	NA	NA
Roth, 2007	Indiplon	Non-benzodiazepines	10,20mg/d	Placebo	NA	NA	NA	NA	NA
Roth, 2010a	Indiplon	Non-benzodiazepines	10,20mg/d	Placebo	NA	NA	NA	NA	NA
Roth, 2010b	Zolpidem	Non-benzodiazepines	10mg/d	Placebo	NA	NA	NA	NA	NA
Roth, 2013	Zolpidem	Non-benzodiazepines	3.5mg/d	Placebo	NA	NA	NA	NA	NA
Huang, 2011	Zaleplon	Non-benzodiazepines	10mg/d	Zolpidem	Non-benzodiazepines	10mg/d	NA	NA	NA
Uchiyama, 2010b	Ramelteon	Melatonin receptor agonists	8mg/d	Placebo	NA	NA	NA	NA	NA
Lankford, 2012	Doxepin	Antidepressant	6mg/d	Placebo	NA	NA	NA	NA	NA
Krystal, 2003	Eszopiclone	Non-benzodiazepines	3mg/d	Placebo	NA	NA	NA	NA	NA
Krystal, 2008	Zolpidem	Non-benzodiazepines	12.5mg/d	Placebo	NA	NA	NA	NA	NA
Krystal, 2010	Doxepin	Antidepressant	1, 3mg/d	Placebo	NA	NA	NA	NA	NA
Krystal, 2011	Doxepin	Antidepressant	3, 6mg/d	Placebo	NA	NA	NA	NA	NA
Wade, 2007	Melatonin	Melatonin receptor agonists	2 mg/d	Placebo	NA	NA	NA	NA	NA

Walsh, 1998a	Zaleplon	Non-benzodiazepines	5, 10mg/d	Triazolam	Benzodiazepines	0.25 mg/d	NA	NA	NA
Walsh, 1998b	Trazodone	Antidepressant	50mg/d	Zolpidem	Non-benzodiazepines	10mg/d	Placebo	NA	NA
Walsh, 2006	Tiagabine	Antiepileptic	4, 6, 8, 10mg/d	Placebo	NA	NA	NA	NA	NA
Walsh, 2007a	Indiplon	Non-benzodiazepines	5, 10mg/d	Placebo	NA	NA	NA	NA	NA
Walsh, 2007b	Eszopiclone	Non-benzodiazepines	3mg/d	Placebo	NA	NA	NA	NA	NA
Walsh, 2008	Zolpidem	Non-benzodiazepines	6.25mg/d	Placebo	NA	NA	NA	NA	NA
Walsh, 2010	EVT 201	Benzodiazepines	1.5, 2.5mg/d	Placebo	NA	NA	NA	NA	NA
Mayer, 2009	Ramelteon	Melatonin receptor agonists	8mg/d	Placebo	NA	NA	NA	NA	NA
Zammit, 2004	Eszopiclone	Non-benzodiazepines	2, 3mg/d	Placebo	NA	NA	NA	NA	NA
Zammit, 2007	Ramelteon	Melatonin receptor agonists	8, 16mg/d	Placebo	NA	NA	NA	NA	NA
Scharf, 1990	Estazolam	Benzodiazepines	2 mg/d	Flurazepam	Benzodiazepines	30mg/d	NA	NA	NA
Scharf, 1994	Zolpidem	Non-benzodiazepines	10, 15mg/d	Placebo	NA	NA	NA	NA	NA
Scharf, 2006	Eszopiclone	Non-benzodiazepines	1, 2mg/d	Placebo	NA	NA	NA	NA	NA
Scharf, 2007	Indiplon	Non-benzodiazepines	10, 20mg/d	Placebo	NA	NA	NA	NA	NA
McCall, 2006	Eszopiclone	Non-benzodiazepines	2 mg/d	Placebo	NA	NA	NA	NA	NA
Fry, 2000	Zaleplon	Non-benzodiazepines	5, 10, 20mg/d	Zolpidem	Non-benzodiazepines	10mg/d	NA	NA	NA
Elie, 1999	Zaleplon	Non-benzodiazepines	5, 10, 20mg/d	Zolpidem	Non-benzodiazepines	10mg/d	Placebo	NA	NA
Lahmeyer, 1997	Zolpidem	Non-benzodiazepines	10, 15mg/d	Placebo	NA	NA	NA	NA	NA
Ancoli-Israel, 2010	Eszopiclone	Non-benzodiazepines	2mg/d	Placebo	NA	NA	NA	NA	NA
Riemann, 2002	Lormetazepam	Benzodiazepines	1mg/d	Trimipramine	Antidepressant	25-200mg/d	NA	NA	NA

Luthringer, 2010	Melatonin	Melatonin receptor agonists	2mg/d	Placebo	NA	NA	NA	NA	NA
Zhou, 2002	Paroxetine	Antidepressant	10-20 mg/d	Alprazolam	Benzodiazepines	0.4-0.8 mg/d	NA	NA	NA
Hajak, 2001	Doxepin	Antidepressant	25-50 mg/d	Placebo	NA	NA	NA	NA	NA
Hajak, 2009	Gaboxadol	Non-benzodiazepines	5, 10, 15mg/d	Zolpidem	Non-benzodiazepines	10mg/d	NA	NA	NA
Lydiard, 2006	Indiplon	Non-benzodiazepines	15 mg/d	Placebo	NA	NA	NA	NA	NA
Fan, 2017	Suvorexant	Orexin receptor antagonists	40mg/d	Placebo	NA	NA	NA	NA	NA
Allain, 2001	Zolpidem	Non-benzodiazepines	10mg/d	Placebo	NA	NA	NA	NA	NA
Allain, 1998	Triazolam	Benzodiazepines	0.125 mg/d	Zolpidem	Non-benzodiazepines	10 mg/d	NA	NA	NA
Herrmann, 1993	Zolpidem	Non-benzodiazepines	10mg/d	Placebo	NA	NA	NA	NA	NA
Sivertsen, 2006	Zopiclone	Non-benzodiazepines	7.5mg/d	Placebo	NA	NA	NA	NA	NA
Morin, 2003	Temazepam	Benzodiazepines	7.5-30mg/d	Placebo	NA	NA	NA	NA	NA
Herring, 2016a	Suvorexant	Orexin receptor antagonists	20/15, 40/30 mg/d	Placebo	NA	NA	NA	NA	NA
Herring, 2016b	Suvorexant	Orexin receptor antagonists	20/15, 40/30 mg/d	Placebo	NA	NA	NA	NA	NA
Leppik, 1997	Zolpidem	Non-benzodiazepines	5 mg/d	Triazolam	Benzodiazepines	0.125mg	NA	NA	NA
Dockhorn, 1996	Zolpidem	Non-benzodiazepines	10mg/d	Placebo	NA	NA	Temazepam	Benzodiazepines	15mg
Tamminen, 1987	Zopiclone	Non-benzodiazepines	7.5mg/d	Nitrazepam	Benzodiazepines	5mg/d	NA	NA	NA
Jovanovic, 1983	Zopiclone	Non-benzodiazepines	7.5mg/d	Nitrazepam	Benzodiazepines	5 mg/d	NA	NA	NA
Ivgy-May, 2015a	Esmirtazapine	Antidepressant	3, 4.5 mg/d	Placebo	NA	NA	NA	NA	NA
Ivgy-May, 2015b	Esmirtazapine	Antidepressant	1.5, 3, 4.5 mg/d	Placebo	NA	NA	NA	NA	NA

Ivgy-May, 2020	Esmirtazapine	Antidepressant	1.5-4.5mg/d	Placebo	NA	NA	NA	NA	NA
NCT00548340	Tasimelteon	Melatonin receptor agonists	20, 50mg/d	Placebo	NA	NA	NA	NA	NA
NCT01463098	Lemborexant	Orexin receptor antagonists	2.5, 10, 25 mg/d	Zolpidem	Non-benzodiazepines	10mg/d	NA	NA	NA
Kärppä, 2020	Lemborexant	Orexin receptor antagonists	5, 10mg/d	Placebo	NA	NA	NA	NA	NA
Dauvilliers, 2020	Daridorexant	Orexin receptor antagonists	5, 10, 25, 50 mg/d	Zolpidem	Non-benzodiazepines	10mg/d	NA	NA	NA
Soares, 2006	Eszopiclone	Non-benzodiazepines	3 mg/d	Placebo	NA	NA	NA	NA	NA
Murphy,2017	Lemborexant	Orexin receptor antagonists	1, 2.5, 5, 10, 25 mg/d	Placebo	NA	NA	NA	NA	NA
Ratti, 2013	Vestipitant	Antidepressant	15 mg/d	Placebo	NA	NA	NA	NA	NA
Lemoine, 2007	Melatonin	Melatonin receptor agonists	2 mg/d	Placebo	NA	NA	NA	NA	NA
Fleming, 1995	Zolpidem	Non-benzodiazepines	10, 20mg/d	Flurazepam	Benzodiazepines	30mg/d	NA	NA	NA
Tsutsui, 2001	Zolpidem	Non-benzodiazepines	10mg/d	Placebo	NA	NA	NA	NA	NA
Zhang, 2004	Zolpidem	Non-benzodiazepines	10mg/d	Zaleplon	Non-benzodiazepines	10mg/d	NA	NA	NA
Uchimura, 2011	Ramelteon	Melatonin receptor agonists	4, 8 mg/d	Placebo	NA	NA	NA	NA	NA
Dehlin, 1995	Zopiclone	Non-benzodiazepines	5 mg/d	Flurazepam	Benzodiazepines	1 mg/d	Zopiclone	Non-benzodiazepines	5 mg/d
Klimm, 1987	Zopiclone	Non-benzodiazepines	7.5 mg/d	Nitrazepam	Benzodiazepines	5 mg/d	NA	NA	NA
Takeda, 2007	Ramelteon	Melatonin receptor agonists	8 mg/d	Placebo	NA	NA	NA	NA	NA
Katz, 2011	Melatonin	Melatonin receptor agonists		Placebo	NA	NA	NA	NA	NA

Cordingley, 1984	Flunitrazepam	Benzodiazepines	1mg/d	Triazolam	Benzodiazepines	0.25mg/d	NA	NA	NA
Dahl, 1982	Triazolam	Benzodiazepines	0.5mg/d	Nitrazepam	Benzodiazepines	5mg/d	NA	NA	NA
Elie,1990	Zopiclone	Non-benzodiazepines	7.5mg/d	Flurazepam	Benzodiazepines	30mg/d	NA	NA	NA
Fabre, 1977	Triazolam	Benzodiazepines	0.5mg/d	Flurazepom	Benzodiazepines	30mg/d	NA	NA	NA
Allen, 1987	Midalozam	Midazolam	15mg/d	Temazepam	Benzodiazepine	30mg/d	NA	NA	NA
Anderson, 1987	Zopiclone	Non-benzodiazepines	7.5mg/d	Nitrazepam	Placebo	5mg/d	Placebo	NA	NA
Bayer, 1986	Loprazolam	Benzodiazepine	0.5, 1mg/d	Placebo	NA	NA	NA	NA	NA
Dominguez, 1985	Brotizolam	Benzodiazepines	0.25mg/d	Placebo	NA	NA	NA	NA	NA
Goldenberg, 1994	Zopiclone	Non-benzodiazepines	7.5mg/d	Placebo	NA	NA	NA	NA	NA
Mignot, 2022a	Daridorexant	Orexin receptor antagonists	50, 25mg/d	Placebo	NA	NA	NA	NA	NA
Mignot, 2022b	Daridorexant	Orexin receptor antagonists	25, 10mg/d	Placebo	NA	NA	NA	NA	NA
Monti, 1994	Zolpidem	BzRAs	10mg/d	Triazolam	Benzodiazepines	0.5mg/d	Placebo	NA	NA
Wang-Weigand, 2011	Ramelteon	Melatonin receptor agonists	8mg/d	Placebo	NA	NA	NA	NA	NA
Heidrich, 1981	Lormetazepam	Benzodiazepine	2mg/d	Placebo	NA	NA	NA	NA	NA
Begg, 1992	Midazolam	Benzodiazepine	15mg/d	Zopiclone	Non-benzodiazepines	7.5mg/d	NA	NA	NA
Moon, 1985	Loprazolam	Loprazolam	1mg/d	Triazolam	Benzodiazepines	0.25mg/d	NA	NA	NA
Roger, 1993	Zolpidem	BzRAs	5mg/d	Zolpidem	BzRAs	10mg/d	Triazolam	Benzodiazepines	0.25mg/d

NCT00156533	Zolpidem	BzRAs	10mg/d	Zolpidem	BzRAs	3-5 pills/week	Placebo	NA	NA
NCT00177216	Zolpidem	BzRAs	5mg/d	Placebo	Placebo	NA	NA	NA	NA
NCT00383643	Zolpidem	BzRAs	NA	Placebo	Placebo	NA	NA	NA	NA
NCT00755495	Ramelteon	Melatonin receptor agonists	8mg/d	Doxepin	Antidepressant	3mg/d	NA	NA	NA
NCT00756002	Ramelteon	Melatonin receptor agonists	4mg/d	Placebo	NA	NA	NA	NA	NA
NCT03375203	Zolpidem	BzRAs	5mg/d	Placebo	Placebo	NA	NA	NA	NA
NCT03545191	Daridorexant	Orexin receptor antagonists	25, 50mg/d	Placebo	NA	NA	NA	NA	NA
NCT03575104	Daridorexant	Orexin receptor antagonists	10mg, 25mg/d	Placebo	NA	NA	NA	NA	NA
Richardson 2009	Ramelteon	Melatonin receptor agonists	16mg/d	Placebo	NA	NA	NA	NA	NA
Rosenberg, 1994	Zolpidem	BzRAs	10mg/d	Triazolam	Benzodiazepines	0.25 mg/d	NA	NA	NA
Rosenberg, 2019	Lemborexant	Orexin receptor antagonists	5, 10 mg/d	Zolpidem	BzRAs	6.25 mg/d	NA	NA	NA

Appendix 6 The incidence of different adverse events

The incidence of gastrointestinal disorders

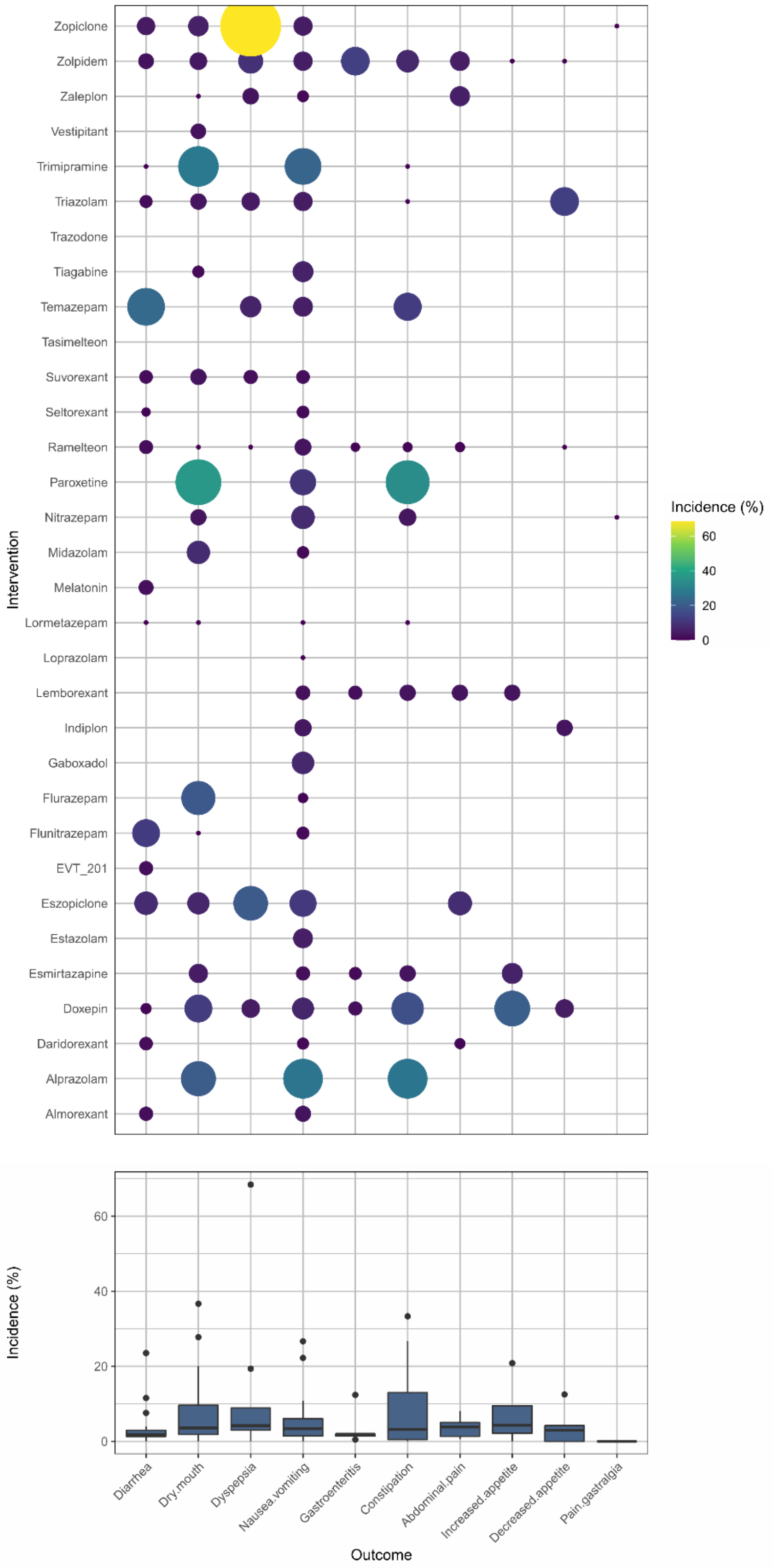

The incidence of general disorders and administration site conditions

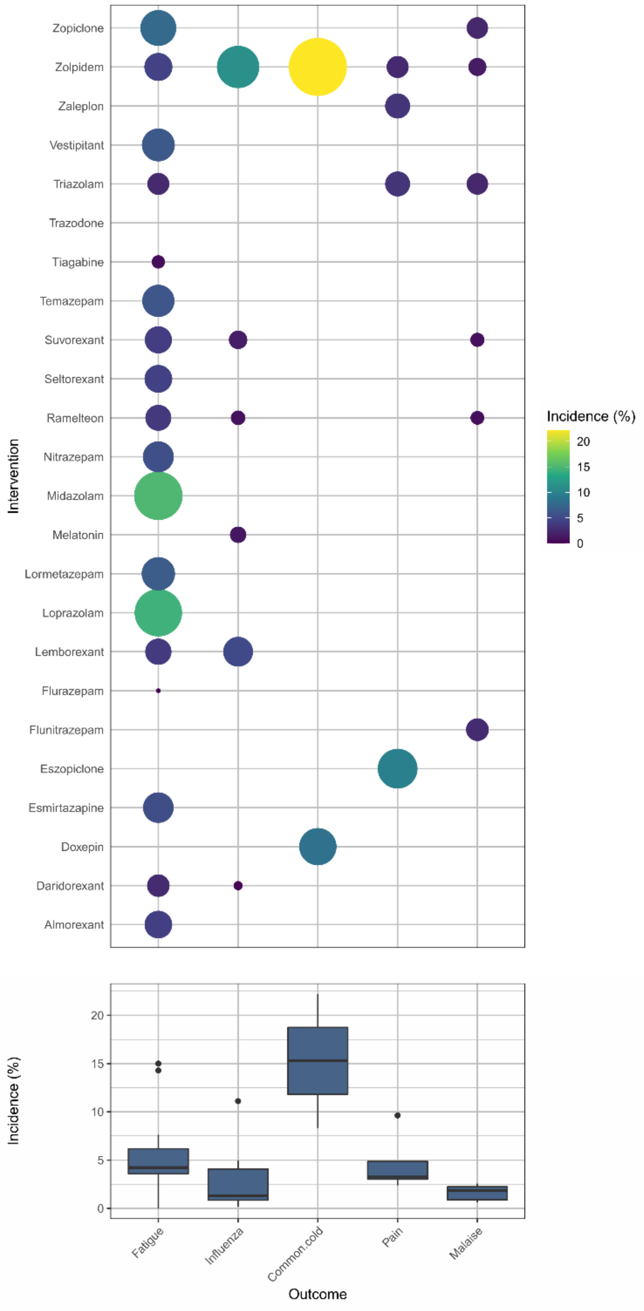

The incidence of respiratory, thoracic and mediastinal disorders

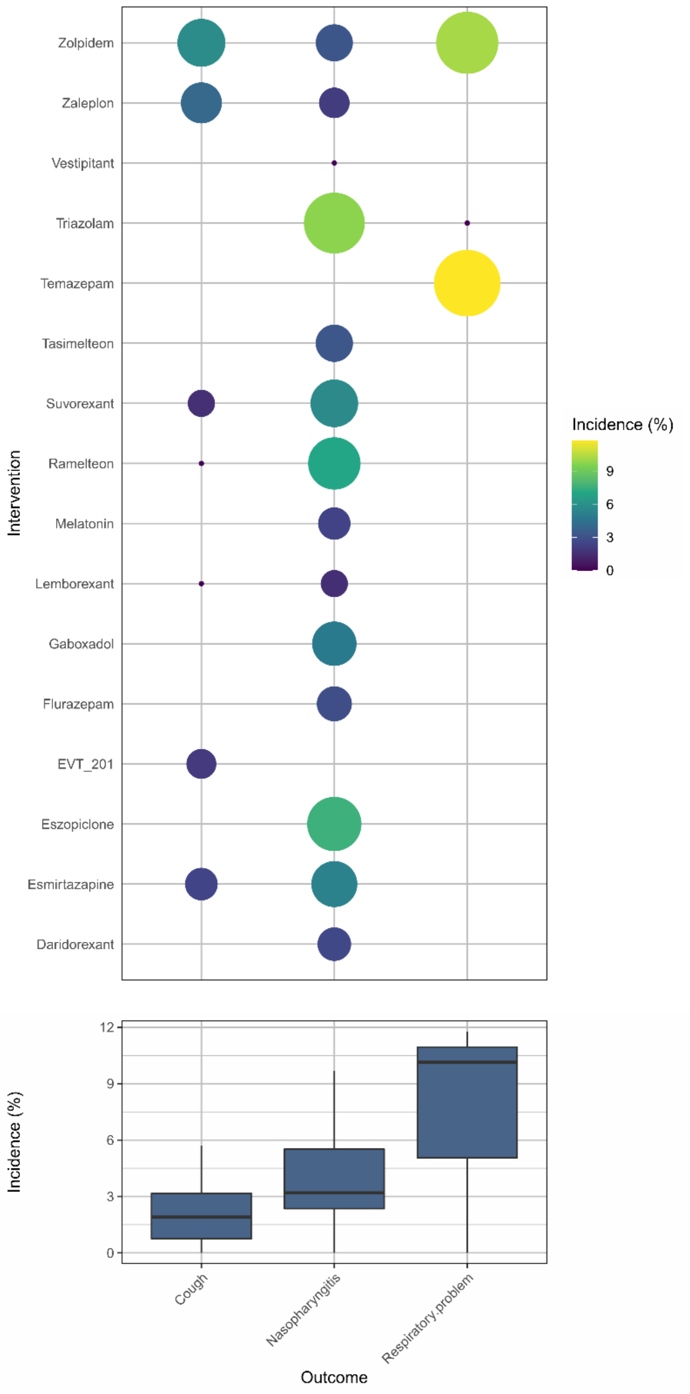

The incidence of psychiatric disorders

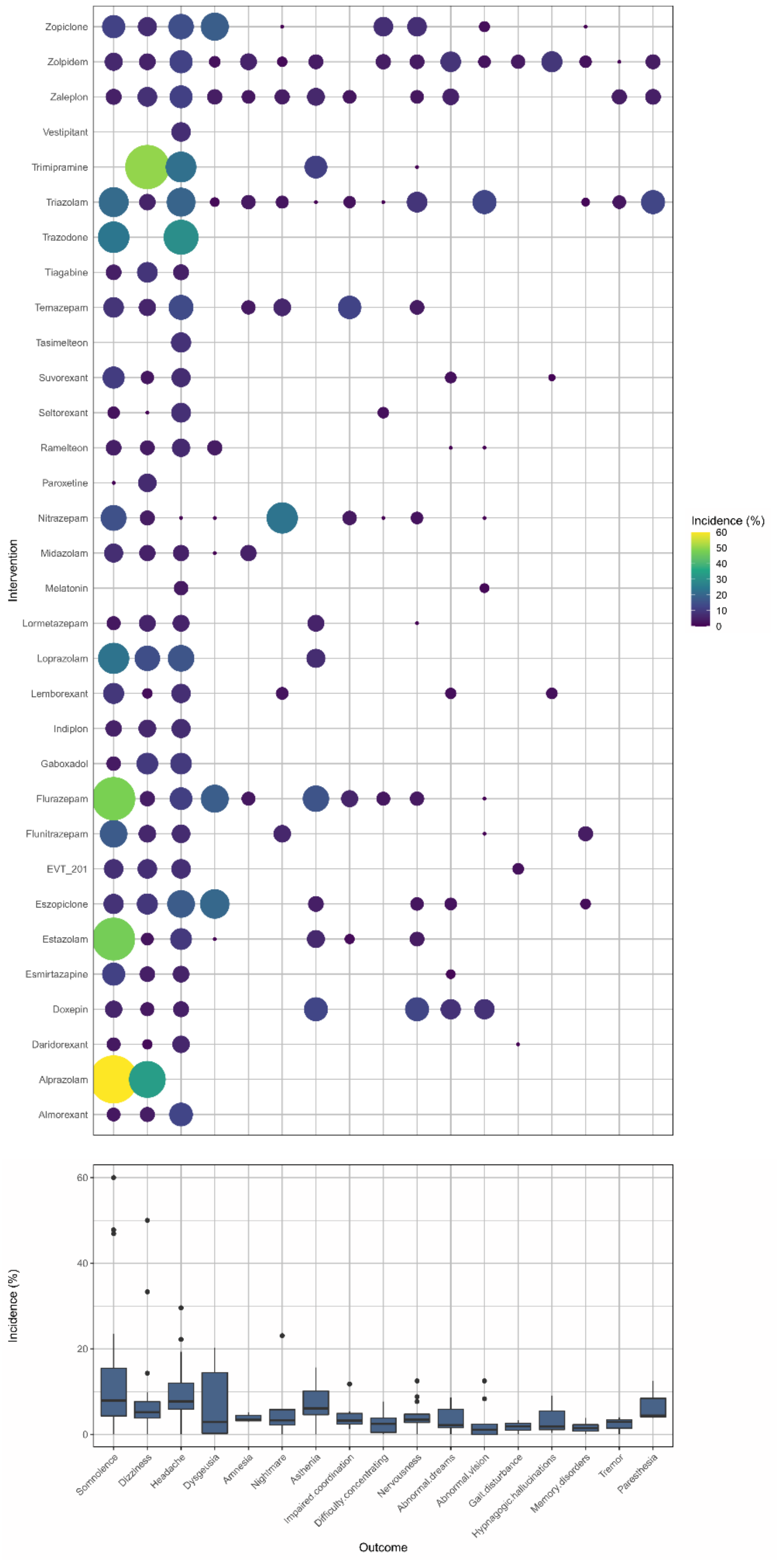

The incidence of injury, poisoning and procedural complications

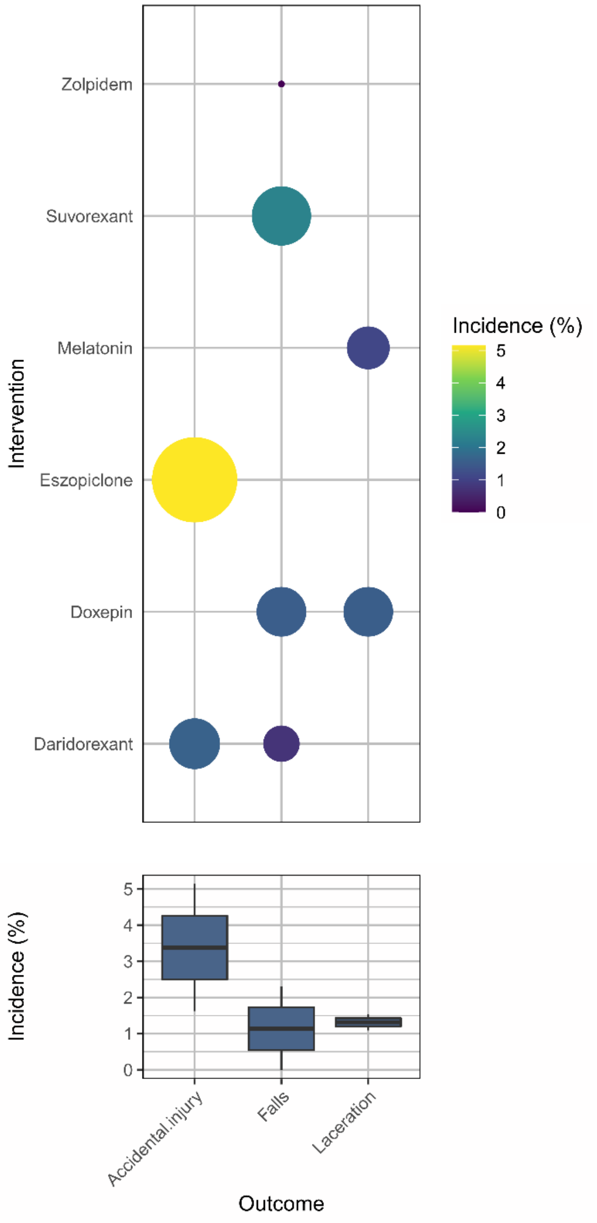

The incidence of musculoskeletal and connective tissue disorders

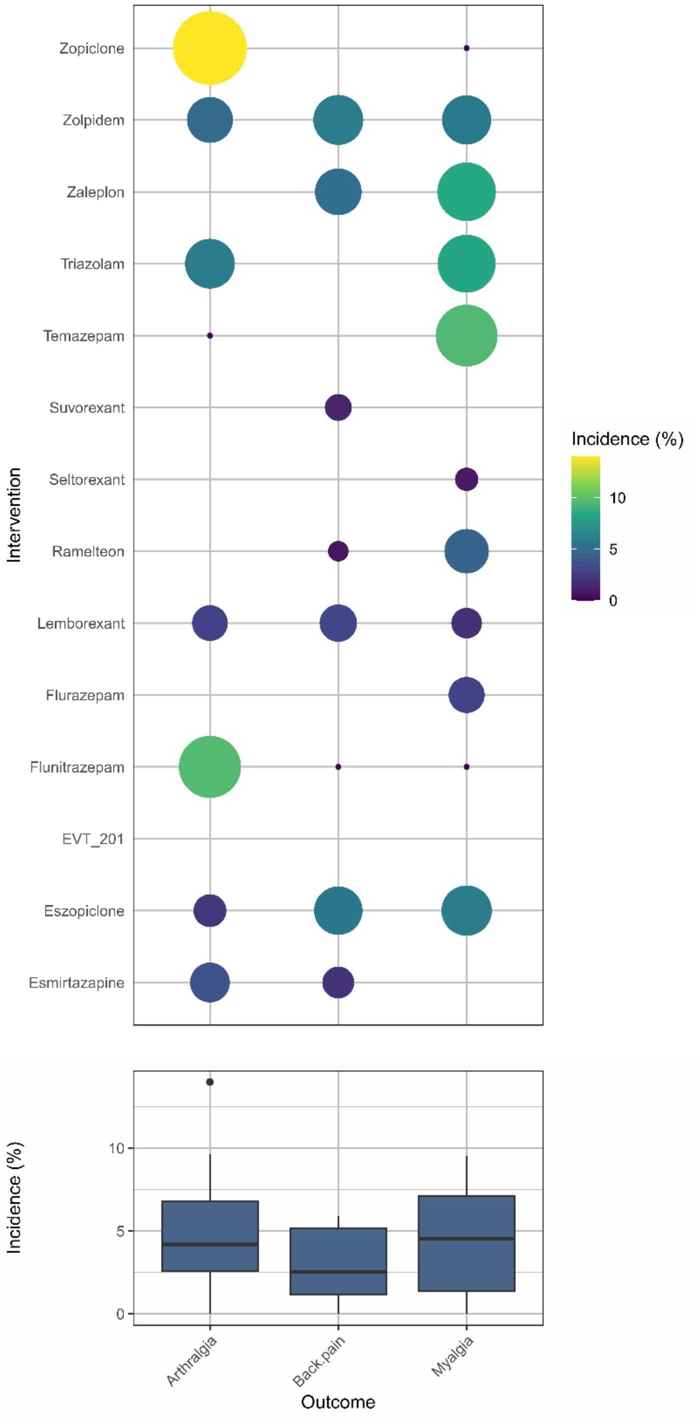

The incidence of eye disorders

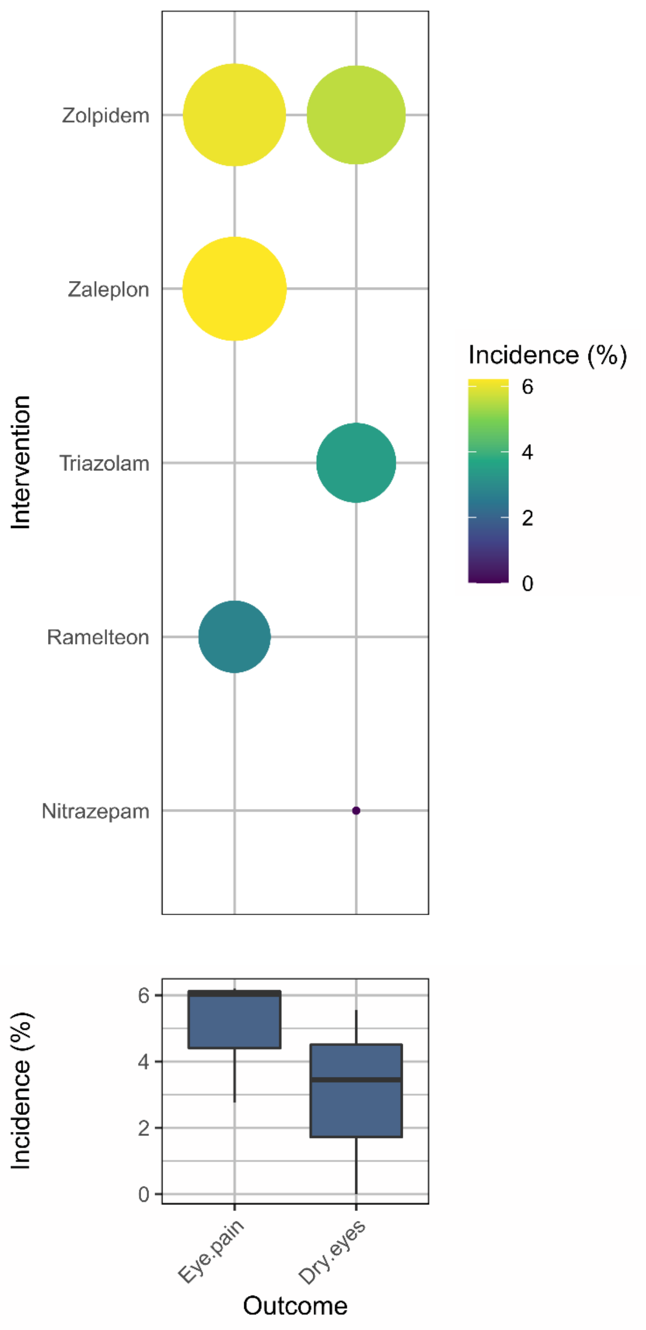

The incidence of infections and infestations

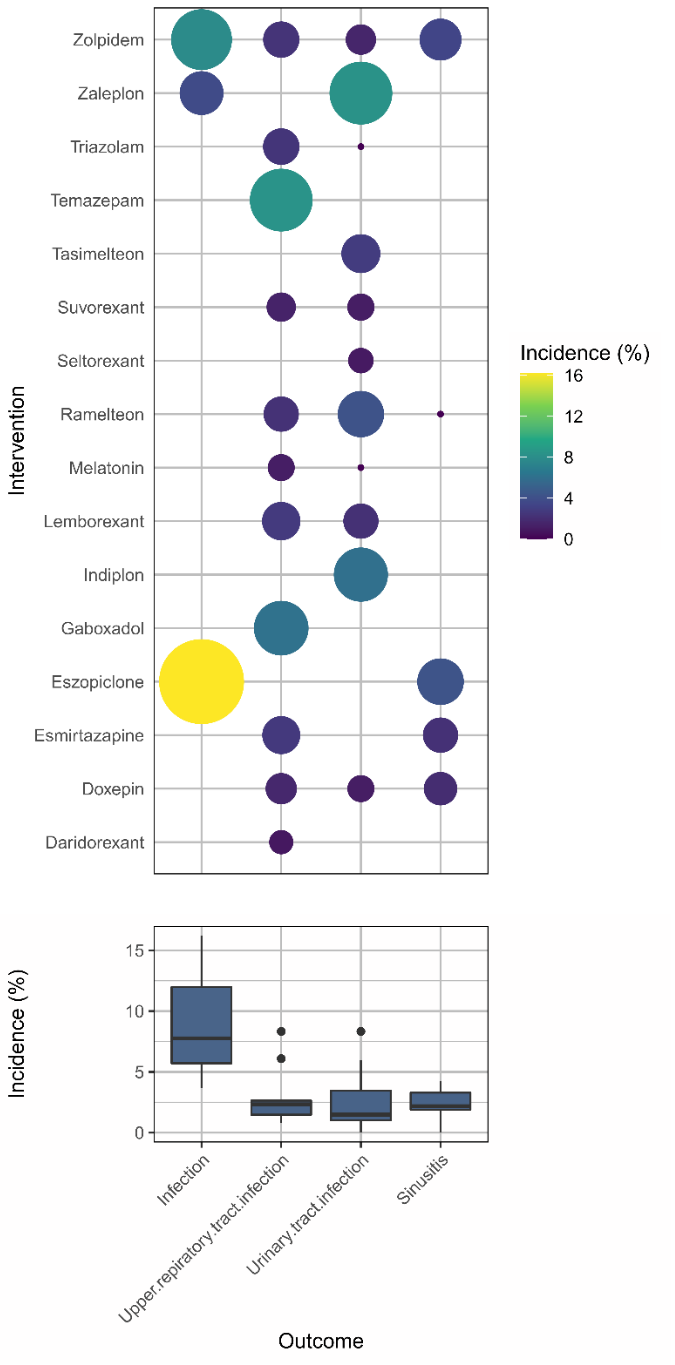

The incidence of sleeping problem

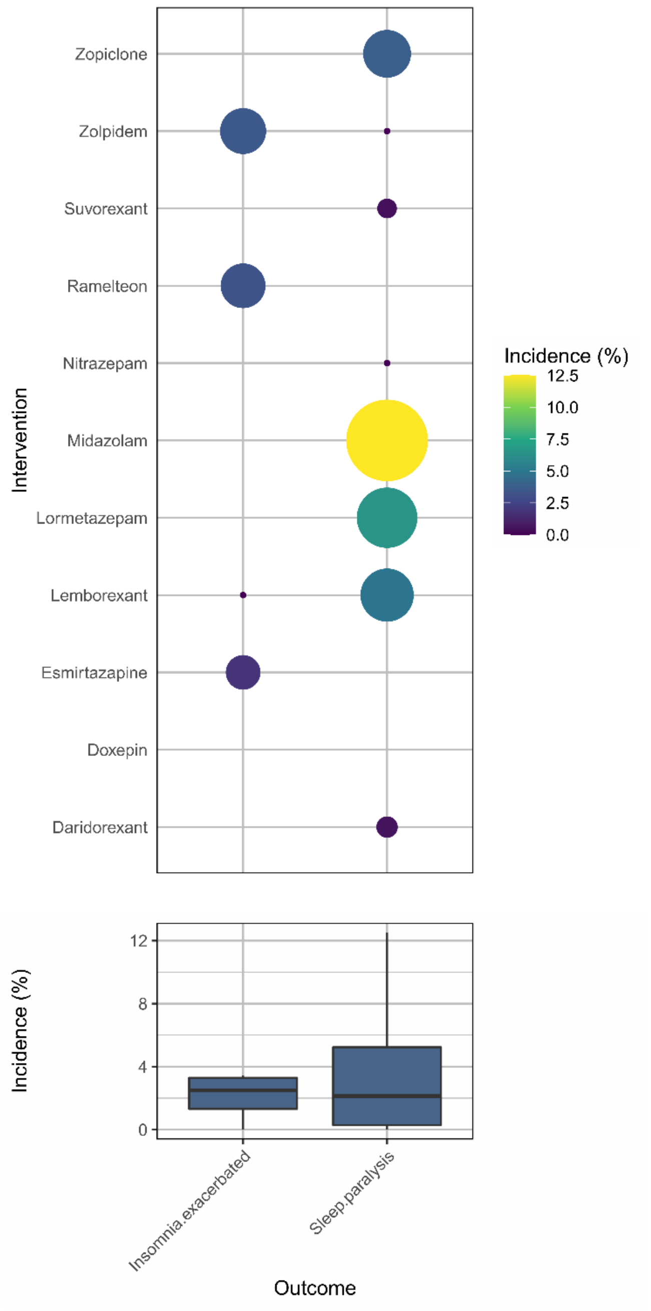

The incidence of investigations

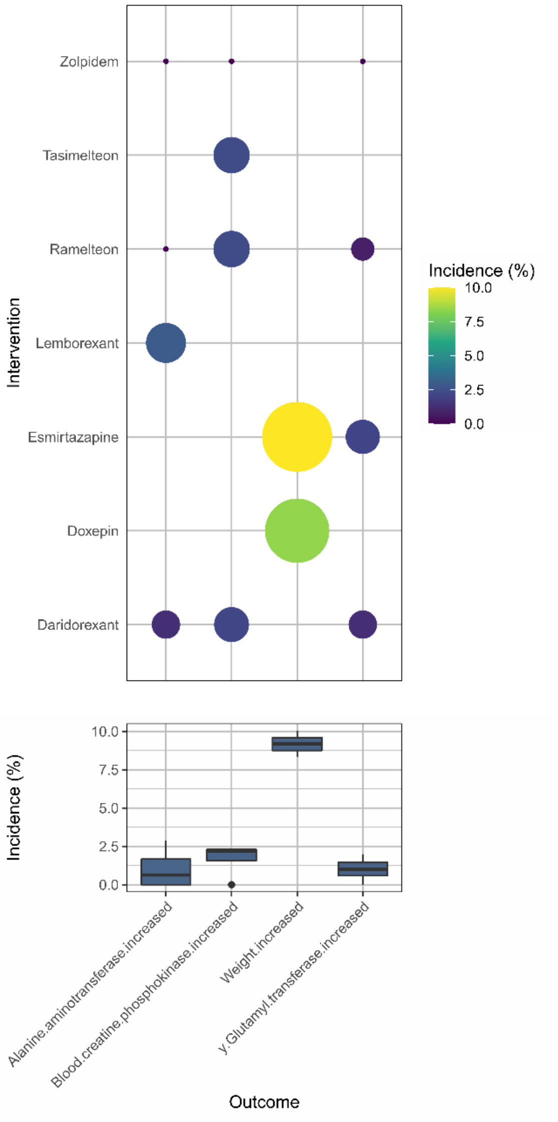

The incidence of skin and subcutaneous tissue disorders

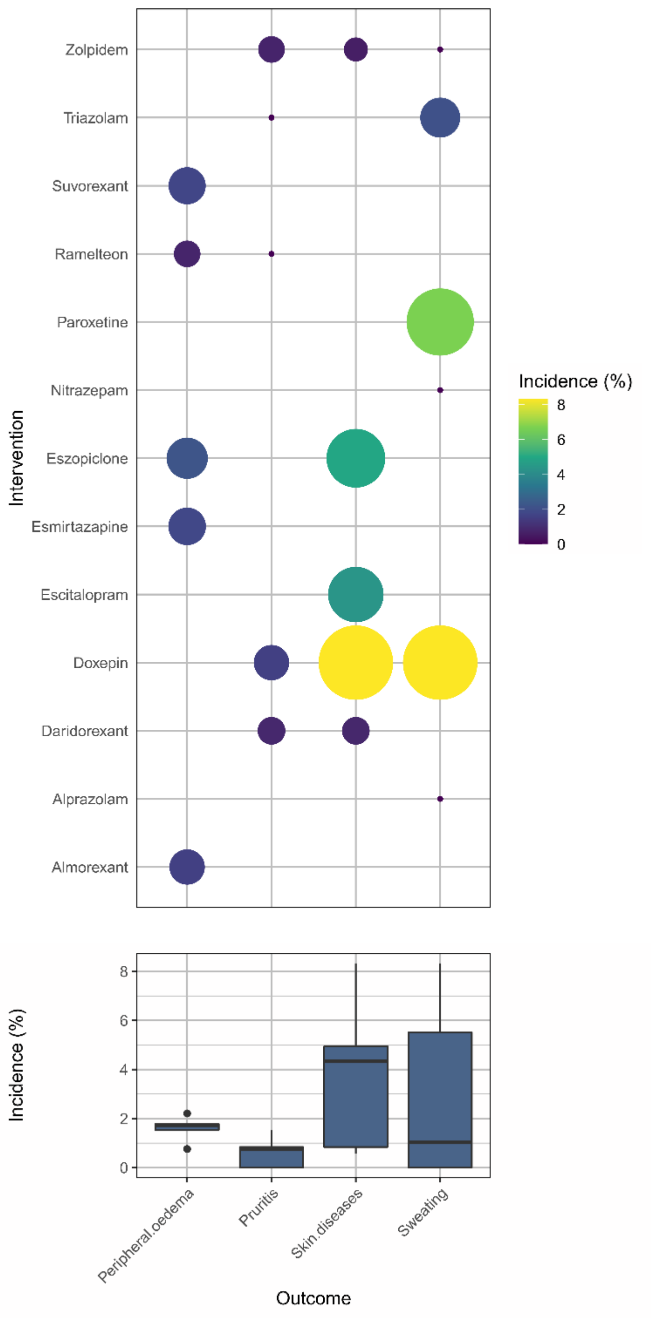

The incidence of other adverse events

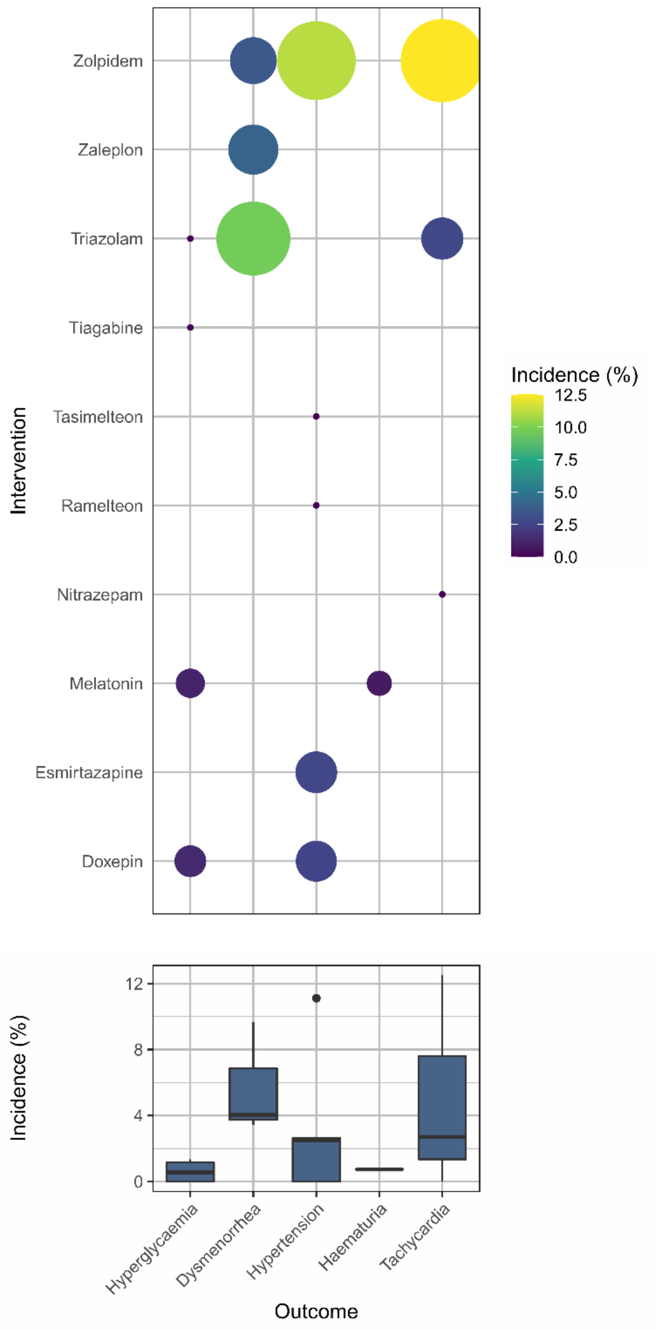

Appendix 7 Principle for risk of bias assessment

**Item 1: Was the allocation sequence adequately generated?**
**1) Definitely Yes:**<br>Trials that assign participants to alternative interventions using a randomly generated sequence. Examples of methods for developing a randomly generated allocation sequence include a random number generator, random number table, coin tossing, shuffling cards or envelopes, and throwing dice. If a trial is described as 'randomized' without any additional details related to how the allocation sequence was developed, we will assume that the allocation sequence was appropriately developed.<br>*Minimization may be implemented without a random element, and this is considered to be equivalent to being random.
**2) Probably Yes: NONE**
**3) Probably No:**<br>* A simple statement such as 'we randomly allocated' or 'using a randomized design' is often insufficient to be confident that the allocation sequence was genuinely randomized
**4) Definitely No:**<br>The investigators describe a non-random component in the sequence generation process. Usually, the description would involve some systematic, non-random approach, for example:<br>• Sequence generated by odd or even date of birth;<br>• Sequence generated by some rule based on date (or day) of admission;<br>• Sequence generated by some rule based on hospital or clinic record number.<br>Other non-random approaches happen much less frequently than the systematic approaches mentioned above and tend to be obvious. They usually involve judgement or some method of non-random categorization of participants, for example:<br>• Allocation by judgement of the clinician;<br>• Allocation by preference of the participant;<br>• Allocation based on the results of a laboratory test or a series of tests;<br>• Allocation by availability of the intervention.
**Item 2: Was the allocation adequately concealed?**
**1) Definitely Yes:**<br>Participants and investigators enrolling participants could not foresee assignment because one of the following, or an equivalent method, was used to conceal allocation:<br>• Central allocation (including telephone, web-based, and pharmacy-controlled, randomization);<br>• Sequentially numbered drug containers of identical appearance;<br>• Sequentially numbered, opaque, sealed envelopes.
**2) Probably Yes:**<br>Trials in which healthcare providers were blind to the intervention but which provide no information on allocation concealment and in which there are no major baseline imbalances.
**3) Probably No:**<br>Insufficient information to permit judgement of risk of bias. This is usually the case if the method of concealment is not described or not described in sufficient detail to allow a definite judgement.
**4) Definitely No:**<br>Participants or investigators enrolling participants could possibly foresee assignments and thus introduce selection bias, such as allocation based on:<br>• Using an open random allocation schedule (e.g. a list of random numbers);<br>• Assignment envelopes were used without appropriate safeguards (e.g. if envelopes were unsealed or nonopaque or not sequentially numbered);

• Alternation or rotation;<br>• Date of birth;<br>• Case record number;<br>• Any other explicitly unconcealed procedure.
**Item 3: Blinding of patients and healthcare providers**
**1) Definitely Yes:**<br>Any one of the following:<br>• No blinding or incomplete blinding of participants and personnel, but the review authors judge that the outcome is not likely to be influenced by lack of blinding;<br>• Blinding of participants and key study personnel ensured, and unlikely that the blinding could have been broken;<br>• If it is described as "double-blind," or "double-dummy"<br>• Explicit statement that a group of interest was blinded → LOW risk of bias for that group<br>• Explicit statement "investigators were blinded → LOW risk of bias for study personnel
**2) Probably Yes: NONE**
**3) Probably No:**<br>• Insufficient information to permit judgment<br>• Therapy trials in which healthcare providers are described as being blind to the intervention but allocation concealment was inadequate.
**4) Definitely No:**<br>Any one of the following:<br>• No blinding or incomplete blinding, and the outcome or outcome measurement is likely to be influenced by lack of blinding.<br>• Blinding of key study participants and personnel attempted, but likely that the blinding could have been broken;<br>• Either participants or some key study personnel were not blinded, and the non-blinding of others likely to introduce bias.<br>• Explicit statement that a group of interest was not blinded<br>• Explicit description of the trial as "open label" or "unblinded", or "single blinded"<br>Please note, if the outcome is an objective outcome (e.g., PSG outcomes), the risk of bias will generally be of less concern
**Item 4: Blinding of outcome assessors**
**1) Definitely Yes:**<br>Any one of the following:<br>• No blinding of outcome assessment, but the review authors judge that the outcome measurement is not likely to be influenced by lack of blinding;<br>• Blinding of outcome assessment ensured, and unlikely that the blinding could have been broken.<br>Explicit statement "investigators were blinded → LOW risk of bias for outcome assessors<br>No explicit statement about blinding status of either patients, health care providers, data collectors, or outcome adjudicators, and:<br>• Placebo controlled drug trial → LOW risk of bias for those groups<br>• Active control drug trial (A vs. B) and mention of "double -dummy" or that medications were identical or matched → LOW risk of bias for those groups
**2) Probably Yes:**
**3) Probably No:**<br>Any one of the following:<br>• Insufficient information to permit judgment;<br>No explicit statement about blinding status of either patients, health care providers, data collectors, or outcome adjudicators, and:<br>• Active control drug trial (A vs. B) but no mention of "double-dummy" or that medications were identical or matched

4) Definitely No:
Any one of the following:

- No blinding of outcome assessment, and the outcome measurement is likely to be influenced by lack of blinding;
- Blinding of outcome assessment, but likely that the blinding could have been broken, and the outcome measurement is likely to be influenced by lack of blinding.

Explicit statement that a group of interest was not blinded
Explicit description of the trial as "open label" or "unblinded"

Item 5: Was loss to follow-up (missing outcome data) infrequent?

1) Definitely Yes:
Trials in which missing outcome data (including outcome data that has been imputed) < 10%.

2) Probably Yes:
Trials in which missing outcome data (including outcome data that has been imputed) is between 10% to 15% and missing outcome data is unlikely to be related to the true outcome and there is no imbalance in numbers of or reasons for missing data across intervention groups.

3) Probably No:
Trials in which missing outcome data (including outcome data that has been imputed) is between 10% to 15% and missing outcome data is likely to be related to the true outcome or there are imbalances in numbers of or reasons for missing data across intervention groups.

4) Definitely No:
Trials in which missing outcome data (including outcome data that has been imputed) > 15%.

Item 6: Are reports of the study free of selective outcome reporting?

1) Definitely Yes:
Results for outcomes that were analyzed and reported according to a pre-specified statistical analysis plan or protocol (including the timepoint for the measurement of the outcome).

2) Probably Yes:
Results for outcomes that were analyzed and reported but that were not prespecified in a statistical analysis plan or protocol but the timepoint at which results are reported is consistent with the timepoint for other outcomes in the trial report or there is little reason to believe the outcome was selectively reported.
Please note that outcomes that were not prespecified in a protocol or statistical analysis plan and that are reported in the trial preprint or publication should be rated at probably low risk of bias unless there are other important reasons to suspect that results for those outcomes were selectively reported (e.g., results are presented at timepoints that don't match the timepoints reported for other outcomes).

3) Probably No:
Results for outcomes that were analyzed and reported but that were not prespecified in a statistical analysis plan or protocol but the timepoint at which results are reported is not consistent with the timepoint for other outcomes in the trial report or there are other reasons to believe that the outcome is selectively reported.

4) Definitely No:
Results for outcomes that were analyzed and reported for which there are inconsistencies with the statistical analysis plan or protocol. These inconsistencies may include outcome measures of interest or the timepoints for the measurement of outcomes.

Appendix 8 Results of risk of bias assessment

Author, yr	Allocation sequence generated	allocation concealment	Blinding of patients ans health providers	Blinding of outcome assessors	Incomplete data	selective outcome reporting	overal
Black, 2017	Definitely yes	Definitely yes	Definitely yes	Definitely yes	Probably yes	Probably yes	Low
Pinto, 2016	Definitely yes	Probably yes	Definitely yes	Definitely yes	Definitely no	Probably yes	High
Michelson, 2014	Definitely yes	Definitely yes	Definitely yes	Definitely yes	Definitely yes	Probably yes	Low
Roth, 2006a	Definitely no	Definitely yes	Definitely yes	Definitely yes	Definitely yes	Probably yes	High
Roth, 2006b	Definitely no	Probably no	Definitely yes	Definitely yes	Definitely yes	Probably yes	High
Roth, 2006c	Definitely no	Probably yes	Definitely yes	Definitely yes	Probably yes	Probably yes	High
Roth, 2007	Definitely no	Probably yes	Definitely yes	Definitely yes	Probably yes	Probably yes	High
Roth, 2010a	Definitely Yes	Definitely yes	Definitely yes	Definitely yes	Definitely no	Definitely yes	High
Roth, 2010b	Definitely Yes	Definitely yes	Definitely yes	Definitely yes	Definitely no	Definitely yes	High
Roth, 2013	Definitely yes	Definitely yes	Definitely yes	Definitely yes	Definitely yes	Probably yes	Low
Huang, 2011	Definitely yes	Probably yes	Definitely yes	Definitely yes	Definitely yes	Probably yes	Low
Uchiyama, 2010b	Definitely yes	Definitely yes	Definitely yes	Definitely yes	Definitely yes	Probably yes	Low
Lankford, 2012	Definitely yes	Definitely yes	Definitely yes	Definitely yes	Definitely yes	Definitely yes	Low
Krystal, 2003	Definitely no	Definitely yes	Definitely yes	Definitely yes	Definitely no	Probably yes	High
Krystal, 2008	Definitely yes	Definitely yes	Definitely yes	Definitely yes	Definitely no	Probably yes	High
Krystal, 2010	Definitely yes	Probably yes	Definitely yes	Definitely yes	Probably yes	Probably yes	Low
Krystal, 2011	Definitely yes	Definitely yes	Definitely yes	Definitely yes	Probably yes	Definitely yes	Low
Wade, 2007	Definitely yes	Probably yes	Definitely yes	Definitely yes	Probably yes	Probably yes	Low
Walsh, 1998a	Definitely yes	Probably yes	Definitely yes	Definitely yes	Probably yes	Probably yes	Low
Walsh, 1998b	Definitely no	Probably yes	Definitely yes	Definitely yes	Definitely yes	Probably yes	High
Walsh, 2006	Definitely no	Probably yes	Definitely yes	Definitely yes	Definitely yes	Probably yes	High
Walsh, 2007a	Definitely no	Probably yes	Definitely yes	Definitely yes	Probably yes	Probably yes	High
Walsh, 2007b	Definitely yes	Probably yes	Definitely yes	Definitely yes	Definitely yes	Probably yes	Low

Walsh, 2008	Definitely no	Probably yes	Definitely yes	Definitely yes	Definitely yes	Probably yes	High
Walsh, 2010	Definitely no	Probably yes	Definitely yes	Definitely yes	Definitely yes	Probably yes	High
Mayer, 2009	Definitely no	Probably yes	Definitely yes	Definitely yes	Definitely yes	Probably yes	High
Zammit, 2004	Definitely no	Definitely yes	Definitely yes	Definitely yes	Definitely yes	Probably yes	High
Zammit, 2007	Definitely yes	Probably yes	Definitely yes	Definitely yes	Probably yes	Probably yes	Low
Scharf, 1990	Definitely yes	Probably yes	Definitely yes	Probably no	Definitely yes	Definitely yes	High
Scharf, 1994	Definitely no	Definitely yes	Definitely yes	Definitely yes	Definitely yes	Probably yes	High
Scharf, 2006	Definitely no	Definitely yes	Definitely yes	Definitely yes	Definitely yes	Probably yes	High
Scharf, 2007	Definitely yes	Probably yes	Definitely yes	Definitely yes	Definitely no	Probably yes	High
McCall, 2006	Definitely no	Probably yes	Definitely yes	Definitely yes	Definitely yes	Probably yes	High
Fry, 2000	Definitely no	Probably yes	Definitely yes	Definitely yes	Definitely yes	Probably yes	High
Elie, 1999	Definitely no	Probably yes	Definitely yes	Definitely yes	Definitely yes	Probably yes	High
Lahmeyer, 1997	Definitely yes	Probably yes	Definitely yes	Definitely yes	Definitely no	Probably yes	High
Ancoli-Israel, 2010	Definitely yes	Probably yes	Definitely yes	Definitely yes	Definitely no	Probably yes	High
Riemann, 2002	Definitely no	Probably yes	Definitely yes	Definitely yes	Definitely yes	Probably yes	High
Luthringer, 2010	Definitely no	Probably yes	Definitely yes	Definitely yes	Definitely yes	Probably yes	High
Zhou, 2002	Definitely no	Probably no	Definitely yes	Definitely yes	Definitely no	Probably yes	High
Hajak, 2001	Definitely no	Probably yes	Definitely yes	Definitely yes	Probably yes	Probably yes	High
Hajak, 2009	Definitely yes	Definitely yes	Definitely yes	Definitely yes	Probably yes	Probably yes	Low
Lydiard, 2006	Definitely no	Probably yes	Definitely yes	Definitely yes	Probably yes	Probably yes	High
Fan, 2017	Definitely yes	Definitely yes	Definitely yes	Definitely yes	Definitely yes	Probably yes	Low
Allain, 2001	Definitely no	Definitely yes	Definitely yes	Definitely yes	Definitely yes	Probably yes	High
Allain, 1998	Definitely yes	Definitely yes	Definitely yes	Definitely yes	Definitely no	Definitely yes	High
Herrmann, 1993	Definitely no	Probably no	Definitely yes	Definitely yes	Definitely yes	Probably yes	High
Sivertsen, 2006	Definitely yes	Definitely yes	Definitely yes	Definitely yes	Definitely yes	Probably yes	Low
Morin, 2003	Definitely no	Probably yes	Definitely yes	Definitely yes	Probably yes	Probably yes	High
Herring, 2016a	Definitely yes	Definitely yes	Definitely yes	Definitely yes	Probably yes	Probably yes	Low

Herring, 2016b	Definitely yes	Definitely yes	Definitely yes	Definitely yes	Probably yes	Probably yes	Low
Leppik, 1997	Definitely no	Probably yes	Definitely yes	Definitely yes	Probably yes	Definitely yes	High
Dockhorn, 1996	Definitely no	Probably yes	Definitely yes	Definitely yes	Definitely yes	Definitely yes	High
Tamminen, 1987	Definitely no	Probably yes	Definitely yes	Probably no	Definitely no	Definitely yes	High
Jovanovic, 1983	Definitely no	Probably yes	Definitely yes	Probably no	Definitely yes	Definitely yes	High
Ivgy-May, 2015a	Definitely yes	Definitely yes	Definitely yes	Definitely yes	Probably yes	Definitely yes	Low
Ivgy-May, 2015b	Definitely yes	Definitely yes	Definitel y yes	Definitel y yes	Probably yes	Definitel y yes	Low
Ivgy-May, 2020	Definitely yes	Definitely yes	Definitely yes	Definitely yes	Definitely no	Definitely yes	High
NCT005483 40	Definitely no	Probably yes	Definitely yes	Definitely yes	Definitely yes	Definitely yes	High
NCT014630 98	Definitely no	Probably yes	Definitely yes	Definitely yes	Definitely yes	Definitely yes	High
Kärppä, 2020	Definitely yes	Probably yes	Definitely yes	Definitely yes	Definitely no	Definitely yes	High
Dauvilliers, 2020	Definitely yes	Definitely yes	Definitely yes	Definitely yes	Definitely yes	Definitely yes	Low
Soares, 2006	Definitely yes	Probably yes	Definitely yes	Definitely yes	Probably yes	Definitely yes	Low
Murphy, 2017	Definitely yes	Probably yes	Definitely yes	Definitely yes	Definitely yes	Probably yes	Low
Ratti, 2013	Definitely yes	Probably yes	Definitely yes	Definitely yes	Probably yes	Probably yes	Low
Lemoine, 2007	Definitely no	Probably yes	Definitely yes	Definitely yes	Definitely yes	Probably yes	High
Fleming, 1995	Definitely no	Probably yes	Definitely yes	Definitely yes	Definitely yes	Probably yes	High
Tsutsui, 2001	Definitely no	Probably yes	Definitely yes	Definitely yes	Probably yes	Probably yes	High
Zhang, 2004	Definitely yes	Probably yes	Definitely yes	Probably no	Definitely yes	Probably yes	High
Uchimura, 2011	Definitely yes	Probably yes	Definitely yes	Definitely yes	Definitely yes	Definitely yes	Low
Dehlin, 1995	Definitely no	Probably yes	Definitely yes	Probably no	Definitely yes	Definitely yes	High
Klimm, 1987	Definitely no	Probably yes	Definitely yes	Probably no	Definitely yes	Definitely yes	High
Takeda, 2007	Definitely yes	Probably yes	Definitely yes	Definitely yes	Definitely yes	Probably yes	Low
Katz, 2011	Definitely yes	Probably yes	Definitely yes	Definitely yes	Definitely yes	Probably yes	Low
Cordingley, 1984	Definitely yes	Probably yes	Definitely yes	Probably no	Definitely yes	Definitely yes	High
Dahl, 1982	Probably no	Probably yes	Definitely yes	Probably no	Definitely yes	Definitely yes	High
Elie, 1990	Definitely no	Probably no	Definitely yes	Definitely no	Definitely yes	Definitely yes	High

Fabre, 1977	Definitely yes	Definitely yes	Definitely yes	Probably no	Definitely no	Probably no	High
Anderson, 1987	Probably no	Probably yes	Probably no	Definitely yes	Definitely yes	Probably no	High
Allen, 1987	Probably no	Probably no	Probably no	Probably no	Definitely yes	Probably no	High
Bayer, 1986	Probably no	Probably no	Definitely yes	Probably no	Definitely no	Definitely no	High
Dominguez, 198	Probably no	Probably no	Definitely yes	Definitely yes	Definitely yes	Definitely yes	High
Goldenberg, 1994	Probably no	Probably no	Definitely yes	Probably no	Definitely no	Definitely no	High
Mignot, 2022a	Definitely yes	Definitely yes	Definitely yes	Definitely yes	Probably yes	Definitely yes	Low
Mignot, 2022b	Definitely yes	Definitely yes	Definitely yes	Definitely yes	Probably yes	Definitely yes	Low
Monti, 1994	Probably no	Probably yes	Definitely yes	Probably yes	Definitely yes	Definitely no	High
Richardson, 2009	Probably no	Probably yes	Definitely yes	Probably yes	Probably no	Probably no	High
Wang-Weigand, 2011	Probably no	Probably no	Definitely yes	Probably no	Definitely yes	Probably no	High
Heidrich, 1981	Probably no	Probably no	Definitely yes	Probably no	Definitely yes	Definitely yes	High
Begg, 1992	Probably no	Probably no	Definitely yes	Probably no	Definitely yes	Definitely yes	High
MOON, 1985	Probably no	Probably no	Definitely yes	Probably no	Definitely yes	Definitely yes	High
Roger, 1993	Probably no	Probably no	Definitely yes	Probably no	Definitely yes	Definitely yes	High
NCT00156533	Probably no	Probably yes	Definitely yes	Definitely yes	Definitely no	Definitely no	High
NCT00177216	Probably no	Probably yes	Definitely yes	Definitely yes	Probably no	Definitely yes	High
NCT00383643	Probably no	Probably yes	Definitely yes	Definitely yes	Definitely yes	Definitely yes	Low
NCT00755495	Probably no	Probably yes	Definitely yes	Definitely yes	Definitely yes	Probably no	High
NCT00756002	Probably no	Probably yes	Definitely yes	Definitely yes	Definitely yes	Definitely yes	Low
NCT03375203	Probably no	Probably yes	Definitely yes	Definitely yes	Definitely yes	Probably no	High
NCT03545191	Definitely yes	Definitely yes	Definitely yes	Definitely yes	Definitely yes	Definitely yes	Low
NCT03575104	Definitely yes	Definitely yes	Definitely yes	Definitely yes	Definitely yes	Definitely yes	Low
Rosenberg, 1994	Definitely yes	Definitely yes	Definitely yes	Probably no	Definitely no	Definitely yes	High
Rosenberg, 2019	Definitely yes	Probably yes	Definitely yes	Definitely yes	Definitely yes	Probably yes	Low

Appendix 9 The network evidence plots for each outcome

The network evidence plots for primary outcomes

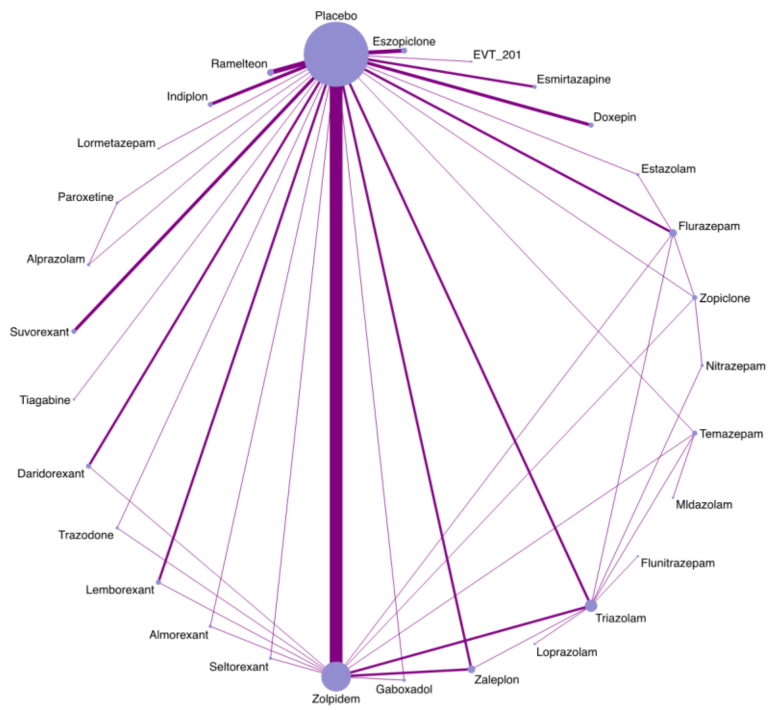

The network evidence plots for Somnolence

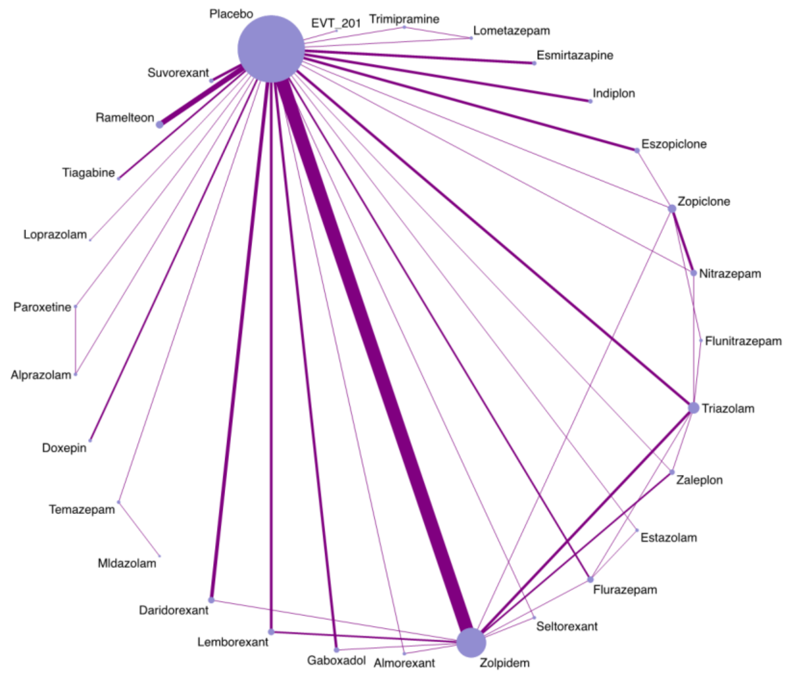

The network evidence plots for Dizziness

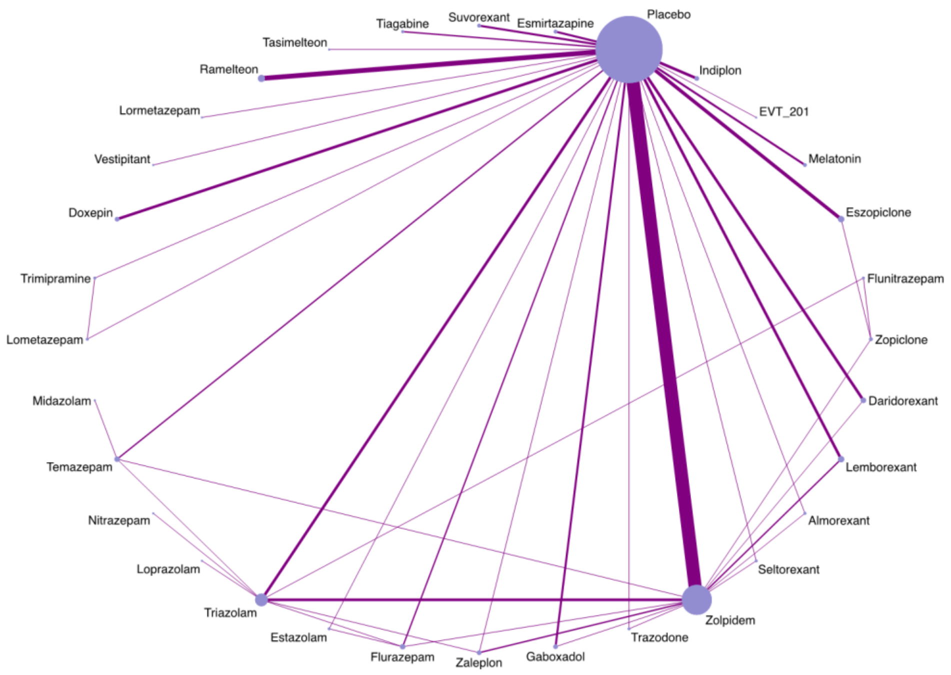

The network evidence plots for Headache

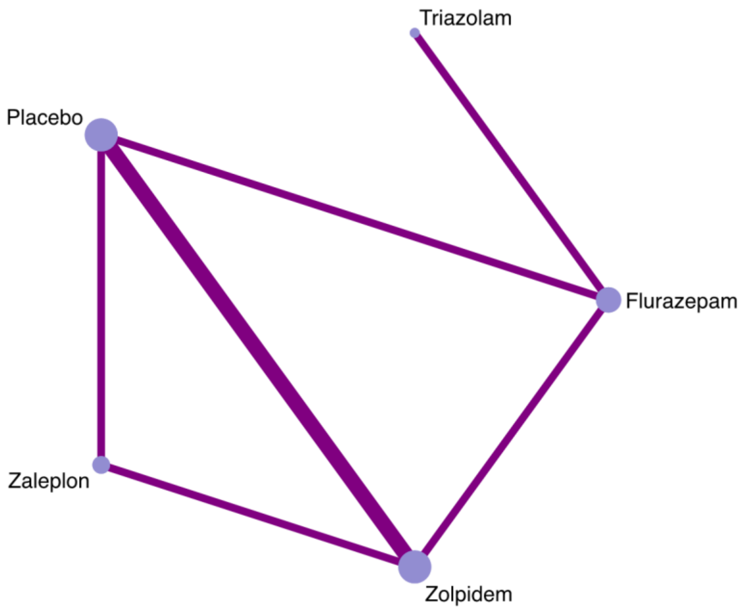

The network evidence plots for Amnesia

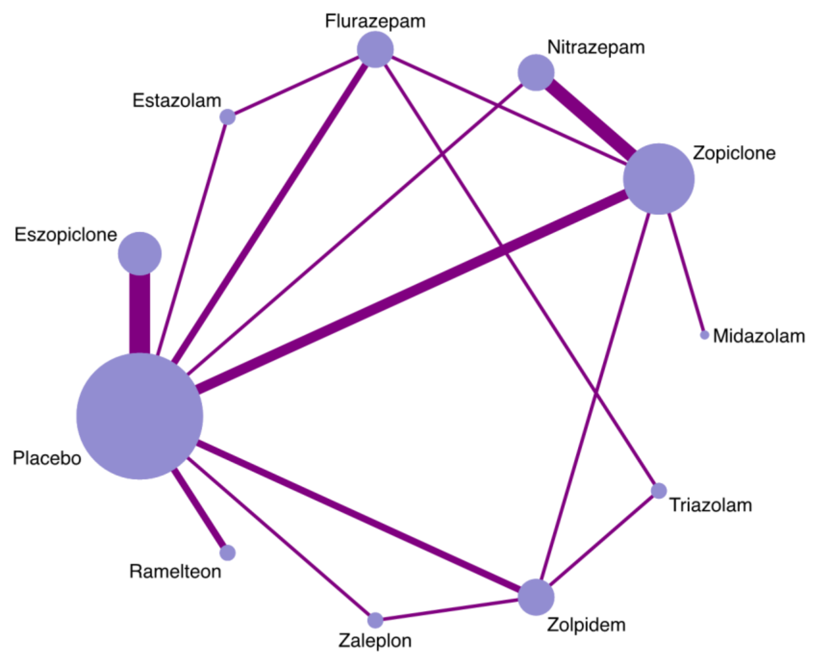

The network evidence plots for dysgeusia

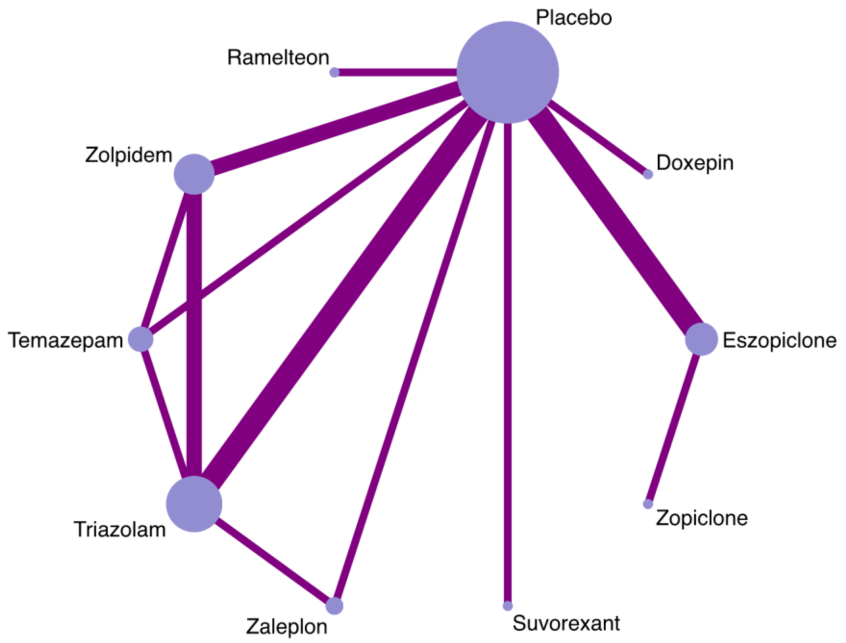

The network evidence plots for dyspepsia

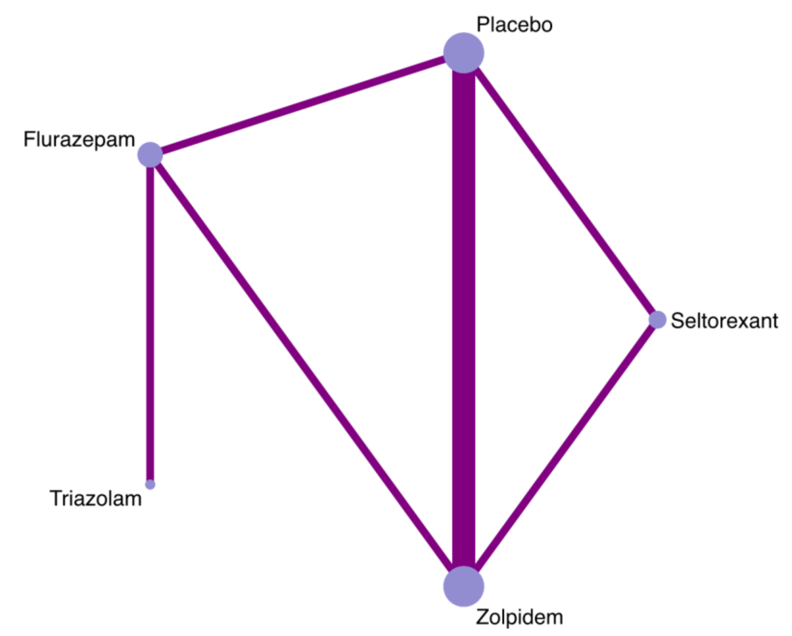

The network evidence plots for difficulty concentrating

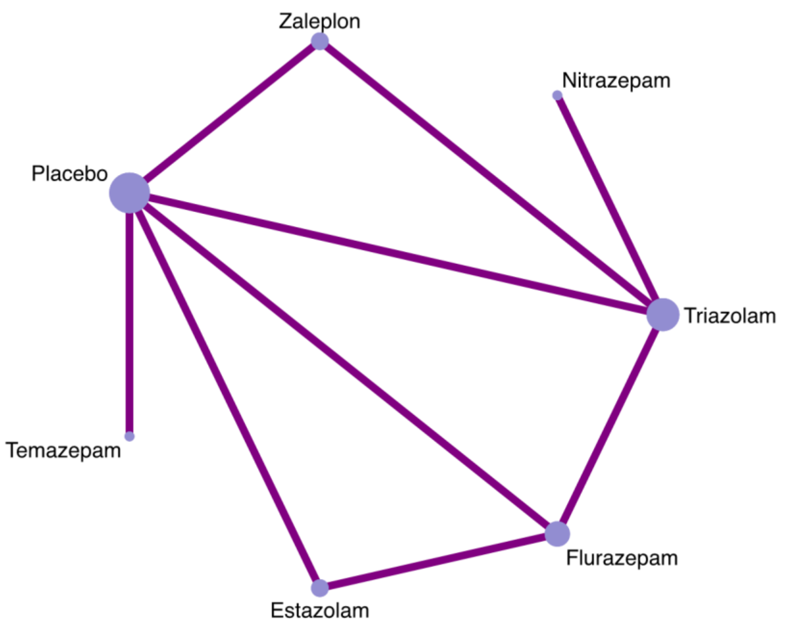

The network evidence plots for impaired coordination

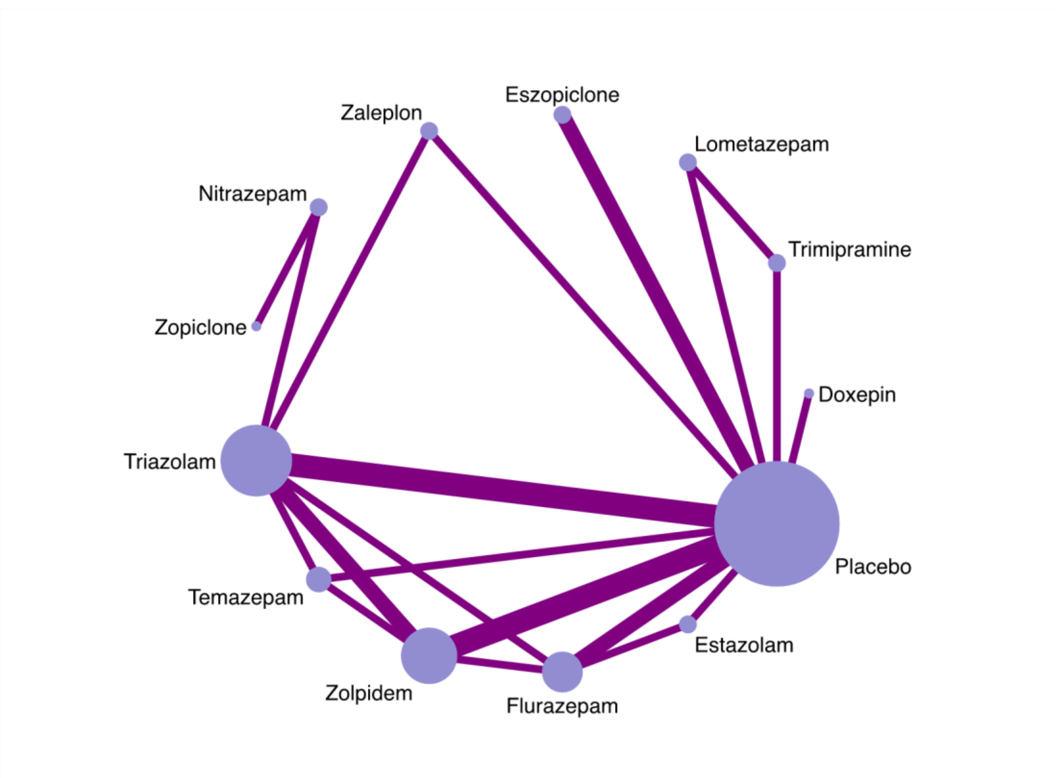

The network evidence plots for nervousness

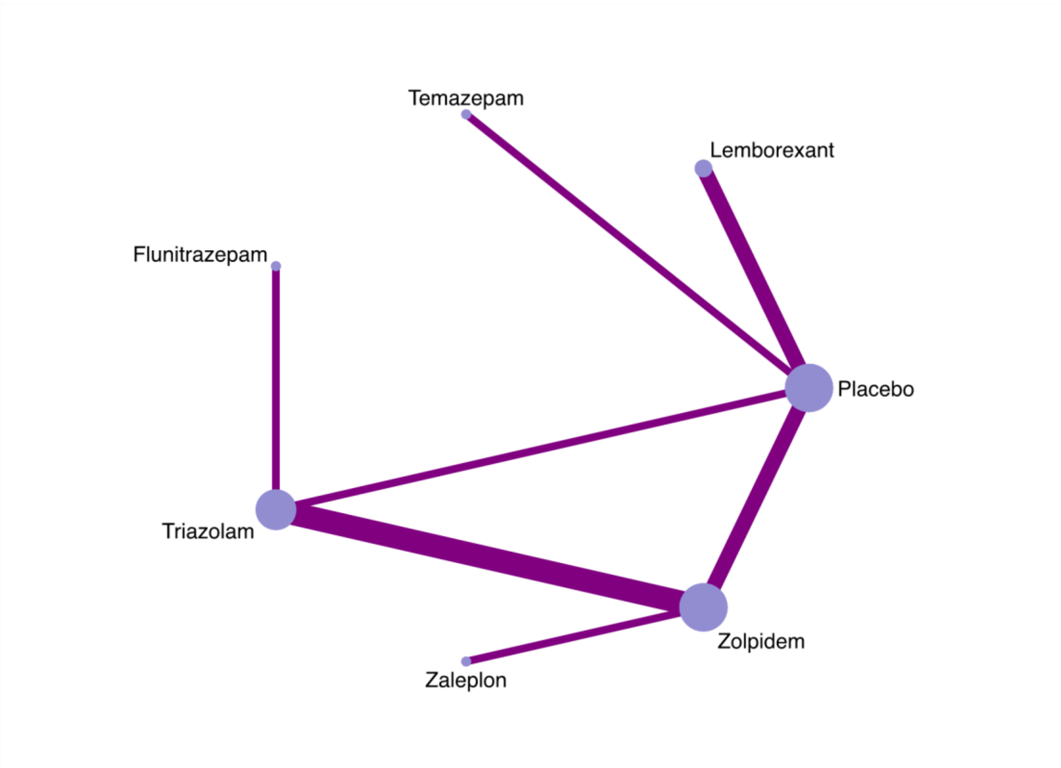

The network evidence plots for nightmare

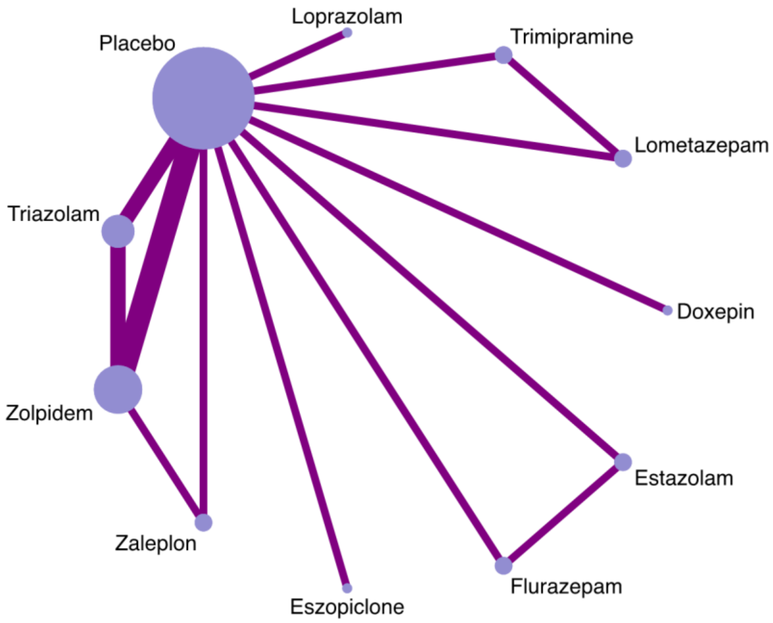

The network evidence plots for Asthenia

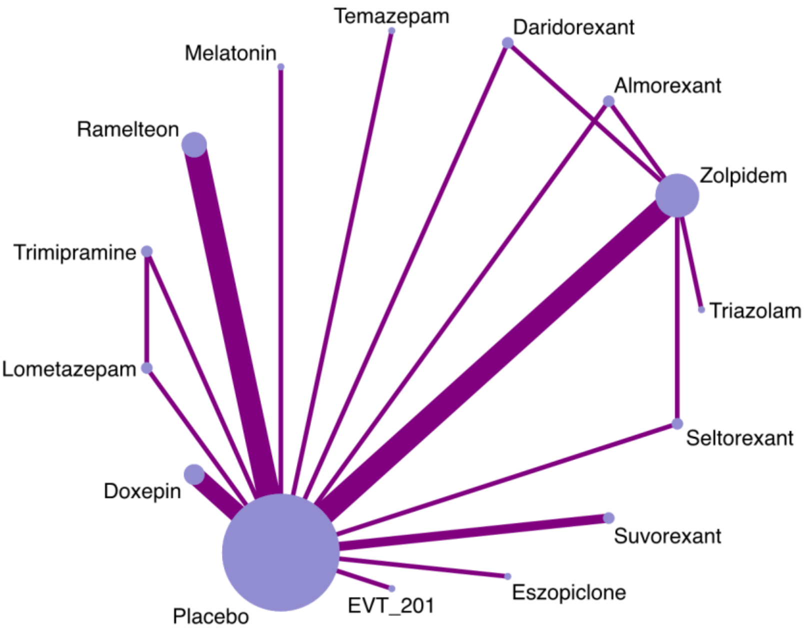

The network evidence plots for diarrhea

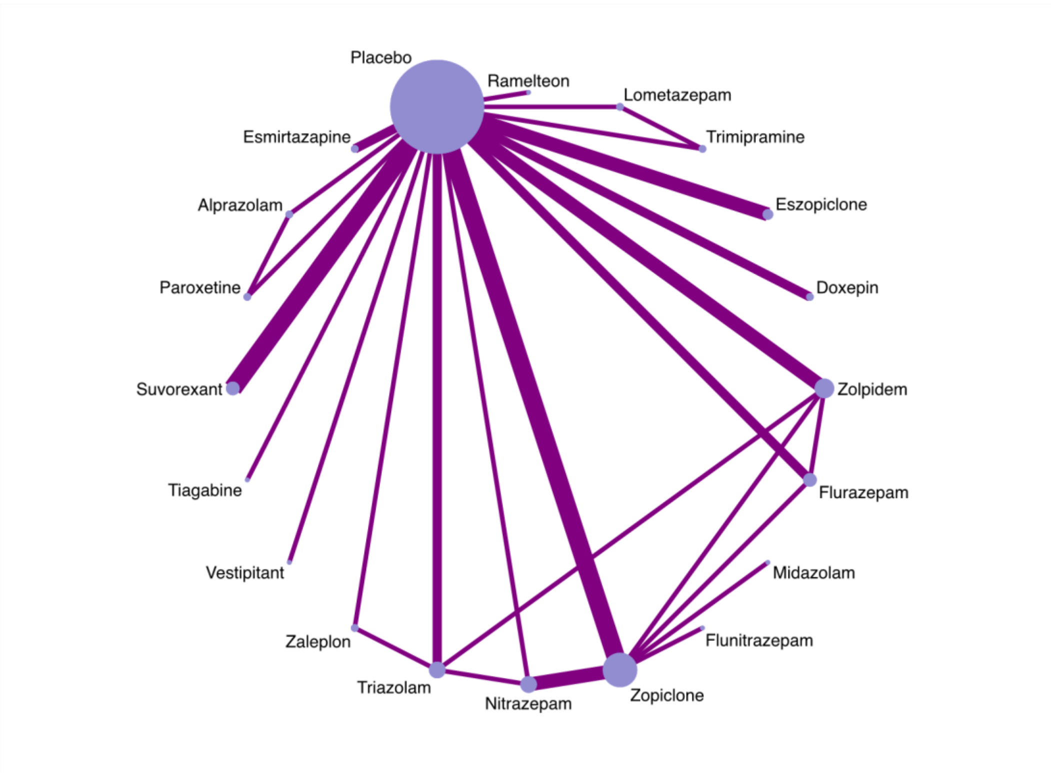

The network evidence plots for dry mouth

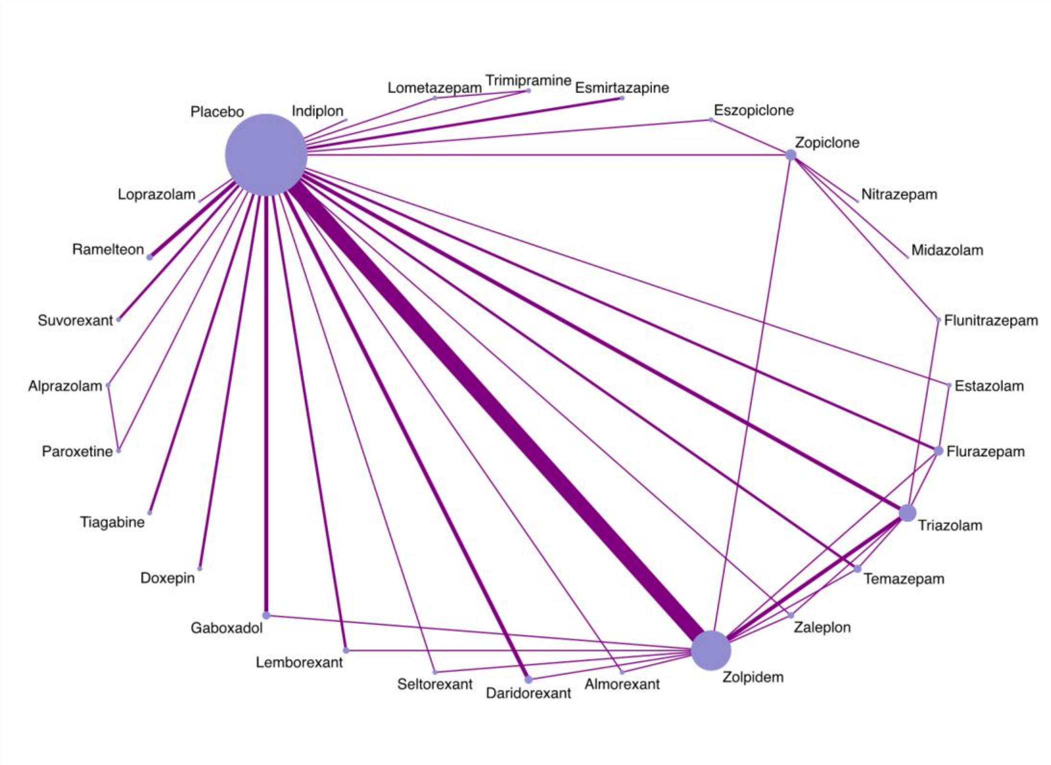

The network evidence plots for nausea/vomiting

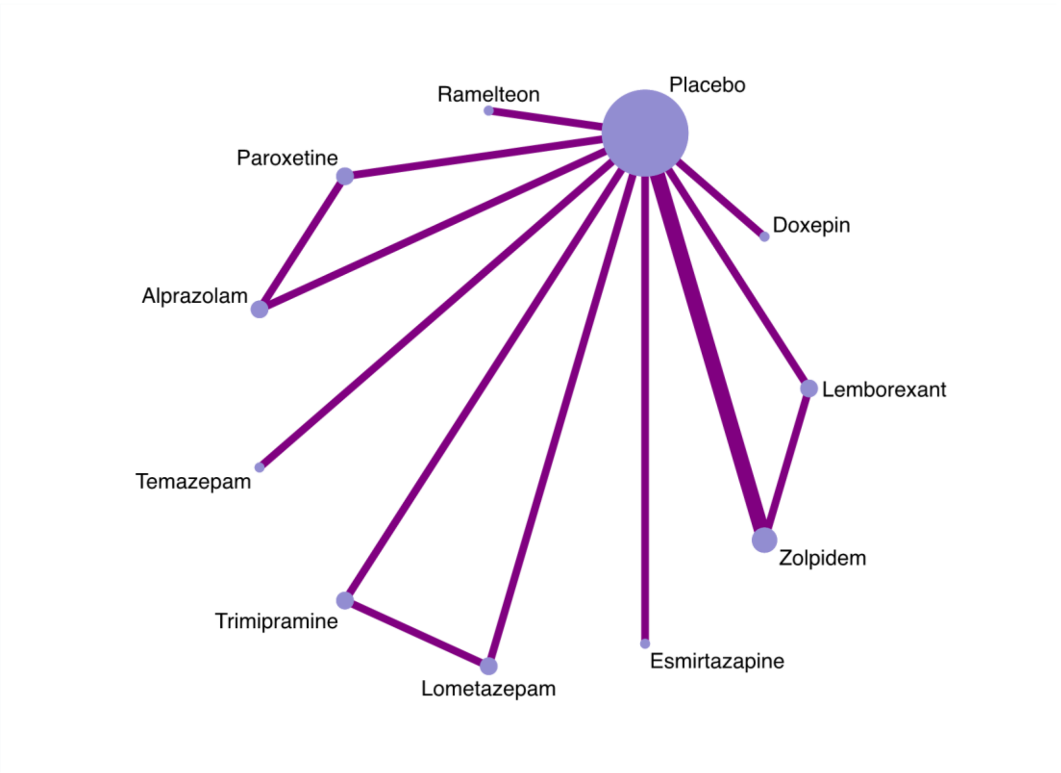

The network evidence plots for constipation

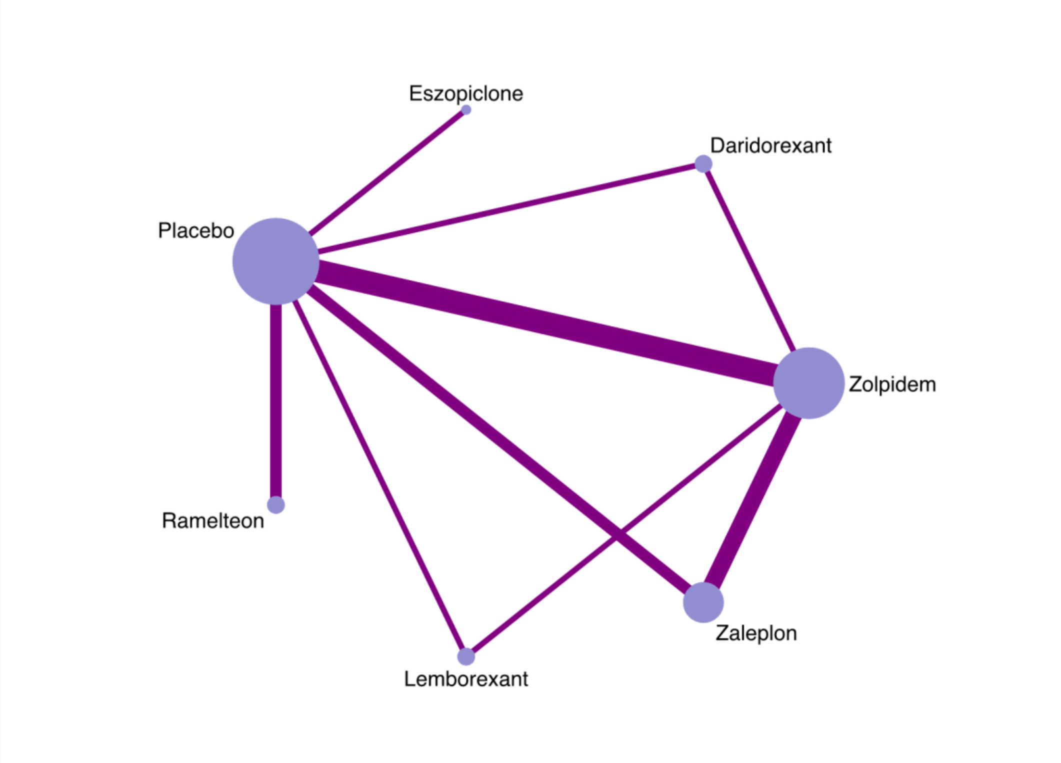

The network evidence plots for abdominal pain

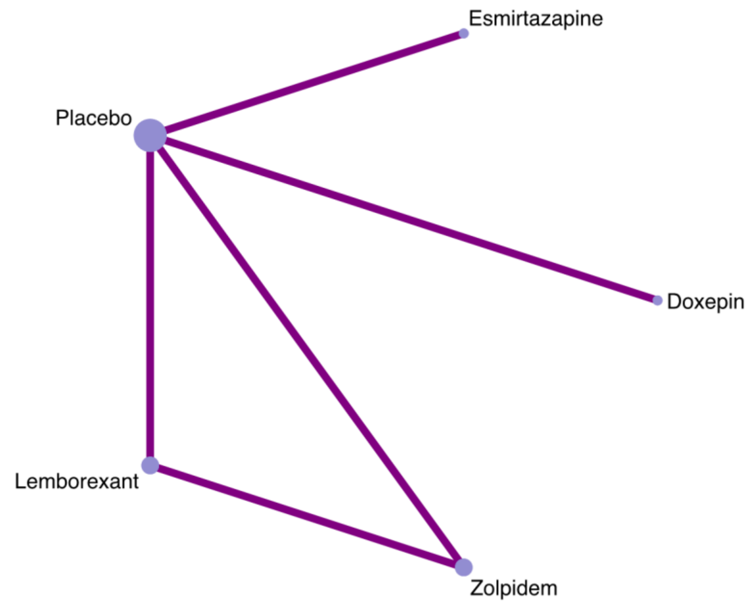

The network evidence plots for increased appetite

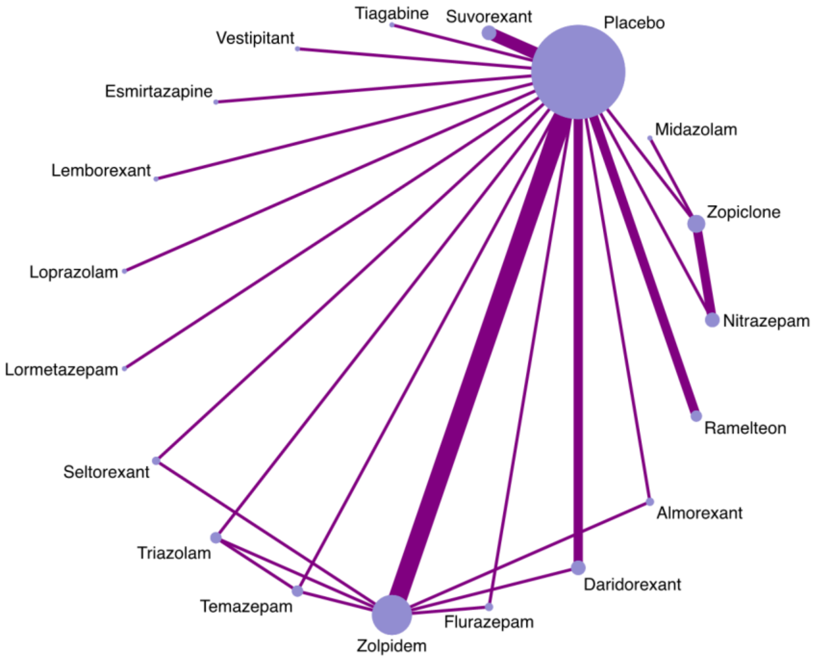

The network evidence plots for fatigue

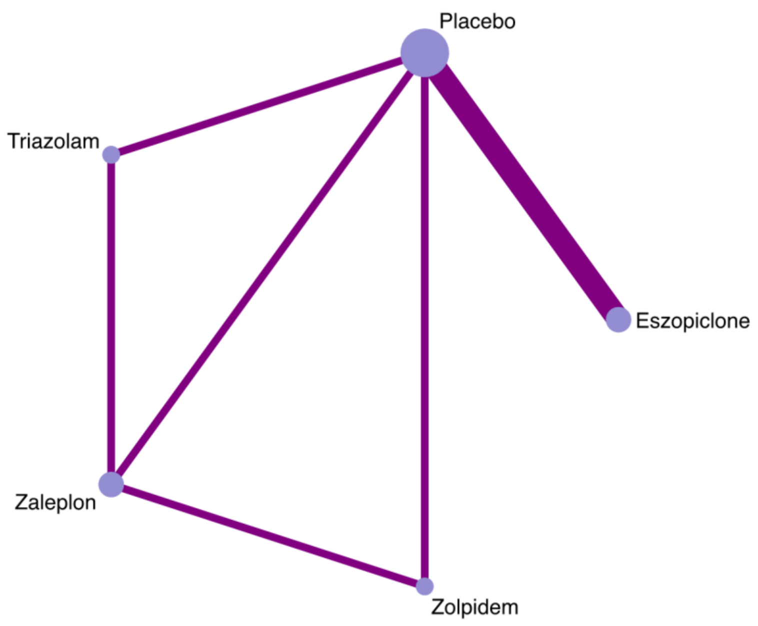

The network evidence plots for pain

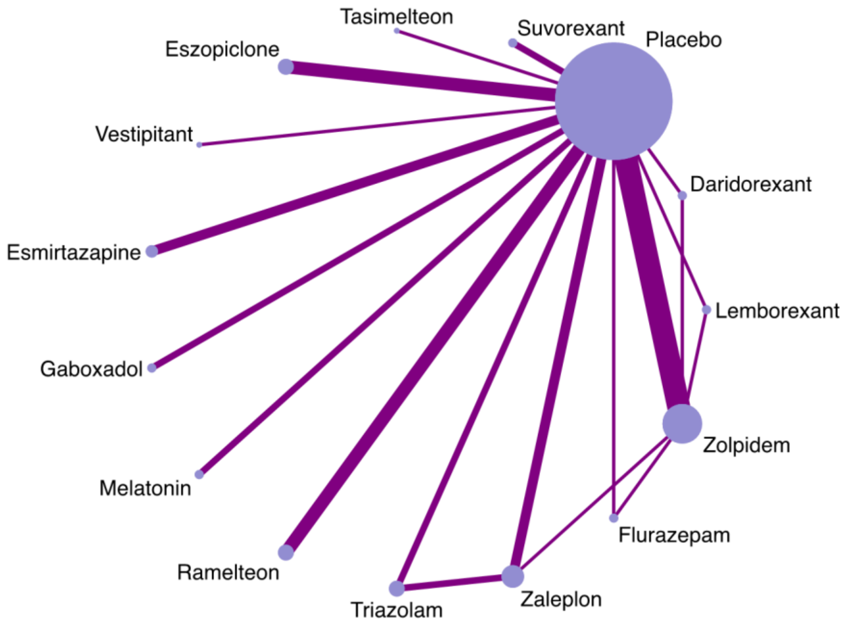

The network evidence plots for nasopharyngitis

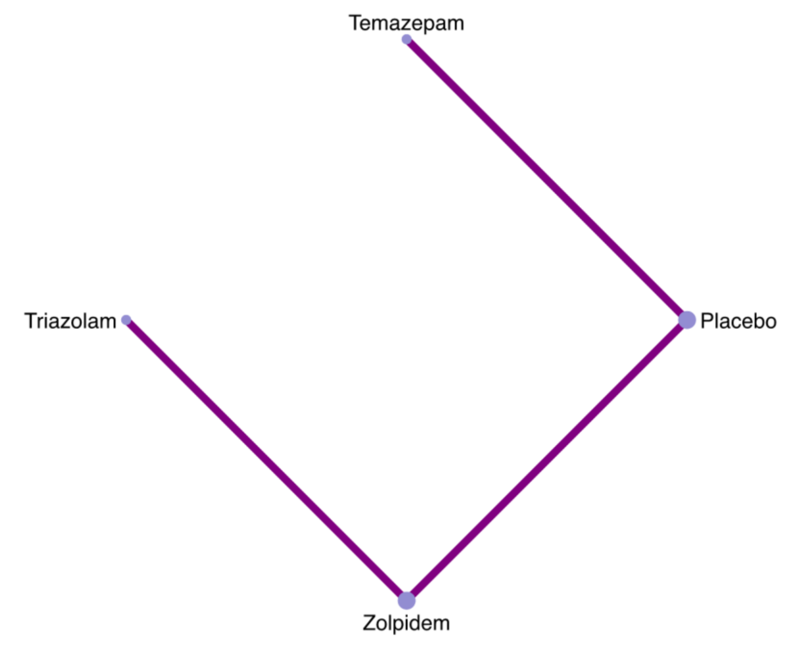

The network evidence plots for respiratory problem

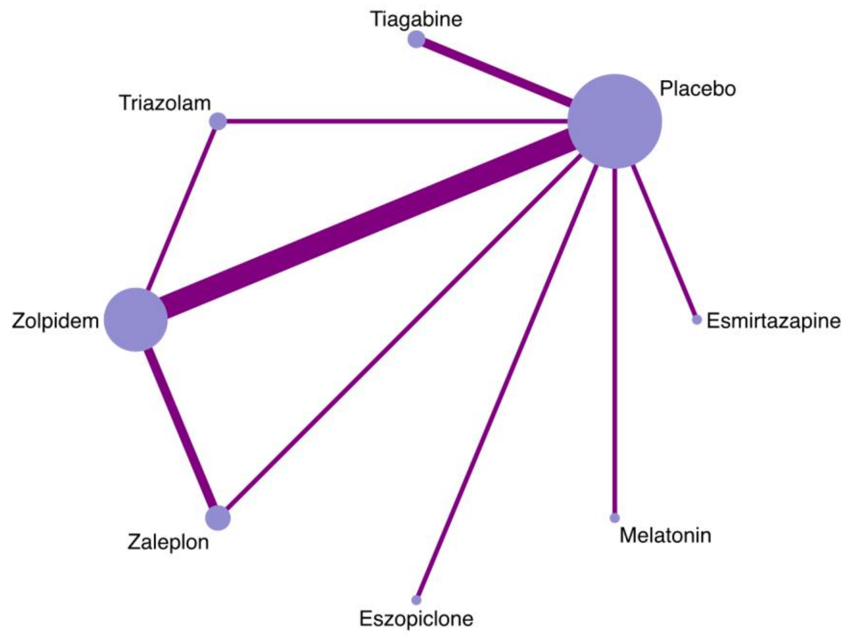

The network evidence plots for anxiety

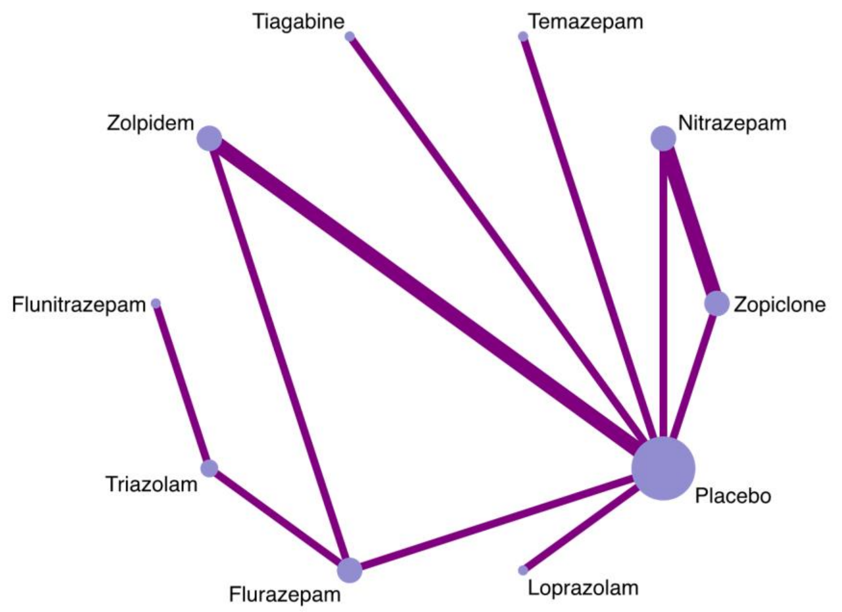

The network evidence plots for confusional state

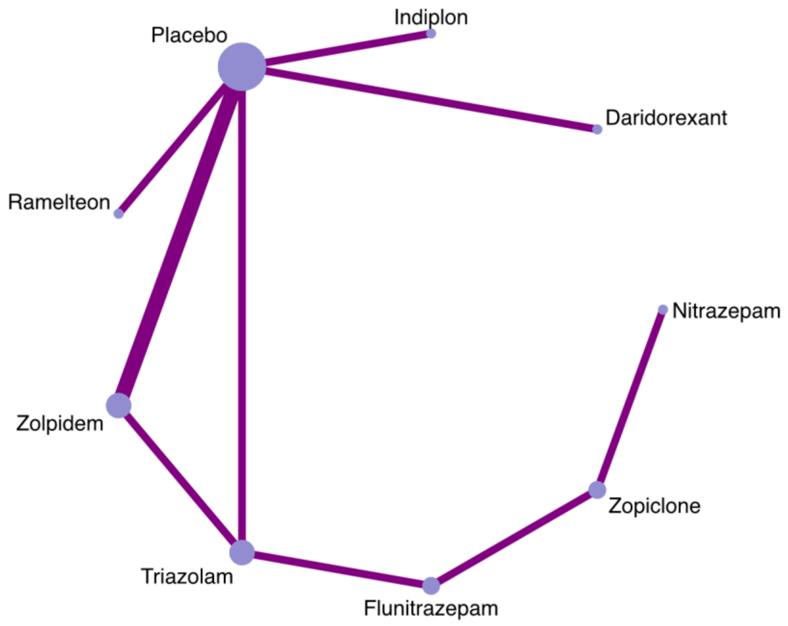

The network evidence plots for depression

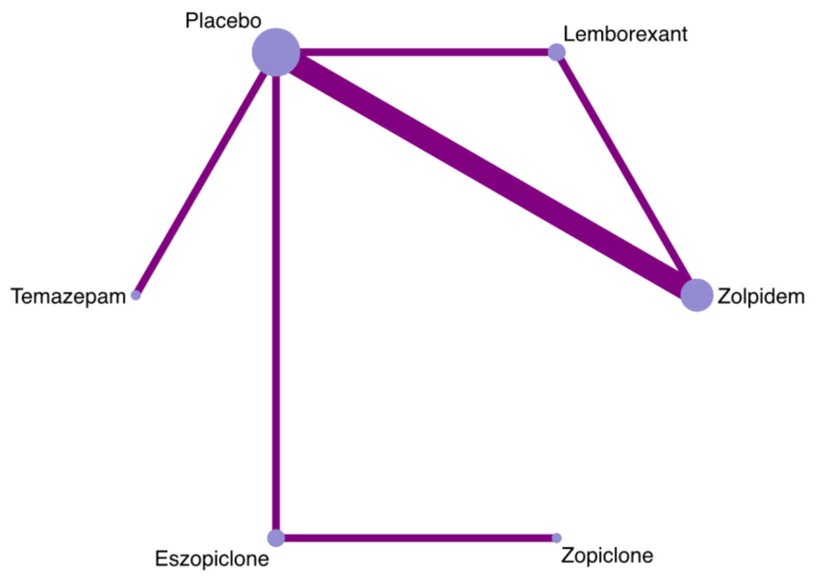

The network evidence plots for emotional lability

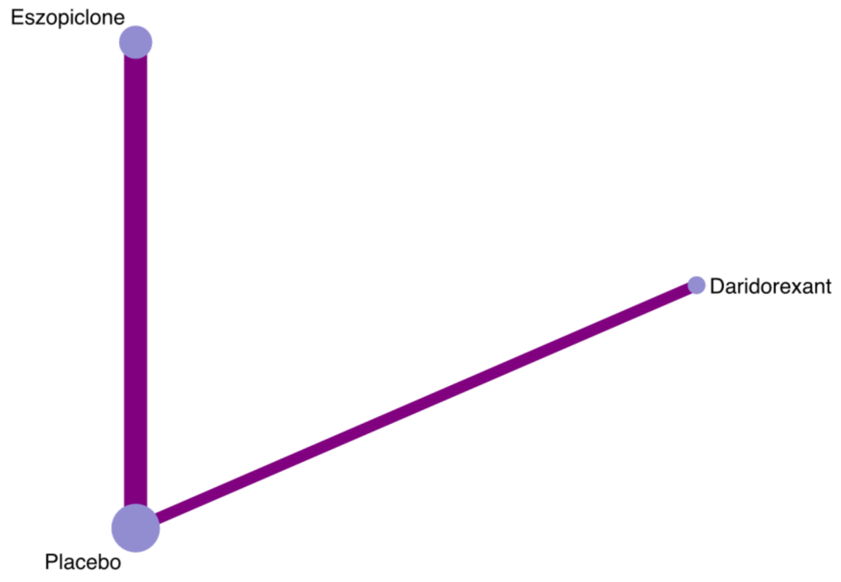

The network evidence plots for accidental injury

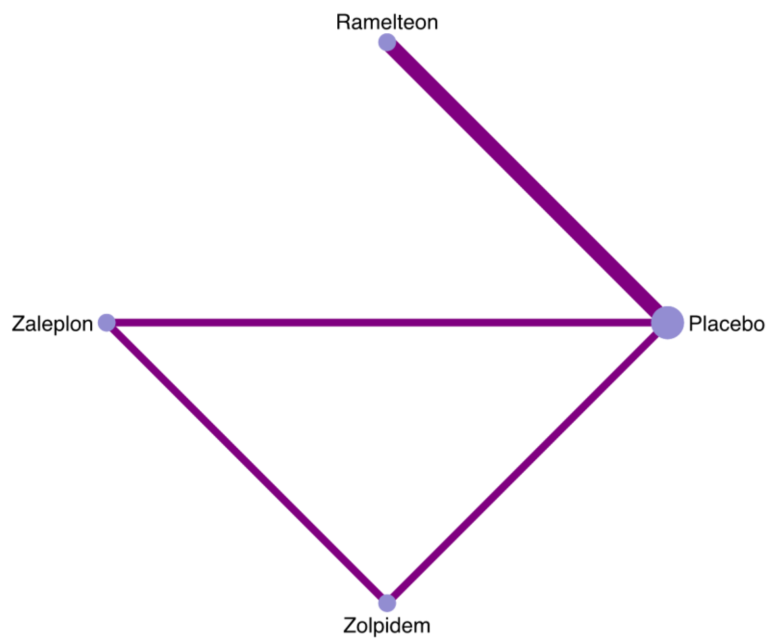

The network evidence plots for eye pain

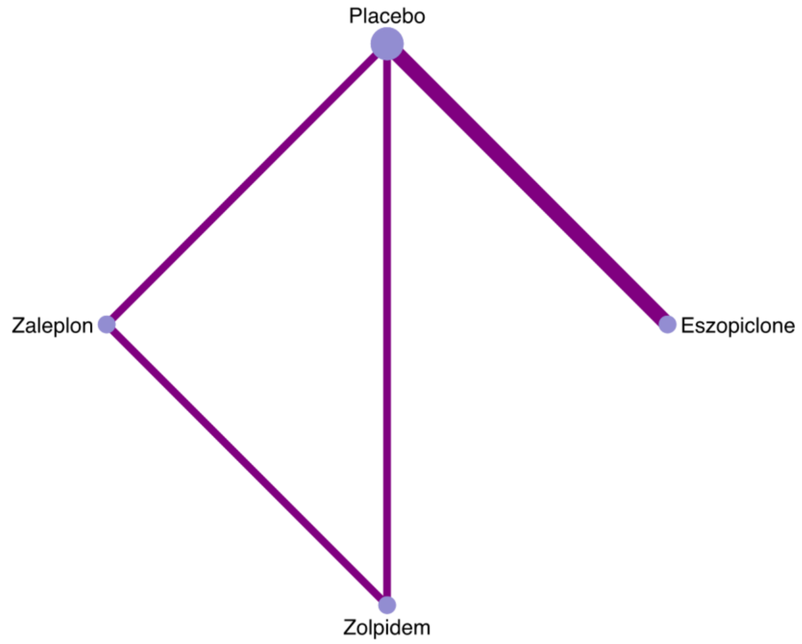

The network evidence plots for infection

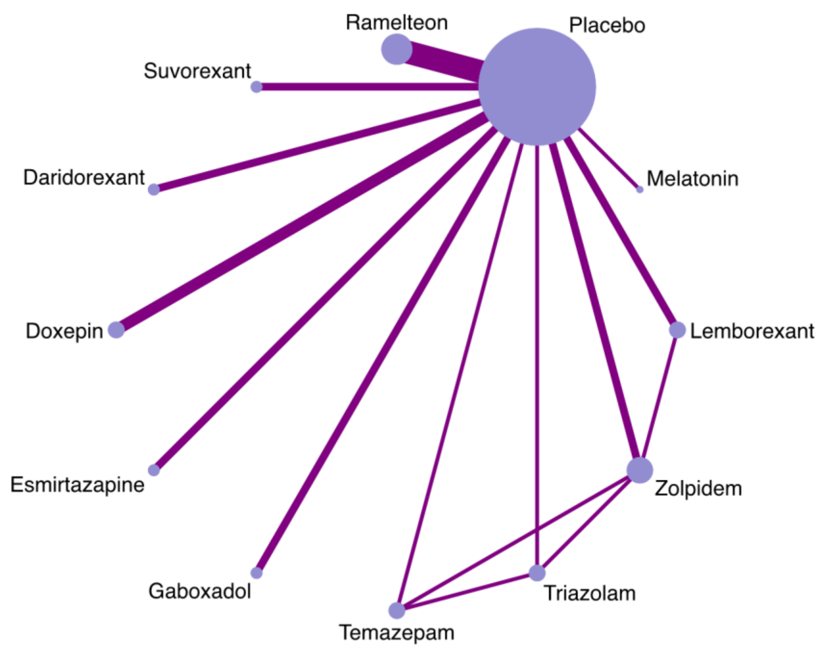

The network evidence plots for upper respiratory tract infection

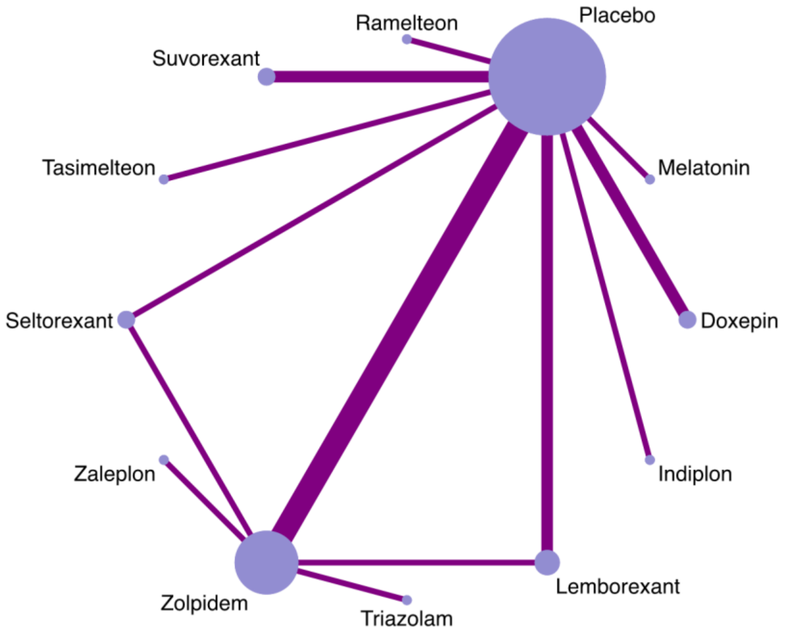

The network evidence plots for urinary tract infection

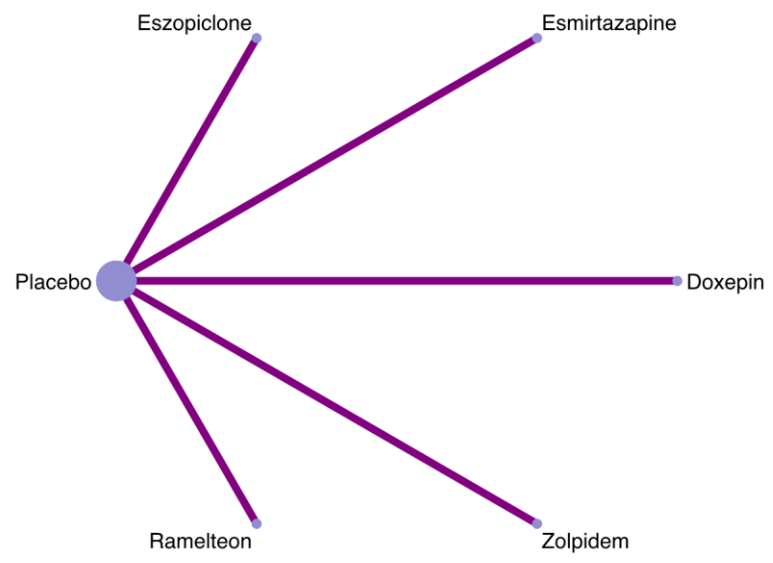

The network evidence plots for sinusitis

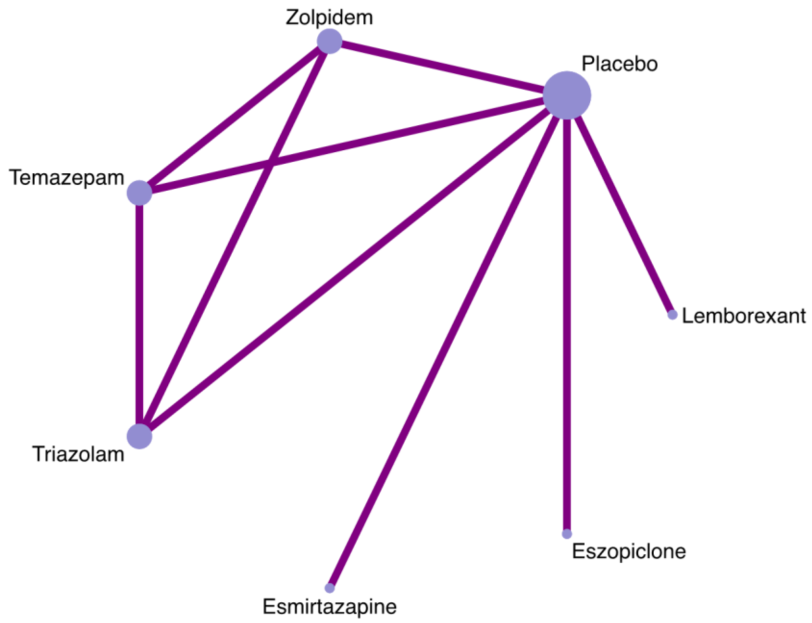

The network evidence plots for arthralgia

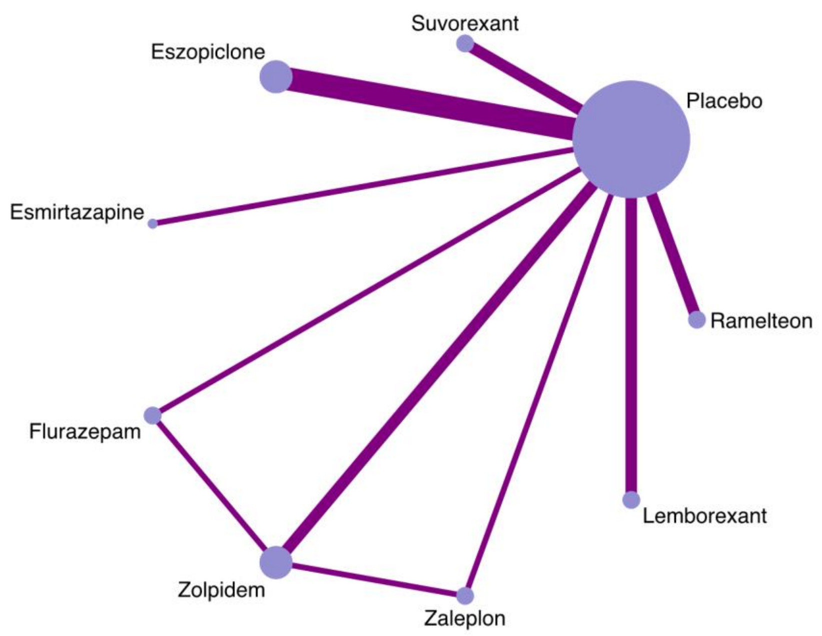

The network evidence plots for back pain

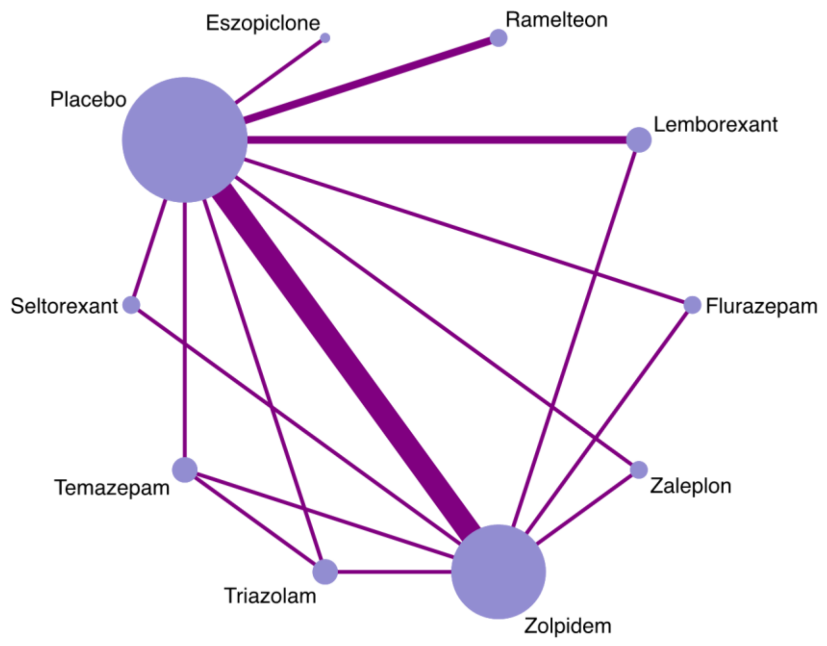

The network evidence plots for myalgia

The network evidence plots for secondary outcomes

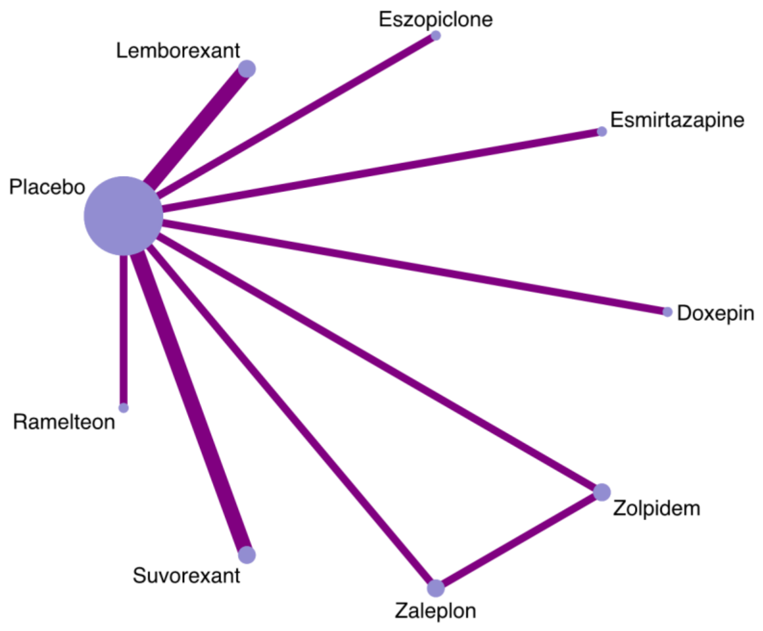

The network evidence plots for abnormal dreams

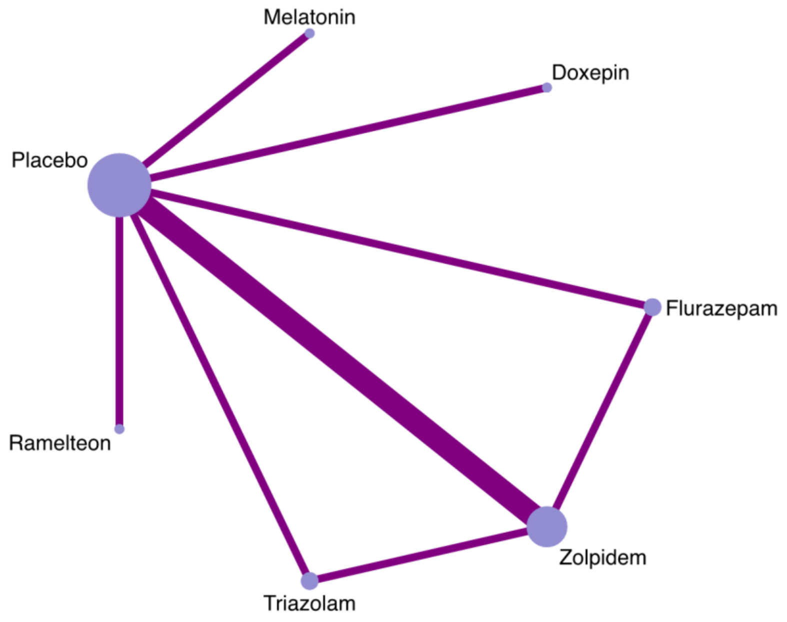

The network evidence plots for abnormal vision

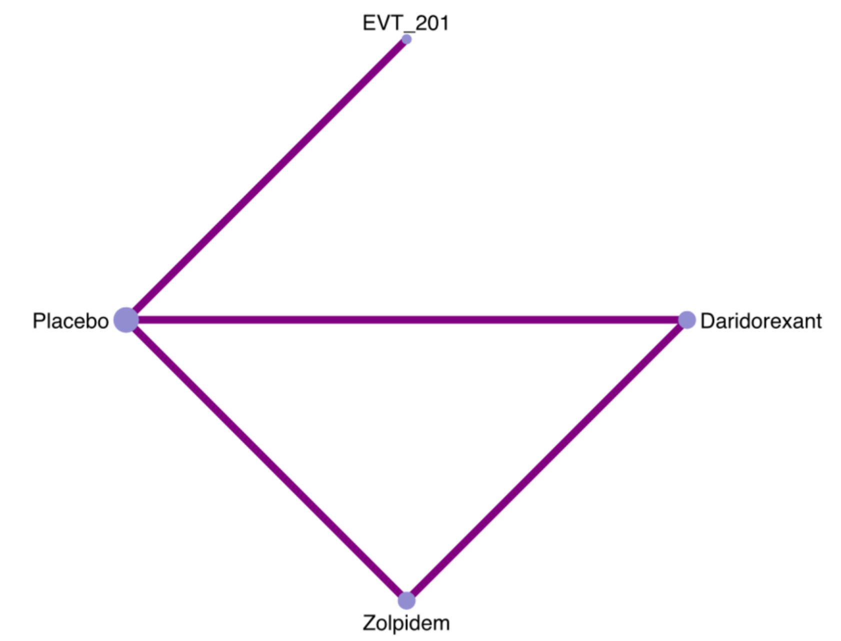

The network evidence plots for gait disturbance

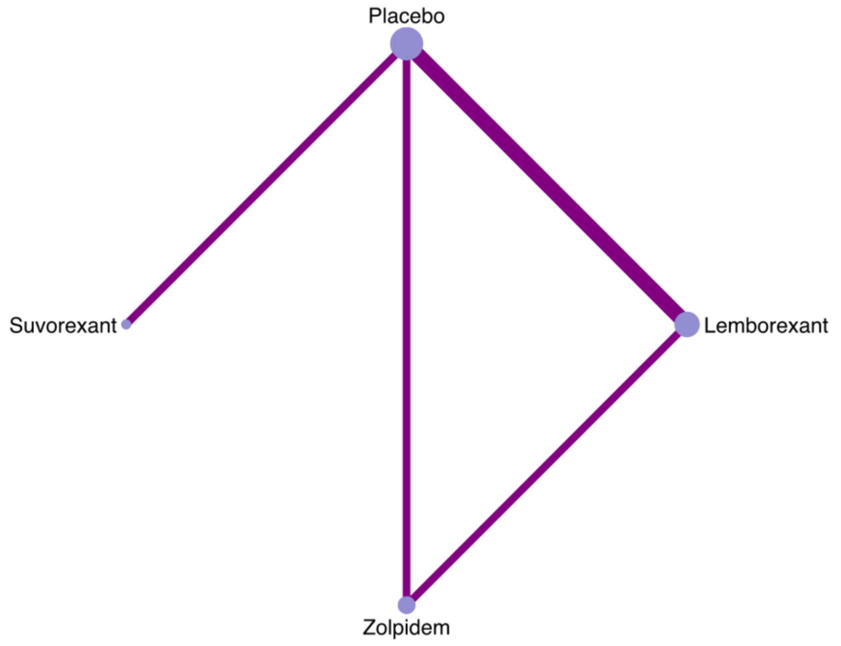

The network evidence plots for hypnagogic hallucinations

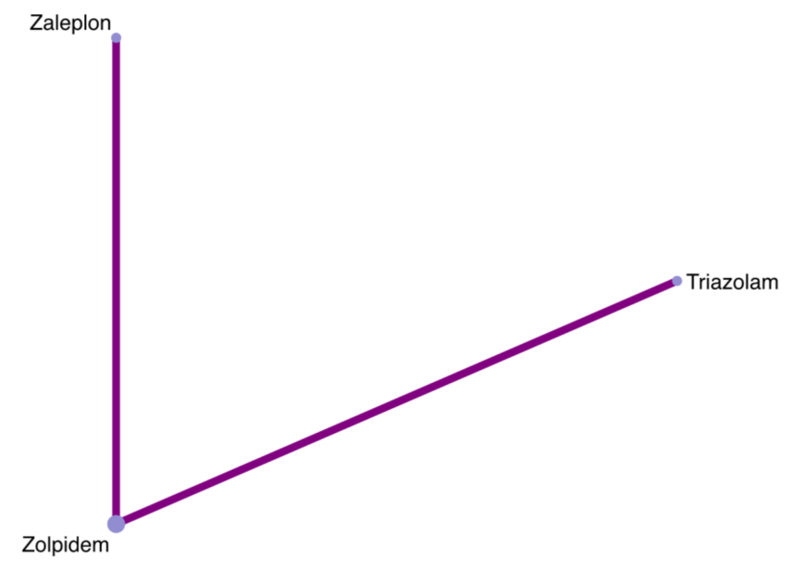

The network evidence plots for tremor

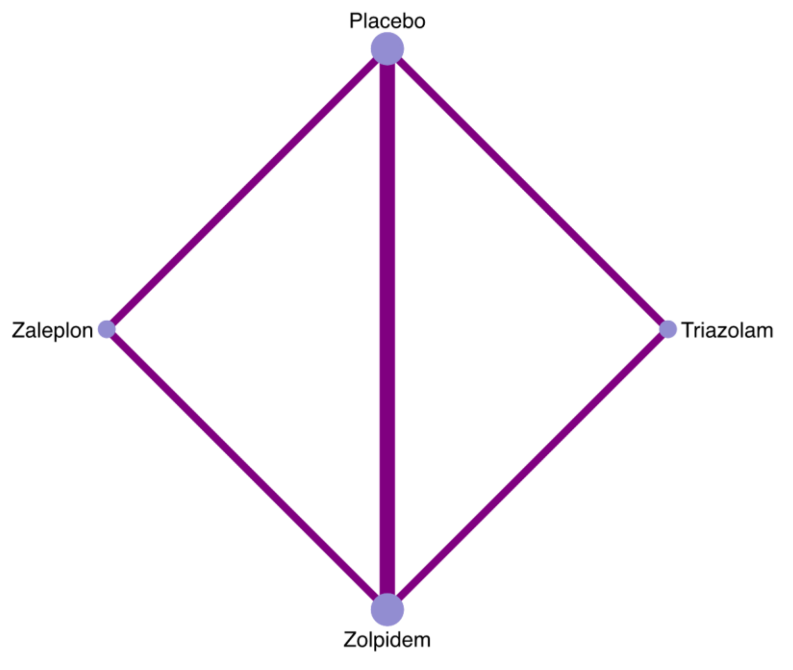

The network evidence plots for paresthesia

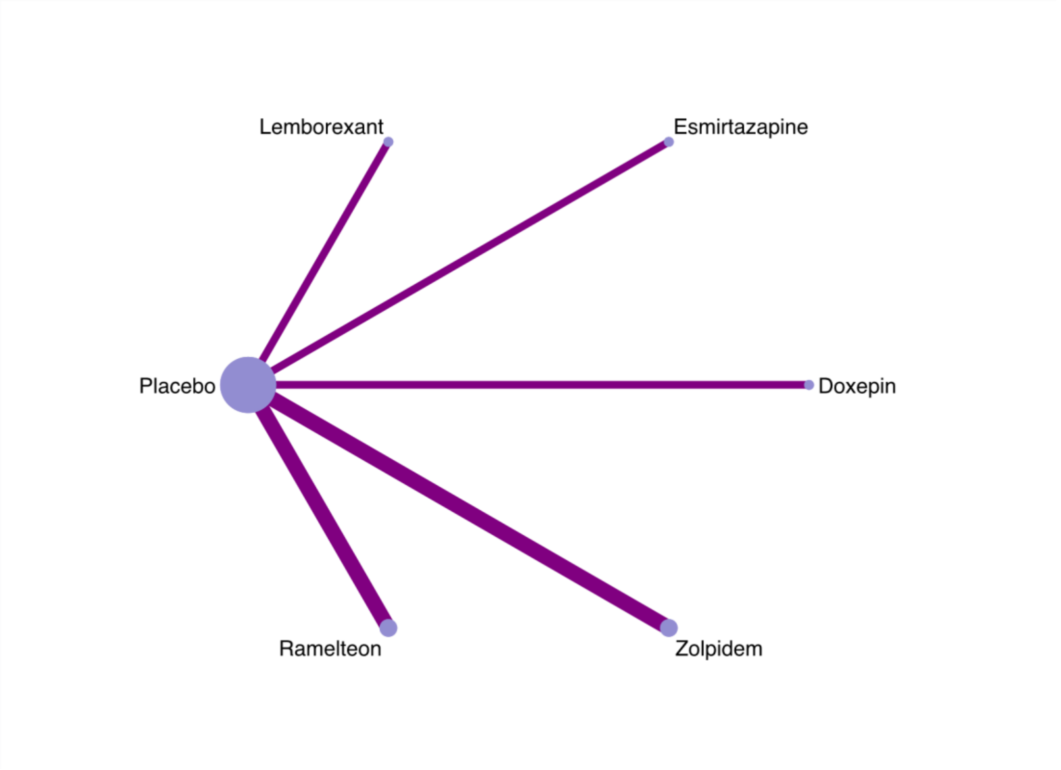

The network evidence plots for gastroenteritis

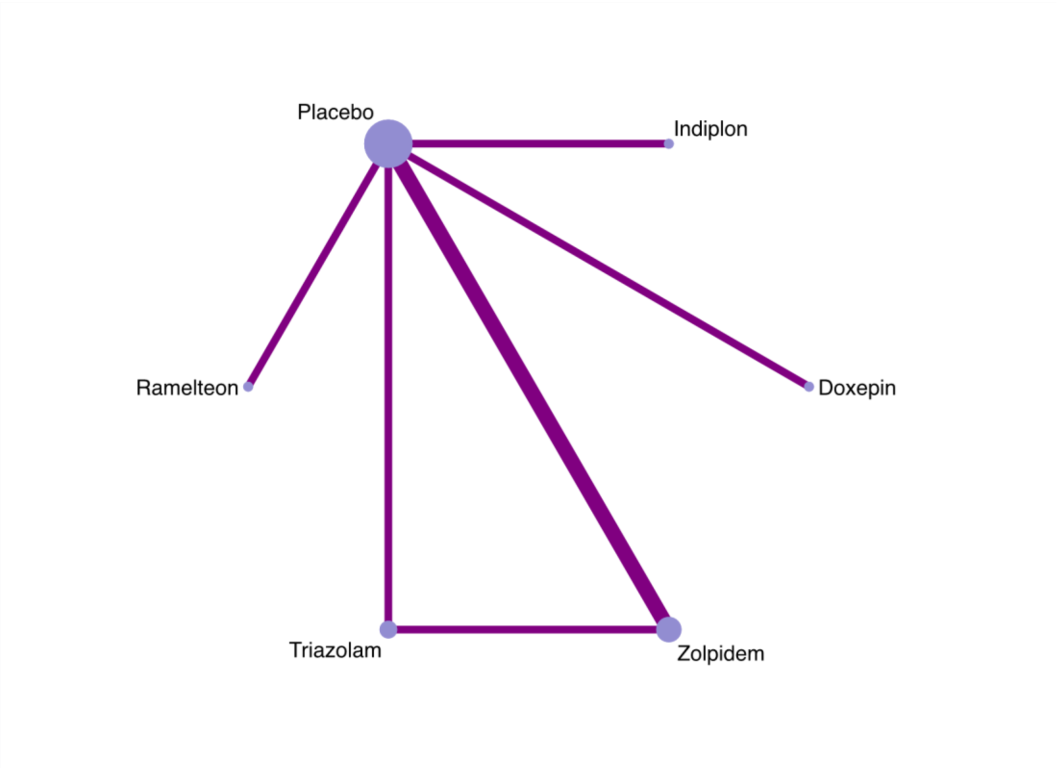

The network evidence plots for decreased appetite

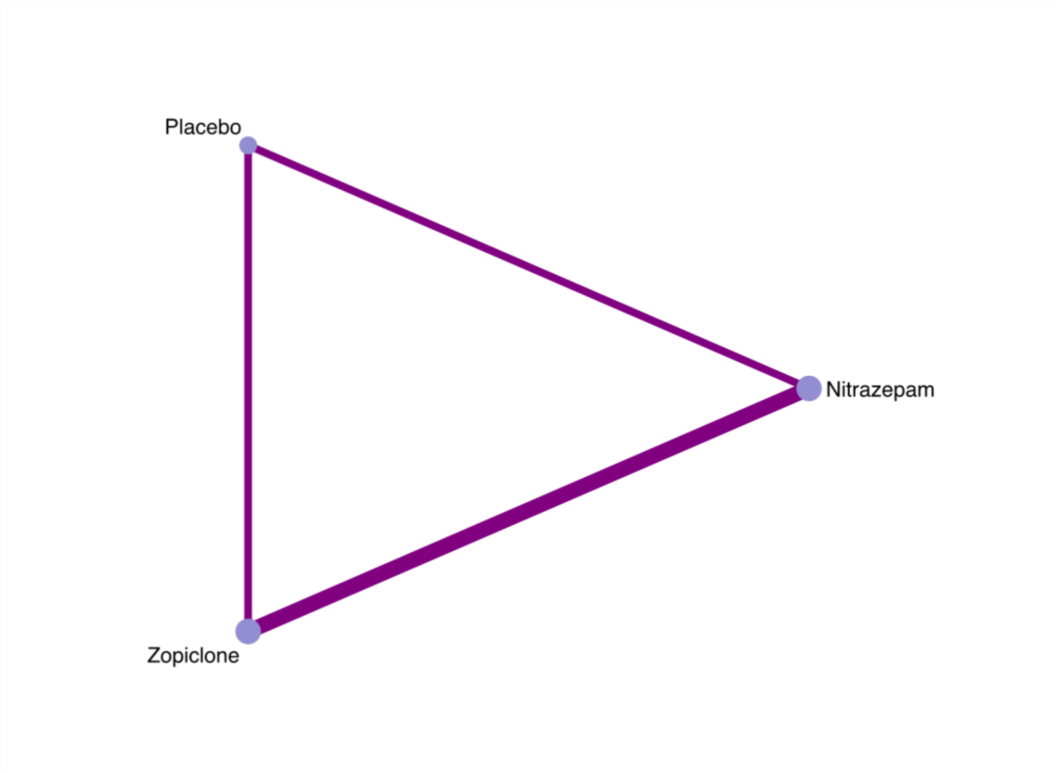

The network evidence plots for pain gastralgia

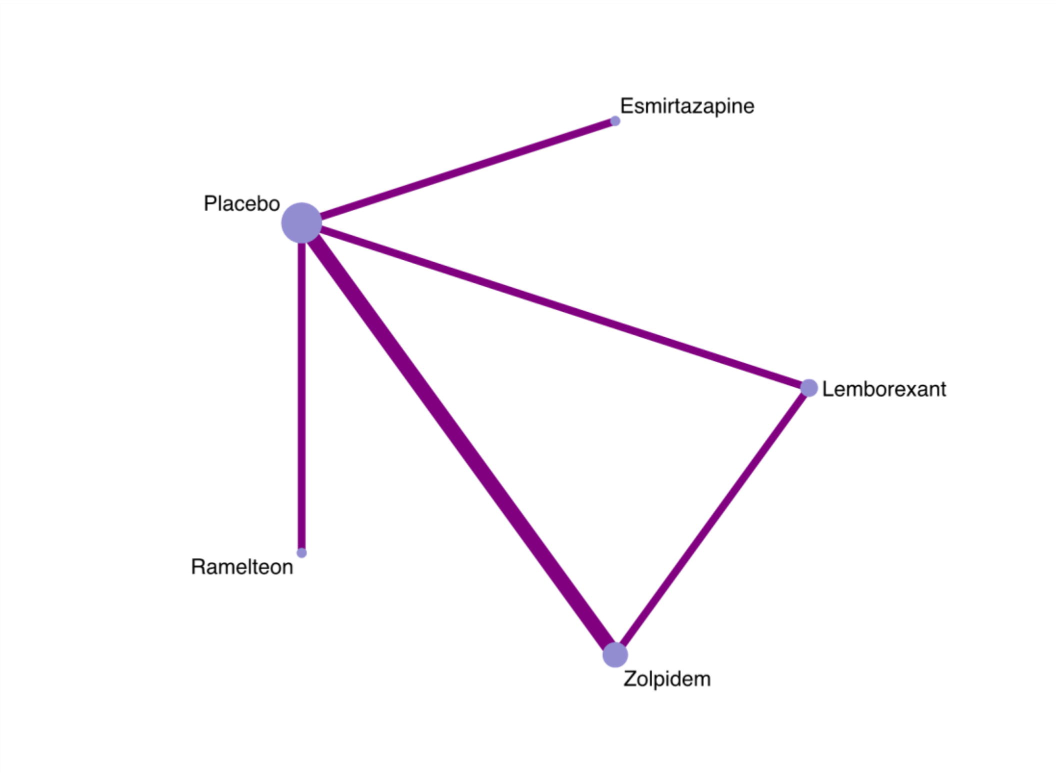

The network evidence plots for insomnia exacerbated

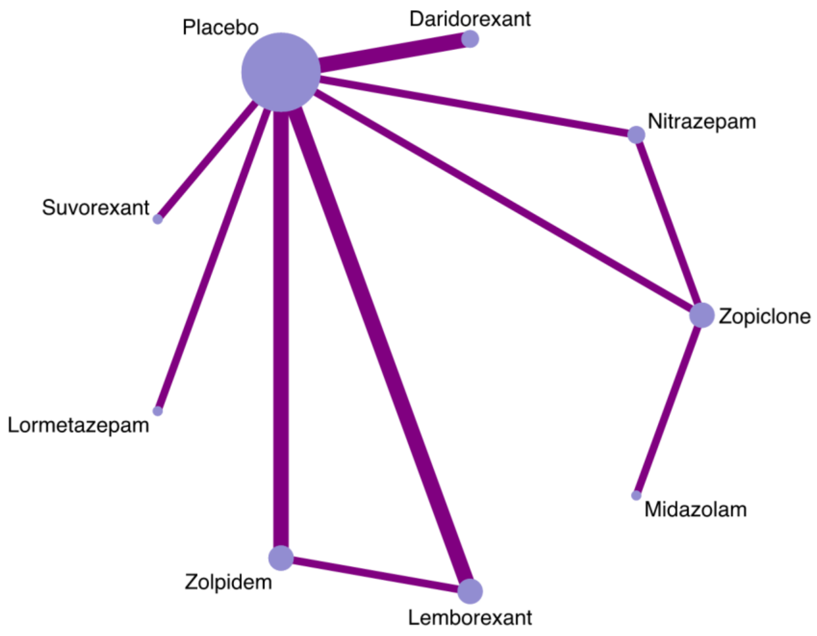

The network evidence plots for sleep paralysis

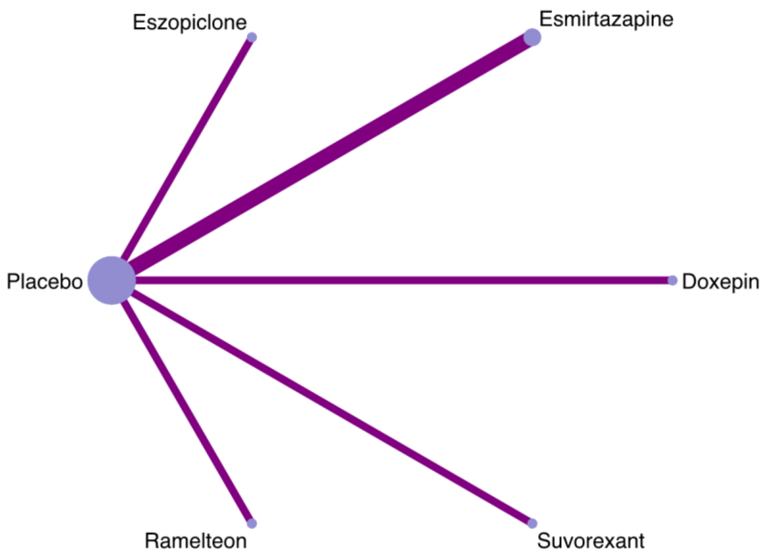

The network evidence plots for oedema

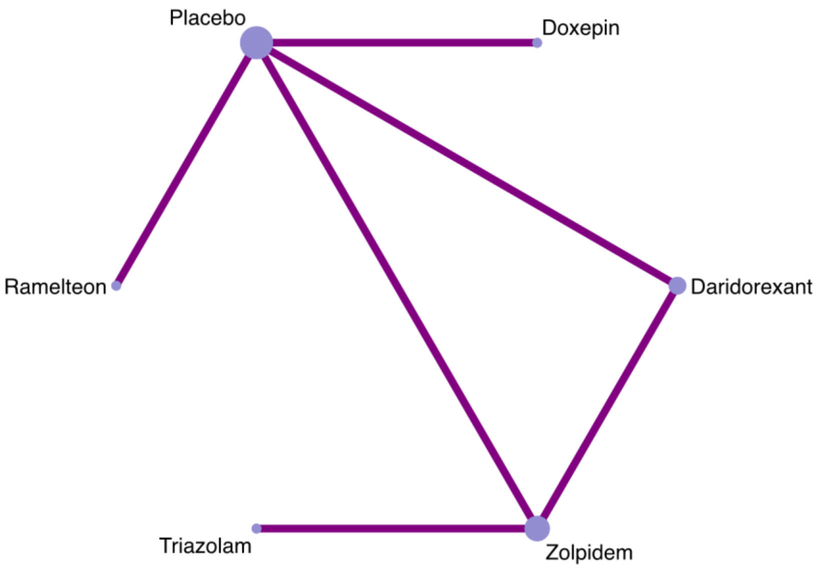

The network evidence plots for pruritis

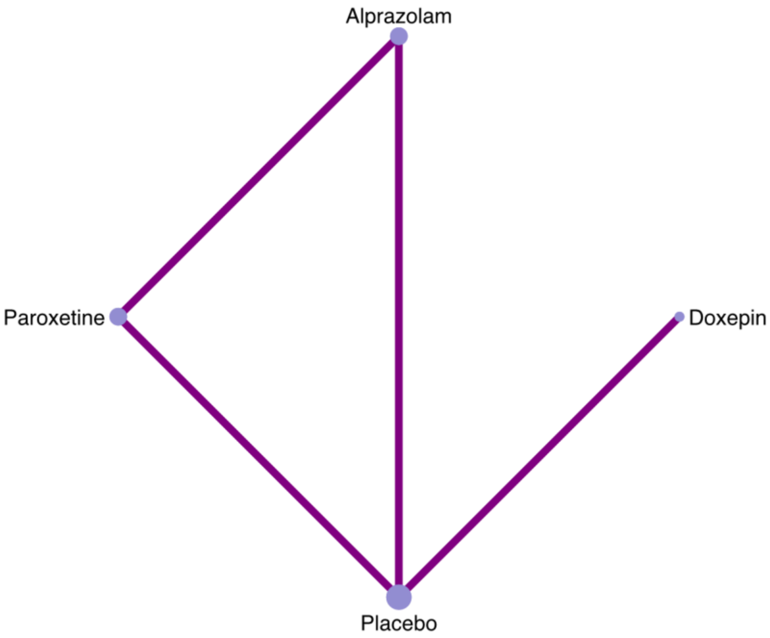

The network evidence plots for sweating

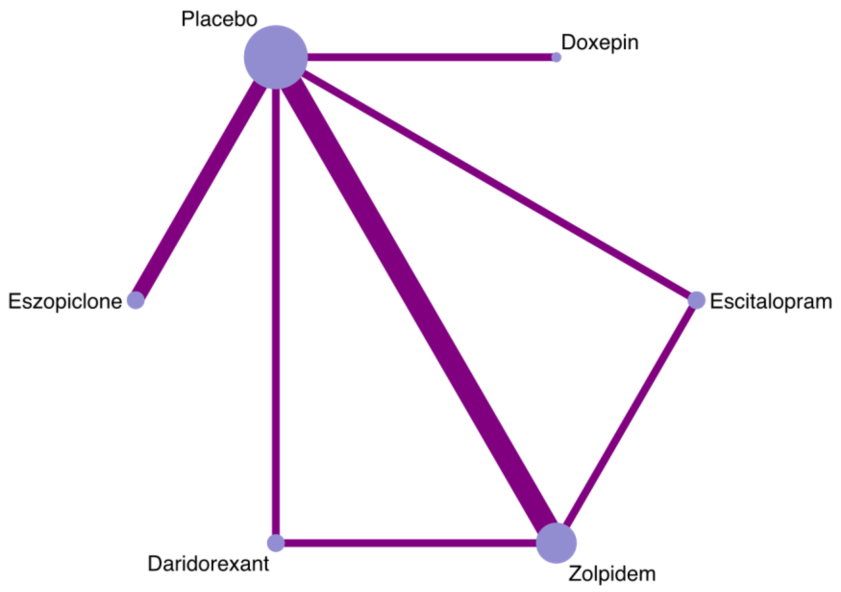

The network evidence plots for skin diseases

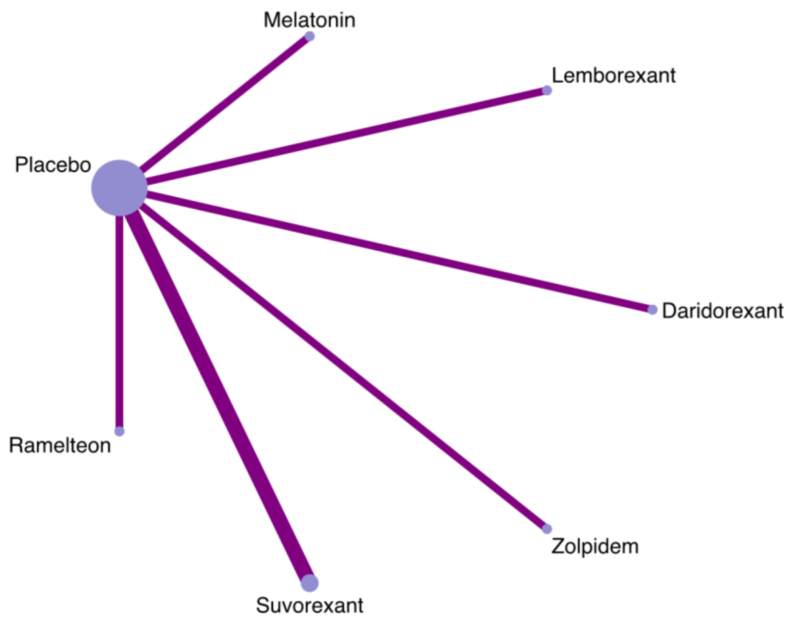

The network evidence plots for influenza

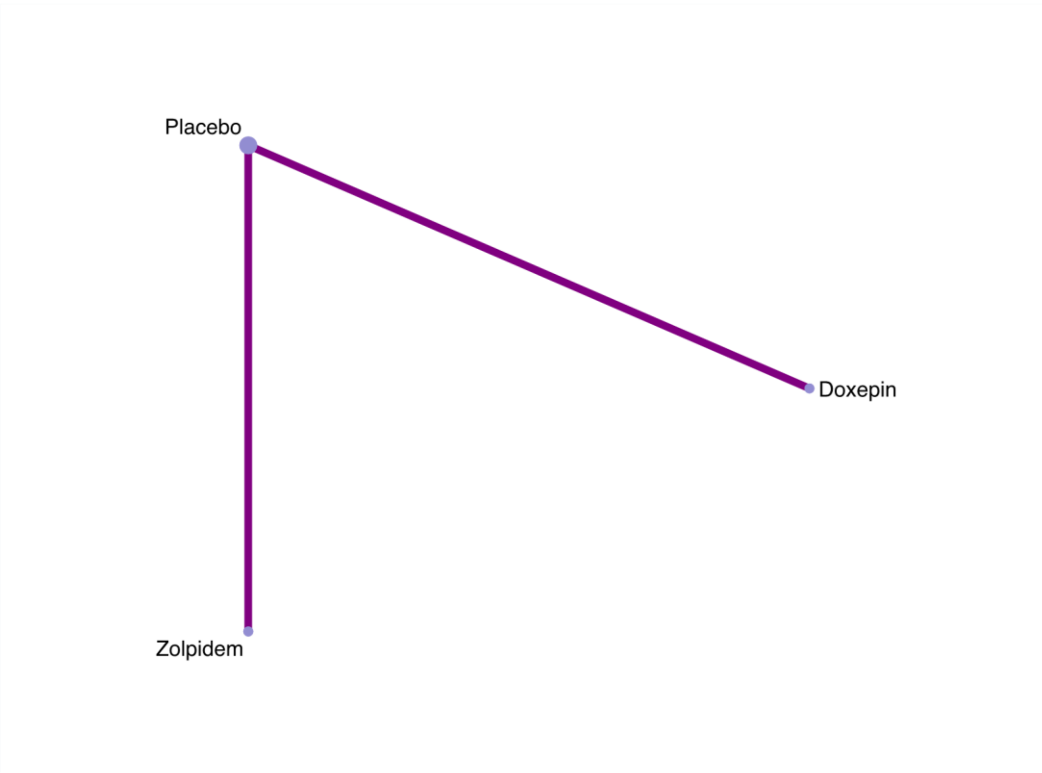

The network evidence plots for common cold

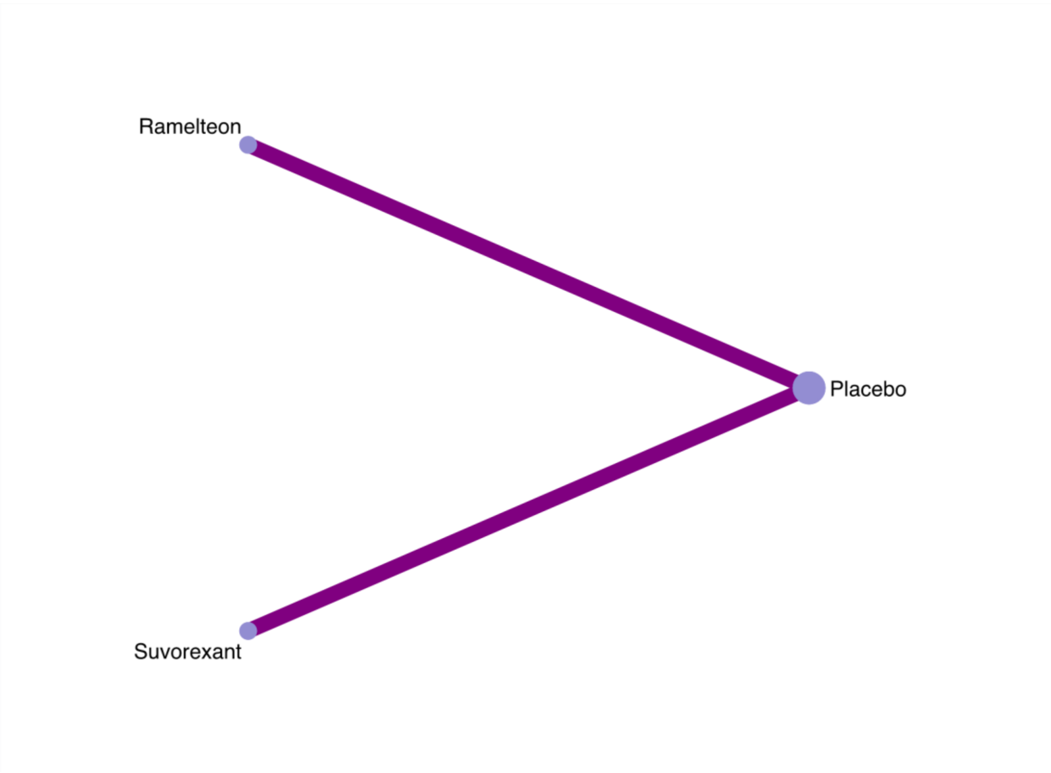

The network evidence plots for malaise

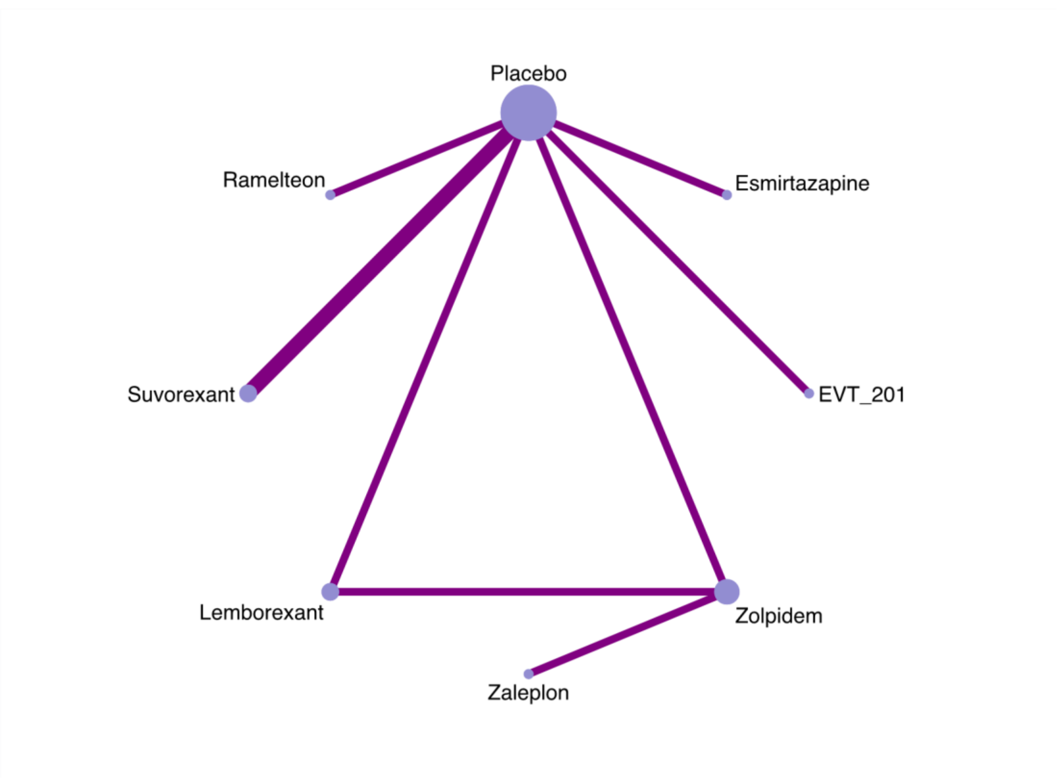

The network evidence plots for cough

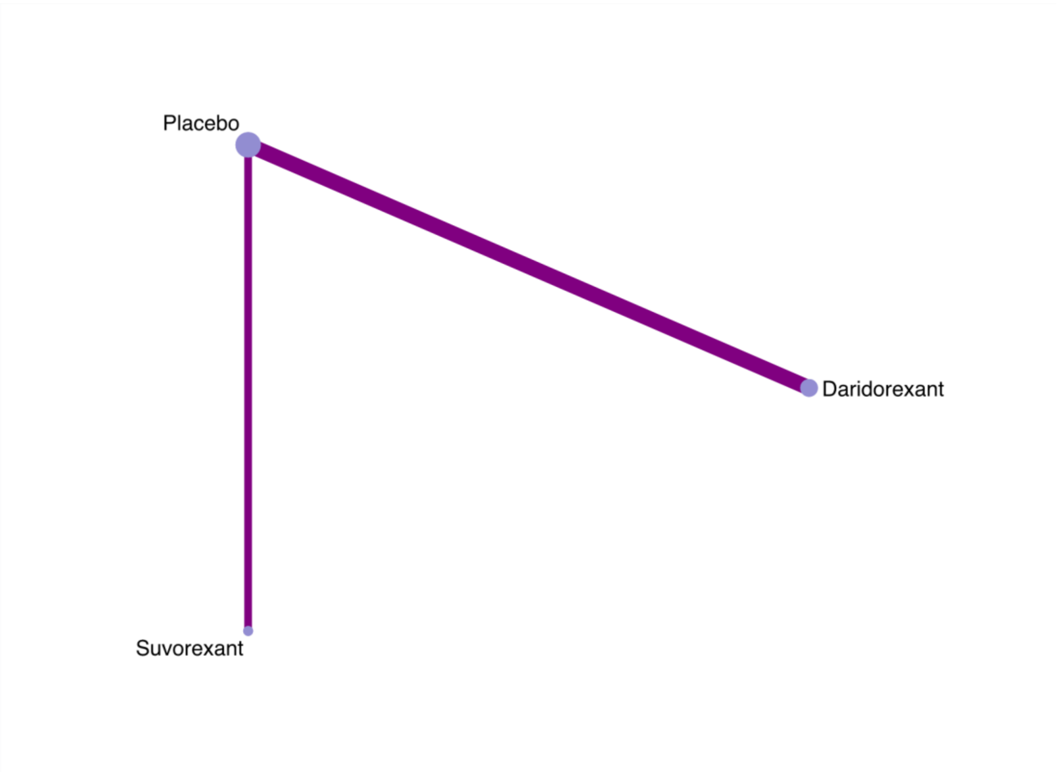

The network evidence plots for suicidal ideation

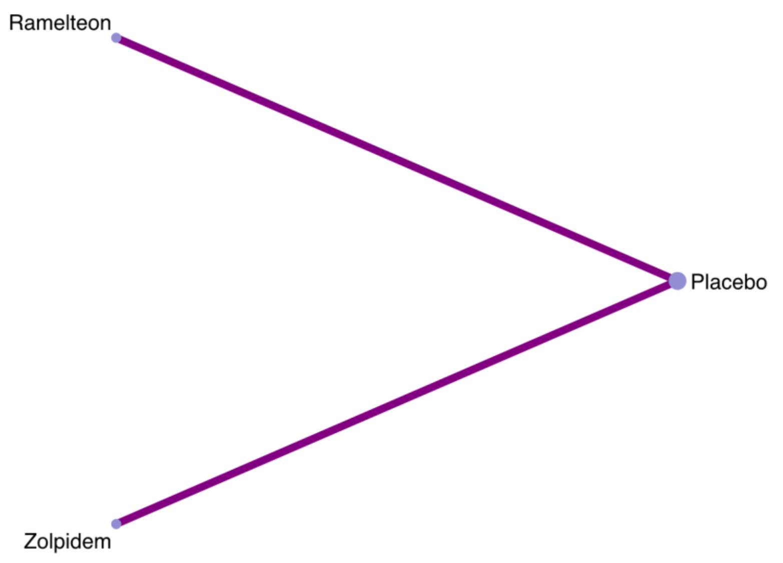

The network evidence plots for irritability

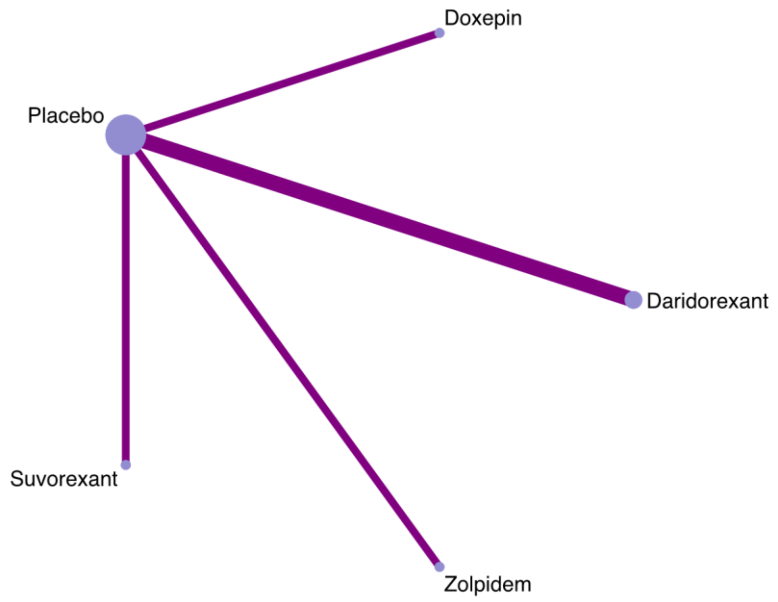

The network evidence plots for falls

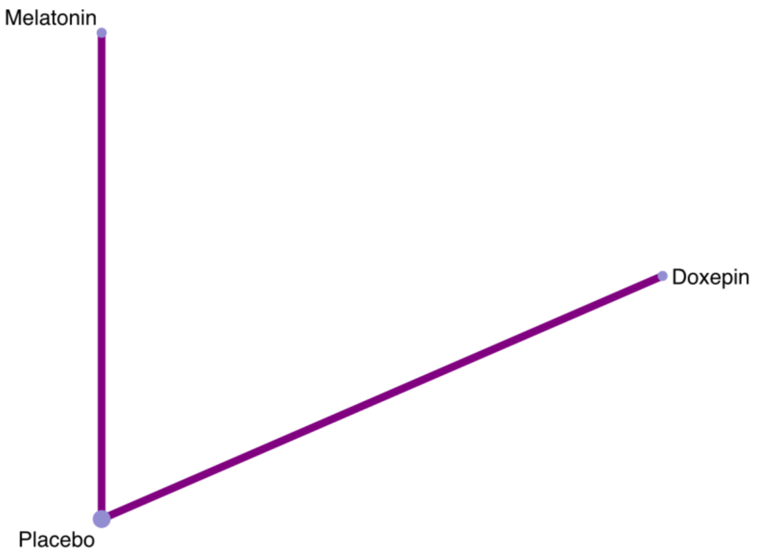

The network evidence plots for laceration

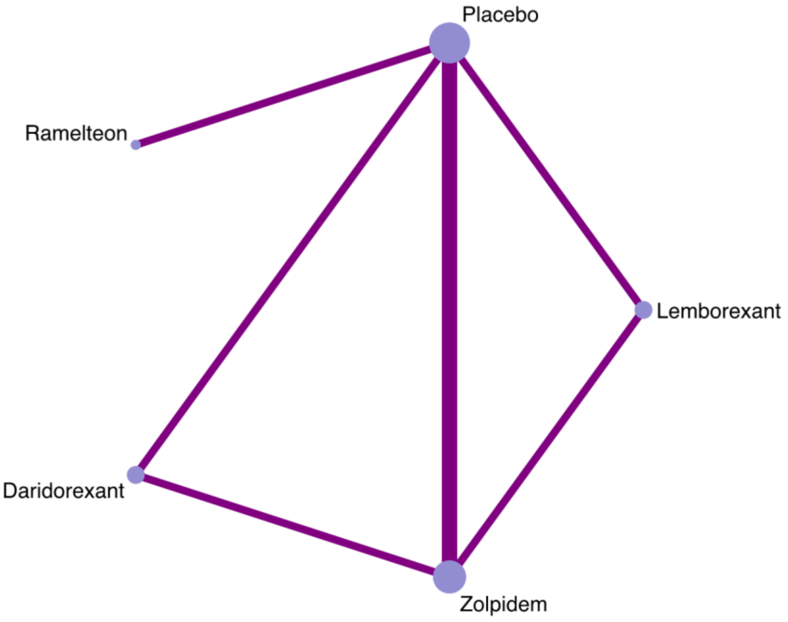

The network evidence plots for alanine aminotransferase increased

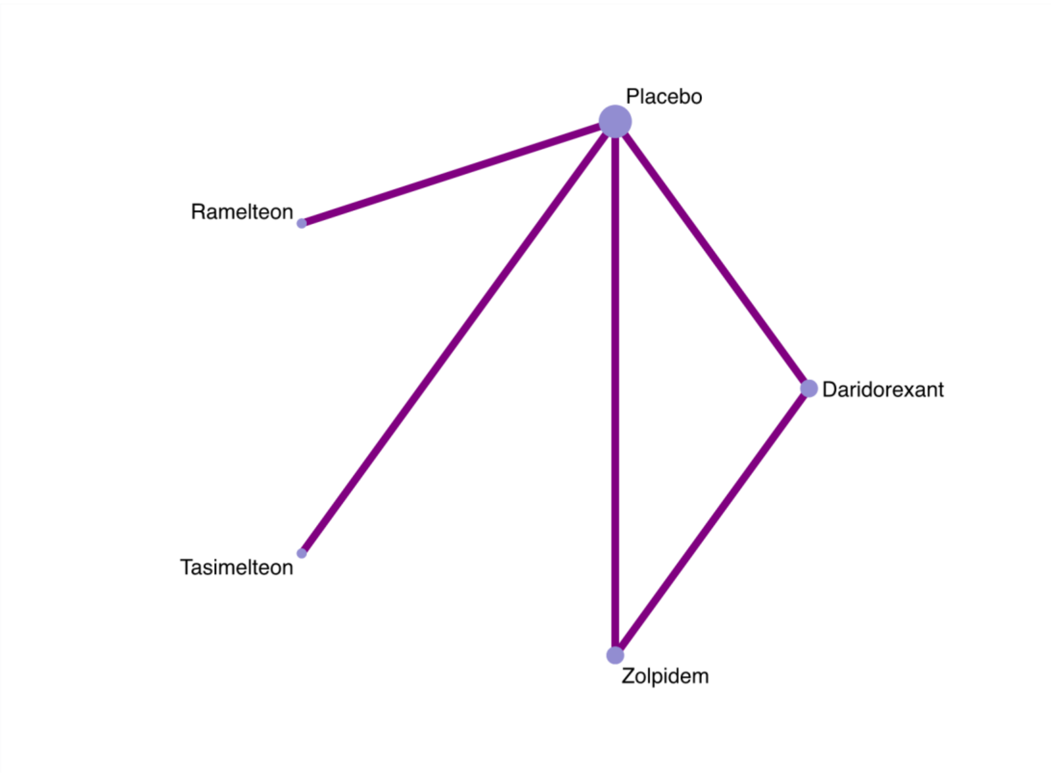

The network evidence plots for blood creatine phosphokinase increased

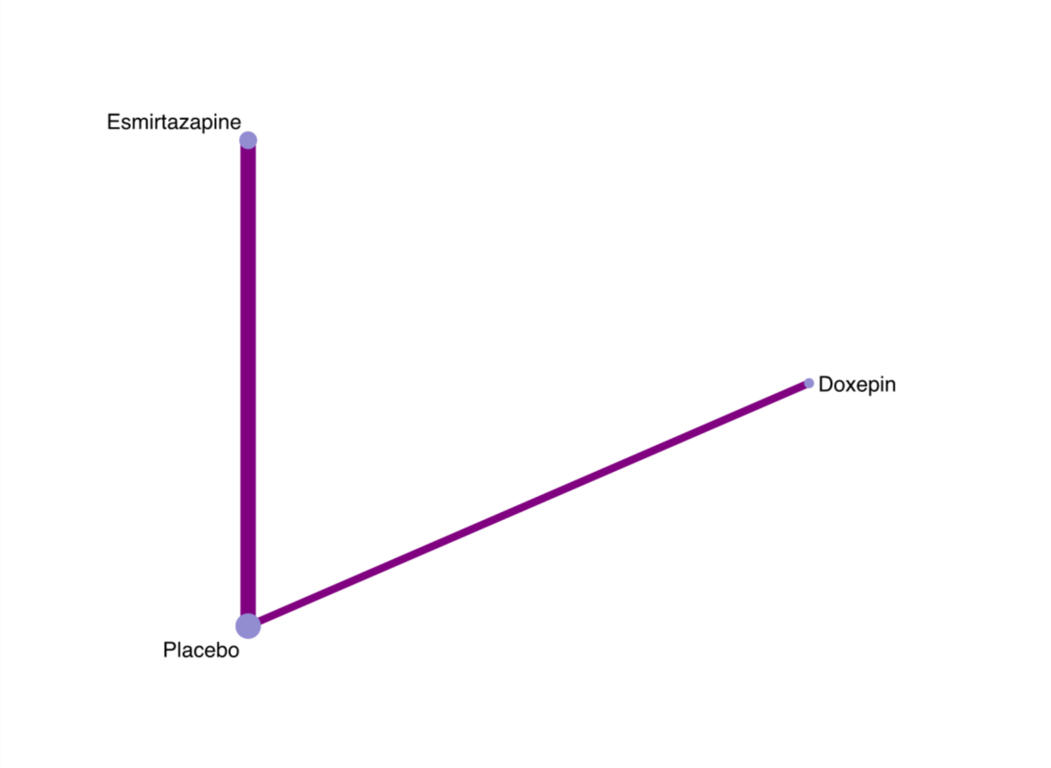

The network evidence plots for weight increased

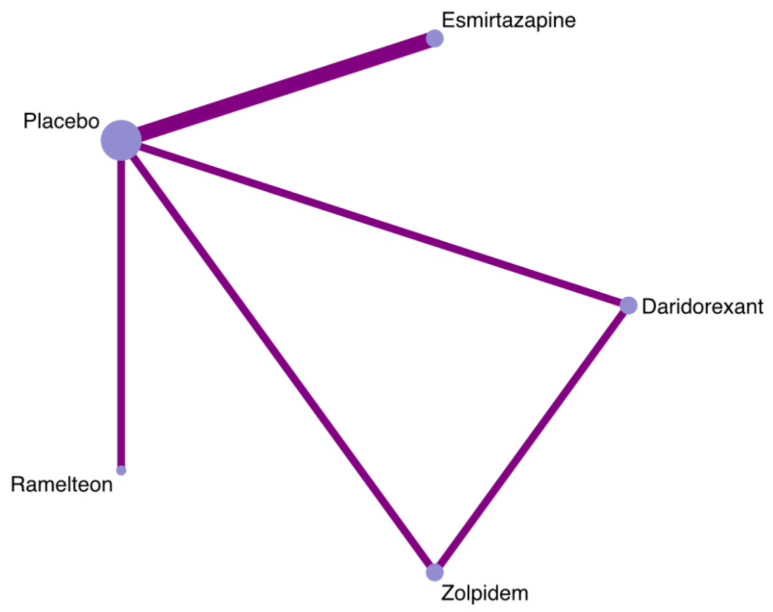

The network evidence plots for γ -Glutamyl transferase increased

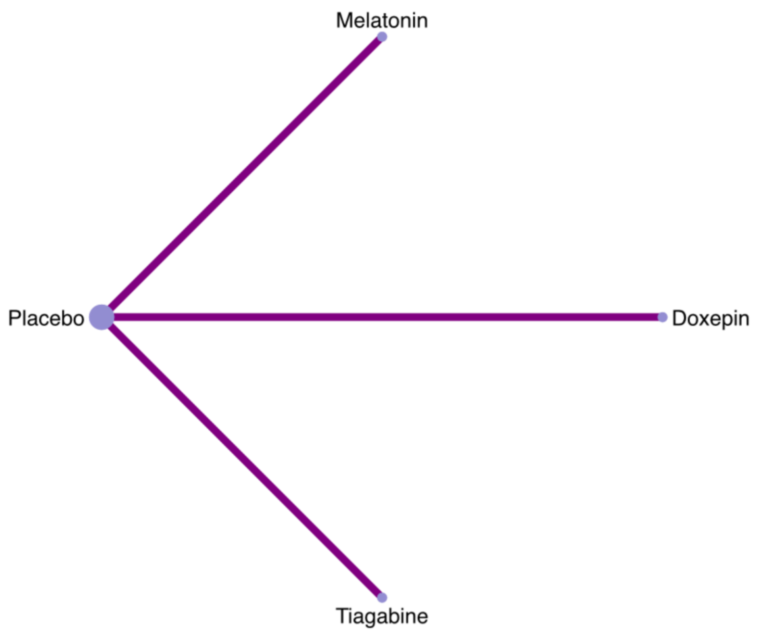

The network evidence plots for hyperglycaemia

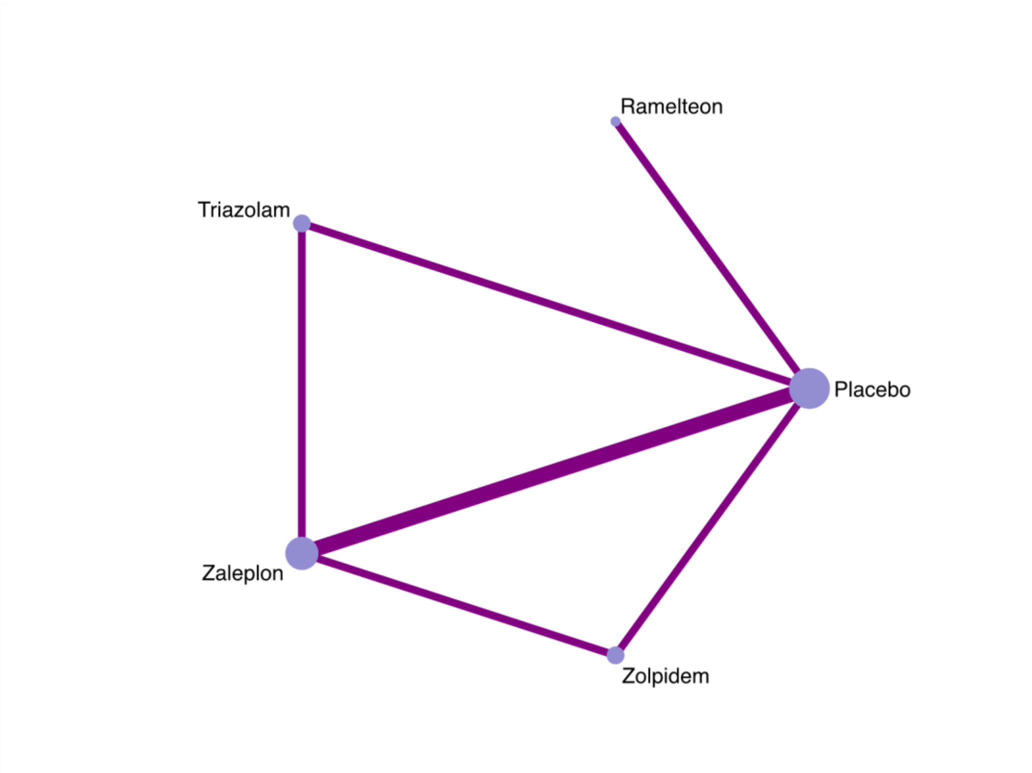

The network evidence plots for dysmenorrhea

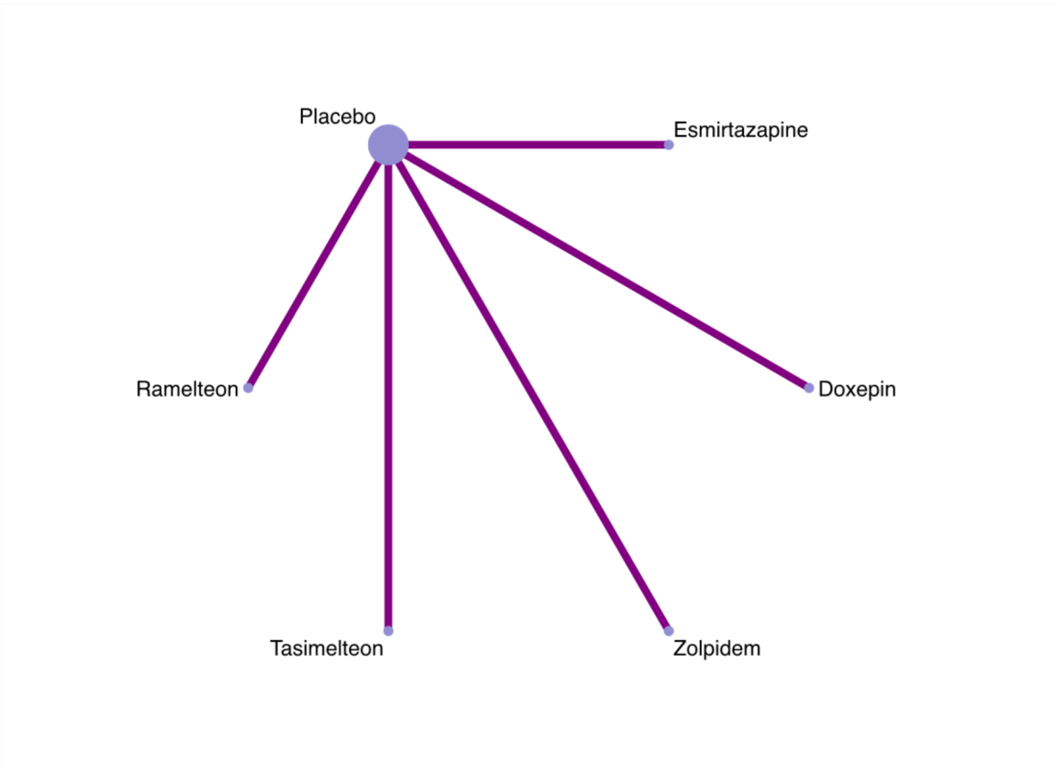

The network evidence plots for hypertension

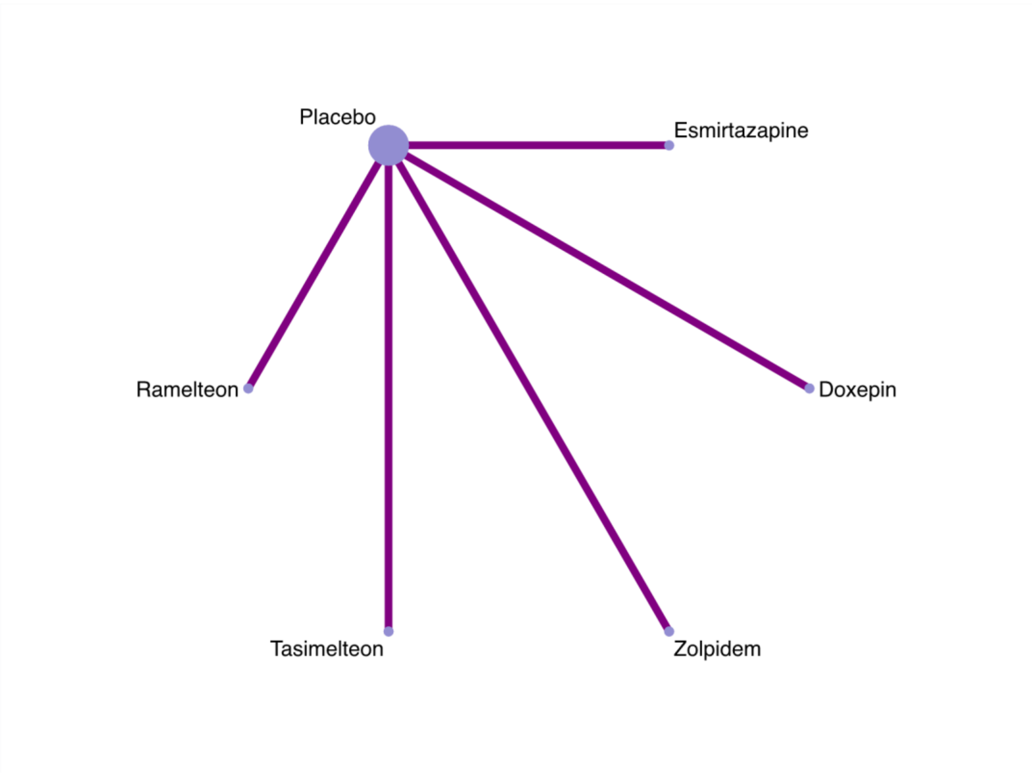

The network evidence plots for tachycardia

Appendix 10 Results of pairwise meta-analysis

Results of pairwise meta-analysis for primary outcomes

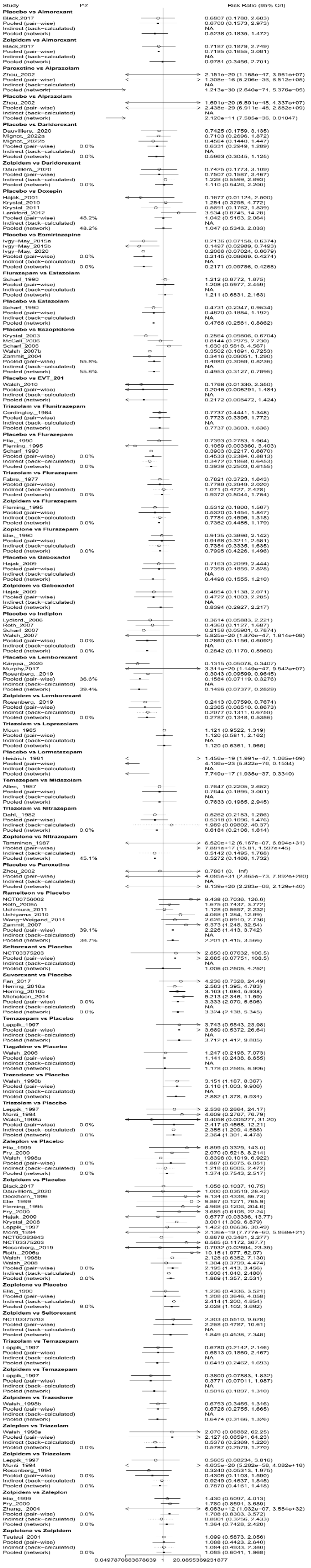

Results of pairwise meta-analysis for somnolence

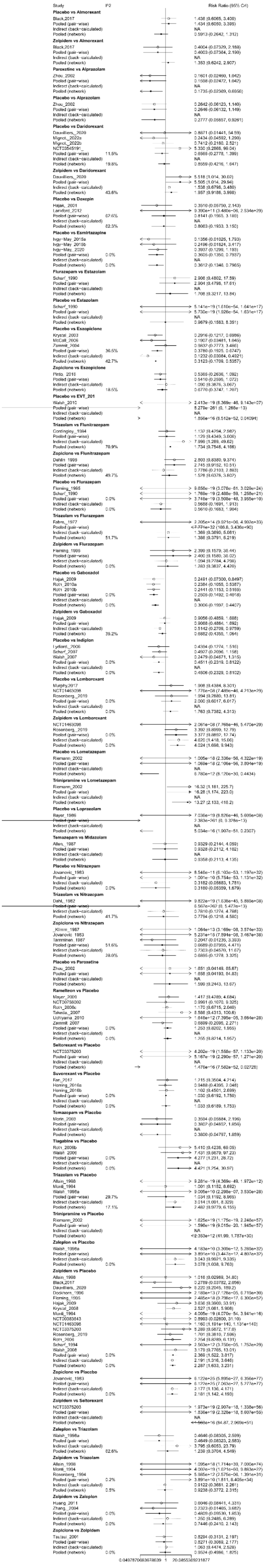

Results of pairwise meta-analysis for dizziness

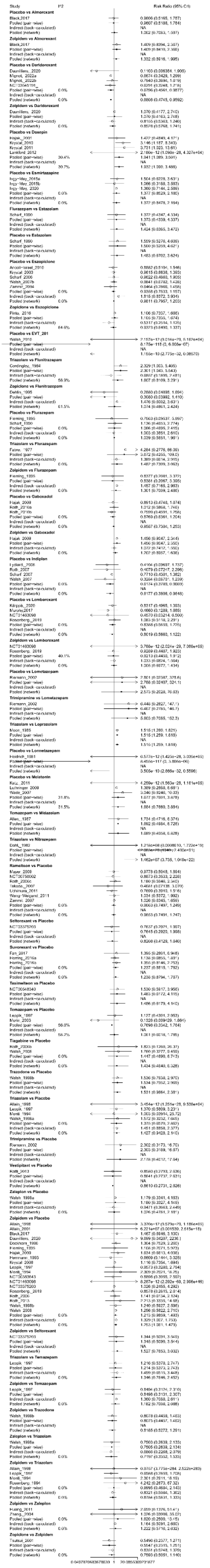

Results of pairwise meta-analysis for headache

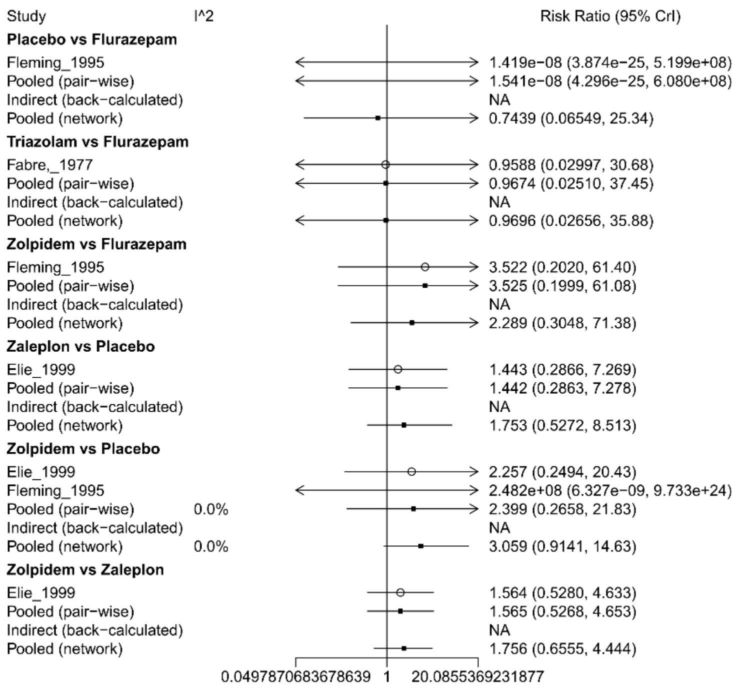

Results of pairwise meta-analysis for amnesia

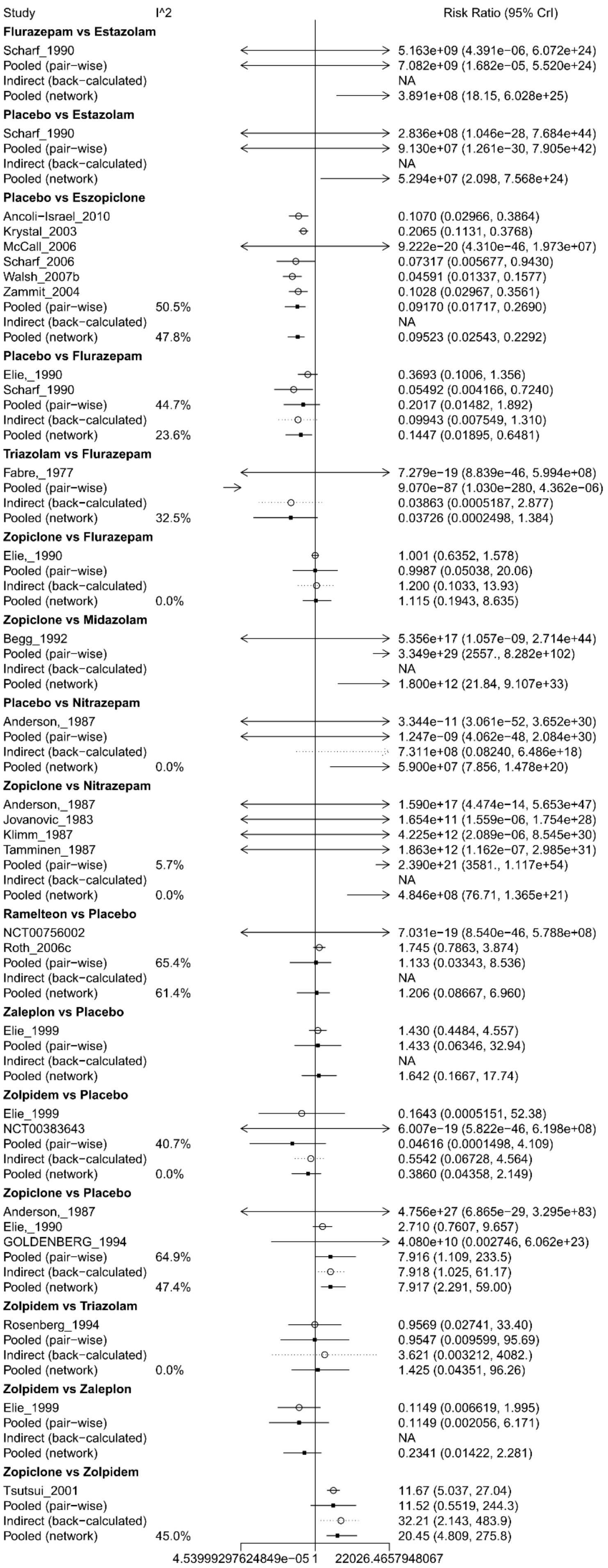

Results of pairwise meta-analysis for dysgeusia

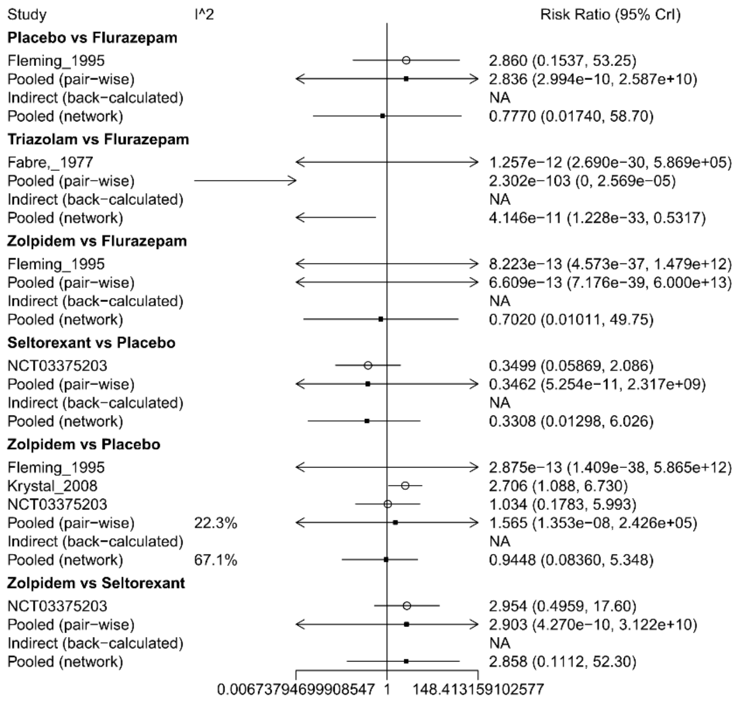

Results of pairwise meta-analysis for difficulty concentrating

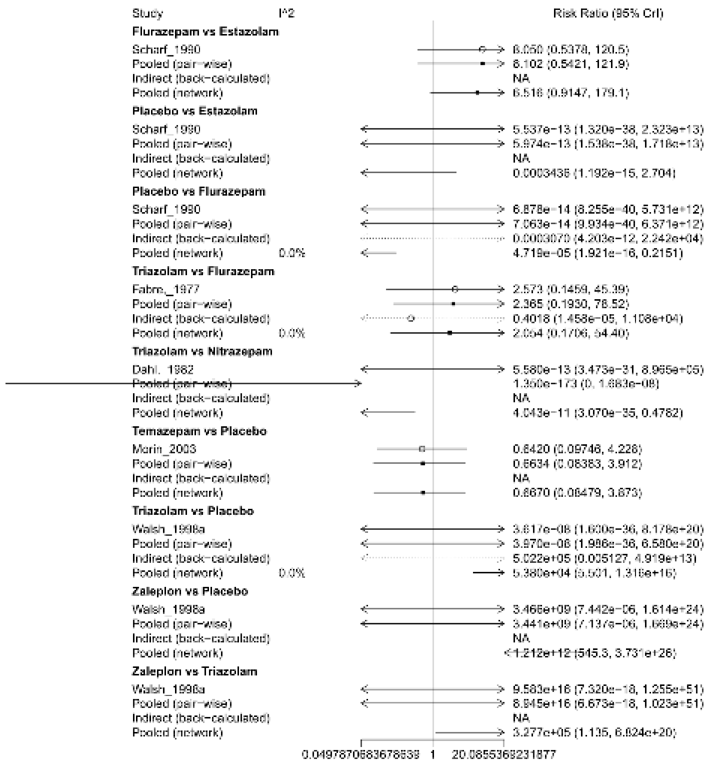

Results of pairwise meta-analysis for impaired coordination

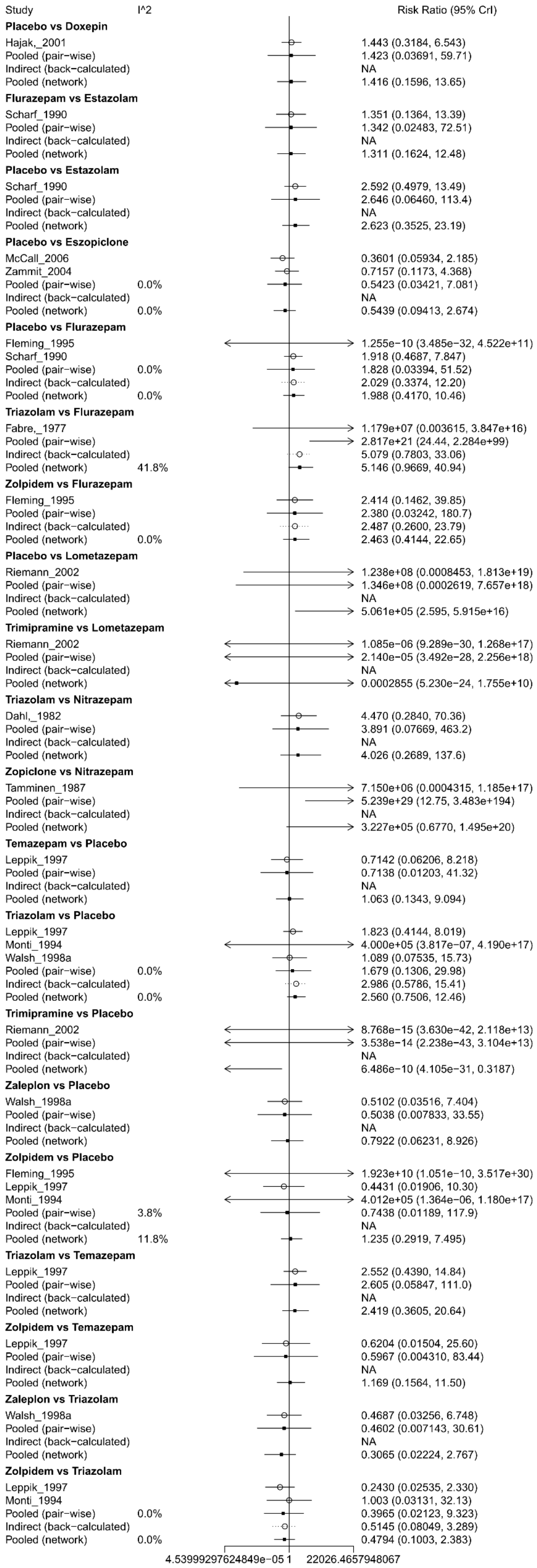

Results of pairwise meta-analysis for nervousness

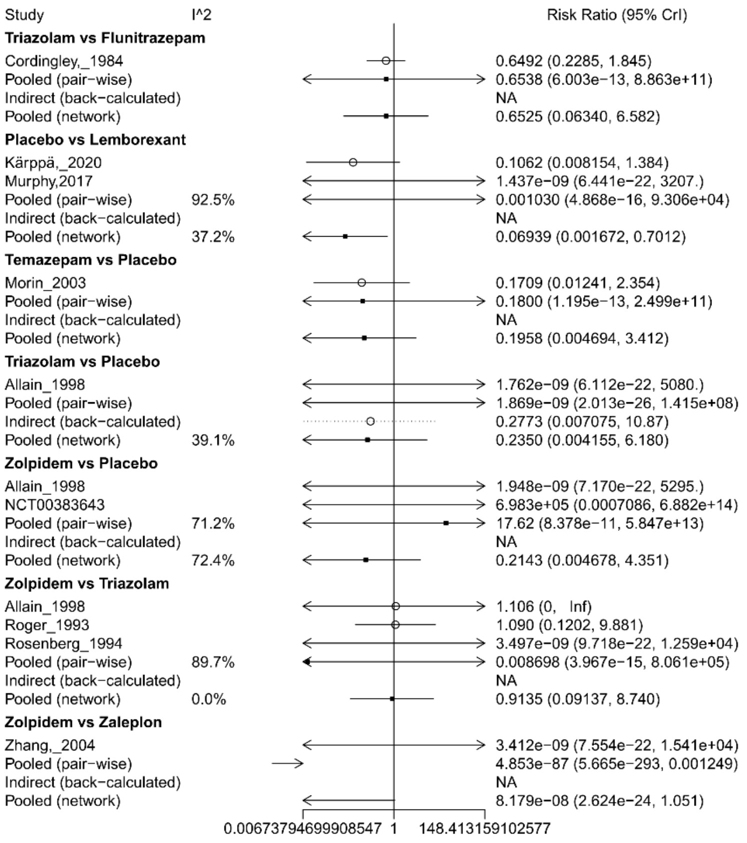

Results of pairwise meta-analysis for nightmare

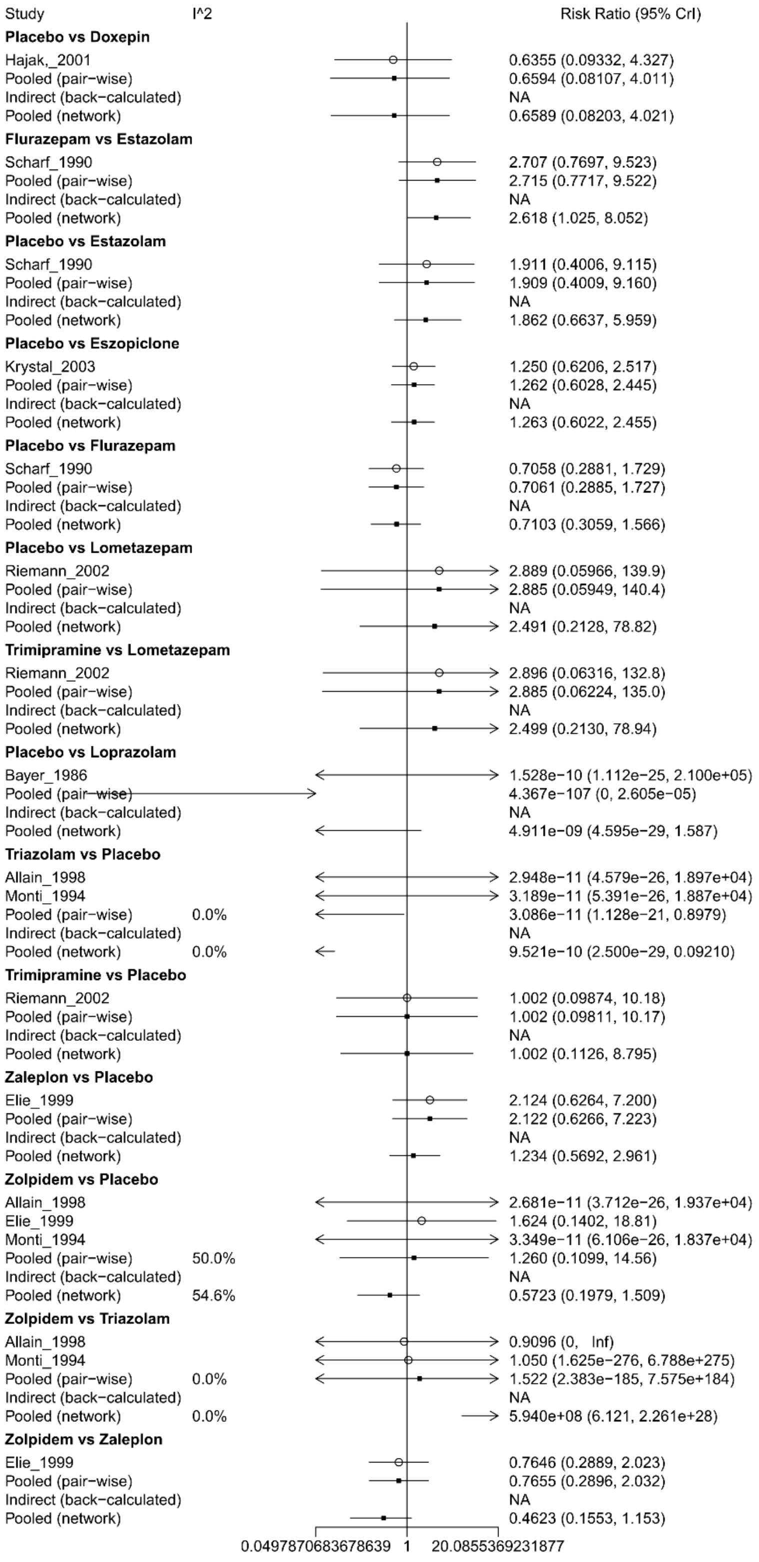

Results of pairwise meta-analysis for asthenia

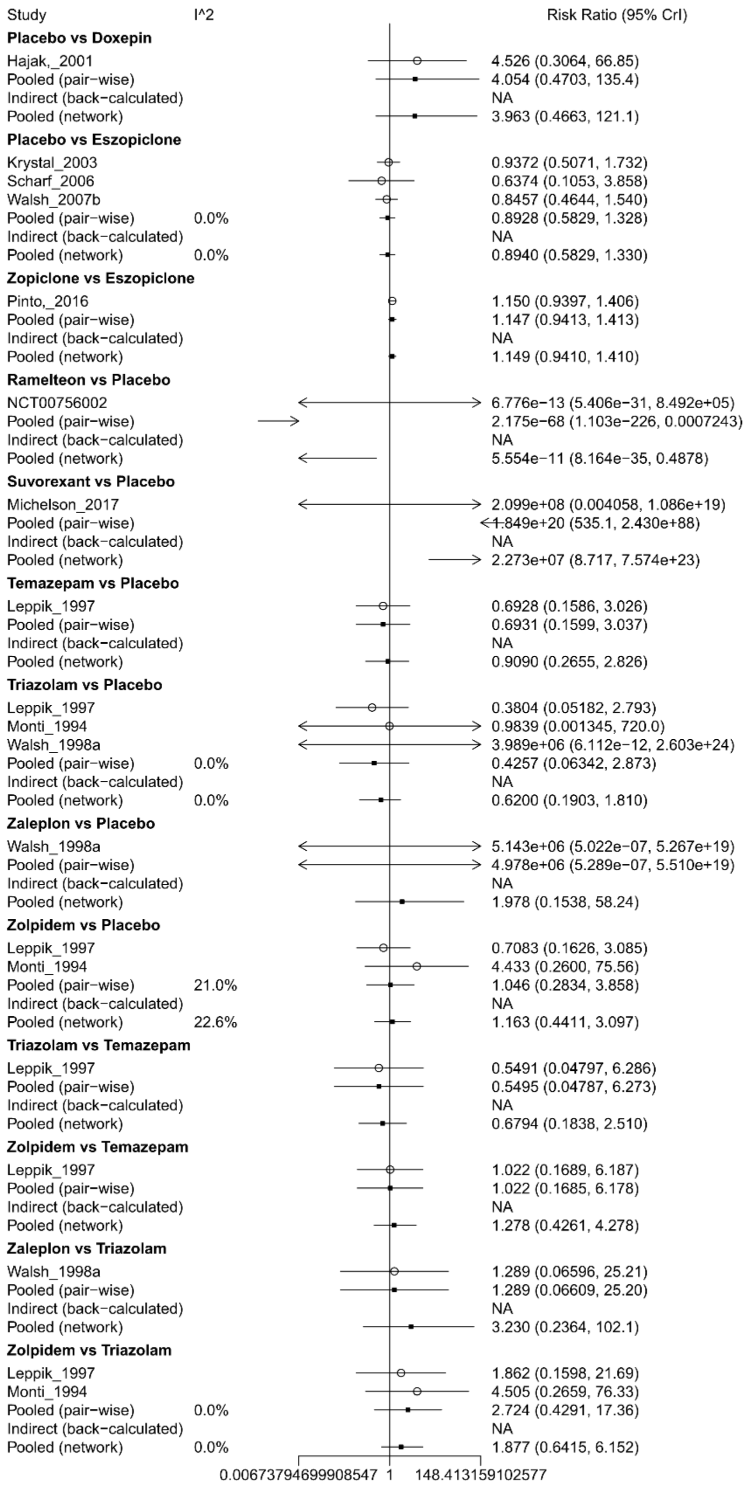

Results of pairwise meta-analysis for dyspepsia

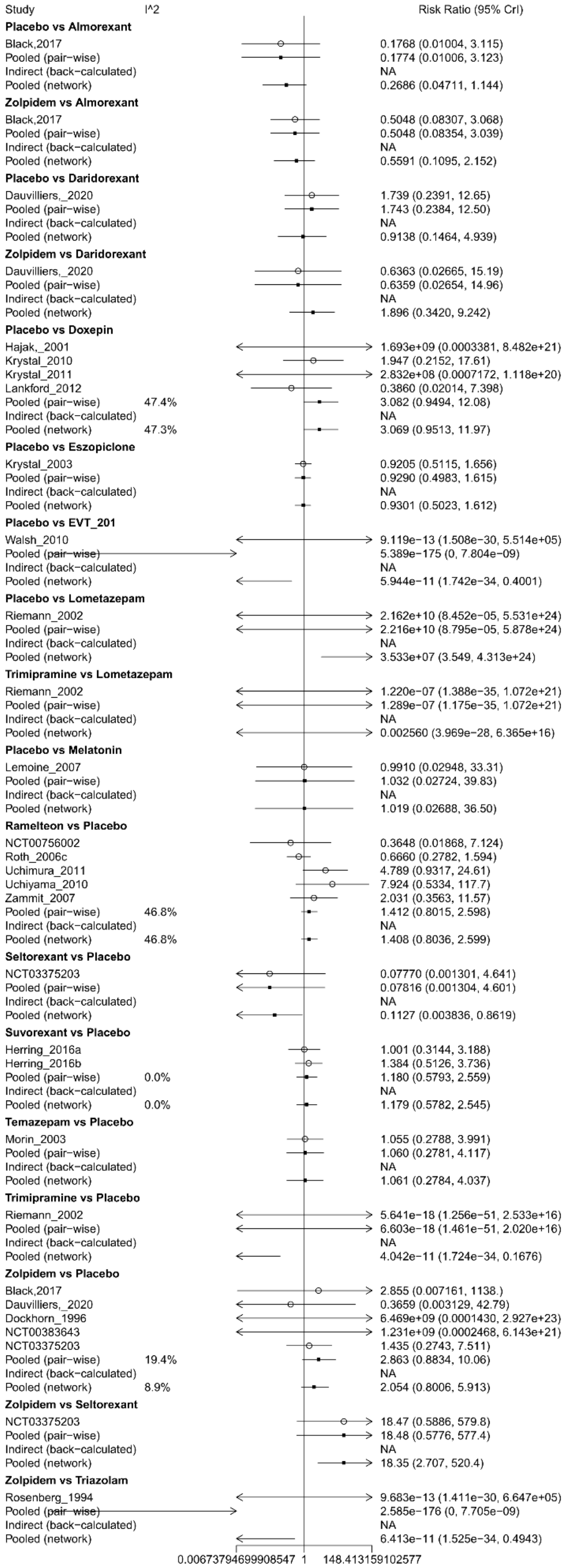

Results of pairwise meta-analysis for diarrhea

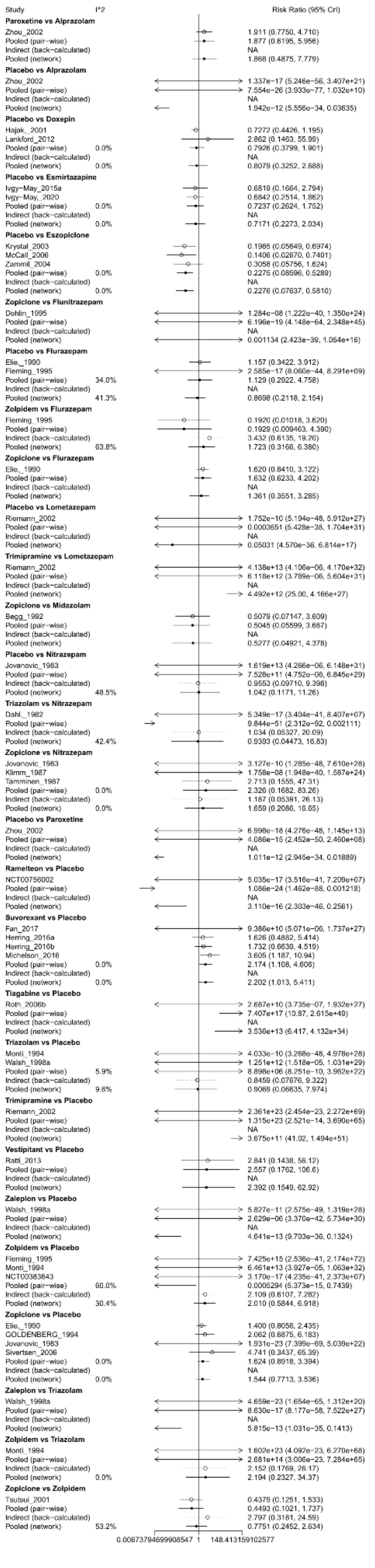

Results of pairwise meta-analysis for dry mouth

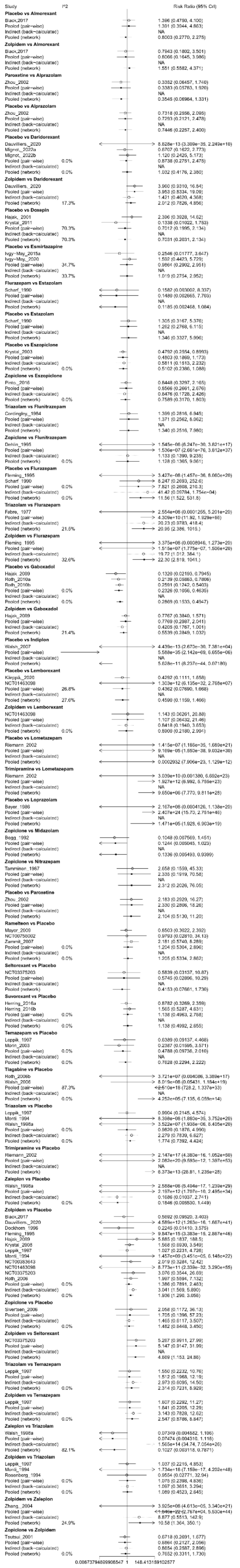

Results of pairwise meta-analysis for nausea/vomiting

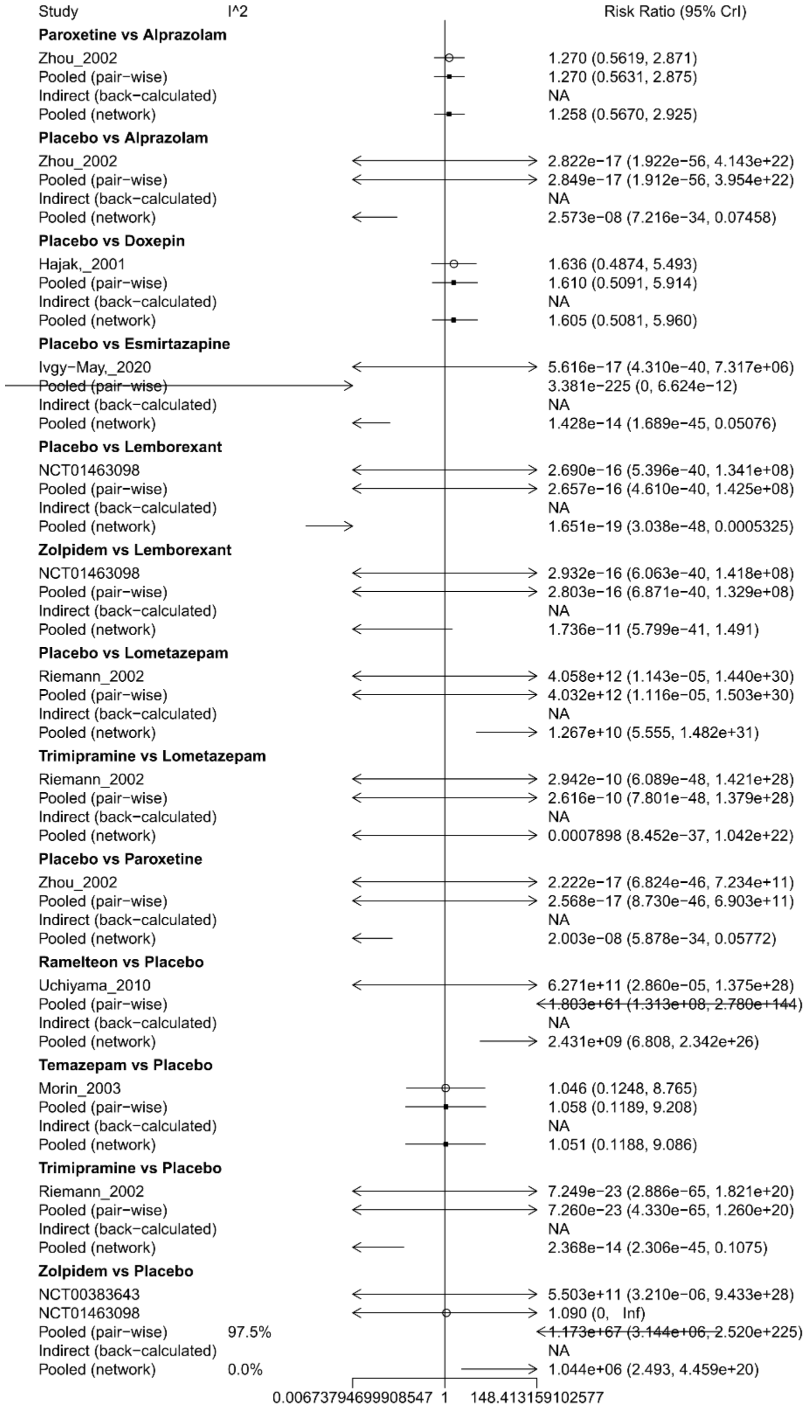

Results of pairwise meta-analysis for constipation

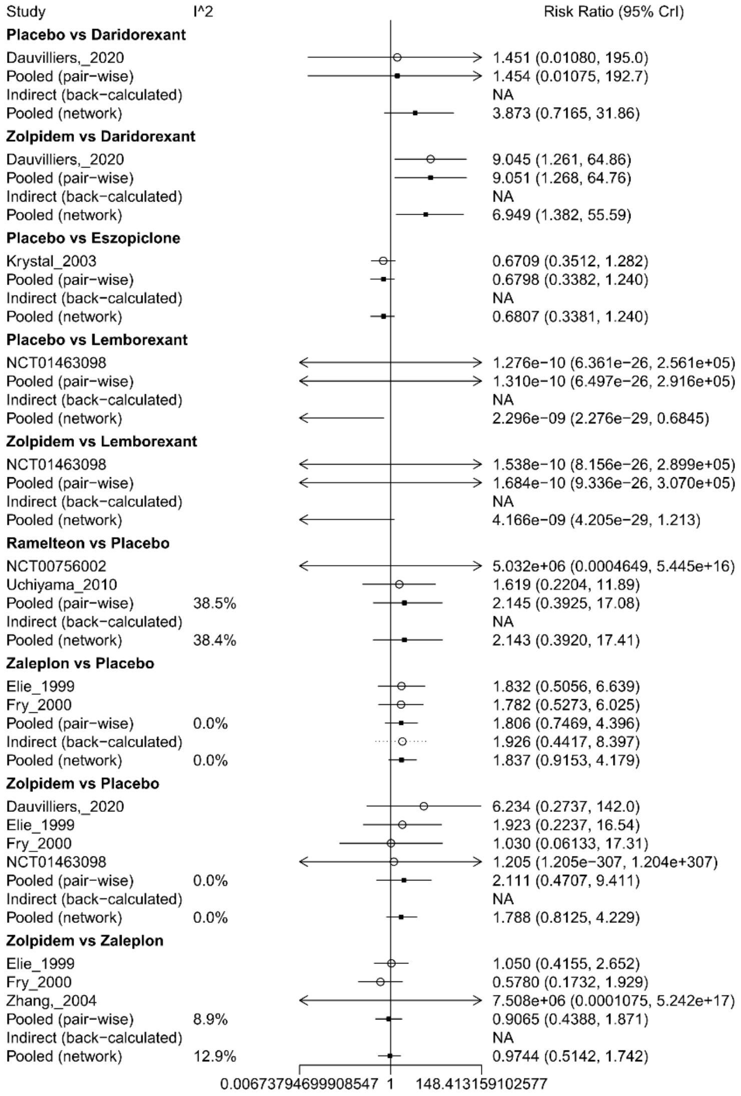

Results of pairwise meta-analysis for abdominal pain

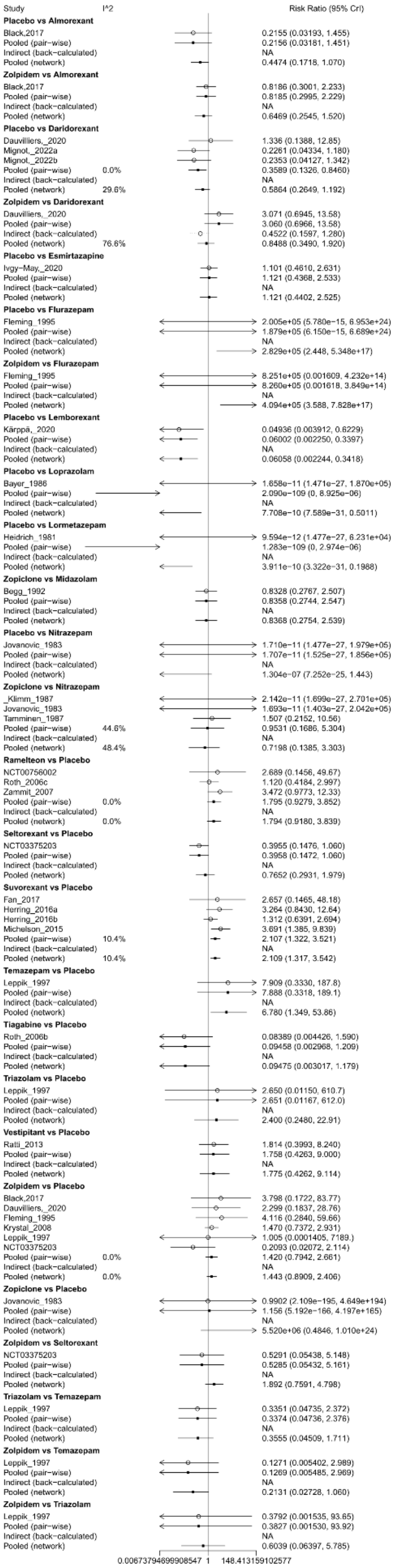

Results of pairwise meta-analysis for fatigue

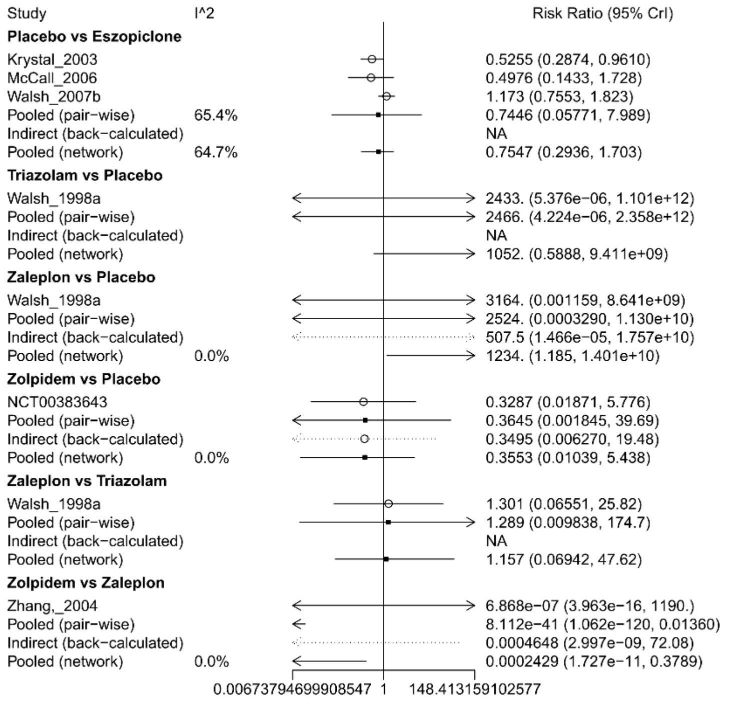

Results of pairwise meta-analysis for pain

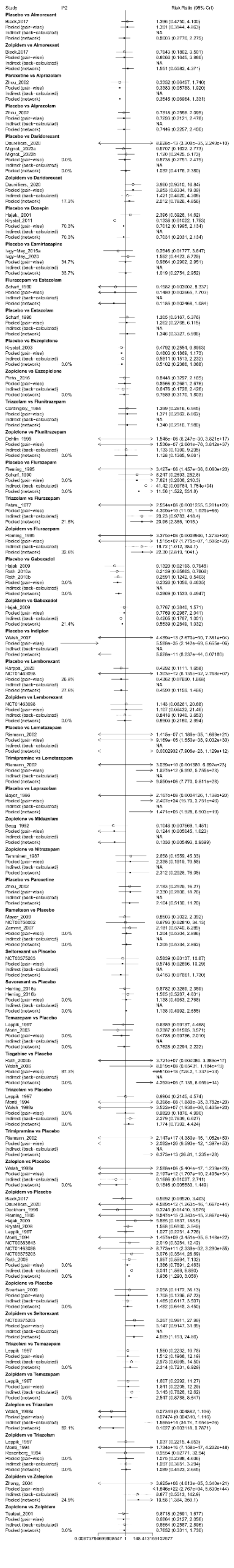

Results of pairwise meta-analysis for nasopharyngitis

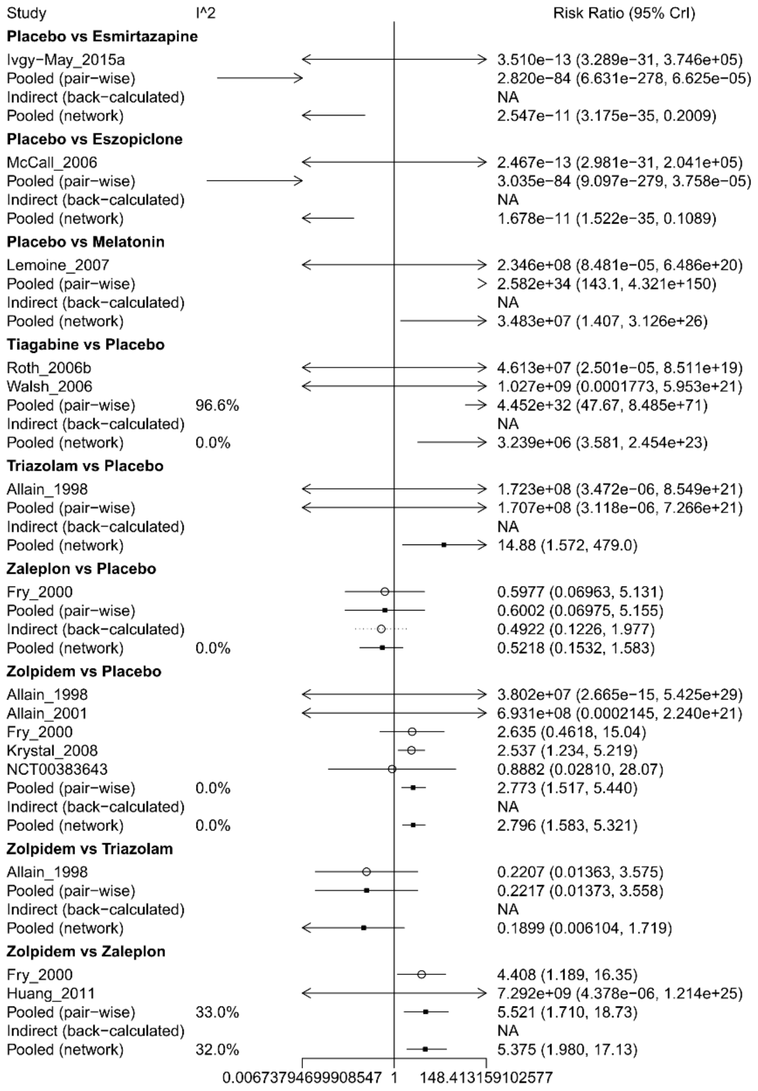

Results of pairwise meta-analysis for anxiety

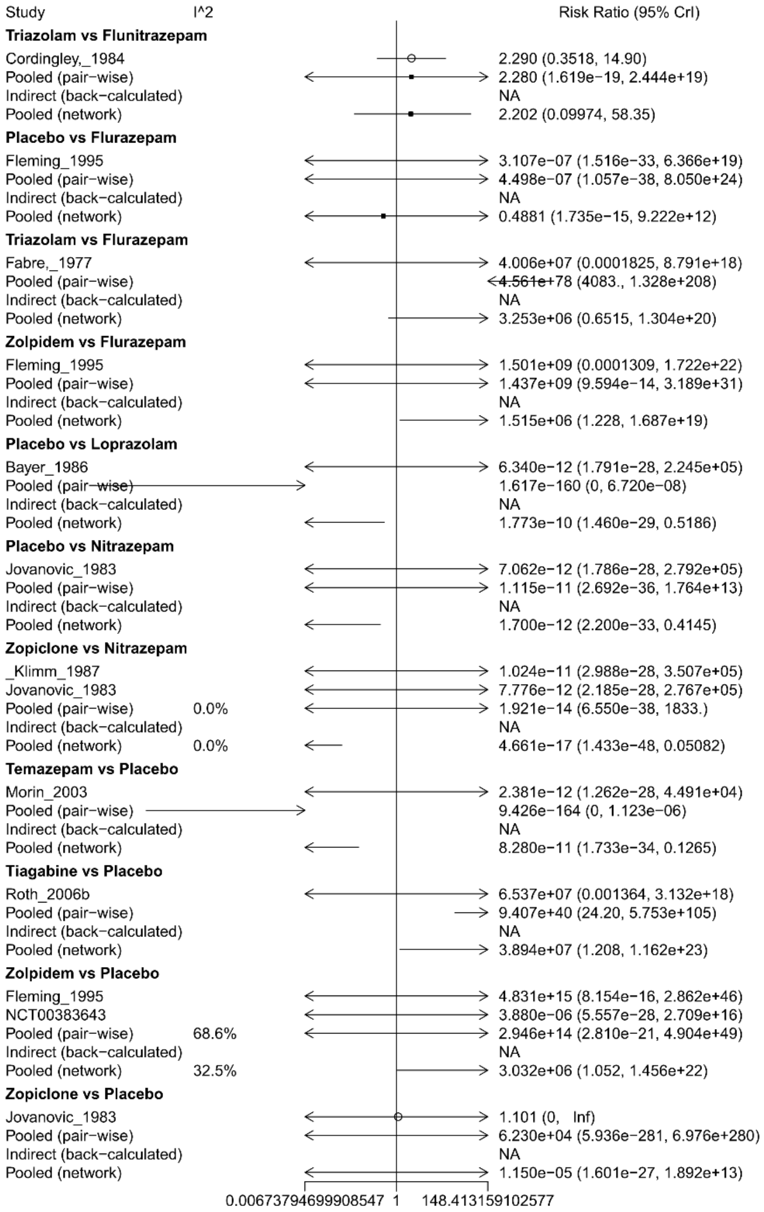

Results of pairwise meta-analysis for confusional state

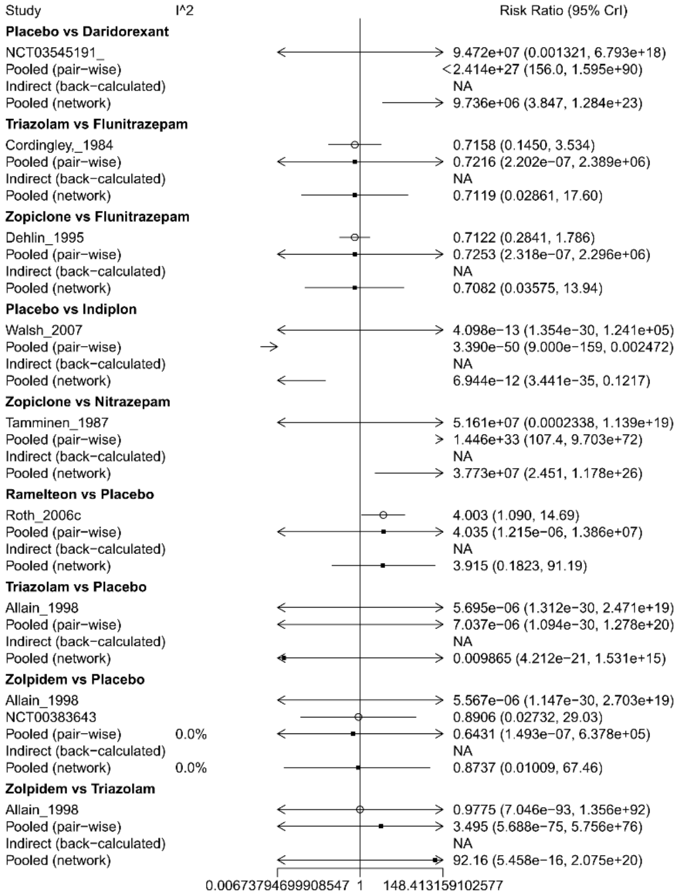

Results of pairwise meta-analysis for depression

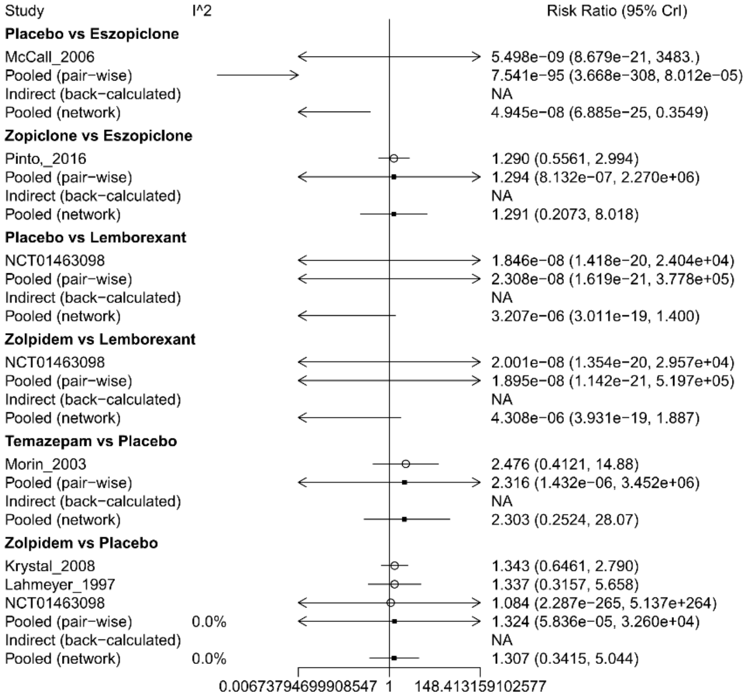

Results of pairwise meta-analysis for emotional lability

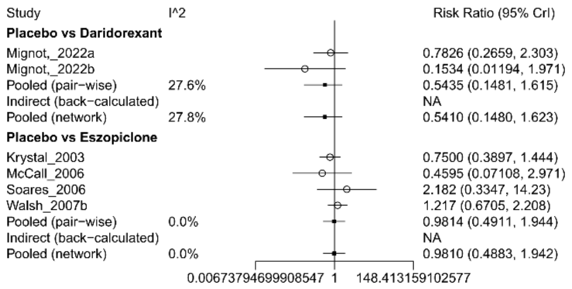

Results of pairwise meta-analysis for accidental injury

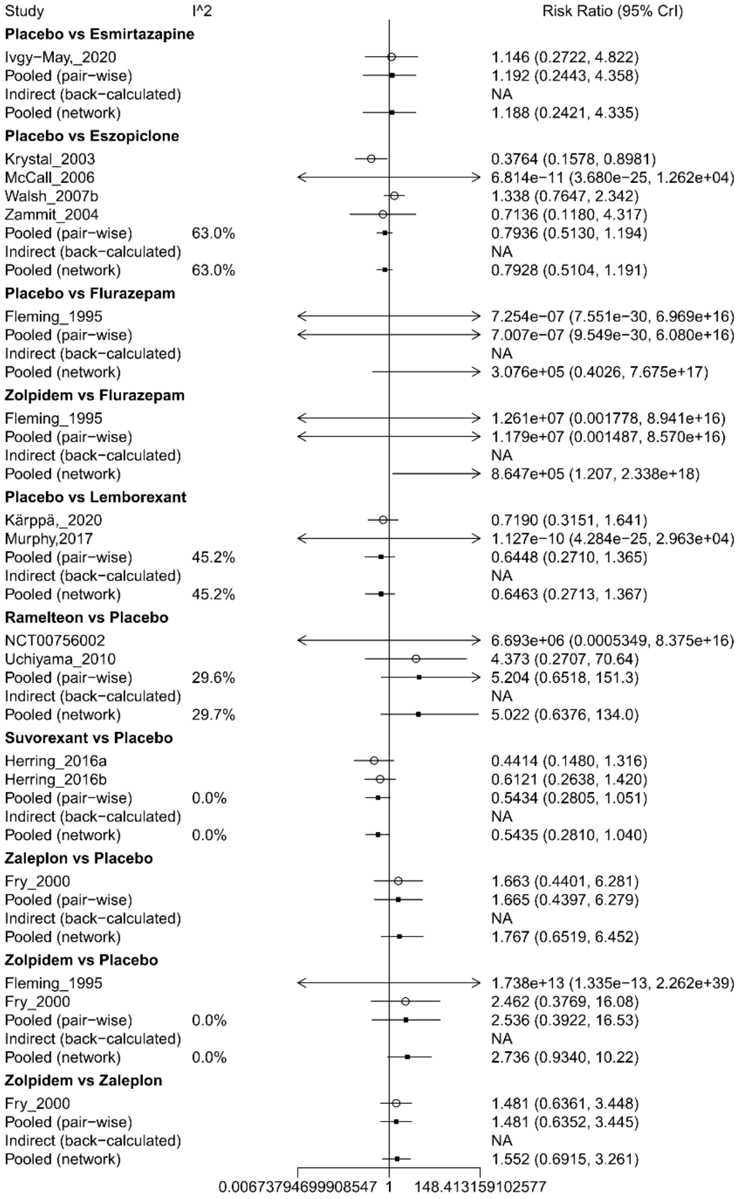

Results of pairwise meta-analysis for back pain

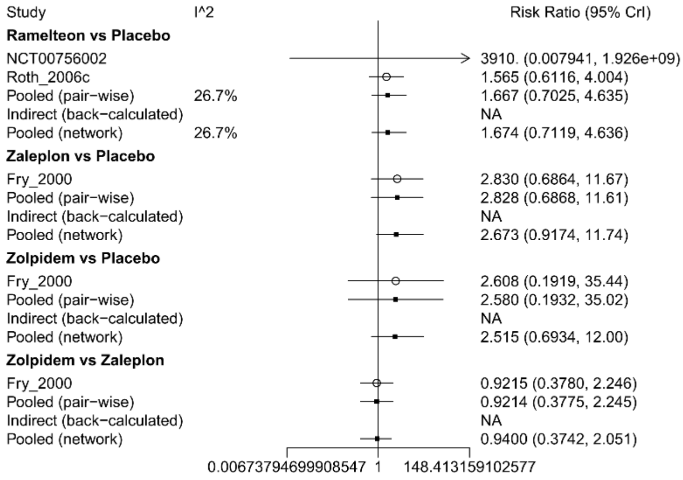

Results of pairwise meta-analysis for eye pain

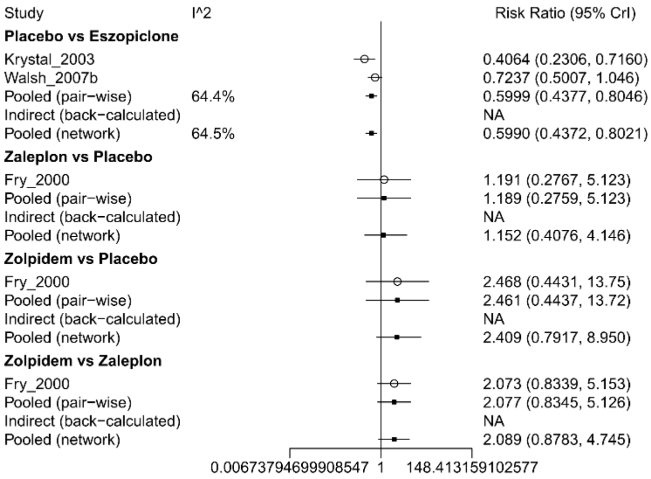

Results of pairwise meta-analysis for infection

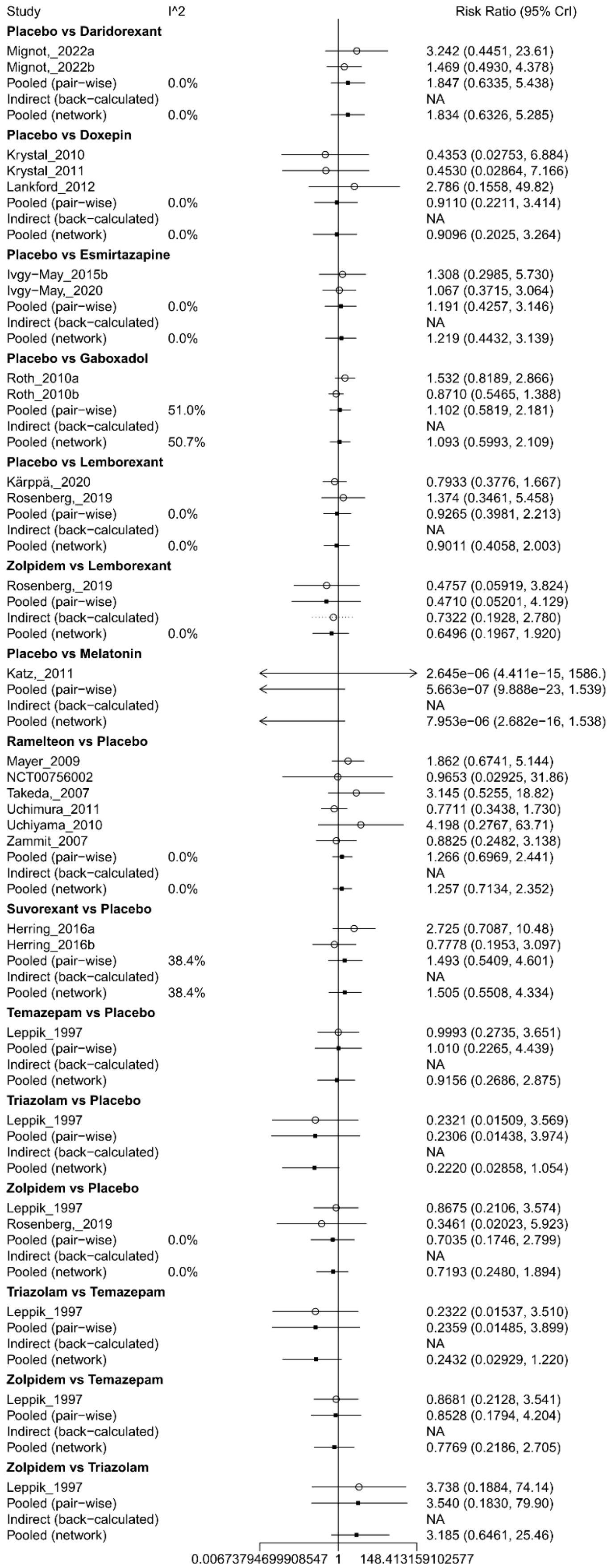

Results of pairwise meta-analysis for upper respiratory tract infection

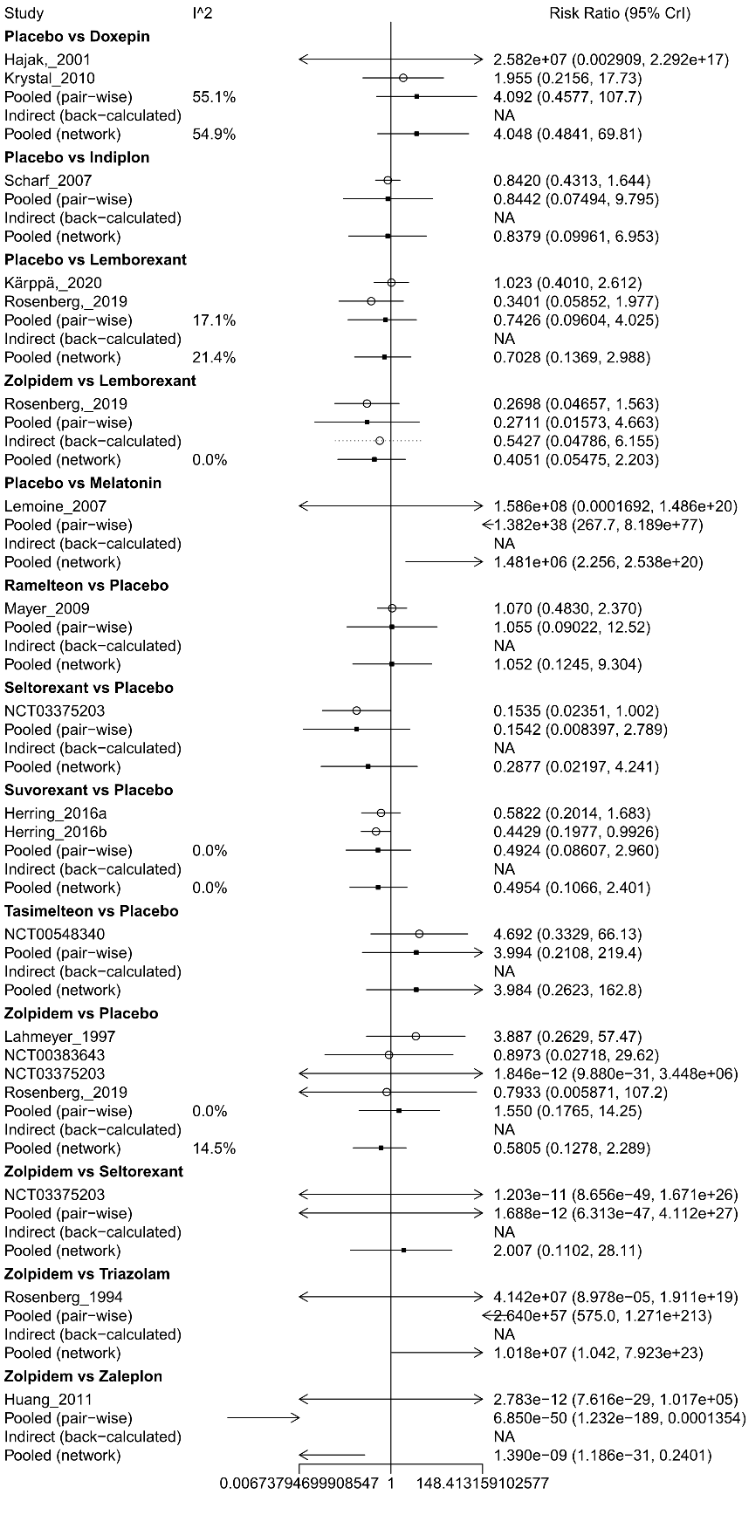

Results of pairwise meta-analysis for urinary tract infection

Results of pairwise meta-analysis for secondary outcomes

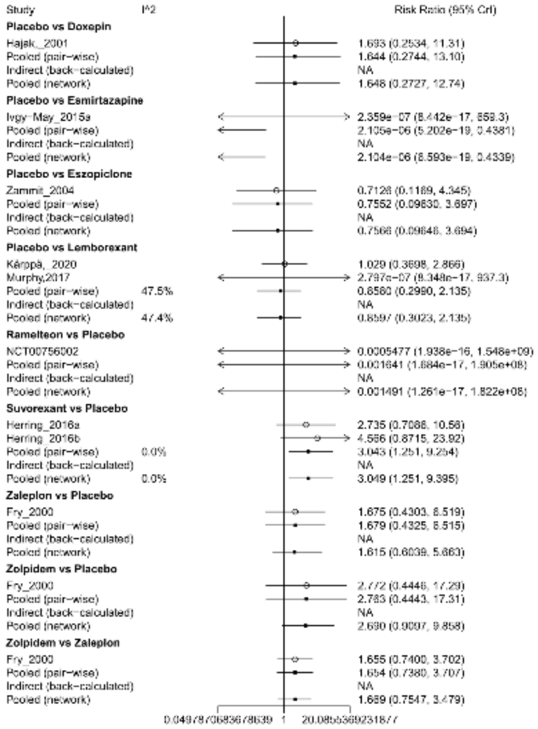

Results of pairwise meta-analysis for abnormal dreams

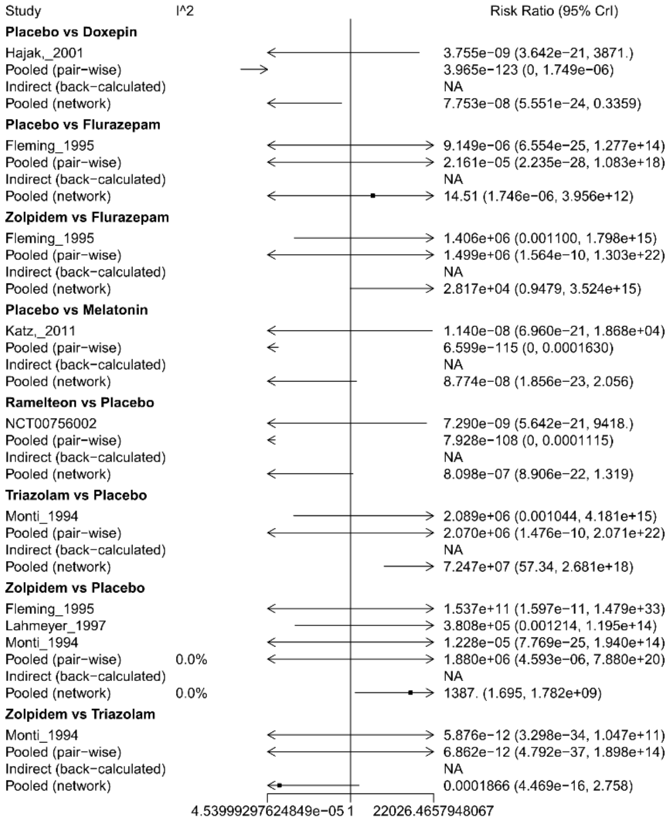

Results of pairwise meta-analysis for abnormal vision

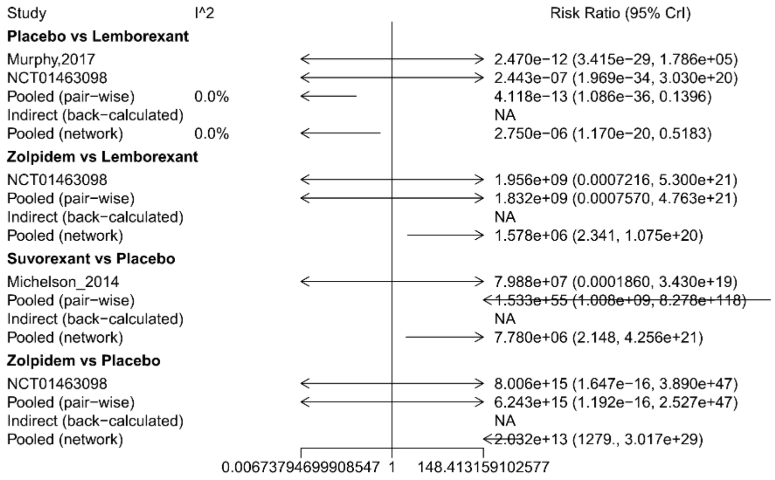

Results of pairwise meta-analysis for hypnagogic hallucinations

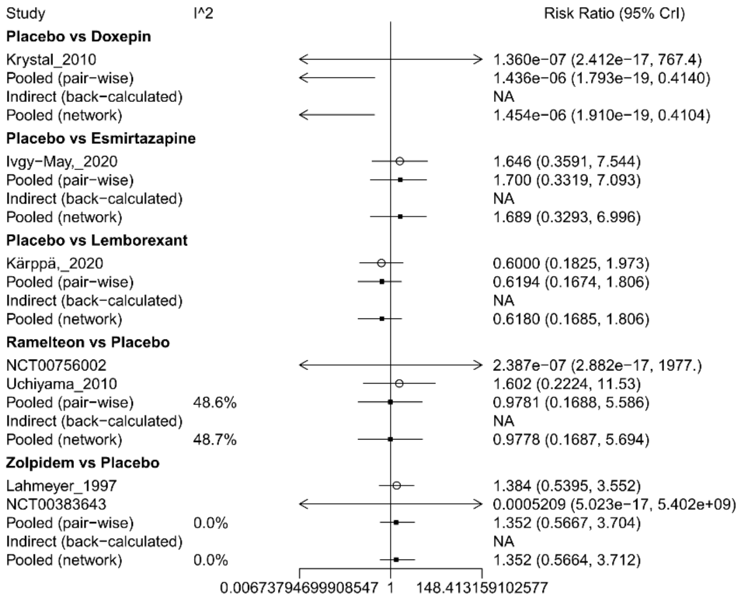

Results of pairwise meta-analysis for gastroenteritis

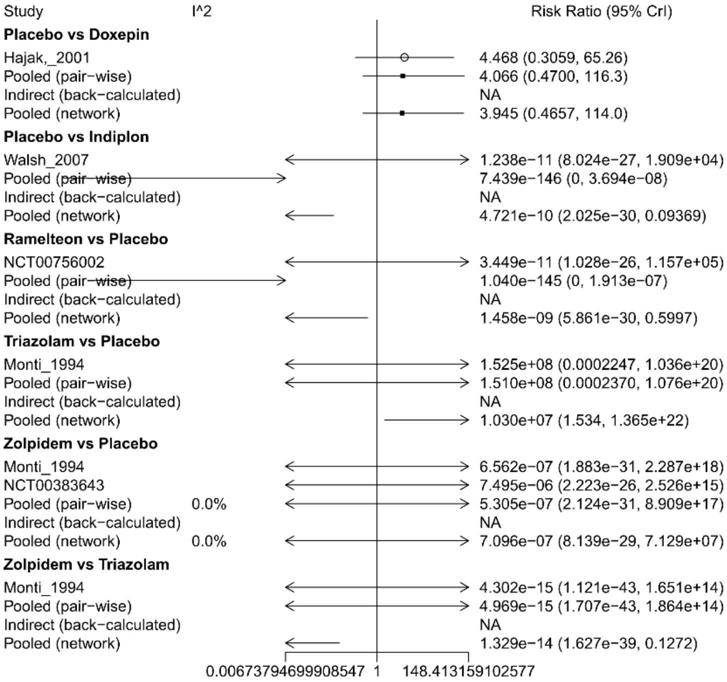

Results of pairwise meta-analysis for decreased appetite

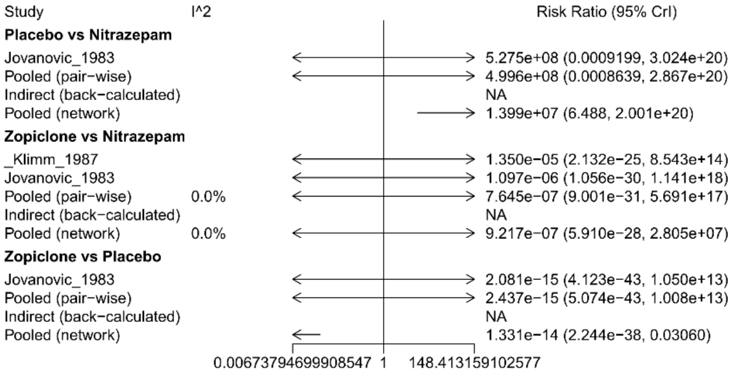

Results of pairwise meta-analysis for pain gastralgia

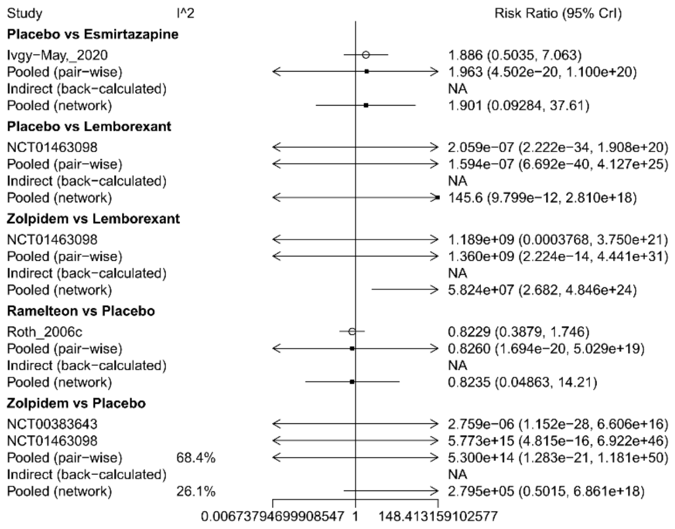

Results of pairwise meta-analysis for insomnia exacerbated

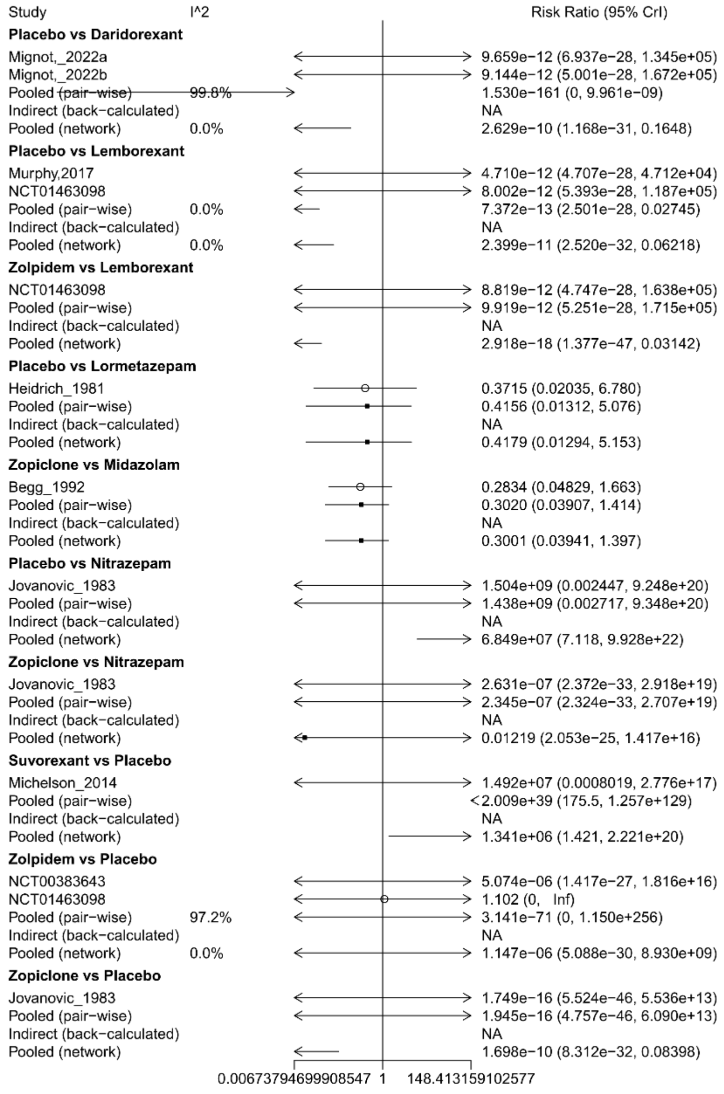

Results of pairwise meta-analysis for sleep paralysis

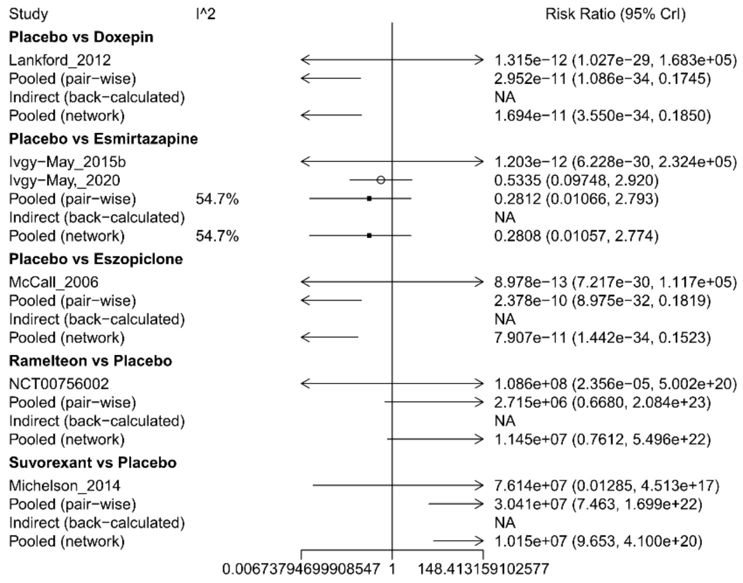

Results of pairwise meta-analysis for peripheral oedema

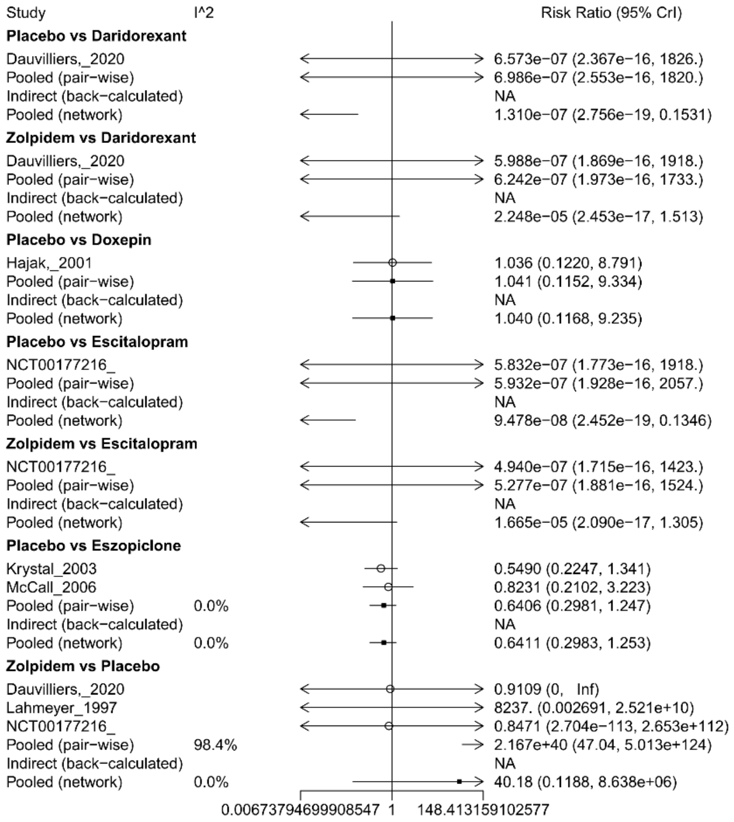

Results of pairwise meta-analysis for skin diseases

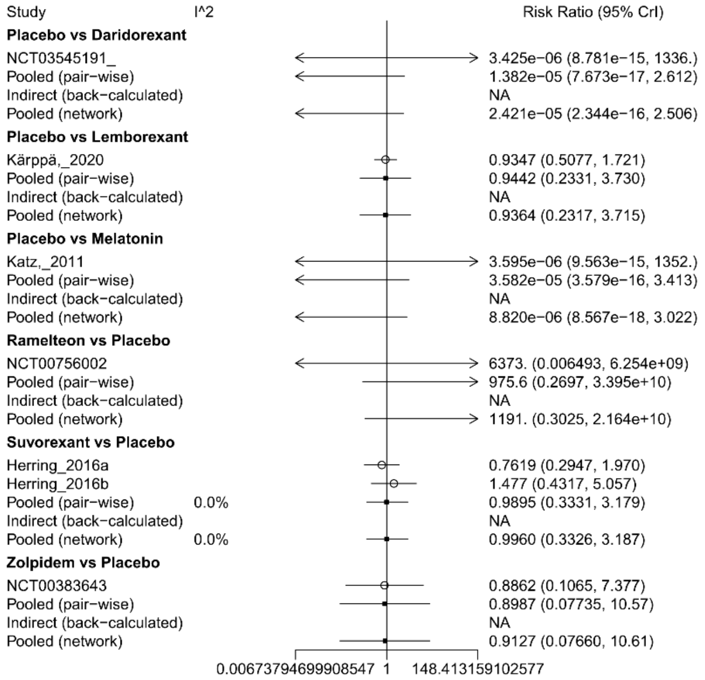

Results of pairwise meta-analysis for influenza

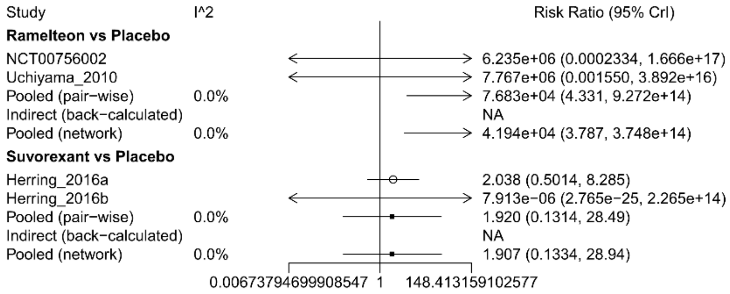

Results of pairwise meta-analysis for malaise

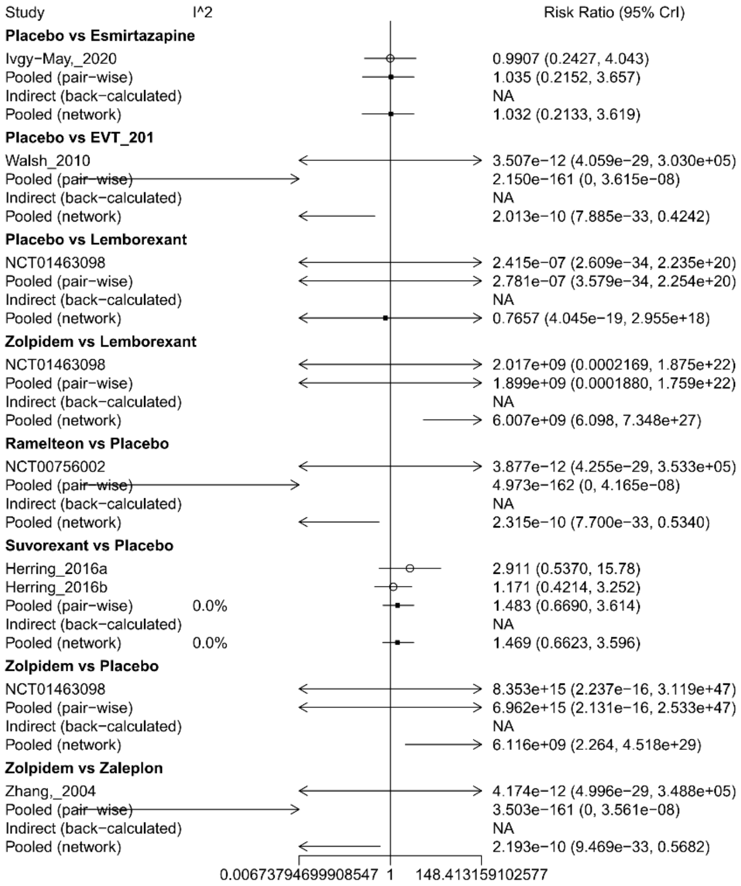

Results of pairwise meta-analysis for cough

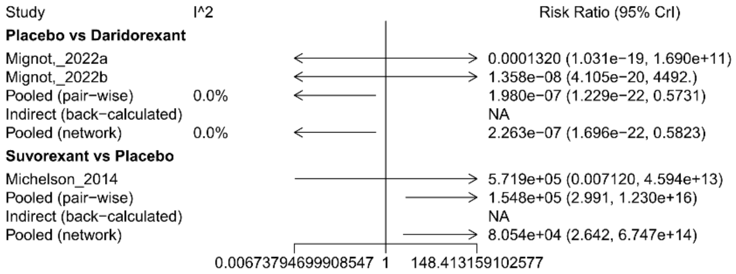

Results of pairwise meta-analysis for suicidal ideation

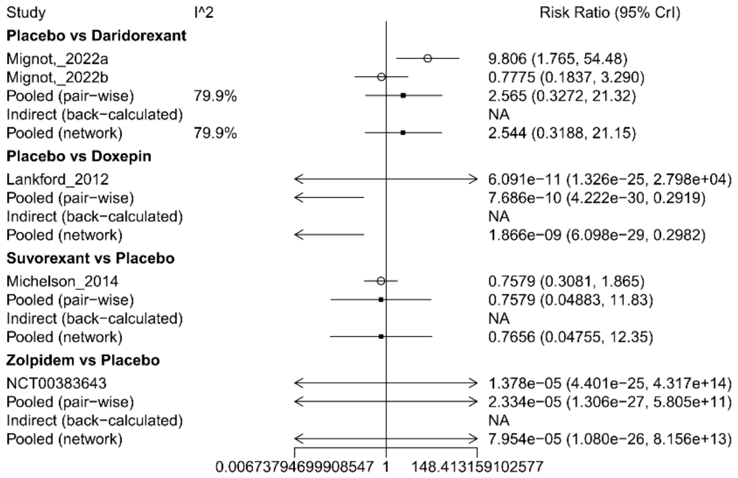

Results of pairwise meta-analysis for falls

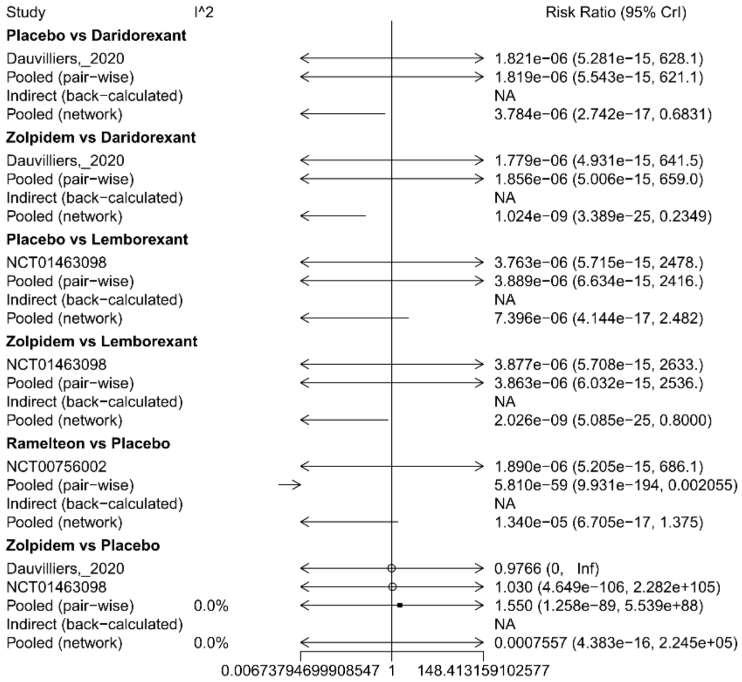

Results of pairwise meta-analysis for alanine aminotransferase increased

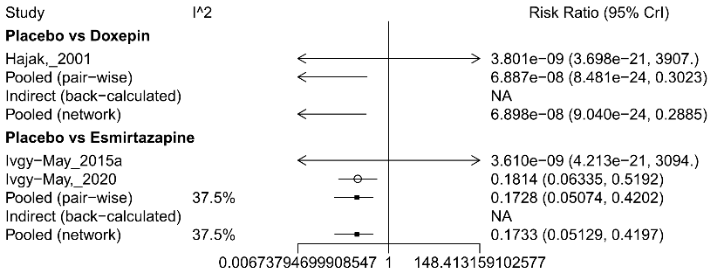

Results of pairwise meta-analysis for weight increased

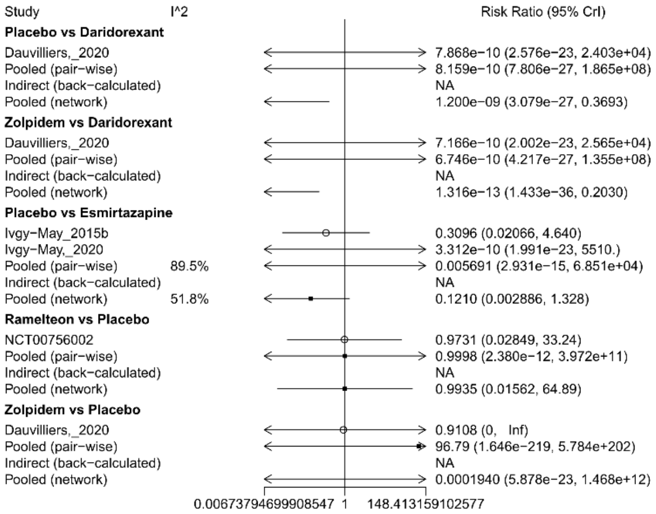

Results of pairwise meta-analysis for wei γ -Glutamyl transferase increased

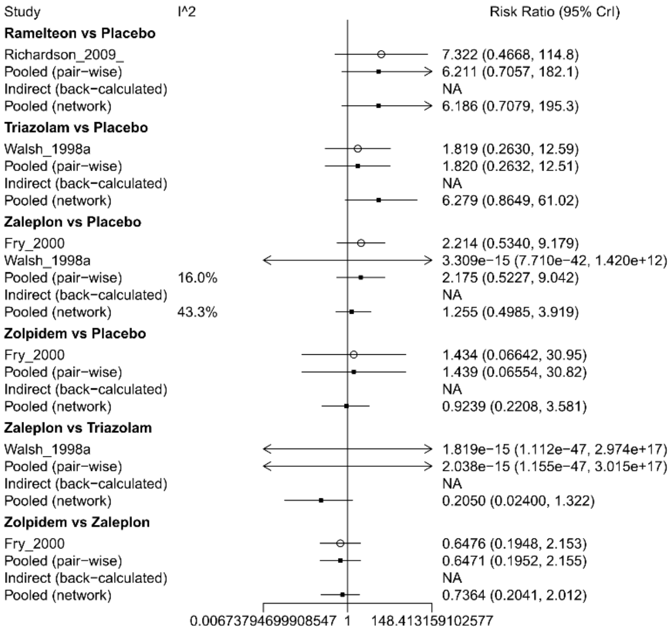

Results of pairwise meta-analysis for dysmenorrhea

Appendix 11 Results of network meta-analysis

Results of network meta-analysis for primary outcomes

League table for somnolence

Almorexant	3782126555790.51 (29.99, 2.67823031002925e+30)	0.89 (0.27, 2.96)	0.51 (0.14, 1.71)	2.43 (0.69, 8.84)	1.09 (0.33, 3.63)	1.05 (0.34, 3.25)	2.48 (0.26, 66.98)	1.61 (0.4, 6.46)	1.33 (0.43, 4.03)	1.15 (0.28, 4.93)	1.85 (0.51, 7.3)	3.5 (1.06, 12.2)	1.11 (0.31, 4.01)	2.38781421858792e+23 (2.2, 9.2335228041538 2e+47)	2.58 (0.38, 18.58)	2.01 (0.45, 9.47)	0 (0, 4831538361455.4)	0.53 (0.19, 1.47)	1.15 (0.38, 3.66)	0.5 (0.1, 2.77)	1.75 (0.57, 5.38)	1.97 (0.48, 7.86)	0.65 (0.1, 6.36)	1.49 (0.44, 5.15)	1.25 (0.38, 3.99)	0.71 (0.23, 2.34)	0.98 (0.35, 2.69)	1.06 (0.32, 3.4)
0 (0, 0.03)	Alprazolam	0 (0, 0.03)	0 (0, 0.02)	0 (0, 0.08)	0 (0, 0.04)	0 (0, 0.03)	0 (0, 0.08)	0 (0, 0.05)	0 (0, 0.04)	0 (0, 0.04)	0 (0, 0.06)	0 (0, 0.12)	0 (0, 0.04)	10275986.46 (0, 2.3432365167406 6e+42)	0 (0, 0.1)	0 (0, 0.06)	0 (0, 0)	0 (0, 0.02)	0 (0, 0.04)	0 (0, 0.02)	0 (0, 0.05)	0 (0, 0.07)	0 (0, 0.02)	0 (0, 0.05)	0 (0, 0.04)	0 (0, 0.02)	0 (0, 0.03)	0 (0, 0.03)
1.13 (0.34, 3.74)	4299538955288.16 (36.1, 2.93352402262925e+30)	Daridorexant	0.57 (0.23, 1.46)	2.77 (1.08, 7.36)	1.24 (0.51, 2.96)	1.19 (0.54, 2.56)	2.72 (0.38, 68.91)	1.82 (0.58, 5.69)	1.5 (0.69, 3.21)	1.29 (0.4, 4.42)	2.09 (0.77, 6.28)	3.97 (1.6, 10.21)	1.25 (0.46, 3.49)	2.7624250086436 2e+23 (2.25, 1.0217351999284 8e+48)	2.93 (0.51, 18.19)	2.26 (0.64, 8.56)	0 (0, 5711830400580.33)	0.6 (0.31, 1.11)	1.3 (0.59, 2.91)	0.56 (0.13, 2.54)	1.98 (0.87, 4.47)	2.23 (0.69, 6.91)	0.73 (0.13, 5.93)	1.7 (0.65, 4.31)	1.4 (0.6, 3.34)	0.81 (0.34, 1.9)	1.11 (0.55, 2.14)	1.2 (0.5, 2.81)
1.97 (0.58, 7.07)	7370682298985.1 (63.87, 5.51661375247021e+30)	1.76 (0.69, 4.43)	Doxepin	4.84 (1.88, 13.17)	2.18 (0.87, 5.3)	2.09 (0.92, 4.69)	4.75 (0.63, 119.9)	3.21 (1, 10.26)	2.64 (1.17, 5.83)	2.26 (0.71, 7.9)	3.64 (1.28, 11.1)	6.9 (2.74, 18.25)	2.21 (0.77, 6.27)	4.1458787222421e+23 (3.88, 2.1592402508904 4e+48)	5.14 (0.89, 32.38)	3.99 (1.06, 15.58)	0 (0, 10364636529494)	1.04 (0.53, 2.01)	2.29 (1.03, 5.31)	0.99 (0.22, 4.55)	3.47 (1.55, 7.93)	3.94 (1.19, 12.52)	1.27 (0.24, 10.59)	2.99 (1.11, 7.83)	2.48 (0.98, 6.06)	1.42 (0.58, 3.47)	1.93 (0.92, 3.98)	2.11 (0.86, 5.06)
0.41 (0.11, 1.46)	1588625965426.7 (12.54, 1.04037854997892e+30)	0.36 (0.14, 0.93)	0.21 (0.08, 0.53)	Esmirtazapine	0.45 (0.17, 1.12)	0.43 (0.18, 0.98)	0.99 (0.13, 24.79)	0.65 (0.2, 2.15)	0.55 (0.23, 1.22)	0.47 (0.14, 1.62)	0.75 (0.26, 2.31)	1.43 (0.54, 3.74)	0.45 (0.15, 1.31)	8.3238503119254 8e+22 (0.82, 4.1629379028275 8e+47)	1.06 (0.18, 6.49)	0.82 (0.21, 3.24)	0 (0, 2047444149653.61)	0.22 (0.1, 0.42)	0.47 (0.2, 1.1)	0.2 (0.04, 0.96)	0.72 (0.3, 1.62)	0.81 (0.23, 2.57)	0.26 (0.05, 2.18)	0.61 (0.21, 1.64)	0.5 (0.2, 1.29)	0.29 (0.11, 0.73)	0.4 (0.18, 0.84)	0.44 (0.17, 1.07)
0.92 (0.28, 3.01)	3470930662094.96 (27.99, 2.61740627627518e+30)	0.81 (0.34, 1.95)	0.46 (0.19, 1.15)	2.22 (0.9, 5.87)	Estazolam	0.96 (0.44, 2.03)	2.18 (0.3, 54.8)	1.47 (0.5, 4.35)	1.21 (0.69, 2.14)	1.04 (0.33, 3.53)	1.67 (0.64, 4.95)	3.17 (1.34, 8.03)	1.01 (0.39, 2.64)	2.2538839131982 7e+23 (1.87, 8.9959614339104 3e+47)	2.36 (0.43, 14.39)	1.84 (0.54, 6.41)	0 (0, 4936226888189.82)	0.48 (0.26, 0.88)	1.06 (0.5, 2.31)	0.46 (0.1, 2.03)	1.6 (0.75, 3.43)	1.81 (0.58, 5.35)	0.59 (0.11, 4.8)	1.37 (0.54, 3.43)	1.13 (0.52, 2.49)	0.65 (0.29, 1.51)	0.89 (0.46, 1.69)	0.97 (0.44, 2.1)
0.95 (0.31, 2.94)	3581703063437.16 (30.13, 2.13887295029855e+30)	0.84 (0.39, 1.87)	0.48 (0.21, 1.09)	2.33 (1.02, 5.56)	1.04 (0.49, 2.27)	Eszopiclone	2.27 (0.32, 56.4)	1.51 (0.53, 4.68)	1.26 (0.67, 2.42)	1.08 (0.38, 3.49)	1.75 (0.73, 4.8)	3.29 (1.52, 7.66)	1.04 (0.43, 2.77)	1.9164094556204 2e+23 (1.98, 8.9526875537045 5e+47)	2.44 (0.46, 14.75)	1.89 (0.58, 6.84)	0 (0, 5050262064093.46)	0.5 (0.31, 0.79)	1.1 (0.59, 2.15)	0.47 (0.11, 2.03)	1.65 (0.88, 3.21)	1.87 (0.63, 5.5)	0.61 (0.12, 4.82)	1.43 (0.61, 3.36)	1.17 (0.56, 2.6)	0.68 (0.32, 1.47)	0.92 (0.53, 1.59)	1.01 (0.48, 2.14)
0.4 (0.01, 3.79)	1439165129303.2 (11.98, 1.217776	0.37 (0.01, 2.64)	0.21 (0.01, 1.58)	1.01 (0.04, 7.93)	0.46 (0.02, 3.34)	0.44 (0.02, 3.09)	EVT_201	0.67 (0.02, 5.6)	0.56 (0.02, 3.87)	0.48 (0.02, 4.16)	0.78 (0.03, 6.02)	1.46 (0.06, 11.02)	0.47 (0.02, 3.56)	9.4453446200070 9e+22 (0.84,	1.03 (0.03, 14.91)	0.83 (0.03, 7.49)	0 (0, 235004	0.22 (0.01, 1.45)	0.48 (0.02, 3.38)	0.2 (0.01, 2.27)	0.73 (0.03, 5.04)	0.81 (0.03, 6.96)	0.26 (0.01, 4.12)	0.62 (0.02, 4.74)	0.53 (0.02, 3.73)	0.3 (0.01, 2.21)	0.41 (0.02, 2.76)	0.44 (0.02, 3.25)

	4095629e+30)													2.49693695448502e+47)			9784227.93)											
0.62 (0.15, 2.48)	2447321104215.58 (18.77, 1.91548227964939e+30)	0.55 (0.18, 1.73)	0.31 (0.1, 1)	1.53 (0.46, 5.08)	0.68 (0.23, 2)	0.66 (0.21, 1.87)	1.5 (0.18, 41.39)	Flunitrazepam	0.83 (0.31, 2.15)	0.71 (0.18, 2.91)	1.15 (0.33, 4.16)	2.16 (0.68, 6.97)	0.69 (0.28, 1.73)	1.25254852870105e+23 (1.19, 5.7704316653109 1e+47)	1.58 (0.27, 10.72)	1.25 (0.37, 4.27)	0 (0, 3132159358027.69)	0.33 (0.12, 0.85)	0.72 (0.24, 2.11)	0.31 (0.06, 1.63)	1.09 (0.37, 3.13)	1.23 (0.36, 4.1)	0.4 (0.06, 3.78)	0.94 (0.28, 3)	0.77 (0.36, 1.62)	0.45 (0.15, 1.31)	0.61 (0.23, 1.55)	0.66 (0.23, 1.89)
0.75 (0.25, 2.31)	2841262334409.73 (23.43, 2.08810186800754e+30)	0.67 (0.31, 1.45)	0.38 (0.17, 0.86)	1.83 (0.82, 4.44)	0.83 (0.47, 1.45)	0.79 (0.41, 1.49)	1.79 (0.26, 44.79)	1.21 (0.46, 3.25)	Flurazepam	0.86 (0.3, 2.69)	1.38 (0.58, 3.79)	2.63 (1.24, 5.98)	0.84 (0.37, 1.9)	1.6125849069211e+23 (1.54, 7.0998619124054 3e+47)	1.95 (0.37, 11.21)	1.52 (0.5, 4.87)	0 (0, 3953730065170.28)	0.4 (0.25, 0.62)	0.87 (0.46, 1.68)	0.38 (0.09, 1.58)	1.32 (0.68, 2.51)	1.49 (0.53, 4.02)	0.48 (0.1, 3.85)	1.13 (0.49, 2.54)	0.94 (0.51, 1.74)	0.54 (0.27, 1.1)	0.73 (0.45, 1.17)	0.8 (0.43, 1.47)
0.87 (0.2, 3.55)	3451870326834.8 (26.02, 2.53552699989104e+30)	0.78 (0.23, 2.48)	0.44 (0.13, 1.41)	2.13 (0.62, 7.25)	0.96 (0.28, 3.01)	0.93 (0.29, 2.65)	2.09 (0.24, 54.92)	1.4 (0.34, 5.58)	1.16 (0.37, 3.37)	Gaboxadol	1.61 (0.45, 6.11)	3 (0.91, 10.17)	0.97 (0.26, 3.44)	1.64573471663841e+23 (1.78, 8.66063570741804e+47)	2.22 (0.34, 15.75)	1.73 (0.39, 8.03)	0 (0, 4384930229478.83)	0.46 (0.16, 1.2)	1.01 (0.33, 2.98)	0.43 (0.08, 2.35)	1.53 (0.49, 4.44)	1.72 (0.42, 6.7)	0.55 (0.08, 5.17)	1.31 (0.38, 4.28)	1.09 (0.33, 3.41)	0.62 (0.19, 1.94)	0.86 (0.3, 2.21)	0.93 (0.28, 2.79)
0.54 (0.14, 1.95)	2051417577151.7 (17.1, 1.39161226741975e+30)	0.48 (0.16, 1.3)	0.27 (0.09, 0.78)	1.34 (0.43, 3.83)	0.6 (0.2, 1.55)	0.57 (0.21, 1.37)	1.28 (0.17, 32.11)	0.87 (0.24, 3.03)	0.73 (0.26, 1.72)	0.62 (0.16, 2.24)	Indiplon	1.89 (0.64, 5.24)	0.6 (0.18, 1.8)	1.16540431303251e+23 (1.05, 4.01351902334281e+47)	1.4 (0.22, 9.16)	1.08 (0.27, 4.26)	0 (0, 3142628195000.28)	0.29 (0.12, 0.6)	0.63 (0.23, 1.51)	0.27 (0.05, 1.29)	0.95 (0.35, 2.3)	1.08 (0.28, 3.64)	0.34 (0.06, 3.06)	0.82 (0.25, 2.26)	0.67 (0.23, 1.76)	0.39 (0.13, 1.02)	0.53 (0.2, 1.18)	0.58 (0.19, 1.5)
0.29 (0.08, 0.94)	1075511133693.75 (8.5, 7.04367748763297e+29)	0.25 (0.1, 0.62)	0.14 (0.05, 0.36)	0.7 (0.27, 1.86)	0.32 (0.12, 0.74)	0.3 (0.13, 0.66)	0.68 (0.09, 17.34)	0.46 (0.14, 1.48)	0.38 (0.17, 0.81)	0.33 (0.1, 1.1)	0.53 (0.19, 1.56)	Lemborexant	0.32 (0.11, 0.87)	6.41418598226258e+22 (0.65, 2.77169077900297e+47)	0.74 (0.13, 4.77)	0.57 (0.16, 2.12)	0 (0, 1606869068494.44)	0.15 (0.07, 0.28)	0.33 (0.15, 0.73)	0.14 (0.03, 0.64)	0.5 (0.22, 1.09)	0.57 (0.17, 1.75)	0.18 (0.03, 1.5)	0.43 (0.16, 1.09)	0.36 (0.14, 0.84)	0.21 (0.08, 0.48)	0.28 (0.13, 0.54)	0.3 (0.12, 0.71)
0.9 (0.25, 3.24)	3408114952390.7 (27.37, 2.5952045863685e+30)	0.8 (0.29, 2.18)	0.45 (0.16, 1.3)	2.22 (0.76, 6.58)	0.99 (0.38, 2.55)	0.96 (0.36, 2.35)	2.15 (0.28, 58.06)	1.46 (0.58, 3.63)	1.19 (0.53, 2.69)	1.03 (0.29, 3.82)	1.66 (0.56, 5.51)	3.15 (1.14, 9.02)	Loprazolam	1.88748082029371e+23 (1.79, 8.45897462474872e+47)	2.3 (0.42, 14.1)	1.82 (0.61, 5.49)	0 (0, 4135213877335.44)	0.48 (0.21, 1.06)	1.05 (0.42, 2.69)	0.45 (0.09, 2.2)	1.58 (0.61, 3.98)	1.79 (0.58, 5.27)	0.58 (0.1, 5.09)	1.36 (0.46, 3.83)	1.12 (0.64, 1.95)	0.64 (0.25, 1.66)	0.88 (0.39, 1.94)	0.96 (0.37, 2.41)
0 (0, 0.46)	0 (0, 9.49072141719934e+26)	0 (0, 0.44)	0 (0, 0.26)	0 (0, 1.22)	0 (0, 0.54)	0 (0, 0.51)	0 (0, 1.19)	0 (0, 0.84)	0 (0, 0.65)	0 (0, 0.56)	0 (0, 0.95)	0 (0, 1.55)	0 (0, 0.56)	Lormetazepam	0 (0, 1.33)	0 (0, 1.05)	0 (0, 1304239.21)	0 (0, 0.27)	0 (0, 0.59)	0 (0, 0.27)	0 (0, 0.88)	0 (0, 1.05)	0 (0, 0.25)	0 (0, 0.74)	0 (0, 0.63)	0 (0, 0.3)	0 (0, 0.47)	0 (0, 0.51)
0.39 (0.05, 2.63)	1256551029273.56 (10.45, 1.29554991368774e+30)	0.34 (0.05, 1.96)	0.19 (0.03, 1.13)	0.94 (0.15, 5.68)	0.42 (0.07, 2.34)	0.41 (0.07, 2.19)	0.97 (0.07, 30.14)	0.63 (0.09, 3.7)	0.51 (0.09, 2.67)	0.45 (0.06, 2.94)	0.72 (0.11, 4.5)	1.36 (0.21, 7.91)	0.43 (0.07, 2.37)	8.59021173656705e+22 (0.75, 3.41523	Mldazolam	0.79 (0.1, 5.22)	0 (0, 184644837520	0.2 (0.04, 1.03)	0.45 (0.08, 2.48)	0.2 (0.02, 1.59)	0.68 (0.11, 3.69)	0.77 (0.18, 2.84)	0.25 (0.02, 3.36)	0.58 (0.09, 3.45)	0.49 (0.09, 2.46)	0.28 (0.05, 1.56)	0.38 (0.07, 1.9)	0.41 (0.07, 2.24)

														362302226e+47)			4.07)											
0.5 (0.11, 2.24)	1750616556120.09 (15.48, 1.63363081961476e+30)	0.44 (0.12, 1.57)	0.25 (0.06, 0.95)	1.22 (0.31, 4.69)	0.54 (0.16, 1.84)	0.53 (0.15, 1.73)	1.21 (0.13, 34.23)	0.8 (0.23, 2.72)	0.66 (0.21, 2.01)	0.58 (0.12, 2.57)	0.92 (0.23, 3.73)	1.75 (0.47, 6.32)	0.55 (0.18, 1.65)	1.09483120947456e+23 (0.95, 3.81461338522542e+47)	1.26 (0.19, 9.74)	Nitrazepam	0 (0, 2217001693563.78)	0.26 (0.08, 0.79)	0.58 (0.16, 1.92)	0.25 (0.04, 1.45)	0.88 (0.24, 2.88)	0.99 (0.24, 3.86)	0.32 (0.05, 3.16)	0.75 (0.19, 2.7)	0.62 (0.23, 1.59)	0.36 (0.1, 1.23)	0.49 (0.15, 1.45)	0.53 (0.15, 1.74)
410.73 (0, 1.17615572868858e+32)	18906878178276524 (11315.18, 2.74265338635245e+42)	379.52 (0, 1.03205647941949e+32)	215.58 (0, 5.88123424973464e+31)	984.19 (0, 3.01090996128157e+32)	466.09 (0, 1.33458492059612e+32)	449.63 (0, 1.24044756753239e+32)	1167.47 (0, 4.23633992497377e+32)	669.69 (0, 1.95073869703116e+32)	556.1 (0, 1.61746488607354e+32)	501.93 (0, 1.34221388009188e+32)	798.57 (0, 2.31652920581661e+32)	1401.32 (0, 4.36405391701194e+32)	463.28 (0, 1.27762049905391e+32)	1.33607069667244e+23 (0, 1.07727771370221e+63)	1071.35 (0, 2.91399708352277e+32)	821.66 (0, 2.43883551458918e+32)	Paroxetine	223.25 (0, 6.14952149746748e+31)	469.88 (0, 1.34410351212312e+32)	225.65 (0, 5.61596641778631e+31)	725.08 (0, 2.09428478335895e+32)	780.64 (0, 2.277196190099e+32)	273 (0, 6.10098982296592e+31)	633.4 (0, 1.73756325753629e+32)	519.25 (0, 1.48736270457195e+32)	292.01 (0, 8.32046877583044e+31)	420.24 (0, 1.14811309485224e+32)	459.44 (0, 1.26326319667577e+32)
1.9 (0.68, 5.36)	7182807636557.39 (60.19, 4.61049090200508e+30)	1.68 (0.9, 3.25)	0.96 (0.5, 1.9)	4.63 (2.38, 9.82)	2.08 (1.14, 3.86)	2 (1.26, 3.19)	4.52 (0.69, 109.55)	3.04 (1.18, 8.27)	2.52 (1.62, 3.97)	2.16 (0.83, 6.27)	3.46 (1.65, 8.59)	6.57 (3.55, 13.43)	2.09 (0.95, 4.8)	3.98854366204195e+23 (3.76, 1.78362365758096e+48)	4.91 (0.97, 27.65)	3.8 (1.27, 12.51)	0 (0, 10339326843726.3)	Placebo	2.19 (1.43, 3.56)	0.94 (0.25, 3.74)	3.32 (2.11, 5.31)	3.77 (1.39, 9.78)	1.21 (0.26, 9.17)	2.86 (1.38, 5.81)	2.35 (1.3, 4.45)	1.36 (0.76, 2.49)	1.85 (1.36, 2.5)	2.02 (1.11, 3.66)
0.87 (0.27, 2.67)	3251495612965.64 (26.99, 1.97441193629954e+30)	0.77 (0.34, 1.69)	0.44 (0.19, 0.97)	2.12 (0.91, 4.99)	0.95 (0.43, 2)	0.91 (0.46, 1.7)	2.08 (0.3, 51.4)	1.39 (0.47, 4.11)	1.15 (0.59, 2.16)	0.99 (0.34, 3.07)	1.59 (0.66, 4.29)	3.02 (1.36, 6.87)	0.96 (0.37, 2.41)	2.16908294062299e+23 (1.71, 8.32445378913955e+47)	2.24 (0.4, 13.18)	1.73 (0.52, 6.17)	0 (0, 5111461239595.08)	0.46 (0.28, 0.7)	Ramelteon	0.43 (0.1, 1.82)	1.52 (0.77, 2.82)	1.7 (0.57, 4.98)	0.55 (0.11, 4.38)	1.3 (0.54, 2.99)	1.07 (0.5, 2.31)	0.62 (0.29, 1.3)	0.84 (0.47, 1.43)	0.92 (0.42, 1.9)
2.01 (0.36, 10.45)	6832724659373.55 (55.95, 4.41543552612171e+30)	1.78 (0.39, 7.72)	1.01 (0.22, 4.49)	4.99 (1.04, 22.61)	2.2 (0.49, 9.58)	2.11 (0.49, 8.86)	5.02 (0.44, 142.17)	3.23 (0.61, 16.59)	2.65 (0.63, 10.85)	2.32 (0.42, 12.38)	3.72 (0.78, 18.47)	6.98 (1.55, 31.47)	2.22 (0.46, 10.71)	6.05801784980634e+23 (3.75, 1.85704124543604e+48)	5.11 (0.63, 47.23)	4 (0.69, 23.54)	0 (0, 10370372016276.7)	1.06 (0.27, 4.06)	2.32 (0.55, 9.71)	Seltorexant	3.53 (0.82, 14.55)	3.97 (0.75, 20.06)	1.32 (0.16, 13.78)	3.02 (0.64, 13.27)	2.49 (0.56, 10.9)	1.45 (0.33, 6.18)	1.95 (0.5, 7.41)	2.12 (0.49, 9.08)
0.57 (0.19, 1.76)	2135845887308.62 (18.59, 1.50050563587637e+30)	0.51 (0.22, 1.15)	0.29 (0.13, 0.64)	1.39 (0.62, 3.33)	0.63 (0.29, 1.34)	0.6 (0.31, 1.14)	1.37 (0.2, 33.78)	0.92 (0.32, 2.7)	0.76 (0.4, 1.47)	0.66 (0.23, 2.02)	1.05 (0.43, 2.85)	1.99 (0.91, 4.59)	0.63 (0.25, 1.64)	1.16542820578151e+23 (1.14, 5.10723621111225e+47)	1.47 (0.27, 8.96)	1.14 (0.35, 4.12)	0 (0, 2925166581617.66)	0.3 (0.19, 0.47)	0.66 (0.35, 1.3)	0.28 (0.07, 1.22)	Suvorexant	1.13 (0.38, 3.24)	0.36 (0.07, 2.87)	0.86 (0.36, 1.97)	0.71 (0.34, 1.56)	0.41 (0.19, 0.87)	0.56 (0.32, 0.95)	0.61 (0.28, 1.29)
0.51 (0.13, 2.09)	2060054612979.78 (14.85, 1.36841147903609e+30)	0.45 (0.14, 1.44)	0.25 (0.08, 0.84)	1.24 (0.39, 4.3)	0.55 (0.19, 1.71)	0.53 (0.18, 1.59)	1.23 (0.14, 32.26)	0.81 (0.24, 2.82)	0.67 (0.25, 1.9)	0.58 (0.15, 2.4)	0.93 (0.27, 3.58)	1.77 (0.57, 5.8)	0.56 (0.19, 1.71)	1.07767496190168e+23 (0.95, 5.48600083174742e+47)	1.29 (0.35, 5.49)	1.01 (0.26, 4.14)	0 (0, 2674371152563.53)	0.26 (0.1, 0.72)	0.59 (0.2, 1.76)	0.25 (0.05, 1.34)	0.88 (0.31, 2.6)	Temazepam	0.33 (0.05, 2.98)	0.76 (0.23, 2.53)	0.62 (0.24, 1.7)	0.36 (0.12, 1.1)	0.49 (0.19, 1.32)	0.54 (0.18, 1.61)
1.54 (0.16, 10.22)	6594868423139.25 (41.79, 3.87306608295892e+30)	1.37 (0.17, 7.55)	0.78 (0.09, 4.21)	3.81 (0.46, 21.68)	1.7 (0.21, 9.02)	1.65 (0.21, 8.26)	3.86 (0.24, 123.78)	2.51 (0.26, 15.99)	2.06 (0.26, 10.28)	1.81 (0.19, 11.81)	2.92 (0.33, 17.34)	5.47 (0.67, 29.98)	1.74 (0.2, 10.2)	4.19067665087043e+23 (4.03, 1.46881	3.94 (0.3, 43.87)	3.15 (0.32, 21.89)	0 (0, 835447851922	0.83 (0.11, 3.82)	1.81 (0.23, 9.28)	0.76 (0.07, 6.34)	2.75 (0.35, 13.91)	3.06 (0.34, 19.74)	Tiagabine	2.31 (0.28, 12.98)	1.95 (0.23, 10.47)	1.11 (0.14, 5.85)	1.53 (0.2, 7.21)	1.66 (0.2, 8.52)

														334582177e+48)			5.62)											
0.67 (0.19, 2.29)	2525771217185.92 (20.42, 1.90637202738937e+30)	0.59 (0.23, 1.53)	0.33 (0.13, 0.9)	1.63 (0.61, 4.66)	0.73 (0.29, 1.87)	0.7 (0.3, 1.65)	1.62 (0.21, 41.38)	1.07 (0.33, 3.56)	0.88 (0.39, 2.04)	0.76 (0.23, 2.64)	1.22 (0.44, 3.93)	2.31 (0.92, 6.28)	0.74 (0.26, 2.17)	1.56193081760076e+23 (1.35, 6.48651690526029e+47)	1.73 (0.29, 11.26)	1.33 (0.37, 5.23)	0 (0, 3522295433912.55)	0.35 (0.17, 0.73)	0.77 (0.33, 1.86)	0.33 (0.08, 1.55)	1.16 (0.51, 2.77)	1.31 (0.4, 4.27)	0.43 (0.08, 3.59)	Trazodone	0.83 (0.34, 2.1)	0.48 (0.2, 1.18)	0.65 (0.32, 1.32)	0.71 (0.29, 1.75)
0.8 (0.25, 2.62)	3084469038097.76 (24.57, 2.31974875959301e+30)	0.71 (0.3, 1.68)	0.4 (0.17, 1.02)	1.98 (0.78, 5.11)	0.88 (0.4, 1.92)	0.86 (0.39, 1.78)	1.9 (0.27, 49.79)	1.3 (0.62, 2.74)	1.07 (0.57, 1.97)	0.92 (0.29, 3.06)	1.49 (0.57, 4.31)	2.81 (1.19, 6.96)	0.89 (0.51, 1.55)	1.6673551527288e+23 (1.58, 7.64507500349117e+47)	2.05 (0.41, 11.63)	1.62 (0.63, 4.36)	0 (0, 3703080733427.46)	0.43 (0.22, 0.77)	0.93 (0.43, 2.01)	0.4 (0.09, 1.78)	1.41 (0.64, 2.98)	1.6 (0.59, 4.11)	0.51 (0.1, 4.27)	1.21 (0.48, 2.96)	Triazolam	0.58 (0.27, 1.26)	0.79 (0.42, 1.41)	0.86 (0.39, 1.83)
1.41 (0.43, 4.42)	5405098993521.11 (42.84, 3.53811986249248e+30)	1.23 (0.53, 2.98)	0.71 (0.29, 1.71)	3.41 (1.36, 8.89)	1.53 (0.66, 3.48)	1.47 (0.68, 3.08)	3.37 (0.45, 80.77)	2.24 (0.76, 6.57)	1.85 (0.91, 3.75)	1.6 (0.51, 5.18)	2.56 (0.98, 7.5)	4.85 (2.08, 11.89)	1.55 (0.6, 3.96)	2.9592799575863e+23 (3.3, 1.37601432401911e+48)	3.59 (0.64, 21.19)	2.8 (0.81, 9.85)	0 (0, 6925450467730.18)	0.73 (0.4, 1.32)	1.61 (0.77, 3.46)	0.69 (0.16, 3.02)	2.45 (1.14, 5.14)	2.77 (0.91, 8.05)	0.9 (0.17, 7.18)	2.1 (0.84, 5.11)	1.73 (0.79, 3.77)	Zaleplon	1.36 (0.75, 2.4)	1.49 (0.66, 3.25)
1.02 (0.37, 2.89)	3875043779037.69 (31.64, 2.73331067894276e+30)	0.9 (0.47, 1.83)	0.52 (0.25, 1.09)	2.5 (1.2, 5.67)	1.12 (0.59, 2.18)	1.08 (0.63, 1.88)	2.45 (0.36, 60.31)	1.64 (0.64, 4.39)	1.36 (0.85, 2.22)	1.17 (0.45, 3.36)	1.88 (0.85, 4.95)	3.56 (1.86, 7.46)	1.13 (0.52, 2.58)	2.15534467050394e+23 (2.15, 9.53547999897159e+47)	2.64 (0.53, 15.2)	2.05 (0.69, 6.77)	0 (0, 5294693342520.04)	0.54 (0.4, 0.73)	1.19 (0.7, 2.12)	0.51 (0.13, 2.02)	1.8 (1.06, 3.13)	2.04 (0.76, 5.23)	0.66 (0.14, 5.09)	1.54 (0.76, 3.12)	1.27 (0.71, 2.37)	0.73 (0.42, 1.33)	Zolpidem	1.09 (0.61, 1.95)
0.94 (0.29, 3.08)	3650759387930.18 (28.92, 2.82356078535331e+30)	0.83 (0.36, 1.99)	0.47 (0.2, 1.16)	2.29 (0.93, 6.03)	1.03 (0.48, 2.27)	0.99 (0.47, 2.1)	2.29 (0.31, 56.99)	1.51 (0.53, 4.44)	1.25 (0.68, 2.33)	1.08 (0.36, 3.55)	1.73 (0.67, 5.17)	3.29 (1.41, 8.16)	1.04 (0.42, 2.68)	2.15719720011552e+23 (1.94, 8.99334972388228e+47)	2.44 (0.45, 14.64)	1.9 (0.58, 6.76)	0 (0, 5187558540311.55)	0.5 (0.27, 0.9)	1.09 (0.53, 2.36)	0.47 (0.11, 2.04)	1.65 (0.77, 3.52)	1.85 (0.62, 5.51)	0.6 (0.12, 4.96)	1.41 (0.57, 3.47)	1.16 (0.55, 2.56)	0.67 (0.31, 1.52)	0.92 (0.51, 1.64)	Zopiclone

League table for dizziness

Almorexant	2.21 (0.38, 15.49)	0.69 (0.19, 2.42)	0.7 (0.12, 4.32)	1.77 (0.45, 7.75)	0.68 (0.06, 6.13)	1.94 (0.58, 6.67)	18457834556998.1 (15.65, 3.41930635328851e+75)	0.9 (0.2, 4.13)	1.11 (0.22, 5.62)	2.09 (0.69, 6.63)	1.4 (0.41, 5.09)	0.35 (0.09, 1.39)	296855697.22 (0.91, 5.39830080422773e+28)	1651503817727288 (1.67, 2.57420327061798e+45)	0.24 (0.01, 3.21)	2 (0.27, 17.14)	0.37 (0.03, 3.64)	0.6 (0.22, 1.67)	0.79 (0.26, 2.67)	0 (0, 0.04)	0.63 (0.19, 2.16)	0.23 (0.02, 1.72)	2.72 (0.51, 24.84)	1.59 (0.41, 6.58)	4492470002.36 (16.4, 1.08028640027194e+30)	1.95 (0.44, 9.34)	1.41 (0.52, 3.92)	1.42 (0.41, 5.28)
0.45 (0.06, 2.61)	Alprazolam	0.31 (0.05, 1.56)	0.32 (0.03, 2.51)	0.8 (0.12, 4.75)	0.31 (0.02, 3.4)	0.89 (0.15, 4.25)	8177047622058.17 (6.86, 1.32845420197881e+75)	0.4 (0.05, 2.62)	0.5 (0.06, 3.49)	0.95 (0.16, 4.42)	0.63 (0.1, 3.24)	0.15 (0.02, 0.89)	135918946.31 (0.36, 2.33310632177353e+28)	857738858992623 (0.72, 1.27030079943296e+45)	0.11 (0, 1.76)	0.88 (0.08, 9.94)	0.17 (0.02, 0.83)	0.28 (0.05, 1.14)	0.36 (0.06, 1.75)	0 (0, 0.02)	0.29 (0.05, 1.4)	0.1 (0.01, 1.01)	1.24 (0.14, 13.85)	0.72 (0.1, 4.27)	2045172979.93 (6.92, 4.99404912487597e+29)	0.89 (0.11, 6)	0.65 (0.12, 2.83)	0.64 (0.1, 3.44)
1.45 (0.41, 5.17)	3.2 (0.64, 20.3)	Daridorexant	1.01 (0.19, 5.61)	2.54 (0.77, 9.8)	0.99 (0.09, 7.98)	2.82 (1, 8)	26107823586322.3 (23.25, 5.24860815246648e+75)	1.31 (0.33, 5.32)	1.63 (0.35, 7.24)	3.04 (1.2, 8.11)	2.03 (0.7, 6.24)	0.5 (0.14, 1.77)	433863219.61 (1.45, 7.47964283197334e+28)	2557252540937738 (2.43, 4.03453280538047e+45)	0.35 (0.02, 4.36)	2.89 (0.43, 22.42)	0.53 (0.05, 4.8)	0.88 (0.4, 1.91)	1.15 (0.45, 3.21)	0 (0, 0.05)	0.92 (0.33, 2.62)	0.32 (0.03, 2.27)	3.91 (0.83, 33.35)	2.32 (0.68, 8.25)	6561286961.61 (25.53, 1.59268311177357e+30)	2.85 (0.71, 12.39)	2.06 (0.89, 4.88)	2.05 (0.7, 6.59)
1.42 (0.23, 8.67)	3.15 (0.4, 29.28)	0.99 (0.18, 5.27)	Doxepin	2.53 (0.44, 15.78)	0.97 (0.06, 11.03)	2.77 (0.53, 14.38)	26737506763266.1 (17.68, 5.29249426519981e+75)	1.28 (0.2, 8.65)	1.61 (0.21, 11.65)	2.99 (0.61, 15.07)	2 (0.38, 10.91)	0.49 (0.08, 2.96)	425060121.56 (1.3, 6.3447827683698e+28)	2475962024477702 (2.29, 4.6834863888519e+45)	0.34 (0.02, 5.82)	2.83 (0.27, 32.36)	0.51 (0.04, 6.89)	0.86 (0.19, 3.84)	1.13 (0.23, 5.82)	0 (0, 0.05)	0.9 (0.18, 4.66)	0.32 (0.02, 3.31)	3.89 (0.51, 45.94)	2.28 (0.37, 14.29)	6253354757.49 (24.09, 1.30370304929005e+30)	2.77 (0.42, 19.32)	2.02 (0.43, 9.62)	2.01 (0.38, 11.74)
0.56 (0.13, 2.2)	1.26 (0.21, 8.62)	0.39 (0.1, 1.3)	0.39 (0.06, 2.28)	Esmirtazapine	0.38 (0.03, 3.21)	1.1 (0.31, 3.56)	10221612244038 (8.44, 2.07893142068936e+75)	0.51 (0.11, 2.27)	0.63 (0.12, 3.1)	1.19 (0.36, 3.52)	0.8 (0.22, 2.68)	0.19 (0.05, 0.76)	176324985.19 (0.5, 2.73804923587698e+28)	999713630461844 (0.88, 1.48677932277993e+45)	0.14 (0.01, 1.77)	1.11 (0.15, 9.51)	0.21 (0.02, 2.02)	0.35 (0.12, 0.86)	0.46 (0.14, 1.38)	0 (0, 0.02)	0.36 (0.1, 1.13)	0.13 (0.01, 0.95)	1.54 (0.27, 13.32)	0.91 (0.21, 3.63)	2574278368.09 (9.26, 6.22573341317275e+29)	1.11 (0.23, 5.27)	0.81 (0.26, 2.21)	0.8 (0.22, 2.85)
1.48 (0.16, 18.05)	3.27 (0.29, 56.1)	1.01 (0.13, 11.55)	1.03 (0.09, 15.57)	2.6 (0.31, 32.67)	Estazolam	2.86 (0.38, 30.7)	29569298556356.8 (20.86, 5.91673655337 [illegible]	1.32 (0.15, 16.28)	1.64 (0.28, 14.5)	3.06 (0.43, 32.83)	2.07 (0.27, 23.48)	0.5 (0.06, 5.98)	415862250.18 (1.35, 9.6578957337798e+28)	3755053055387512 (2.61, 4.25089990922 [illegible]	0.36 (0.01, 9.46)	2.99 (0.23, 54.03)	0.55 (0.03, 11.88)	0.89 (0.13, 8.83)	1.17 (0.16, 12.86)	0 (0, 0.06)	0.93 (0.12, 10.18)	0.33 (0.02, 5.91)	4.18 (0.36, 83.06)	2.34 (0.31, 25.8)	6428932764.86 (23.63, 2.20271372492 [illegible]	2.93 (0.29, 38.66)	2.09 (0.3, 21.16)	2.1 (0.27, 23.77)

							965e +75)							388e +45)											279e +30)			
0.52 (0.15, 1.72)	1.13 (0.24, 6.89)	0.35 (0.13, 1)	0.36 (0.07, 1.88)	0.91 (0.28, 3.28)	0.35 (0.03, 2.66)	Eszopiclone	9054067042019.88 (8.19, 2.04804434772269e+75)	0.46 (0.14, 1.61)	0.58 (0.13, 2.44)	1.08 (0.45, 2.67)	0.72 (0.26, 2.09)	0.18 (0.05, 0.6)	156388053.75 (0.51, 2.73177913869303e+28)	910095026158456 (0.86, 1.37512927764929e+45)	0.12 (0.01, 1.49)	1.01 (0.18, 7.09)	0.19 (0.02, 1.66)	0.31 (0.15, 0.62)	0.41 (0.17, 1.06)	0 (0, 0.02)	0.33 (0.12, 0.86)	0.12 (0.01, 0.78)	1.38 (0.3, 11.11)	0.82 (0.26, 2.66)	2199723418.68 (8.93, 5.59638772423061e+29)	1.01 (0.25, 4.07)	0.73 (0.34, 1.57)	0.73 (0.34, 1.73)
0 (0, 0.06)	0 (0, 0.15)	0 (0, 0.04)	0 (0, 0.06)	0 (0, 0.12)	0 (0, 0.05)	0 (0, 0.12)	EVT_201	0 (0, 0.06)	0 (0, 0.07)	0 (0, 0.13)	0 (0, 0.09)	0 (0, 0.02)	0 (0, 6.21239339829398e+20)	5.38 (0, 1.1872679147631e+30)	0 (0, 0.02)	0 (0, 0.15)	0 (0, 0.02)	0 (0, 0.04)	0 (0, 0.05)	0 (0, 0)	0 (0, 0.04)	0 (0, 0.01)	0 (0, 0.2)	0 (0, 0.1)	0 (0, 1.51796201839387e+22)	0 (0, 0.12)	0 (0, 0.09)	0 (0, 0.09)
1.11 (0.24, 4.91)	2.47 (0.38, 18.5)	0.77 (0.19, 3.01)	0.78 (0.12, 5.05)	1.95 (0.44, 9.38)	0.75 (0.06, 6.52)	2.17 (0.62, 7.23)	19841209544287 (17.27, 3.60616371065339e+75)	Flunitrazepam	1.24 (0.24, 6.01)	2.32 (0.66, 8.29)	1.55 (0.39, 6.26)	0.38 (0.08, 1.69)	342013819.33 (1, 5.41085565543486e+28)	2087171825066922 (1.74, 2.86864571374539e+45)	0.27 (0.01, 3.72)	2.18 (0.34, 17.04)	0.41 (0.03, 4.27)	0.67 (0.21, 2.13)	0.89 (0.25, 3.29)	0 (0, 0.04)	0.7 (0.18, 2.69)	0.25 (0.02, 2.1)	3.01 (0.5, 28.58)	1.76 (0.62, 5.03)	4781351160.22 (18.15, 1.34815553813101e+30)	2.15 (0.48, 10.19)	1.57 (0.5, 4.98)	1.57 (0.56, 4.71)
0.9 (0.18, 4.58)	1.98 (0.29, 16.63)	0.61 (0.14, 2.86)	0.62 (0.09, 4.77)	1.58 (0.32, 8.64)	0.61 (0.07, 3.55)	1.73 (0.41, 7.62)	15279781077465.2 (13.59, 3.65271359929706e+75)	0.81 (0.17, 4.21)	Flurazepam	1.87 (0.48, 7.88)	1.25 (0.29, 5.82)	0.31 (0.06, 1.59)	270668203.82 (0.85, 4.78924486877271e+28)	1546475106817794 (1.48, 2.46508639462485e+45)	0.21 (0.01, 3.31)	1.75 (0.23, 16.73)	0.32 (0.03, 3.8)	0.54 (0.15, 2.07)	0.71 (0.18, 3.13)	0 (0, 0.03)	0.56 (0.13, 2.54)	0.2 (0.02, 1.85)	2.45 (0.37, 25.58)	1.43 (0.36, 6.11)	3862552622 (15.61, 1.09868838520258e+30)	1.76 (0.32, 10.2)	1.26 (0.35, 4.84)	1.27 (0.3, 5.88)
0.48 (0.15, 1.45)	1.05 (0.23, 6.17)	0.33 (0.12, 0.84)	0.33 (0.07, 1.64)	0.84 (0.28, 2.79)	0.33 (0.03, 2.35)	0.93 (0.37, 2.21)	8456864933792.39 (7.74, 1.87333348198159e+75)	0.43 (0.12, 1.52)	0.53 (0.13, 2.09)	Gaboxadol	0.67 (0.26, 1.74)	0.17 (0.05, 0.5)	146524607.35 (0.47, 2.34696479471736e+28)	844992587236612 (0.8, 1.29104157726132e+45)	0.11 (0.01, 1.33)	0.94 (0.15, 6.88)	0.18 (0.02, 1.52)	0.29 (0.16, 0.5)	0.38 (0.17, 0.88)	0 (0, 0.02)	0.3 (0.12, 0.73)	0.11 (0.01, 0.68)	1.28 (0.3, 9.86)	0.76 (0.25, 2.4)	2156024715.05 (8.37, 5.20869914943334e+29)	0.93 (0.26, 3.51)	0.68 (0.36, 1.25)	0.68 (0.26, 1.84)
0.72 (0.2, 2.45)	1.58 (0.31, 9.71)	0.49 (0.16, 1.43)	0.5 (0.09, 2.65)	1.25 (0.37, 4.59)	0.48 (0.04, 3.76)	1.38 (0.48, 3.85)	12471068463006.9 (11.5, 2.70093430720046e+75)	0.65 (0.16, 2.55)	0.8 (0.17, 3.51)	1.5 (0.58, 3.81)	Indiplon	0.25 (0.07, 0.84)	209024884.34 (0.69, 3.27309764555461e+28)	1280229642255324 (1.14, 1.87026232780778e+45)	0.17 (0.01, 2.1)	1.42 (0.21, 10.82)	0.26 (0.03, 2.34)	0.44 (0.19, 0.9)	0.57 (0.22, 1.5)	0 (0, 0.02)	0.45 (0.16, 1.23)	0.16 (0.02, 1.09)	1.92 (0.41, 15.63)	1.14 (0.32, 4.06)	3108746002.51 (12.3, 7.4163530056909e+29)	1.4 (0.33, 5.97)	1.02 (0.42, 2.36)	1.01 (0.33, 3.17)
2.88 (0.72,	6.5 (1.12,	1.99 (0.56, 6.95)	2.04 (0.34,	5.13 (1.32,	1.98 (0.17,	5.65 (1.68, 18.7)	508209670075	2.61 (0.59,	3.26 (0.63,	6.05 (2,	4.06 (1.2,	Lemborexant	903072672.77	515273938069	0.69 (0.04, 9.39)	5.74 (0.79,	1.07 (0.09,	1.76 (0.65, 4.75)	2.31 (0.75, 7.5)	0 (0, 0.1)	1.83 (0.56, 6.13)	0.66 (0.06, 5.02)	7.82 (1.46,	4.66 (1.19,	13132938509.0	5.68 (1.25,	4.11 (1.53,	4.13 (1.2,

11.52)	43.46)		12.13)	21.81)	17.25)		55.1 (44.43, 1.07823197100366e+76)	12.14)	16.13)	19.35)	14.52)		(2.67, 1.37370246153799e+29)	4799 (4.85, 7.53436197459677e+45)		48.54)	10.29)						69.12)	19.12)	4 (47.14, 2.94377962209424e+30)	26.85)	11.83)	15.11)
0 (0, 1.1)	0 (0, 2.77)	0 (0, 0.69)	0 (0, 0.77)	0 (0, 1.98)	0 (0, 0.74)	0 (0, 1.96)	242124.48 (0, 4.41696683097078e+69)	0 (0, 1)	0 (0, 1.18)	0 (0, 2.14)	0 (0, 1.44)	0 (0, 0.37)	Lometazepam	4329246.61 (0, 4.6600472192414e+38)	0 (0, 0.29)	0 (0, 2.39)	0 (0, 0.53)	0 (0, 0.61)	0 (0, 0.83)	0 (0, 0)	0 (0, 0.62)	0 (0, 0.23)	0 (0, 3.3)	0 (0, 1.61)	13.67 (1.87, 371.36)	0 (0, 2.11)	0 (0, 1.43)	0 (0, 1.45)
0 (0, 0.6)	0 (0, 1.38)	0 (0, 0.41)	0 (0, 0.44)	0 (0, 1.14)	0 (0, 0.38)	0 (0, 1.16)	0.19 (0, 1.94215508195264e+73)	0 (0, 0.58)	0 (0, 0.68)	0 (0, 1.25)	0 (0, 0.88)	0 (0, 0.21)	0 (0, 7.57706108029682e+22)	Loprazolam	0 (0, 0.17)	0 (0, 1.26)	0 (0, 0.26)	0 (0, 0.36)	0 (0, 0.49)	0 (0, 0)	0 (0, 0.38)	0 (0, 0.14)	0 (0, 1.92)	0 (0, 1)	0 (0, 1.25831861653197e+24)	0 (0, 1.26)	0 (0, 0.85)	0 (0, 0.89)
4.13 (0.31, 73.45)	9.37 (0.57, 217.93)	2.89 (0.23, 45.78)	2.96 (0.17, 59.8)	7.36 (0.56, 130.76)	2.79 (0.11, 77.03)	8.18 (0.67, 128.82)	73254238519811.6 (57.16, 1.33588703601963e+76)	3.77 (0.27, 71.57)	4.72 (0.3, 89.67)	8.7 (0.75, 136.13)	5.83 (0.48, 97.93)	1.45 (0.11, 24.92)	1179948672.91 (3.49, 2.84425741379439e+29)	9666742585368788 (5.83, 1.5613588637552e+46)	Mldazolam	8.41 (0.41, 227.9)	1.53 (0.06, 47.87)	2.52 (0.23, 37.02)	3.35 (0.29, 53.15)	0 (0, 0.21)	2.63 (0.22, 42.59)	0.93 (0.18, 4.82)	11.94 (0.71, 323.8)	6.66 (0.51, 119.48)	17664907204.68 (65.77, 5.60152197620506e+30)	8.18 (0.57, 157.82)	5.88 (0.53, 89.65)	5.94 (0.48, 101.94)
0.5 (0.06, 3.69)	1.13 (0.1, 12.47)	0.35 (0.04, 2.34)	0.35 (0.03, 3.67)	0.9 (0.11, 6.87)	0.33 (0.02, 4.31)	0.99 (0.14, 5.67)	9898102283114.16 (6.81, 1.32309729848764e+75)	0.46 (0.06, 2.94)	0.57 (0.06, 4.4)	1.06 (0.15, 6.54)	0.7 (0.09, 4.83)	0.17 (0.02, 1.26)	152715371.88 (0.42, 1.65976478710933e+28)	76991718698427 (0.79, 1.37226420045339e+45)	0.12 (0, 2.43)	Nitrazepam	0.18 (0.01, 2.64)	0.31 (0.05, 1.77)	0.4 (0.06, 2.57)	0 (0, 0.02)	0.32 (0.04, 2.06)	0.11 (0.01, 1.39)	1.38 (0.13, 17.94)	0.82 (0.11, 4.99)	212541571.73 (7.26, 3.82907530875814e+29)	0.98 (0.11, 7.67)	0.72 (0.1, 4.09)	0.72 (0.12, 3.66)
2.73 (0.27, 30.5)	5.96 (1.2, 47.48)	1.88 (0.21, 19.36)	1.96 (0.15, 26.6)	4.78 (0.49, 54.18)	1.83 (0.08, 35.61)	5.27 (0.6, 53.96)	55270521379517.7 (41.65, 8.84393542229521e+75)	2.45 (0.23, 29.87)	3.09 (0.26, 38.49)	5.71 (0.66, 54.9)	3.81 (0.43, 37.78)	0.94 (0.1, 10.72)	813335907.92 (1.9, 1.05961175212887e+29)	4902911231337239 (3.86, 8.61360648727672e+45)	0.65 (0.02, 17.15)	5.47 (0.38, 99.08)	Paroxetine	1.65 (0.21, 14.82)	2.17 (0.25, 21.32)	0 (0, 0.11)	1.73 (0.19, 17.57)	0.6 (0.03, 10.57)	7.82 (0.6, 136.83)	4.36 (0.44, 50.73)	12807001640.67 (38.63, 2.429260618527e+30)	5.34 (0.49, 68.15)	3.86 (0.47, 36.34)	3.85 (0.42, 42.08)
1.65 (0.6, 4.56)	3.6 (0.88, 19.62)	1.13 (0.52, 2.47)	1.16 (0.26, 5.17)	2.87 (1.16, 8.49)	1.13 (0.11, 7.58)	3.18 (1.61, 6.5)	28239183952241.9 (26.36, 6.37692689420	1.48 (0.47, 4.77)	1.84 (0.48, 6.63)	3.44 (2.01, 6.26)	2.3 (1.12, 5.13)	0.57 (0.21, 1.53)	502620917.52 (1.65, 8.97800490309223e+28)	2917483307586088 (2.79, 4.38072994522198e+45)	0.4 (0.03, 4.34)	3.25 (0.56, 22.19)	0.61 (0.07, 4.88)	Placebo	1.3 (0.76, 2.48)	0 (0, 0.06)	1.04 (0.54, 2.08)	0.37 (0.04, 2.18)	4.41 (1.16, 31.75)	2.64 (0.98, 7.42)	7280538949.57 (28.84, 1.85825303442935e+30)	3.21 (0.99, 11.2)	2.33 (1.57, 3.54)	2.33 (1.06, 5.6)

							633e+75)																					
1.27 (0.37, 3.92)	2.74 (0.57, 16.36)	0.87 (0.31, 2.22)	0.88 (0.17, 4.32)	2.2 (0.72, 7.19)	0.85 (0.08, 6.11)	2.44 (0.94, 5.94)	2190019121663 2.7 (19.79, 4.92479306908025e+75)	1.12 (0.3, 4.06)	1.41 (0.32, 5.63)	2.63 (1.13, 5.8)	1.76 (0.67, 4.52)	0.43 (0.13, 1.33)	375548849.49 (1.2, 6.13758724609518e+28)	2291004878761241 (2.02, 3.31973026659268e+45)	0.3 (0.02, 3.48)	2.48 (0.39, 18)	0.46 (0.05, 4)	0.77 (0.4, 1.32)	Ramelteon	0 (0, 0.04)	0.8 (0.32, 1.89)	0.28 (0.03, 1.76)	3.37 (0.76, 25.9)	2.01 (0.62, 6.39)	5647417317.43 (21.34, 1.40429048450857e+30)	2.46 (0.63, 9.37)	1.79 (0.85, 3.52)	1.77 (0.65, 4.89)
196836811632.88 (27.96, 9.0903736788028e+29)	439465897921.5 (62.83, 1.79926381570494e+30)	131155994279.31 (19.53, 5.79055535300582e+29)	147361317466.55 (18.54, 6.47865715409165e+29)	365535339445.07 (51.33, 1.5195688468644e+30)	134171622350.29 (17.54, 7.24488236512887e+29)	393329506296.44 (55.8, 1.62665126550851e+30)	3.19386132775922e+27 (2694124.85, 1.69198357491871e+92)	197904304186.72 (24.65, 8.23841376017509e+29)	235917063804.49 (30.84, 1.06085361555754e+30)	405760128049.31 (61.05, 1.76692974444328e+30)	278629894091.63 (40.1, 1.24867565887913e+30)	67943488874.79 (9.61, 2.86722996909185e+29)	1.57023084994798e+22 (14458.36, 2.52026186782776e+42)	4.12149382687661e+29 (99077.5, 7.13033475998197e+63)	47849275140.36 (4.78, 1.97055102830699e+29)	408969849791.35 (45.75, 1.82345024352189e+30)	72474477471.55 (8.84, 3.31466725415578e+29)	118137825991.56 (17.95, 4.84799592331051e+29)	165442944268.65 (23.37, 6.73051611286603e+29)	Seltorexant	125738213570.18 (18.19, 5.07634737808675e+29)	44706332170.04 (5.17, 1.61187190364851e+29)	704208434663.36 (73.25, 2.98064468925053e+30)	339254317312.77 (45.46, 1.37577456738672e+30)	2.17737285934705e+23 (223885.57, 3.82573990822742e+43)	431127360964.79 (49.71, 1.85612231906239e+30)	282333863769.34 (41.37, 1.16052285346984e+30)	293591961230.96 (40.82, 1.25417521473457e+30)
1.58 (0.46, 5.21)	3.47 (0.71, 21.23)	1.08 (0.38, 3.04)	1.11 (0.21, 5.65)	2.78 (0.89, 9.82)	1.07 (0.1, 8.17)	3.06 (1.16, 8.07)	27758868748960.4 (25.31, 6.38312883487796e+75)	1.42 (0.37, 5.45)	1.77 (0.39, 7.54)	3.31 (1.38, 8.04)	2.21 (0.81, 6.28)	0.55 (0.16, 1.79)	469803306.64 (1.61, 7.58519999678785e+28)	2868463712993836 (2.63, 4.10524259419438e+45)	0.38 (0.02, 4.55)	3.11 (0.49, 24.01)	0.58 (0.06, 5.19)	0.96 (0.48, 1.86)	1.26 (0.53, 3.17)	0 (0, 0.05)	Suvorexant	0.36 (0.04, 2.34)	4.27 (0.94, 33.8)	2.53 (0.75, 8.58)	6962189671.98 (27.51, 1.62509467073057e+30)	3.08 (0.8, 12.58)	2.24 (1.02, 4.91)	2.23 (0.8, 6.77)
4.41 (0.58, 48.55)	10.07 (0.99, 149.16)	3.08 (0.44, 29.8)	3.15 (0.3, 42.24)	7.84 (1.06, 87.73)	3.03 (0.17, 54.83)	8.62 (1.28, 84.27)	81998719253229 (72.08, 1.50573699369862e+76)	4.02 (0.48, 46.73)	4.99 (0.54, 61.56)	9.28 (1.47, 89.81)	6.2 (0.92, 63.72)	1.52 (0.2, 16.88)	1346640326.2 (4.3, 2.67843913137826e+29)	8838389369772453 (7.12, 1.4476974682378e+46)	1.08 (0.21, 5.49)	8.94 (0.72, 158.5)	1.66 (0.09, 32.27)	2.67 (0.46, 23.96)	3.53 (0.57, 34.57)	0 (0, 0.19)	2.81 (0.43, 27.69)	Temazepam	12.48 (1.26, 224.53)	7.15 (0.93, 78.47)	18936825544.17 (76, 5.59581537113724e+30)	8.71 (1.04, 104.28)	6.24 (1.04, 59.14)	6.29 (0.92, 67.25)
0.37 (0.04, 1.97)	0.8 (0.07, 7.12)	0.26 (0.03, 1.21)	0.26 (0.02, 1.97)	0.65 (0.08, 3.7)	0.24 (0.01, 2.79)	0.72 (0.09, 3.35)	6386455166013.08 (5.08, 1.44343904431 1e+75)	0.33 (0.03, 1.99)	0.41 (0.04, 2.67)	0.78 (0.1, 3.37)	0.52 (0.06, 2.45)	0.13 (0.01, 0.69)	100666167.9 (0.3, 1.71271385313003e+28)	697100490693452 (0.52, 8.28783591335334e+44)	0.08 (0, 1.4)	0.73 (0.06, 7.64)	0.13 (0.01, 1.66)	0.23 (0.03, 0.87)	0.3 (0.04, 1.31)	0 (0, 0.01)	0.23 (0.03, 1.07)	0.08 (0, 0.79)	Tiagabine	0.59 (0.07, 3.3)	1507649629.95 (5.58, 4.06830155787417e+29)	0.71 (0.07, 4.69)	0.53 (0.07, 2.14)	0.53 (0.06, 2.6)
0.63 (0.15, 2.45)	1.4 (0.23, 9.69)	0.43 (0.12, 1.47)	0.44 (0.07, 2.69)	1.1 (0.28, 4.8)	0.43 (0.04, 3.27)	1.22 (0.38, 3.78)	11485179354038.7 (9.63, 2.226	0.57 (0.2, 1.61)	0.7 (0.16, 2.74)	1.31 (0.42, 4.07)	0.88 (0.25, 3.14)	0.21 (0.05, 0.84)	195572011.18 (0.62, 3.4201607	1049934625724674 (1, 1.587	0.15 (0.01, 1.97)	1.22 (0.2, 9.15)	0.23 (0.02, 2.26)	0.38 (0.13, 1.02)	0.5 (0.16, 1.62)	0 (0, 0.02)	0.4 (0.12, 1.32)	0.14 (0.01, 1.08)	1.69 (0.3, 14.82)	Triazolam	2787859263.38 (11.01, 7.504	1.21 (0.33, 4.88)	0.88 (0.32, 2.42)	0.89 (0.3, 2.69)

							0535 7719 502e +75)						5434 337e +28)	4003 0517 665e +45)											9404 4117 416e +29)			
0 (0, 0.06)	0 (0, 0.14)	0 (0, 0.04)	0 (0, 0.04)	0 (0, 0.11)	0 (0, 0.04)	0 (0, 0.11)	1263 3.2 (0, 4.929 7194 3557 816e +68)	0 (0, 0.06)	0 (0, 0.06)	0 (0, 0.12)	0 (0, 0.08)	0 (0, 0.02)	0.07 (0, 0.53)	2739 10.44 (0, 3.121 4958 0898 023e +37)	0 (0, 0.02)	0 (0, 0.14)	0 (0, 0.03)	0 (0, 0.03)	0 (0, 0.05)	0 (0, 0)	0 (0, 0.04)	0 (0, 0.01)	0 (0, 0.18)	0 (0, 0.09)	Trimi prami ne	0 (0, 0.11)	0 (0, 0.08)	0 (0, 0.08)
0.51 (0.11, 2.27)	1.13 (0.17, 8.78)	0.35 (0.08, 1.41)	0.36 (0.05, 2.41)	0.9 (0.19, 4.36)	0.34 (0.03, 3.43)	0.99 (0.25, 3.95)	8805 5846 9112 3.06 (8.53, 1.876 8651 8047 013e +75)	0.47 (0.1, 2.07)	0.57 (0.1, 3.09)	1.08 (0.28, 3.81)	0.71 (0.17, 3)	0.18 (0.04, 0.8)	1511 4064 9.84 (0.47, 2.406 0713 2858 527e +28)	9760 0564 1824 597 (0.8, 1.323 2164 4427 28e+ 45)	0.12 (0.01, 1.75)	1.02 (0.13, 9.05)	0.19 (0.01, 2.04)	0.31 (0.09, 1.01)	0.41 (0.11, 1.58)	0 (0, 0.02)	0.32 (0.08, 1.25)	0.11 (0.01, 0.96)	1.4 (0.21, 13.42)	0.83 (0.2, 3.03)	2362 9454 34.82 (9.14, 5.118 9686 3910 805e +29)	Zalep lon	0.73 (0.22, 2.32)	0.73 (0.18, 2.92)
0.71 (0.25, 1.91)	1.55 (0.35, 8.68)	0.49 (0.21, 1.12)	0.5 (0.1, 2.32)	1.23 (0.45, 3.89)	0.48 (0.05, 3.31)	1.37 (0.64, 2.93)	1236 2840 4391 67.3 (11.3 8, 2.509 2430 2516 391e +75)	0.64 (0.2, 1.99)	0.79 (0.21, 2.84)	1.48 (0.8, 2.78)	0.98 (0.42, 2.39)	0.24 (0.08, 0.65)	2154 3081 2.97 (0.7, 3.604 3151 3449 213e +28)	1195 3701 1127 0014 (1.17, 1.864 5354 3106 926e +45)	0.17 (0.01, 1.89)	1.39 (0.24, 9.59)	0.26 (0.03, 2.13)	0.43 (0.28, 0.64)	0.56 (0.28, 1.18)	0 (0, 0.02)	0.45 (0.2, 0.98)	0.16 (0.02, 0.97)	1.89 (0.47, 14.17)	1.13 (0.41, 3.08)	3194 0610 43.1 (12.0 5, 7.682 9348 0127 334e +29)	1.37 (0.43, 4.57)	Zolpi dem	1 (0.46, 2.32)
0.7 (0.19, 2.45)	1.55 (0.29, 9.83)	0.49 (0.15, 1.43)	0.5 (0.09, 2.66)	1.24 (0.35, 4.63)	0.48 (0.04, 3.71)	1.38 (0.58, 2.95)	1241 8421 6001 69.9 (10.8, 2.769 6257 0435 204e +75)	0.64 (0.21, 1.78)	0.79 (0.17, 3.32)	1.48 (0.54, 3.8)	0.99 (0.32, 3.03)	0.24 (0.07, 0.83)	2098 9427 0.62 (0.69, 3.487 2201 1888 052e +28)	1255 5322 3611 7923 (1.12, 1.757 4106 8276 595e +45)	0.17 (0.01, 2.07)	1.38 (0.27, 8.37)	0.26 (0.02, 2.37)	0.43 (0.18, 0.95)	0.56 (0.2, 1.53)	0 (0, 0.02)	0.45 (0.15, 1.26)	0.16 (0.01, 1.09)	1.89 (0.38, 15.5)	1.12 (0.37, 3.35)	3013 1451 89.7 (12.1 2, 7.383 8207 0781 838e +29)	1.37 (0.34, 5.48)	1 (0.43, 2.18)	Zopic lone

League table for headache

Almorexant	1.57 (0.9, 2.78)	0.54 (0.26, 1.09)	0.77 (0.41, 1.42)	0.69 (0.26, 1.72)	1.08 (0.67, 1.74)	24982.38 (9.21, 5167307412.58)	0.94 (0.41, 2.21)	1.01 (0.48, 2.13)	1.11 (0.67, 1.88)	1.74 (0.93, 3.38)	1.31 (0.76, 2.27)	0.53 (0.02, 6.56)	0.99 (0.54, 1.82)	20323.15 (1.01, 2566530679235636736)	0.64 (0.25, 1.51)	0.67 (0.15, 3.01)	0 (0, 0.4)	1.06 (0.69, 1.63)	1.03 (0.62, 1.72)	0.88 (0.39, 2.01)	1.31 (0.75, 2.34)	1.56 (0.62, 4.7)	1.1 (0.54, 2.17)	1.44 (0.44, 8.02)	1.64 (0.88, 2.99)	1.5 (0.85, 2.61)	2.34 (0.43, 19.12)	0.95 (0.28, 3.12)	1.03 (0.44, 2.49)	1.33 (0.87, 2.06)	1.01 (0.56, 1.85)
0.64 (0.36, 1.11)	Daridorexant	0.35 (0.16, 0.7)	0.49 (0.27, 0.87)	0.44 (0.18, 1.08)	0.69 (0.45, 1.04)	15949.19 (5.99, 3203483502.24)	0.6 (0.26, 1.37)	0.64 (0.31, 1.32)	0.71 (0.44, 1.1)	1.12 (0.6, 1.95)	0.84 (0.5, 1.38)	0.34 (0.01, 4.11)	0.63 (0.36, 1.11)	10852.56 (0.64, 1498473645720733184)	0.41 (0.15, 0.95)	0.41 (0.1, 1.83)	0 (0, 0.25)	0.68 (0.46, 0.97)	0.65 (0.42, 1.02)	0.56 (0.26, 1.24)	0.84 (0.5, 1.38)	1 (0.4, 2.85)	0.71 (0.35, 1.34)	0.91 (0.29, 5.23)	1.04 (0.58, 1.84)	0.95 (0.57, 1.6)	1.51 (0.28, 11.85)	0.61 (0.18, 1.9)	0.67 (0.28, 1.49)	0.85 (0.57, 1.26)	0.65 (0.37, 1.09)
1.84 (0.91, 3.91)	2.9 (1.43, 6.07)	Doxepin	1.41 (0.66, 2.97)	1.29 (0.46, 3.47)	2 (1.06, 3.72)	46229.55 (17.05, 9587252538.75)	1.76 (0.63, 4.35)	1.89 (0.81, 4.29)	2.06 (1.04, 4.1)	3.25 (1.48, 6.5)	2.46 (1.25, 4.82)	0.94 (0.04, 11.74)	1.82 (0.88, 3.79)	33851.76 (1.94, 4457380511351428096)	1.2 (0.42, 3.16)	1.21 (0.25, 5.64)	0 (0, 0.75)	1.97 (1.08, 3.57)	1.89 (1.01, 3.67)	1.62 (0.62, 4.19)	2.43 (1.21, 4.95)	2.9 (1.04, 8.73)	2.03 (0.92, 4.59)	2.7 (0.72, 16.93)	3.04 (1.44, 6.21)	2.75 (1.38, 5.58)	4.37 (0.77, 36.03)	1.81 (0.46, 5.74)	1.9 (0.79, 5.12)	2.46 (1.33, 4.62)	1.86 (0.92, 3.88)
1.3 (0.71, 2.43)	2.06 (1.15, 3.7)	0.71 (0.34, 1.51)	Esmirtazapine	0.92 (0.33, 2.3)	1.42 (0.84, 2.32)	33028.14 (12.48, 5827899961.71)	1.21 (0.51, 2.94)	1.3 (0.62, 2.93)	1.45 (0.82, 2.5)	2.27 (1.19, 4.37)	1.71 (0.96, 3.12)	0.69 (0.03, 8.81)	1.29 (0.67, 2.5)	23652.11 (1.32, 3138121482976325632)	0.84 (0.3, 2.14)	0.85 (0.19, 3.93)	0 (0, 0.52)	1.38 (0.87, 2.25)	1.35 (0.79, 2.29)	1.14 (0.5, 2.72)	1.72 (0.96, 3.1)	2.03 (0.8, 6.43)	1.45 (0.66, 2.98)	1.91 (0.57, 10.54)	2.12 (1.11, 4.11)	1.95 (1.05, 3.64)	3.04 (0.54, 25.41)	1.24 (0.35, 3.98)	1.37 (0.57, 3.25)	1.74 (1.07, 2.82)	1.32 (0.71, 2.39)
1.44 (0.58, 3.92)	2.27 (0.93, 5.63)	0.77 (0.29, 2.18)	1.09 (0.44, 3.04)	Estazolam	1.55 (0.68, 3.92)	34890.7 (15.2, 7350754738.31)	1.34 (0.46, 4.24)	1.45 (0.65, 3.46)	1.58 (0.68, 4)	2.48 (0.93, 6.86)	1.88 (0.77, 4.99)	0.77 (0.03, 10.3)	1.43 (0.56, 3.73)	24425.3 (1.35, 3117409861341151744)	0.91 (0.27, 2.9)	0.93 (0.19, 5.05)	0 (0, 0.58)	1.52 (0.68, 3.74)	1.47 (0.63, 3.82)	1.27 (0.44, 4.1)	1.88 (0.79, 5.03)	2.22 (0.7, 8.77)	1.58 (0.54, 4.61)	2.19 (0.52, 11.91)	2.34 (0.9, 6.35)	2.15 (0.88, 5.49)	3.37 (0.54, 28.85)	1.35 (0.33, 5.53)	1.51 (0.5, 4.7)	1.9 (0.85, 4.81)	1.43 (0.58, 3.95)
0.92 (0.57, 1.49)	1.44 (0.96, 2.21)	0.5 (0.27, 0.94)	0.71 (0.43, 1.18)	0.65 (0.26, 1.47)	Eszopiclone	22820.8 (8.45, 4238530223.76)	0.87 (0.4, 1.86)	0.93 (0.48, 1.8)	1.02 (0.71, 1.44)	1.6 (0.96, 2.71)	1.22 (0.81, 1.86)	0.5 (0.02, 5.98)	0.91 (0.56, 1.49)	17616.1 (0.96, 2307019619304102912)	0.59 (0.24, 1.29)	0.6 (0.14, 2.64)	0 (0, 0.36)	0.98 (0.79, 1.21)	0.95 (0.67, 1.33)	0.81 (0.39, 1.76)	1.21 (0.81, 1.89)	1.45 (0.6, 3.95)	1.02 (0.55, 1.84)	1.33 (0.43, 7.65)	1.51 (0.89, 2.51)	1.38 (0.89, 2.12)	2.16 (0.42, 17.68)	0.88 (0.27, 2.66)	0.97 (0.42, 2.09)	1.23 (0.94, 1.61)	0.93 (0.63, 1.36)
0 (0, 0.11)	0 (0, 0.17)	0 (0, 0.06)	0 (0, 0.08)	0 (0, 0.07)	0 (0, 0.12)	EVT_201	0 (0, 0.11)	0 (0, 0.12)	0 (0, 0.13)	0 (0, 0.18)	0 (0, 0.14)	0 (0, 0.11)	0 (0, 0.11)	9.66 (0, 1347604839148 9.1)	0 (0, 0.08)	0 (0, 0.08)	0 (0, 0.01)	0 (0, 0.12)	0 (0, 0.11)	0 (0, 0.11)	0 (0, 0.15)	0 (0, 0.17)	0 (0, 0.13)	0 (0, 0.15)	0 (0, 0.19)	0 (0, 0.16)	0 (0, 0.34)	0 (0, 0.13)	0 (0, 0.12)	0 (0, 0.15)	0 (0, 0.12)

1.07 (0.45, 2.47)	1.67 (0.73, 3.87)	0.57 (0.23, 1.59)	0.83 (0.34, 1.97)	0.75 (0.24, 2.18)	1.15 (0.54, 2.52)	25320.78 (9.33, 4759238354.41)	Flunitrazepam	1.07 (0.4, 2.82)	1.18 (0.54, 2.69)	1.85 (0.78, 4.44)	1.39 (0.62, 3.34)	0.58 (0.02, 6.76)	1.05 (0.52, 2.27)	21102.98 (0.96, 2715890878128246272)	0.67 (0.21, 2.07)	0.69 (0.14, 3.5)	0 (0, 0.44)	1.13 (0.54, 2.44)	1.09 (0.5, 2.51)	0.93 (0.32, 2.78)	1.4 (0.61, 3.31)	1.7 (0.54, 5.39)	1.15 (0.5, 2.87)	1.6 (0.38, 9.25)	1.73 (0.72, 4.12)	1.58 (0.81, 3.34)	2.5 (0.43, 19.54)	1.01 (0.25, 3.98)	1.11 (0.41, 3.09)	1.41 (0.66, 3.03)	1.08 (0.48, 2.37)
0.99 (0.47, 2.07)	1.57 (0.76, 3.25)	0.53 (0.23, 1.23)	0.77 (0.34, 1.62)	0.69 (0.29, 1.53)	1.07 (0.56, 2.06)	25022.53 (8.65, 4722954477.82)	0.93 (0.35, 2.52)	Flurazepam	1.1 (0.56, 2.13)	1.72 (0.78, 3.73)	1.3 (0.64, 2.72)	0.52 (0.02, 6.91)	0.97 (0.46, 2.04)	18935.78 (0.97, 2362037657197563904)	0.63 (0.22, 1.63)	0.65 (0.14, 3.16)	0 (0, 0.42)	1.06 (0.56, 1.94)	1.02 (0.52, 1.99)	0.88 (0.34, 2.21)	1.3 (0.64, 2.7)	1.54 (0.54, 5.13)	1.09 (0.46, 2.52)	1.48 (0.4, 9.95)	1.63 (0.76, 3.48)	1.47 (0.73, 2.99)	2.3 (0.39, 20.06)	0.93 (0.25, 3.4)	1.02 (0.4, 2.8)	1.32 (0.71, 2.46)	0.99 (0.47, 2.09)
0.9 (0.53, 1.5)	1.41 (0.91, 2.3)	0.48 (0.24, 0.96)	0.69 (0.4, 1.22)	0.63 (0.25, 1.47)	0.98 (0.69, 1.4)	21988.79 (7.9, 4186145316.77)	0.85 (0.37, 1.84)	0.91 (0.47, 1.8)	Gaboxadol	1.57 (0.91, 2.72)	1.19 (0.75, 1.9)	0.49 (0.02, 5.9)	0.89 (0.53, 1.51)	17577.85 (0.95, 2025536409810729472)	0.58 (0.23, 1.33)	0.58 (0.14, 2.63)	0 (0, 0.34)	0.96 (0.72, 1.28)	0.93 (0.63, 1.37)	0.79 (0.37, 1.69)	1.18 (0.76, 1.9)	1.41 (0.59, 3.87)	0.99 (0.51, 1.85)	1.32 (0.42, 7.21)	1.46 (0.86, 2.55)	1.34 (0.84, 2.19)	2.12 (0.41, 17.6)	0.85 (0.26, 2.66)	0.95 (0.42, 2.12)	1.2 (0.89, 1.65)	0.92 (0.56, 1.47)
0.58 (0.3, 1.08)	0.9 (0.51, 1.66)	0.31 (0.15, 0.67)	0.44 (0.23, 0.84)	0.4 (0.15, 1.07)	0.62 (0.37, 1.04)	14524.74 (5.47, 2425256066.32)	0.54 (0.23, 1.29)	0.58 (0.27, 1.28)	0.64 (0.37, 1.1)	Indiplon	0.76 (0.41, 1.38)	0.31 (0.01, 4.29)	0.57 (0.29, 1.1)	10478.34 (0.58, 1457889247739480320)	0.37 (0.14, 0.88)	0.37 (0.08, 1.68)	0 (0, 0.22)	0.61 (0.39, 0.97)	0.59 (0.35, 1.04)	0.51 (0.21, 1.21)	0.76 (0.43, 1.37)	0.9 (0.35, 2.62)	0.64 (0.28, 1.35)	0.86 (0.25, 4.04)	0.94 (0.48, 1.81)	0.86 (0.46, 1.61)	1.36 (0.25, 12.2)	0.54 (0.16, 1.81)	0.61 (0.24, 1.47)	0.77 (0.47, 1.25)	0.58 (0.31, 1.06)
0.76 (0.44, 1.32)	1.19 (0.72, 1.98)	0.41 (0.21, 0.8)	0.59 (0.32, 1.04)	0.53 (0.2, 1.29)	0.82 (0.54, 1.24)	18576.08 (7.09, 3883433164.94)	0.72 (0.3, 1.6)	0.77 (0.37, 1.57)	0.84 (0.53, 1.34)	1.31 (0.73, 2.43)	Lemborexant	0.4 (0.02, 4.82)	0.75 (0.42, 1.29)	13424.29 (0.81, 1886028763793719296)	0.49 (0.19, 1.12)	0.5 (0.12, 2.2)	0 (0, 0.29)	0.81 (0.55, 1.17)	0.78 (0.49, 1.25)	0.66 (0.3, 1.49)	1 (0.6, 1.66)	1.18 (0.48, 3.42)	0.84 (0.44, 1.55)	1.09 (0.34, 6.34)	1.24 (0.69, 2.2)	1.14 (0.68, 1.86)	1.76 (0.33, 13.31)	0.72 (0.21, 2.22)	0.78 (0.34, 1.85)	1.01 (0.69, 1.45)	0.76 (0.44, 1.35)
1.88 (0.15, 47.74)	2.95 (0.24, 70.33)	1.06 (0.09, 23.87)	1.44 (0.11, 36.13)	1.3 (0.1, 34.57)	2.01 (0.17, 46.8)	39940.02 (9.28, 17219873934.04)	1.71 (0.15, 44.04)	1.92 (0.14, 42.89)	2.06 (0.17, 51.41)	3.25 (0.23, 76.74)	2.47 (0.21, 60.21)	Lometazepam	1.87 (0.16, 43.69)	24488.23 (0.62, 7885467008897296384)	1.25 (0.09, 30.68)	1.32 (0.07, 34.4)	0 (0, 1.1)	1.97 (0.16, 46.45)	1.93 (0.16, 46.32)	1.66 (0.13, 42.47)	2.45 (0.2, 58.26)	2.98 (0.23, 81.26)	2.1 (0.18, 47.61)	2.92 (0.18, 77.85)	3.1 (0.25, 67.84)	2.83 (0.25, 64.94)	4.29 (0.75, 79.56)	1.84 (0.14, 49.65)	1.99 (0.15, 49.45)	2.43 (0.21, 58.76)	1.87 (0.15, 44.53)
1.01 (0.55, 1.86)	1.59 (0.9, 2.8)	0.55 (0.26, 1.14)	0.77 (0.4, 1.5)	0.7 (0.27, 1.79)	1.1 (0.67, 1.8)	25252.36 (9.36, 4465544922.83)	0.95 (0.44, 1.94)	1.03 (0.49, 2.17)	1.12 (0.66, 1.9)	1.76 (0.91, 3.48)	1.33 (0.77, 2.36)	0.53 (0.02, 6.09)	Loprazolam	17989.57 (1.03, 2417259342315445248)	0.65 (0.23, 1.6)	0.66 (0.15, 2.86)	0 (0, 0.37)	1.07 (0.68, 1.7)	1.03 (0.61, 1.78)	0.88 (0.38, 2.13)	1.33 (0.76, 2.36)	1.58 (0.61, 4.56)	1.12 (0.59, 2.1)	1.46 (0.44, 8.86)	1.66 (0.86, 3.1)	1.51 (1.2, 1.91)	2.38 (0.44, 17.7)	0.97 (0.27, 3.14)	1.06 (0.46, 2.37)	1.35 (0.86, 2.14)	1.02 (0.57, 1.84)

0 (0, 0.99)	0 (0, 1.57)	0 (0, 0.52)	0 (0, 0.76)	0 (0, 0.74)	0 (0, 1.04)	0.1 (0, 83839251.76)	0 (0, 1.04)	0 (0, 1.03)	0 (0, 1.05)	0 (0, 1.72)	0 (0, 1.24)	0 (0, 1.62)	0 (0, 0.97)	Lormetazepam	0 (0, 0.66)	0 (0, 0.77)	0 (0, 0.01)	0 (0, 1.03)	0 (0, 1.01)	0 (0, 0.84)	0 (0, 1.27)	0 (0, 1.57)	0 (0, 1.09)	0 (0, 1.94)	0 (0, 1.61)	0 (0, 1.46)	0 (0, 4.31)	0 (0, 1.13)	0 (0, 1.17)	0 (0, 1.29)	0 (0, 1)
1.55 (0.66, 4.07)	2.46 (1.05, 6.53)	0.83 (0.32, 2.4)	1.19 (0.47, 3.28)	1.1 (0.34, 3.68)	1.68 (0.77, 4.19)	41155.2 (12.37, 7181017920.76)	1.48 (0.48, 4.78)	1.59 (0.61, 4.62)	1.73 (0.75, 4.32)	2.71 (1.14, 7.13)	2.05 (0.89, 5.33)	0.8 (0.03, 11.7)	1.54 (0.63, 4.27)	30324.58 (1.52, 344921964051083008)	Melatonin	1.06 (0.2, 5.08)	0 (0, 0.68)	1.64 (0.77, 3.94)	1.6 (0.73, 3.99)	1.38 (0.46, 4.21)	2.05 (0.89, 5.28)	2.54 (0.73, 8.96)	1.73 (0.68, 4.88)	2.35 (0.58, 14.03)	2.53 (1.02, 6.6)	2.33 (0.97, 6.3)	3.76 (0.58, 35.16)	1.49 (0.4, 6.03)	1.64 (0.54, 5.3)	2.09 (0.96, 5.07)	1.6 (0.67, 4.14)
1.5 (0.33, 6.84)	2.43 (0.55, 10.21)	0.83 (0.18, 4.01)	1.18 (0.25, 5.2)	1.07 (0.2, 5.41)	1.67 (0.38, 6.93)	42235.45 (11.93, 5898655292.38)	1.44 (0.29, 6.92)	1.54 (0.32, 7.22)	1.71 (0.38, 7.23)	2.67 (0.59, 11.91)	1.99 (0.45, 8.63)	0.76 (0.03, 14.08)	1.52 (0.35, 6.61)	30275.02 (1.3, 4466312809992209408)	0.94 (0.2, 5.05)	Midazolam	0 (0, 0.71)	1.65 (0.38, 6.8)	1.6 (0.37, 6.85)	1.35 (0.28, 6.6)	2 (0.45, 8.86)	2.43 (0.48, 13.09)	1.65 (0.49, 6.08)	2.26 (0.41, 18.03)	2.53 (0.54, 11.05)	2.28 (0.55, 9.9)	3.52 (0.45, 40.64)	1.43 (0.23, 9.33)	1.61 (0.31, 8.45)	2.07 (0.47, 8.46)	1.54 (0.35, 6.83)
2464.21 (2.53, 6619701951650543)	3829.2 (4, 9713334755667788)	1182.22 (1.34, 4037569578572606)	1896.57 (1.91, 5197806807266388)	1764.32 (1.74, 4373816430176796)	2552.37 (2.75, 6589136250398431)	190806846.71 (188.13, 8.33347096199098e+21)	2465.48 (2.26, 6509656001664508)	2578.58 (2.4, 5785427955198616)	2720.66 (2.94, 7414162756838757)	4314.39 (4.5, 11658265440013124)	3091.34 (3.39, 8845392591314280)	1099.96 (0.91, 1350417963166788)	2511.24 (2.68, 5843327553754121)	107559669.65 (147.22, 1.27436401111161e+33)	1504.74 (1.47, 4352548131549824)	1780.69 (1.41, 3438744256527272)	Nitrazepam	2523.81 (2.77, 6664742510720662)	2474.93 (2.71, 6557813209014445)	2245.93 (2.03, 5815226256972403)	3137.32 (3.37, 8493580375090738)	4113.95 (3.34, 9775605764200490)	2701.34 (2.7, 6678817362876883)	3776.59 (2.94, 10317556023002688)	3893.27 (4.03, 9734723823151766)	3816.94 (4.06, 8866712673286097)	6407.14 (4.65, 15429767710020960)	2290.76 (2.38, 7030936410811355)	2385.79 (2.6, 6038633015537579)	3159.07 (3.42, 8233671330300521)	2548.94 (2.52, 6114178137690539)
0.94 (0.61, 1.45)	1.47 (1.04, 2.15)	0.51 (0.28, 0.92)	0.72 (0.44, 1.15)	0.66 (0.27, 1.46)	1.02 (0.82, 1.26)	23077.45 (8.65, 4267976382.66)	0.88 (0.41, 1.87)	0.94 (0.51, 1.77)	1.04 (0.78, 1.38)	1.63 (1.03, 2.6)	1.24 (0.86, 1.81)	0.51 (0.02, 6.14)	0.93 (0.59, 1.47)	17632.62 (0.98, 2264860225974790912)	0.61 (0.25, 1.29)	0.61 (0.15, 2.62)	0 (0, 0.36)	Placebo	0.97 (0.75, 1.26)	0.83 (0.41, 1.72)	1.23 (0.87, 1.8)	1.47 (0.63, 3.95)	1.04 (0.56, 1.82)	1.36 (0.45, 7.92)	1.53 (0.95, 2.44)	1.4 (0.95, 2.1)	2.2 (0.43, 17.89)	0.9 (0.29, 2.65)	0.99 (0.45, 2.11)	1.26 (1.04, 1.48)	0.95 (0.63, 1.42)
0.97 (0.58, 1.61)	1.53 (0.98, 2.4)	0.53 (0.27, 0.99)	0.74 (0.44, 1.27)	0.68 (0.26, 1.59)	1.05 (0.75, 1.49)	23968.37 (9.1, 4337660908.23)	0.92 (0.4, 2.02)	0.98 (0.5, 1.93)	1.08 (0.73, 1.58)	1.69 (0.96, 2.86)	1.28 (0.8, 2.03)	0.52 (0.02, 6.25)	0.97 (0.56, 1.63)	18317.58 (0.99, 2351761374003654656)	0.63 (0.25, 1.38)	0.63 (0.15, 2.7)	0 (0, 0.37)	1.03 (0.79, 1.33)	Ramelteon	0.85 (0.41, 1.82)	1.27 (0.82, 2.02)	1.52 (0.62, 4.22)	1.08 (0.54, 1.99)	1.4 (0.46, 7.88)	1.58 (0.92, 2.72)	1.46 (0.89, 2.35)	2.27 (0.44, 18.03)	0.92 (0.28, 2.78)	1.02 (0.44, 2.27)	1.3 (0.94, 1.77)	0.98 (0.61, 1.58)
1.14 (0.5, 2.58)	1.79 (0.81, 3.91)	0.62 (0.24, 1.62)	0.87 (0.37, 2)	0.79 (0.24, 2.27)	1.23 (0.57, 2.55)	29184.07 (8.77, 5503492319.65)	1.07 (0.36, 3.12)	1.13 (0.45, 2.91)	1.26 (0.59, 2.67)	1.96 (0.83, 4.72)	1.51 (0.67, 3.34)	0.6 (0.02, 7.57)	1.13 (0.47, 2.63)	19722.49 (1.19, 2760102707490614272)	0.72 (0.24, 2.16)	0.74 (0.15, 3.63)	0 (0, 0.49)	1.21 (0.58, 2.42)	1.18 (0.55, 2.47)	Seltorexant	1.5 (0.67, 3.29)	1.79 (0.6, 6.15)	1.25 (0.48, 3.02)	1.66 (0.44, 9.96)	1.85 (0.8, 4.23)	1.72 (0.72, 3.83)	2.67 (0.43, 23.54)	1.08 (0.27, 3.99)	1.18 (0.42, 3.33)	1.52 (0.73, 3.06)	1.15 (0.51, 2.53)
0.76 (0.43,	1.19 (0.73,	0.41 (0.2, 0.83)	0.58 (0.32,	0.53 (0.2, 1.27)	0.83 (0.53,	18348.2 (6.72,	0.71 (0.3, 1.65)	0.77 (0.37,	0.85 (0.53,	1.32 (0.73,	1 (0.6, 1.67)	0.41 (0.02,	0.75 (0.42,	15339.12 (0.79,	0.49 (0.19,	0.5 (0.11,	0 (0, 0.3)	0.81 (0.56,	0.79 (0.5, 1.21)	0.67 (0.3, 1.5)	Suvorexant	1.19 (0.48,	0.84 (0.42, 1.6)	1.1 (0.34,	1.24 (0.68,	1.13 (0.67, 1.9)	1.77 (0.34,	0.72 (0.22,	0.8 (0.33,	1.02 (0.68,	0.77 (0.44,

1.33)	2.01)		1.05)		1.23)	3531052425.94)		1.56)	1.32)	2.35)		4.99)	1.32)	1804288978173148928)	1.12)	2.21)		1.14)				3.46)		6.55)	2.22)		14.24)	2.29)	1.82)	1.49)	1.29)
0.64 (0.21, 1.62)	1 (0.35, 2.5)	0.34 (0.11, 0.96)	0.49 (0.16, 1.25)	0.45 (0.11, 1.42)	0.69 (0.25, 1.67)	15889.28 (5.86, 2619786153.38)	0.59 (0.19, 1.86)	0.65 (0.19, 1.87)	0.71 (0.26, 1.71)	1.11 (0.38, 2.86)	0.85 (0.29, 2.09)	0.34 (0.01, 4.41)	0.63 (0.22, 1.64)	12073.33 (0.64, 1721950462454287104)	0.39 (0.11, 1.37)	0.41 (0.08, 2.07)	0 (0, 0.3)	0.68 (0.25, 1.58)	0.66 (0.24, 1.6)	0.56 (0.16, 1.68)	0.84 (0.29, 2.08)	Tasimelteon	0.71 (0.23, 1.91)	0.94 (0.21, 5.26)	1.02 (0.34, 2.72)	0.95 (0.34, 2.4)	1.47 (0.23, 11.99)	0.59 (0.14, 2.53)	0.66 (0.19, 2.06)	0.85 (0.31, 2.03)	0.64 (0.22, 1.63)
0.91 (0.46, 1.84)	1.41 (0.74, 2.9)	0.49 (0.22, 1.09)	0.69 (0.34, 1.52)	0.63 (0.22, 1.86)	0.98 (0.54, 1.83)	23555.4 (7.98, 4456633550.9)	0.87 (0.35, 2)	0.92 (0.4, 2.18)	1.01 (0.54, 1.95)	1.56 (0.74, 3.55)	1.2 (0.64, 2.29)	0.48 (0.02, 5.63)	0.9 (0.48, 1.71)	16798.12 (0.91, 2151455420600413440)	0.58 (0.21, 1.47)	0.61 (0.16, 2.05)	0 (0, 0.37)	0.96 (0.55, 1.79)	0.93 (0.5, 1.87)	0.8 (0.33, 2.06)	1.19 (0.62, 2.38)	1.42 (0.52, 4.41)	Temazepam	1.31 (0.4, 7.72)	1.49 (0.73, 3.04)	1.35 (0.76, 2.47)	2.1 (0.38, 16.12)	0.88 (0.23, 2.88)	0.95 (0.36, 2.56)	1.21 (0.7, 2.19)	0.91 (0.46, 1.93)
0.69 (0.12, 2.27)	1.1 (0.19, 3.46)	0.37 (0.06, 1.4)	0.52 (0.09, 1.76)	0.46 (0.08, 1.93)	0.75 (0.13, 2.32)	18133.59 (6.62, 3533748405.33)	0.63 (0.11, 2.66)	0.68 (0.1, 2.47)	0.76 (0.14, 2.4)	1.16 (0.25, 4)	0.91 (0.16, 2.97)	0.34 (0.01, 5.6)	0.68 (0.11, 2.26)	13784.88 (0.51, 1571593365395150336)	0.43 (0.07, 1.72)	0.44 (0.06, 2.44)	0 (0, 0.34)	0.74 (0.13, 2.23)	0.71 (0.13, 2.17)	0.6 (0.1, 2.29)	0.91 (0.15, 2.93)	1.06 (0.19, 4.72)	0.76 (0.13, 2.5)	Tiagabine	1.13 (0.18, 3.73)	1.04 (0.17, 3.29)	1.53 (0.18, 15.61)	0.63 (0.1, 3.25)	0.69 (0.14, 2.87)	0.92 (0.16, 2.84)	0.69 (0.13, 2.27)
0.61 (0.33, 1.14)	0.96 (0.54, 1.73)	0.33 (0.16, 0.69)	0.47 (0.24, 0.9)	0.43 (0.16, 1.12)	0.66 (0.4, 1.12)	15731.24 (5.13, 2920781127.29)	0.58 (0.24, 1.38)	0.61 (0.29, 1.32)	0.68 (0.39, 1.16)	1.06 (0.55, 2.09)	0.81 (0.46, 1.45)	0.32 (0.01, 4.04)	0.6 (0.32, 1.16)	10592.09 (0.62, 1426561144994260736)	0.4 (0.15, 0.98)	0.4 (0.09, 1.86)	0 (0, 0.25)	0.65 (0.41, 1.06)	0.63 (0.37, 1.08)	0.54 (0.24, 1.25)	0.81 (0.45, 1.47)	0.98 (0.37, 2.91)	0.67 (0.33, 1.38)	0.88 (0.27, 5.54)	Trazodone	0.91 (0.51, 1.67)	1.45 (0.26, 11.82)	0.59 (0.17, 1.85)	0.64 (0.27, 1.58)	0.82 (0.52, 1.33)	0.62 (0.33, 1.15)
0.67 (0.38, 1.18)	1.05 (0.62, 1.76)	0.36 (0.18, 0.72)	0.51 (0.27, 0.95)	0.46 (0.18, 1.14)	0.73 (0.47, 1.12)	16659.7 (6.31, 3002276571.5)	0.63 (0.3, 1.24)	0.68 (0.33, 1.38)	0.74 (0.46, 1.19)	1.16 (0.62, 2.18)	0.88 (0.54, 1.48)	0.35 (0.02, 4.01)	0.66 (0.52, 0.83)	11990.79 (0.68, 1.588552123664e+18)	0.43 (0.16, 1.03)	0.44 (0.1, 1.83)	0 (0, 0.25)	0.71 (0.48, 1.06)	0.69 (0.42, 1.12)	0.58 (0.26, 1.38)	0.88 (0.53, 1.49)	1.05 (0.42, 2.98)	0.74 (0.4, 1.32)	0.96 (0.3, 5.79)	1.09 (0.6, 1.96)	Triazolam	1.57 (0.3, 11.74)	0.64 (0.18, 2.04)	0.7 (0.31, 1.48)	0.89 (0.6, 1.31)	0.67 (0.39, 1.14)
0.43 (0.05, 2.33)	0.66 (0.08, 3.61)	0.23 (0.03, 1.29)	0.33 (0.04, 1.87)	0.3 (0.03, 1.86)	0.46 (0.06, 2.37)	9289.19 (2.93, 2585535383.52)	0.4 (0.05, 2.3)	0.43 (0.05, 2.54)	0.47 (0.06, 2.46)	0.74 (0.08, 4.06)	0.57 (0.08, 3.04)	0.23 (0.01, 1.33)	0.42 (0.06, 2.28)	3900.68 (0.23, 1209035565379407872)	0.27 (0.03, 1.71)	0.28 (0.02, 2.24)	0 (0, 0.21)	0.45 (0.06, 2.33)	0.44 (0.06, 2.3)	0.37 (0.04, 2.34)	0.57 (0.07, 2.95)	0.68 (0.08, 4.33)	0.48 (0.06, 2.65)	0.66 (0.06, 5.53)	0.69 (0.08, 3.85)	0.64 (0.09, 3.38)	Trimipramine	0.39 (0.04, 2.72)	0.44 (0.05, 2.78)	0.57 (0.07, 2.97)	0.43 (0.05, 2.24)
1.06 (0.32, 3.63)	1.64 (0.53, 5.54)	0.55 (0.17, 2.16)	0.81 (0.25, 2.85)	0.74 (0.18, 3.02)	1.13 (0.38, 3.72)	27348.72 (7.47, 5376	0.99 (0.25, 3.97)	1.08 (0.29, 4.01)	1.17 (0.38, 3.8)	1.84 (0.55, 6.21)	1.39 (0.45, 4.75)	0.54 (0.02, 7.36)	1.03 (0.32, 3.68)	17163.18 (0.89, 2960	0.67 (0.17, 2.5)	0.7 (0.11, 4.27)	0 (0, 0.42)	1.11 (0.38, 3.51)	1.08 (0.36, 3.53)	0.93 (0.25, 3.75)	1.38 (0.44, 4.59)	1.7 (0.39, 7.2)	1.14 (0.35, 4.26)	1.58 (0.31, 10.12)	1.7 (0.54, 5.9)	1.56 (0.49, 5.42)	2.54 (0.37, 23.1)	Vestipitant	1.1 (0.28, 4.54)	1.4 (0.46, 4.55)	1.07 (0.34, 3.58)

						627236.7)								464898765712896)																	
0.97 (0.4, 2.27)	1.49 (0.67, 3.59)	0.53 (0.2, 1.27)	0.73 (0.31, 1.75)	0.66 (0.21, 2)	1.03 (0.48, 2.36)	25055.98 (8.04, 4329787060.81)	0.9 (0.32, 2.44)	0.98 (0.36, 2.49)	1.05 (0.47, 2.36)	1.65 (0.68, 4.08)	1.27 (0.54, 2.95)	0.5 (0.02, 6.77)	0.95 (0.42, 2.19)	17983.34 (0.85, 2593657031611710976)	0.61 (0.19, 1.85)	0.62 (0.12, 3.21)	0 (0, 0.39)	1.01 (0.47, 2.2)	0.98 (0.44, 2.25)	0.85 (0.3, 2.35)	1.24 (0.55, 3.04)	1.51 (0.48, 5.15)	1.05 (0.39, 2.78)	1.44 (0.35, 7.22)	1.56 (0.63, 3.75)	1.43 (0.68, 3.21)	2.28 (0.36, 19.99)	0.91 (0.22, 3.53)	Zaleplon	1.27 (0.59, 2.76)	0.96 (0.42, 2.25)
0.75 (0.49, 1.15)	1.17 (0.79, 1.75)	0.41 (0.22, 0.75)	0.57 (0.36, 0.93)	0.53 (0.21, 1.17)	0.81 (0.62, 1.07)	18632.03 (6.76, 3276333748.48)	0.71 (0.33, 1.51)	0.76 (0.41, 1.4)	0.83 (0.61, 1.13)	1.3 (0.8, 2.15)	0.99 (0.69, 1.44)	0.41 (0.02, 4.84)	0.74 (0.47, 1.17)	13791.02 (0.78, 1797374115069873152)	0.48 (0.2, 1.05)	0.48 (0.12, 2.11)	0 (0, 0.29)	0.8 (0.67, 0.96)	0.77 (0.56, 1.06)	0.66 (0.33, 1.37)	0.98 (0.67, 1.48)	1.17 (0.49, 3.19)	0.82 (0.46, 1.42)	1.09 (0.35, 6.26)	1.22 (0.75, 1.94)	1.12 (0.76, 1.66)	1.75 (0.34, 13.84)	0.71 (0.22, 2.16)	0.79 (0.36, 1.71)	Zolpidem	0.75 (0.49, 1.14)
0.99 (0.54, 1.78)	1.55 (0.92, 2.73)	0.54 (0.26, 1.09)	0.76 (0.42, 1.42)	0.7 (0.25, 1.72)	1.08 (0.74, 1.58)	25246.42 (8.5, 4593765502.9)	0.92 (0.42, 2.08)	1.01 (0.48, 2.11)	1.09 (0.68, 1.8)	1.73 (0.94, 3.22)	1.31 (0.74, 2.28)	0.53 (0.02, 6.46)	0.99 (0.54, 1.75)	19431.06 (1, 2415594453246526464)	0.63 (0.24, 1.49)	0.65 (0.15, 2.86)	0 (0, 0.4)	1.05 (0.71, 1.59)	1.02 (0.63, 1.64)	0.87 (0.4, 1.98)	1.3 (0.77, 2.26)	1.56 (0.61, 4.48)	1.09 (0.52, 2.16)	1.45 (0.44, 7.46)	1.61 (0.87, 3)	1.49 (0.88, 2.54)	2.31 (0.45, 18.48)	0.94 (0.28, 2.98)	1.05 (0.44, 2.4)	1.32 (0.88, 2.02)	Zopiclone

League table for amnesia

Flurazepam	0.52 (0.02, 23.67)	0.96 (0.02, 54.63)	1.04 (0.03, 55.2)	1.99 (0.13, 74.41)
1.91 (0.04, 60.26)	Placebo	1.78 (0.01, 364.83)	1.95 (0.25, 20.94)	3.76 (0.65, 39.51)
1.04 (0.02, 56.3)	0.56 (0, 126.39)	Triazolam	1.13 (0.01, 280.73)	2.24 (0.02, 431.75)
0.96 (0.02, 30.46)	0.51 (0.05, 3.96)	0.88 (0, 180.16)	Zaleplon	1.93 (0.29, 17.11)
0.5 (0.01, 7.71)	0.27 (0.03, 1.55)	0.45 (0, 65.24)	0.52 (0.06, 3.48)	Zolpidem

League table for dysgeusia

Estazolam	46337147997.97 (33.53, 1.17663286864429e+41)	29724574470.71 (22.11, 8.03366770087882e+40)	0.27 (0, 3.62789718097115e+34)	15.78 (0, 7.17779671701765e+29)	4080938666.17 (3.17, 1.03777754862486e+40)	4481871327.72 (3.05, 1.11993688792412e+40)	1054118524.4 (0.45, 2.08273520953615e+39)	6837345505.24 (4.46, 1.7433449688923e+40)	1500956831.99 (1.03, 3.77983866971957e+39)	34888620495.68 (24.89, 9.12200297050217e+40)
0 (0, 0.03)	Eszopiclone	0.65 (0.09, 5.12)	0 (0, 0.05)	0 (0, 0.03)	0.09 (0.03, 0.23)	0.11 (0.01, 0.74)	0.03 (0, 1.08)	0.16 (0.01, 1.7)	0.04 (0, 0.23)	0.74 (0.13, 5.65)
0 (0, 0.05)	1.55 (0.2, 11.06)	Flurazepam	0 (0, 0.07)	0 (0, 0.04)	0.15 (0.02, 0.65)	0.18 (0.01, 1.59)	0.04 (0, 1.5)	0.24 (0.01, 3.28)	0.06 (0, 0.39)	1.12 (0.2, 8.26)
3.67 (0, 1.04066122326557e+30)	299717018235.84 (20.48, 6.27182360868629e+35)	201025344700.21 (13.9, 4.06081171237411e+35)	Midazolam	952.9 (0, 8.51466889268459e+27)	27192419369.48 (1.94, 6.74285871523507e+34)	31252790369.51 (1.78, 8.26916214796682e+34)	7758690259.16 (0.26, 1.91939328485452e+34)	46666680467.09 (2.75, 1.30147043638179e+35)	9799486659.7 (0.69, 2.65069062601517e+34)	223300412325.36 (17.92, 4.93879345663196e+35)
0.06 (0, 100208741872.74)	2286899500.8 (39.27, 2.5386315206172e+22)	1496112528.47 (24.98, 1.62832761169025e+22)	0 (0, 4086903823412988)	Nitrazepam	202854667.06 (3.65, 2.18319406913801e+21)	220613729.99 (3.42, 2.98383754960996e+21)	52695703.91 (0.56, 6.67906351075101e+20)	341038148.67 (5.26, 4.38115947773792e+21)	75396290 (1.29, 8.69433015615437e+20)	1788551831.06 (32.71, 1.68192204981776e+22)
0 (0, 0.32)	10.54 (4.4, 38.85)	6.81 (1.55, 50.97)	0 (0, 0.52)	0 (0, 0.27)	Placebo	1.22 (0.09, 6.96)	0.29 (0, 10.83)	1.65 (0.17, 17.19)	0.39 (0.05, 2.14)	7.84 (2.29, 55.11)
0 (0, 0.33)	8.7 (1.35, 194.6)	5.67 (0.63, 188.41)	0 (0, 0.56)	0 (0, 0.29)	0.82 (0.14, 10.85)	Ramelteon	0.24 (0, 19.47)	1.36 (0.09, 50.21)	0.32 (0.02, 6.71)	6.47 (0.84, 222.92)
0 (0, 2.22)	38.18 (0.93, 5010.24)	24.53 (0.67, 3463.34)	0 (0, 3.77)	0 (0, 1.8)	3.49 (0.09, 361.45)	4.09 (0.05, 514.97)	Triazolam	5.91 (0.1, 833.12)	1.33 (0.04, 90.21)	28.73 (0.82, 3739.09)
0 (0, 0.22)	6.41 (0.59, 97.88)	4.15 (0.3, 87.7)	0 (0, 0.36)	0 (0, 0.19)	0.61 (0.06, 5.9)	0.73 (0.02, 11.13)	0.17 (0, 9.62)	Zaleplon	0.23 (0.01, 2.25)	4.74 (0.47, 92.72)
0 (0, 0.97)	27.58 (4.33, 383.01)	17.61 (2.58, 300.48)	0 (0, 1.45)	0 (0, 0.78)	2.58 (0.47, 22.03)	3.11 (0.15, 44.96)	0.75 (0.01, 24.13)	4.26 (0.45, 69.13)	Zolpidem	20.29 (4.79, 259.11)
0 (0, 0.04)	1.36 (0.18, 7.48)	0.89 (0.12, 4.97)	0 (0, 0.06)	0 (0, 0.03)	0.13 (0.02, 0.44)	0.15 (0, 1.19)	0.03 (0, 1.22)	0.21 (0.01, 2.15)	0.05 (0, 0.21)	Zopiclone

League table for difficulty concentrating

Flurazepam	0.78 (0.02, 57.56)	0.26 (0, 37.85)	0 (0, 0.67)	0.7 (0.01, 48.59)
1.29 (0.02, 53.28)	Placebo	0.33 (0.01, 5.91)	0 (0, 1.43)	0.94 (0.08, 5.29)
3.84 (0.03, 489.63)	2.99 (0.17, 77.47)	Seltorexant	0 (0, 5.75)	2.84 (0.11, 51.91)
5050545414.11 (1.5, 6.37477032925039e+33)	4555269785.57 (0.7, 5.96052285069289e+33)	1452179740.5 (0.17, 2.15085081022069e+33)	Triazolam	3844444643.85 (0.58, 4.93968784612731e+33)
1.42 (0.02, 91.84)	1.06 (0.19, 12.04)	0.35 (0.02, 9.15)	0 (0, 1.73)	Zolpidem

League table for impaired coordination

Estazolam	6.68 (0.19, 495.57)	1592891076702.98 (9.77, 9.8148354754061e+36)	0 (0, 4.21)	0 (0, 5.11)	14.83 (0.07, 5016.8)	9562836.25 (3.43, 2.87889706580348e+22)
0.15 (0, 5.38)	Flurazepam	187270954579.22 (2.19, 1.03814488168515e+36)	0 (0, 0.35)	0 (0, 0.44)	2.02 (0.04, 135.96)	1111097.35 (0.83, 3.92751801771538e+21)
0 (0, 0.1)	0 (0, 0.46)	Nitrazepam	0 (0, 0)	0 (0, 0)	0 (0, 0.59)	0 (0, 2346679398550520)
9381.88 (0.24, 1985168036512689)	69236.33 (2.89, 12507209125575436)	233966859380266176 (2487.84, 6.42509347999195e+43)	Placebo	0.64 (0.02, 18.95)	169589.2 (2.35, 34897162296079036)	2993435688199.69 (657.17, 7.47936462083584e+27)
16069.65 (0.2, 3528672924776536)	119286.63 (2.26, 22938782285079904)	362845903100858752 (3005.84, 1.07102127223705e+44)	1.56 (0.05, 50.54)	Temazepam	290279.83 (2.02, 65100391911272960)	4992736595546.09 (801.76, 1.41229698595877e+28)
0.07 (0, 14.45)	0.49 (0.01, 23)	81647024129.9 (1.7, 5.40704741200006e+35)	0 (0, 0.43)	0 (0, 0.49)	Triazolam	484446.62 (0.8, 1.85160569337427e+21)
0 (0, 0.29)	0 (0, 1.21)	55310.42 (0, 3.65505040977605e+31)	0 (0, 0)	0 (0, 0)	0 (0, 1.25)	Zaleplon

League table for nervousness

Doxepin	0.54 (0.02, 11.55)	2.7 (0.17, 47.03)	0.71 (0.05, 11.65)	0 (0, 0.53)	0.94 (0.01, 48.6)	1.43 (0.16, 14.09)	1.49 (0.08, 36.18)	3.7 (0.32, 64.62)	0 (0, 0.29)	1.11 (0.04, 32.43)	1.82 (0.14, 35.8)	213364.97 (0.26, 1.10528138514058e+20)
1.85 (0.09, 41.52)	Estazolam	4.99 (0.36, 81.84)	1.3 (0.16, 12.48)	0 (0, 0.96)	1.73 (0.02, 81.77)	2.6 (0.35, 24.14)	2.74 (0.16, 60.14)	6.74 (0.75, 101.82)	0 (0, 0.51)	2.01 (0.09, 53.83)	3.33 (0.32, 56.31)	403994.22 (0.51, 2.19293359411974e+20)
0.37 (0.02, 5.72)	0.2 (0.01, 2.76)	Eszopiclone	0.26 (0.02, 2.6)	0 (0, 0.17)	0.35 (0, 12.99)	0.54 (0.09, 2.76)	0.55 (0.04, 8.81)	1.38 (0.17, 14)	0 (0, 0.1)	0.41 (0.02, 8.05)	0.67 (0.08, 7.93)	76900.51 (0.09, 39106705352513388544)
1.41 (0.09, 22.13)	0.77 (0.08, 6.33)	3.79 (0.38, 42.17)	Flurazepam	0 (0, 0.65)	1.34 (0.02, 43.7)	2 (0.42, 10.83)	2.1 (0.18, 29.87)	5.15 (0.98, 43.84)	0 (0, 0.37)	1.56 (0.09, 27.83)	2.54 (0.42, 23.5)	298793.13 (0.42, 1.53616064227376e+20)
3844847.78 (1.9, 25973010712932605952)	2113355.69 (1.05, 1.261142473803e+19)	11142800.89 (5.89, 68157333996651487232)	2909366.14 (1.55, 17778391180428163072)	Lometazepam	3443567.7 (1.18, 27838470358076440576)	5622760.5 (3.76, 37968082396669149184)	6299426.53 (3.08, 39085190171132698624)	15861086.52 (8.98, 98868003260391849984)	0 (0, 18995206012639.1)	4426425.42 (2.11, 30663387874211053568)	7921296.85 (4.19, 49337516416830644224)	4425910421950.98 (270.14, 7.06692390681171e+31)
1.06 (0.02, 86.27)	0.58 (0.01, 42.04)	2.86 (0.08, 200.22)	0.74 (0.02, 45.51)	0 (0, 0.85)	Nitrazepam	1.5 (0.06, 74.81)	1.55 (0.05, 105.61)	3.78 (0.26, 160.5)	0 (0, 0.39)	1.14 (0.03, 83.07)	1.9 (0.08, 112.7)	221900.78 (0.71, 1.10389316040908e+20)
0.7 (0.07, 6.27)	0.38 (0.04, 2.88)	1.86 (0.36, 10.74)	0.5 (0.09, 2.4)	0 (0, 0.27)	0.67 (0.01, 15.98)	Placebo	1.05 (0.13, 9.28)	2.58 (0.74, 12.57)	0 (0, 0.17)	0.78 (0.06, 8.94)	1.27 (0.3, 7.48)	146255.68 (0.22, 68593952327170433024)
0.67 (0.03, 12.71)	0.37 (0.02, 6.13)	1.82 (0.11, 25.34)	0.48 (0.03, 5.48)	0 (0, 0.32)	0.65 (0.01, 20.04)	0.95 (0.11, 7.77)	Temazepam	2.46 (0.36, 21.85)	0 (0, 0.19)	0.74 (0.03, 14.83)	1.22 (0.15, 11.94)	143411.11 (0.17, 63552449401680388096)
0.27 (0.02, 3.13)	0.15 (0.01, 1.34)	0.73 (0.07, 5.83)	0.19 (0.02, 1.03)	0 (0, 0.11)	0.26 (0.01, 3.9)	0.39 (0.08, 1.36)	0.41 (0.05, 2.82)	Triazolam	0 (0, 0.07)	0.3 (0.02, 2.79)	0.49 (0.1, 2.45)	58884.1 (0.09, 23349601200825421824)
975108622.5 (3.44, 7.09071191336028e+28)	570338405.4 (1.94, 3.50264566667494e+28)	2794151693.48 (9.76, 1.69291003352013e+29)	738336160.15 (2.69, 4.88133684810065e+28)	308.87 (0, 2.20454505488019e+22)	922204471.6 (2.58, 6.88576040798918e+28)	1443349243.19 (5.85, 9.62352905671349e+28)	1473795037.89 (5.29, 9.43735519448532e+28)	3810497706.38 (14.87, 2.55303935215372e+29)	Trimipramine	1047244740.83 (3.94, 7.43378964535418e+28)	1883673205.97 (7.2, 1.29653983345236e+29)	2402358012929017 (1556.63, 1.80568347474611e+38)
0.9 (0.03, 24.9)	0.5 (0.02, 11.66)	2.44 (0.12, 52.69)	0.64 (0.04, 11.31)	0 (0, 0.47)	0.88 (0.01, 35.62)	1.29 (0.11, 16.44)	1.35 (0.07, 30.63)	3.36 (0.36, 45.57)	0 (0, 0.25)	Zaleplon	1.65 (0.12, 30.3)	211252.41 (0.22, 81272585896333737984)

0.55 (0.03, 7.05)	0.3 (0.02, 3.08)	1.49 (0.13, 13.31)	0.39 (0.04, 2.38)	0 (0, 0.24)	0.53 (0.01, 12.22)	0.79 (0.13, 3.35)	0.82 (0.08, 6.61)	2.04 (0.41, 10.26)	0 (0, 0.14)	0.6 (0.03, 8.21)	Zolpidem	120863.03 (0.16, 49542453227141300224)
0 (0, 3.86)	0 (0, 1.98)	0 (0, 10.81)	0 (0, 2.38)	0 (0, 0)	0 (0, 1.42)	0 (0, 4.56)	0 (0, 5.85)	0 (0, 11.14)	0 (0, 0)	0 (0, 4.57)	0 (0, 6.24)	Zopiclone

League table for nightmare

Flunitrazepam	42.73 (0.4, 12717.36)	2.76 (0.05, 242.61)	0.49 (0, 101.46)	0.65 (0.06, 6.55)	12001870.13 (0.34, 8.84867165179218e+23)	0.6 (0.02, 14.65)
0.02 (0, 2.53)	Lemborexant	0.07 (0, 0.7)	0.01 (0, 0.52)	0.02 (0, 0.93)	253262.49 (0, 1.69452156146054e+22)	0.01 (0, 0.69)
0.36 (0, 19.75)	13.94 (1.43, 496.72)	Placebo	0.19 (0, 3.33)	0.24 (0, 6.18)	4109971.27 (0.12, 2.58668331047147e+23)	0.22 (0.01, 4.42)
2.03 (0.01, 486.71)	84.92 (1.92, 13538.74)	5.41 (0.3, 249.34)	Temazepam	1.32 (0.01, 195.13)	25840686.19 (0.48, 1.86377106181452e+24)	1.2 (0.01, 145.46)
1.55 (0.15, 15.93)	65.67 (1.08, 12618.36)	4.21 (0.16, 208.52)	0.76 (0.01, 97.35)	Triazolam	18129379.17 (0.68, 1.33619210642682e+24)	0.92 (0.09, 8.78)
0 (0, 2.93)	0 (0, 211.4)	0 (0, 8.19)	0 (0, 2.08)	0 (0, 1.48)	Zaleplon	0 (0, 1.03)
1.68 (0.07, 44.31)	70.67 (1.46, 11288.38)	4.53 (0.23, 179.22)	0.83 (0.01, 87.88)	1.08 (0.11, 11.11)	19224240.93 (0.97, 1.39589903785774e+24)	Zolpidem

League table for asthenia

Doxepin	0.33 (0, 30.96)	0.52 (0.01, 44.04)	0.9 (0.01, 78.81)	0.23 (0, 36.92)	38915004.97 (0.16, 2.02364483811609e+27)	0.64 (0.02, 18.53)	0 (0, 0.07)	0.64 (0, 82.56)	0.53 (0, 35.3)	0.16 (0, 7)
3.01 (0.03, 300.03)	Estazolam	1.55 (0.02, 105.15)	2.7 (0.13, 55.96)	0.7 (0, 91)	118514142.12 (0.55, 5.83989556424994e+27)	1.92 (0.09, 40.17)	0 (0, 0.22)	1.92 (0.02, 201.26)	1.6 (0.02, 82.73)	0.51 (0.01, 15.75)
1.93 (0.02, 184.57)	0.65 (0.01, 45.02)	Eszopiclone	1.74 (0.03, 113.8)	0.46 (0, 55.57)	78347713.44 (0.36, 3.54264630987244e+27)	1.25 (0.07, 23.73)	0 (0, 0.13)	1.23 (0.01, 120.42)	1.05 (0.01, 49.78)	0.33 (0.01, 9.07)
1.12 (0.01, 103.51)	0.37 (0.02, 7.61)	0.57 (0.01, 36.67)	Flurazepam	0.26 (0, 31.87)	42714921.82 (0.21, 2.11188993820086e+27)	0.71 (0.04, 13.74)	0 (0, 0.08)	0.7 (0.01, 69.04)	0.6 (0.01, 28.72)	0.19 (0, 5.32)
4.31 (0.03, 1039.72)	1.43 (0.01, 279.42)	2.17 (0.02, 387.6)	3.81 (0.03, 703.3)	Lometazepam	185699821.35 (0.57, 8.47947686838883e+27)	2.64 (0.06, 204.09)	0 (0, 0.44)	2.63 (0.06, 210.19)	2.19 (0.02, 330.56)	0.67 (0.01, 72.05)
0 (0, 6.33)	0 (0, 1.82)	0 (0, 2.8)	0 (0, 4.77)	0 (0, 1.74)	Loprazolam	0 (0, 2.4)	0 (0, 0)	0 (0, 4.35)	0 (0, 2.59)	0 (0, 0.69)

1.55 (0.05, 50.73)	0.52 (0.02, 10.99)	0.8 (0.04, 15.28)	1.41 (0.07, 27.32)	0.38 (0, 15.9)	60603982.94 (0.42, 2.627319855 28508e+27)	Placebo	0 (0, 0.08)	0.99 (0.03, 33.88)	0.84 (0.04, 11.57)	0.26 (0.02, 1.78)
2311582659. 82 (13.67, 7.658897290 41104e+27)	726018903.9 7 (4.61, 2.544320209 23541e+27)	1145920337. 75 (7.46, 3.650906453 73686e+27)	2029286838. 75 (13.2, 6.296308897 33955e+27)	497498654.9 2 (2.29, 1.567269281 00197e+27)	4534297359 943562240 (11611.34, 4.987501787 98692e+42)	1375224349. 46 (13.27, 4.373561586 56144e+27)	Triazolam	1446693664. 73 (7.9, 4.531598995 83012e+27)	1073474607. 37 (7.48, 3.416789208 09581e+27)	305374102. 25 (2.56, 9.78340382 185071e+26)
1.57 (0.01, 216.26)	0.52 (0, 54.65)	0.81 (0.01, 77.81)	1.42 (0.01, 138.06)	0.38 (0, 16.48)	62793736.81 (0.23, 2.947545111 98317e+27)	1.01 (0.03, 33.74)	0 (0, 0.13)	Trimipramin e	0.82 (0.01, 63.55)	0.25 (0, 12.49)
1.9 (0.03, 202.71)	0.62 (0.01, 48.97)	0.95 (0.02, 71.24)	1.66 (0.03, 124.08)	0.46 (0, 59.89)	77531136.49 (0.39, 3.484202755 29613e+27)	1.19 (0.09, 25.06)	0 (0, 0.13)	1.22 (0.02, 131.15)	Zaleplon	0.32 (0.02, 4.41)
6.16 (0.14, 554.53)	1.97 (0.06, 138.52)	3 (0.11, 196.01)	5.27 (0.19, 354.2)	1.49 (0.01, 169.4)	260102437.3 5 (1.46, 1.143170449 56853e+28)	3.85 (0.56, 60.32)	0 (0, 0.39)	3.92 (0.08, 372.74)	3.11 (0.23, 65.65)	Zolpidem

Results of network meta-analysis for secondary outcomes

League table for abnormal dreams

Doxepin	330619.83 (2.42, 243948831128003040)	2.32 (0.11, 69.18)	2.31 (0.17, 47.43)	1.68 (0.18, 19.84)	0 (0, 947421362.41)	5.44 (0.4, 93.54)	2.82 (0.16, 60.66)	4.71 (0.27, 102.63)
0 (0, 0.41)	Esmirtazapine	0 (0, 1.05)	0 (0, 0.88)	0 (0, 0.53)	0 (0, 59439.21)	0 (0, 1.98)	0 (0, 1.06)	0 (0, 1.79)
0.43 (0.01, 9.52)	131912.45 (0.96, 102760625696135072)	Eszopiclone	1.01 (0.07, 15.7)	0.75 (0.07, 5.96)	0 (0, 381003661.08)	2.38 (0.15, 30.88)	1.23 (0.06, 19.66)	2.04 (0.1, 33.59)
0.43 (0.02, 5.73)	130385.45 (1.14, 92999932947147072)	0.99 (0.06, 15.35)	Lemborexant	0.75 (0.13, 2.82)	0 (0, 371470573.21)	2.38 (0.26, 16.96)	1.23 (0.1, 11.34)	2.04 (0.16, 19.45)
0.6 (0.05, 5.58)	182685.6 (1.9, 121350028117336496)	1.34 (0.17, 15.1)	1.33 (0.36, 7.8)	Placebo	0 (0, 467659809.55)	3.17 (0.86, 13.96)	1.64 (0.3, 10.45)	2.73 (0.47, 18.03)
292.43 (0, 12273510033356630)	763921377.92 (0, 9.35871863461166e+24)	707 (0, 27597353357882280)	705.56 (0, 30224521956082612)	498.95 (0, 18325892750273296)	Ramelteon	1652.46 (0, 63390571574489376)	818.31 (0, 31733859638381364)	1370.08 (0, 50248762872300248)
0.18 (0.01, 2.49)	56353.41 (0.51, 40932091644412656)	0.42 (0.03, 6.47)	0.42 (0.06, 3.84)	0.32 (0.07, 1.16)	0 (0, 151444806.71)	Suvorexant	0.52 (0.05, 4.82)	0.87 (0.09, 8.37)

0.35 (0.02, 6.12)	110479.65 (0.95, 75269291868233328)	0.81 (0.05, 15.74)	0.81 (0.09, 9.93)	0.61 (0.1, 3.35)	0 (0, 334133660.64)	1.92 (0.21, 18.69)	Zaleplon	1.67 (0.34, 8.07)
0.21 (0.01, 3.75)	68178.69 (0.56, 46216936629850600)	0.49 (0.03, 9.8)	0.49 (0.05, 6.09)	0.37 (0.06, 2.11)	0 (0, 190701329.52)	1.15 (0.12, 11.72)	0.6 (0.12, 2.97)	Zolpidem

League table for abnormal vision

Doxepin	0 (0, 15.66)	0.05 (0, 38067898736494680)	0 (0, 0.3)	0 (0, 0)	0.64 (0, 20127773843050)	0 (0, 569484.22)
235507955498.76 (0.06, 1.72239099755608e+29)	Flurazepam	13823372913.26 (0, 2.24630695536412e+29)	299.17 (0, 4358726367353805)	0 (0, 44852034067.52)	39769202504.92 (53.85, 2.37359669601586e+26)	899711.08 (1.72, 7048821820667620352)
19.26 (0, 2741905522899928064)	0 (0, 615.67)	Melatonin	0 (0, 2.75)	0 (0, 0.01)	15 (0, 33023996185155)	0 (0, 11771935.43)
45879233.4 (3.3, 3.20895370041416e+23)	0 (0, 342127.2)	2678701.62 (0.36, 5.88708287425962e+21)	Placebo	0 (0, 1.11)	56218932.05 (44.49, 818067890916035584)	1158.33 (1.75, 17913647420.41)
6553784044639811 (1031.39, 3.1703308010705e+37)	11208.47 (0, 4.02180255042673e+23)	196320146558718 (144.32, 3.27723464593412e+35)	4545030.12 (0.9, 1.0646316896446e+24)	Ramelteon	1734059180779563 (25966.14, 1.03409079750339e+34)	22133329042.64 (97.13, 3.00871997567575e+28)
1.56 (0, 28821653837878268)	0 (0, 0.02)	0.07 (0, 870971559918847)	0 (0, 0.02)	0 (0, 0)	Triazolam	0 (0, 2.76)
19754.11 (0, 1.06797578278769e+20)	0 (0, 0.58)	1081.99 (0, 5362028100828246016)	0 (0, 0.57)	0 (0, 0.01)	6047.12 (0.36, 68802305483543)	Zolpidem

League table for gait disturbance

Daridorexant	8.39123008786731e+25 (163679.69, 5.68799251003114e+62)	1694249704.29 (3.46, 1.18926923468434e+30)	4940199590.05 (11.13, 3.43672964758662e+30)
0 (0, 0)	EVT_201	0 (0, 0.24)	0 (0, 1.15)
0 (0, 0.29)	435352202871855 (4.17, 1.37327225585815e+49)	Placebo	2.62 (0.03, 343.53)
0 (0, 0.09)	159681652807359 (0.87, 4.94726658404461e+48)	0.38 (0, 31.14)	Zolpidem

League table for hypnagogic hallucinations

Lemborexant	0 (0, 0.69)	0.97 (0, 1328688329849682944)	2762936.69 (1.76, 2.79978122508132e+21)
1693878.05 (1.45, 1.89997016674926e+23)	Placebo	4202361.72 (1.53, 1.82092673901922e+23)	373503799246374 (1194.98, 1.23539502492741e+33)
1.03 (0, 274726420818005696)	0 (0, 0.65)	Suvorexant	14323769.45 (0, 1.73655302504527e+27)
0 (0, 0.57)	0 (0, 0)	0 (0, 1659313260606.9)	Zolpidem

League table for tremor

Triazolam	0.28 (0, 156695295077771616)	0 (0, 0.28)
3.52 (0, 19495652055359909888)	Zaleplon	0 (0, 1)
77328768.01 (3.53, 1.68600631597514e+24)	20545775.1 (1, 2.30336667179923e+23)	Zolpidem

League table for paresthesia

Placebo	0.97 (0.04, 12.73)	1.87 (0.57, 7.56)	1.24 (0.31, 5.19)
1.04 (0.08, 26.82)	Triazolam	1.97 (0.14, 56.62)	1.29 (0.1, 34.11)
0.53 (0.13, 1.75)	0.51 (0.02, 7.03)	Zaleplon	0.67 (0.18, 2.06)
0.8 (0.19, 3.2)	0.78 (0.03, 9.72)	1.5 (0.49, 5.49)	Zolpidem

League table for gastroenteritis

Doxepin	0 (0, 0.35)	0 (0, 0.89)	0 (0, 0.44)	0 (0, 0.49)	0 (0, 0.69)
2517018.92 (2.87, 2784437208905069056)	Esmirtazapine	2.77 (0.15, 48.81)	1.69 (0.18, 13.68)	1.42 (0.06, 27.71)	2.26 (0.13, 35.94)
893398.49 (1.12, 974190076636475776)	0.36 (0.02, 6.58)	Lemborexant	0.61 (0.08, 4.03)	0.52 (0.03, 8.35)	0.82 (0.06, 11.1)
1504585.38 (2.27, 1558503679363963648)	0.59 (0.07, 5.46)	1.63 (0.25, 12.07)	Placebo	0.86 (0.09, 6.99)	1.35 (0.22, 8.25)
1780098.37 (2.06, 1872661011215551744)	0.7 (0.04, 16.68)	1.93 (0.12, 39.42)	1.16 (0.14, 11.12)	Ramelteon	1.55 (0.11, 28.32)
1100535.56 (1.44, 1240362319628773888)	0.44 (0.03, 7.68)	1.22 (0.09, 17.61)	0.74 (0.12, 4.51)	0.64 (0.04, 9.44)	Zolpidem

League table for decreased appetite

Doxepin	3889842348.3 (18.46, 1.26516315534247e+31)	4.08 (0.16, 221.19)	0 (0, 5.74)	22102393.67 (2.67, 2.09882900480145e+22)	0 (0, 511298400.08)
0 (0, 0.05)	Indiplon	0 (0, 0.13)	0 (0, 0)	0.01 (0, 348999712292194)	0 (0, 494.31)
0.24 (0, 6.35)	810579859.25 (7.51, 2.89387549645783e+30)	Placebo	0 (0, 0.77)	4564458.12 (1, 4.12924532672087e+21)	0 (0, 137310942.38)
339034912.25 (0.17, 5.03705677959022e+27)	66489221521106239488 (20380.87, 6.50737047394375e+45)	1613751125.9 (1.3, 1.92160555412535e+28)	Ramelteon	160112602177368000 (1088.04, 2.31700324503698e+40)	2162.88 (0, 5.88870810895489e+26)
0 (0, 0.37)	173.83 (0, 2.62197059697039e+23)	0 (0, 1)	0 (0, 0)	Triazolam	0 (0, 0.13)
166076.25 (0, 1.0093309551396e+27)	22323532998572256 (0, 3.10801645765528e+43)	711311.79 (0, 3.14181074530977e+27)	0 (0, 5.22086716516428e+20)	5398800045811.42 (7.44, 7.38339697908574e+38)	Zolpidem

League table for pain gastralgia

Nitrazepam	11913976.43 (4.87, 917929945544680960)	0 (0, 639478.79)
0 (0, 0.21)	Placebo	0 (0, 0.03)
911056.45 (0, 2.13496582579114e+27)	76468616483717.2 (37.53, 1.18612682918159e+34)	Zopiclone

League table for insomnia exacerbated

Esmirtazapine	0.03 (0, 52225632926160.2)	1.92 (0.09, 37.49)	1.57 (0.03, 95.44)	3757782.07 (1.01, 80368387373012877312)
31.33 (0, 1591348667516940544)	Lemborexant	58.25 (0, 2712792398511800832)	47.61 (0, 2383804162682025472)	105871184.33 (3.47, 5.12095209959319e+23)
0.52 (0.03, 10.69)	0.02 (0, 22317539523212.3)	Placebo	0.82 (0.05, 13.93)	1897994.95 (0.76, 39294846146070847488)
0.63 (0.01, 39.05)	0.02 (0, 31639141718842.1)	1.22 (0.07, 20.43)	Ramelteon	2326447.13 (0.67, 47926454330504544256)
0 (0, 0.99)	0 (0, 0.29)	0 (0, 1.31)	0 (0, 1.5)	Zolpidem

League table for sleep paralysis

Daridorexant	111.61 (0, 2.79760412740528e+22)	0 (0, 0.81)	0 (0, 0)	0 (0, 0)	0 (0, 0.16)	0 (0, 1366495710587.21)	0 (0, 1980.27)	0 (0, 0)
0.01 (0, 3128969418741852160)	Lemborexant	0 (0, 0.18)	0 (0, 0)	0 (0, 0)	0 (0, 0.04)	0 (0, 244051325966.01)	0 (0, 0.07)	0 (0, 0)
820577896.57 (1.23, 3.51518815883869e+27)	172572620385.62 (5.65, 1.12020692604743e+30)	Lormetazepam	0 (0, 0.36)	0 (0, 0.11)	0.41 (0.01, 14.2)	105216.91 (0.15, 11831313603140794368)	0 (0, 2552357402.85)	0 (0, 0.07)
1.18443236672893e+20 (11360.48, 3.13999969196719e+48)	8.00853483435366e+22 (178380.9, 7.8938118047802e+50)	3104527589.11 (2.75, 2.48590366066988e+31)	Midazolam	32.66 (0, 5.39829650288235e+24)	1077747013.08 (1.79, 8.28378730981661e+30)	10801077997309120 (1103.22, 7.38077083470258e+39)	114317.64 (0, 8.9957694278492e+29)	0.29 (0.01, 5.75)
1961433459396806912 (12363.83, 2.65706344258413e+39)	3.42121547292443e+20 (373297.01, 2.00819215157212e+42)	72284154.28 (9.31, 2.34945408051265e+23)	0.03 (0, 160636914032798688)	Nitrazepam	26902841.04 (6.83, 7.86677672902518e+22)	50179404076630 (1443.94, 4.37897663675748e+31)	292.82 (0, 1.67120252210635e+26)	0.01 (0, 48065596786500560)
1929222588.89 (6.11, 7.13206493150523e+27)	443514943029.65 (24, 2.19323436410022e+30)	2.46 (0.07, 152.48)	0 (0, 0.56)	0 (0, 0.15)	Placebo	251942.29 (0.88, 24589316673429147648)	0 (0, 4981161911.48)	0 (0, 0.11)
2190.54 (0, 5.43474456533424e+23)	445428.9 (0, 3.97378557255314e+25)	0 (0, 6.89)	0 (0, 0)	0 (0, 0)	0 (0, 1.14)	Suvorexant	0 (0, 114803.01)	0 (0, 0)
1427838689180016 (0, 1.46178473836725e+46)	27506998477088880 (14.15, 1.5409864955033e+47)	93785.66 (0, 1.5365068364447e+28)	0 (0, 9.82008936766127e+21)	0 (0, 1.78886582769488e+21)	33970.5 (0, 3.31574429274767e+27)	207806861033.96 (0, 1.13952776472232e+38)	Zolpidem	0 (0, 2.28567676549444e+21)
4.09423516061261e+20 (47687.71, 1.23221810463737e+49)	2.97344248293809e+23 (718023.22, 2.42546383556443e+51)	10461610007.5 (13.97, 7.94490756840577e+31)	3.42 (0.17, 83.57)	112.56 (0, 1.62825692654473e+25)	3571399183.93 (9.09, 2.85304411964342e+31)	36692956510480232 (5357.09, 2.20740428932694e+40)	419230.42 (0, 3.16881302220918e+30)	Zopiclone

League table for peripheral oedema

Doxepin	0 (0, 1.67)	13.37 (0, 9.78399086964804e+24)	0 (0, 0.3)	0 (0, 1363895331165008)	0.57 (0, 67268020345443912)
627116097.31 (0.6, 1.0653609818079e+29)	Esmirtazapine	12547236393.44 (1.46, 8.77341005440815e+31)	0.28 (0.01, 2.81)	726616.85 (0.11, 1.94038953254408e+21)	483736140.75 (1.9, 1.58994958201958e+24)
0.07 (0, 4.23419833923316e+22)	0 (0, 0.69)	Eszopiclone	0 (0, 0.13)	0 (0, 1397256350856842)	0.03 (0, 3471370561414254)
2384386092.65 (3.29, 4.64943826509332e+29)	3.56 (0.36, 95.45)	47882896862.63 (7.45, 3.68591373590545e+32)	Placebo	2807789.47 (0.66, 7.3736025064609e+21)	1945273454.18 (10.28, 6.10264404044428e+24)
270.3 (0, 2.31689006834568e+24)	0 (0, 8.79)	4331.04 (0, 3.49775838945089e+27)	0 (0, 1.51)	Ramelteon	231.9 (0, 406999645607679 26272)
1.74 (0, 7.35686539506643e+23)	0 (0, 0.53)	34.54 (0, 6.25148475637247e+24)	0 (0, 0.1)	0 (0, 4515515516738390)	Suvorexant

League table for pruritis

Daridorexant	0.27 (0, 102484882752369024)	0 (0, 0.65)	0 (0, 23439.29)	0 (0, 0)	0 (0, 0.29)
3.74 (0, 425805455016921728)	Doxepin	0 (0, 0.37)	0 (0, 211288.21)	0 (0, 221.19)	0 (0, 1496970.86)
227080755.35 (1.54, 1.96183647412538e+23)	51544290.06 (2.69, 7.3918468554157e+22)	Placebo	0 (0, 12107948904.67)	0 (0, 18138230.05)	0 (0, 61483167346.16)
3602699229448.52 (0, 2.59248495412154e+33)	1001909427863.48 (0, 2.22200698123633e+33)	896.46 (0, 6.12607856262329e+20)	Ramelteon	0 (0, 6745932961530683392)	1.14 (0, 2.16548212542367e+23)
105999040321632592 (1326.03, 6.24334240429726e+39)	123337781945439920 (0, 1.43629967288364e+42)	200741716.4 (0, 4.50710230044948e+29)	118525.85 (0, 3.74563728472197e+29)	Triazolam	21785.72 (0.43, 493512471243807)
250011976310.22 (3.43, 3.64459222044718e+34)	540372134538.48 (0, 5.77054673896266e+36)	1155.02 (0, 8.26370298165691e+23)	0.88 (0, 1.34140618481573e+23)	0 (0, 2.3)	Zolpidem

League table for sweating

Alprazolam	0.14 (0, 19060663856328.9)	8658956 (2.6, 4.8254067016119e+21)	0.51 (0, 60699814702498.7)
7.24 (0, 263143391137886)	Doxepin	116873683.96 (8.77, 1.18794791760741e+21)	3.51 (0.31, 51.28)
0 (0, 0.39)	0 (0, 0.11)	Paroxetine	0 (0, 0.31)
1.95 (0, 64819787073141.7)	0.29 (0.02, 3.19)	30846003.41 (3.21, 2.65500880098464e+20)	Placebo

League table for skin diseases

Daridorexant	0 (0, 0.11)	0.1 (0, 1985577209189.89)	0 (0, 0.17)	0 (0, 0.1)	0 (0, 1.3)
43872133.01 (8.89, 2.20433578689336e+21)	Doxepin	4515061.85 (4.11, 5978424128885941248)	1.63 (0.1, 26.78)	1.06 (0.08, 13.46)	55.86 (0.07, 11854011.09)
10.01 (0, 894823970605254)	0 (0, 0.24)	Escitalopram	0 (0, 0.32)	0 (0, 0.2)	0 (0, 2.03)
25355792.41 (5.86, 1.35113402447056e+21)	0.61 (0.04, 9.95)	2736788.97 (3.1, 3458309058117180416)	Eszopiclone	0.65 (0.19, 2.17)	32.01 (0.06, 6073795.25)
39236351.54 (9.65, 1.92242379995707e+21)	0.95 (0.07, 11.98)	4149562.6 (5.08, 4916144898486404096)	1.54 (0.46, 5.17)	Placebo	48.4 (0.11, 8715070.19)
235677.3 (0.77, 2.4939557515409e+19)	0.02 (0, 15.22)	24081.86 (0.49, 81941916836554704)	0.03 (0, 16.07)	0.02 (0, 9.23)	Zolpidem

League table for influenza

Daridorexant	0 (0, 2.95)	4.55 (0, 23380947016373.2)	0 (0, 2.47)	0.02 (0, 15088355.56)	0 (0, 2.63)	0 (0, 3.34)
47761.21 (0.34, 718418319561112)	Lemborexant	433903.63 (0.39, 40877027926709680)	0.94 (0.24, 3.68)	1031.66 (0.26, 9065779327.29)	0.93 (0.16, 5.6)	0.84 (0.05, 13.11)
0.22 (0, 274173317094.35)	0 (0, 2.56)	Melatonin	0 (0, 2.19)	0 (0, 10680161.08)	0 (0, 2.41)	0 (0, 2.7)
50770.91 (0.4, 712044922521845)	1.07 (0.27, 4.25)	469900.86 (0.46, 41607711014221496)	Placebo	1100.81 (0.31, 9222357163.47)	0.99 (0.33, 3.18)	0.9 (0.08, 10.07)
42.88 (0, 3516146864067.69)	0 (0, 3.78)	216.22 (0, 178771581698174)	0 (0, 3.18)	Ramelteon	0 (0, 3.44)	0 (0, 4.44)
50246.88 (0.38, 758081949452940)	1.08 (0.18, 6.18)	466681.49 (0.42, 42894667176582136)	1.01 (0.31, 2.99)	1101.67 (0.29, 9015920925.21)	Suvorexant	0.9 (0.06, 12.71)
59771.47 (0.3, 1003332525970480)	1.19 (0.08, 18.95)	539781.59 (0.37, 54542602011462496)	1.11 (0.1, 12.75)	1256.38 (0.23, 13957377975.9)	1.11 (0.08, 16.44)	Zolpidem

League table for common cold

Doxepin	2.89 (0.49, 26.48)	2.64 (0.23, 40.22)
0.35 (0.04, 2.04)	Placebo	0.89 (0.17, 4.64)
0.38 (0.02, 4.28)	1.12 (0.22, 5.73)	Zolpidem

League table for malaise

Placebo	203430.48 (4.61, 9128285883135182)	1.9 (0.13, 28.09)
0 (0, 0.22)	Ramelteon	0 (0, 0.64)
0.53 (0.04, 7.82)	112263.05 (1.56, 4603890771295450)	Suvorexant

League table for cough

Esmirtazapine	520302368.64 (1.24, 2.1397029779127e+30)	1.17 (0, 91374658498089648128)	1.02 (0.06, 15.19)	0 (0, 0.83)	1.64 (0.06, 48.83)	6.54783177841959e+21 (17654.3, 1.94531515371736e+48)	24633277959.31 (2.05, 6.68141822840215e+29)
0 (0, 0.81)	EVT_201	0 (0, 90473625849067.8)	0 (0, 0.64)	0 (0, 0)	0 (0, 1.19)	1342687814142.89 (0, 1.25773039776356e+41)	14.92 (0, 1.02044343862385e+23)
0.85 (0, 10313587827626854)	1680601473.96 (0, 8.18349487283152e+34)	Lemborexant	0.9 (0, 9305614783073636)	0 (0, 6426933443.91)	1.52 (0, 16135492758975522)	8.7468532968643e+20 (44571.31, 5.58881973554527e+47)	4932321292.18 (6.36, 4.18780696215901e+28)
0.98 (0.07, 16.35)	515843088.2 (1.56, 2.04423088363898e+30)	1.12 (0, 83099821354820370432)	Placebo	0 (0, 0.64)	1.6 (0.26, 11.98)	6.36492753458199e+21 (17779.66, 1.65443593953573e+48)	24275124076.58 (2.43, 5.83041175295986e+29)
11907118906.92 (1.21, 2.26962771715442e+30)	2.21388096124268e+20 (5492.35, 5.81771713969503e+48)	563605114932.83 (0, 2.54030154605267e+37)	11167909191.69 (1.56, 2.10959280707371e+30)	Ramelteon	19399538429.1 (2.3, 3.44066753431882e+30)	3.01877575556741e+33 (40312023159.82, 3.81475285952478e+64)	1.89001922986101e+22 (32062.59, 1.74807601536426e+48)
0.61 (0.02, 16.76)	317245927.42 (0.84, 1.37858651767231e+30)	0.66 (0, 54186917775886901248)	0.62 (0.08, 3.82)	0 (0, 0.44)	Suvorexant	3.87569941495557e+21 (10796.15, 1.11077079572103e+48)	14433925544.82 (1.36, 3.86634785885907e+29)
0 (0, 0)	0 (0, 3772996555560278)	0 (0, 0)	0 (0, 0)	0 (0, 0)	0 (0, 0)	Zaleplon	0 (0, 0.69)
0 (0, 0.49)	0.07 (0, 1.40506496488535e+22)	0 (0, 0.16)	0 (0, 0.41)	0 (0, 0)	0 (0, 0.74)	11591555869.02 (1.45, 7.18795099458417e+31)	Zolpidem

League table for suicidal ideation

Daridorexant	0 (0, 0.68)	0.06 (0, 195798169675.2)
5806684.85 (1.48, 4.4321189864524e+21)	Placebo	140217.31 (2.54, 5945747927368511)
18.06 (0, 1705324174742885632)	0 (0, 0.39)	Suvorexant

League table for irritability

Placebo	1633.93 (0.35, 334403828844.12)	0.87 (0.02, 33.76)
0 (0, 2.82)	Ramelteon	0 (0, 6.21)
1.15 (0.03, 47.02)	2140.79 (0.16, 447511332329.54)	Zolpidem

League table for falls

Daridorexant	837250565.65 (6.13, 7.08633422788112e+26)	2.56 (0.32, 21.19)	1.95 (0.06, 62.86)	0 (0, 12967082108397.9)
0 (0, 0.16)	Doxepin	0 (0, 0.35)	0 (0, 0.36)	0 (0, 7705080.18)
0.39 (0.05, 3.1)	326608731.12 (2.89, 2.52387749121376e+26)	Placebo	0.76 (0.05, 12.01)	0 (0, 4887578640505.38)
0.51 (0.02, 15.68)	423753533.46 (2.81, 3.79480256091672e+26)	1.31 (0.08, 20.74)	Suvorexant	0 (0, 7109394828994.4)
11484.34 (0, 1.11376346243454e+25)	226170073206880 (0, 8.07512745822011e+39)	30062.29 (0, 2.85273937288083e+25)	22409.29 (0, 2.37778314745812e+25)	Zolpidem

League table for laceration

Doxepin	0.18 (0, 54287239163941120)	0 (0, 0.35)
5.7 (0, 23450124952711782400)	Melatonin	0 (0, 1.86)
22286294.66 (2.84, 1.00368289729877e+24)	5933985.07 (0.54, 8.48077474169396e+21)	Placebo

League table for alanine aminotransferase increased

Daridorexant	0.64 (0, 4075612964035.43)	0 (0, 0.84)	0 (0, 0.02)	0 (0, 0.22)
1.56 (0, 237460358224.14)	Lemborexant	0 (0, 2.38)	0 (0, 0.03)	0 (0, 1.4)
168899.93 (1.19, 19539110313883264)	197243.8 (0.42, 96267844421715596 8)	Placebo	0 (0, 1.6)	0.01 (0, 707878.51)
78513198848 (55.16, 1.21144286170345e+25)	69714914200.01 (29.41, 1.81587469395316e+27)	74918.02 (0.63, 1955657623604847)	Ramelteon	718.94 (0, 9773001300353452)
140127219.71 (4.61, 2.32103183496378e+20)	56331508.65 (0.71, 7.28307754419667e+23)	158.5 (0, 36482703800627.8)	0 (0, 25835733088.14)	Zolpidem

League table for blood creatine phosphokinase increased

Daridorexant	0.52 (0.01, 11.5)	2458372.19 (0.92, 1.10230536494962e+21)	0.69 (0.01, 55.56)	0 (0, 0.3)
1.91 (0.09, 103.26)	Placebo	5096974.47 (3.66, 2.11073634675287e+21)	1.32 (0.08, 28.69)	0 (0, 0.69)
0 (0, 1.09)	0 (0, 0.27)	Ramelteon	0 (0, 0.59)	0 (0, 0)
1.45 (0.02, 187.93)	0.76 (0.03, 12.85)	3840157.71 (1.7, 1.68384994011468e+21)	Tasimelteon	0 (0, 0.7)
3232076367.69 (3.34, 8.11725034088958e+27)	1534149938.4 (1.45, 3.34698632647395e+27)	164613727224525280 (3491.35, 9.03054683778306e+38)	2124855813.23 (1.42, 4.90418571627839e+27)	Zolpidem

League table for weight increased

Doxepin	0 (0, 3.47)	0 (0, 0.38)
4787472.32 (0.29, 5.76012554365722e+22)	Esmirtazapine	0.15 (0.02, 0.76)
34512497.48 (2.63, 4.33832060963542e+23)	6.84 (1.32, 64.45)	Placebo

League table for γ -Glutamyl transferase increased

Daridorexant	0 (0, 4.45)	0 (0, 0.32)	0 (0, 0.55)	0 (0, 0.22)
913217147.79 (0.22, 1.1774151009706e+27)	Esmirtazapine	0.12 (0, 1.32)	0.11 (0, 13.23)	0 (0, 9740500157377.84)
8468023944.9 (3.15, 1.03653330040891e+28)	8.18 (0.76, 321.72)	Placebo	0.98 (0.02, 60.55)	0 (0, 90017730991772)
9168427081.09 (1.83, 1.1422840211759e+28)	9.18 (0.08, 2044.18)	1.02 (0.02, 63.25)	Ramelteon	0 (0, 97062276220850.1)
36550784760237.4 (4.51, 2.90073529658618e+36)	29428.54 (0, 6.13542620341244e+24)	2892 (0, 5.55189809716547e+23)	3181.56 (0, 6.33124646982233e+23)	Zolpidem

League table for hyperglycaemia

Doxepin	0.01 (0, 5.45909128516193e+30)	0 (0, 0.2)	0 (0, 0)
143.84 (0, 2.23148424863244e+36)	Melatonin	0 (0, 0.92)	0 (0, 0)
2859050433316658 (4.9, 1.75271583936211e+48)	40182307317671.9 (1.08, 1.86313772909748e+41)	Placebo	0 (0, 0.03)
2.02066873201764e+32 (18863364.45, 1.09168232041615e+74)	2.62979152104391e+30 (1600408.29, 1.75877371166456e+66)	346573635833272 (32.14, 1.45113545255601e+42)	Tiagabine

League table for dysmenorrhea

Placebo	6.69 (0.08, 885.24)	5.21 (0.09, 334.73)	0.52 (0.01, 8.2)	0.63 (0.01, 25.54)
0.15 (0, 12.96)	Ramelteon	0.76 (0, 354.11)	0.07 (0, 11.86)	0.09 (0, 28.98)
0.19 (0, 10.7)	1.31 (0, 690.07)	Triazolam	0.1 (0, 5.09)	0.12 (0, 18.45)
1.93 (0.12, 93.04)	13.68 (0.08, 7413.71)	10.05 (0.2, 1933.76)	Zaleplon	1.2 (0.03, 75.77)
1.59 (0.04, 102.85)	10.97 (0.03, 6705.74)	8.34 (0.05, 2363.46)	0.83 (0.01, 29.06)	Zolpidem

League table for hypertension

Doxepin	0 (0, 2.31)	0 (0, 0.27)	0 (0, 0)	0 (0, 0)	0 (0, 55420052331268.5)
74313136.78 (0.43, 1.60617576053402e+27)	Esmirtazapine	0.23 (0, 4.18)	0 (0, 0.31)	0 (0, 0.16)	155147.79 (0.19, 8701775396935227392)
359389799.44 (3.75, 7.04131551597424e+27)	4.34 (0.24, 206.64)	Placebo	0 (0, 0.96)	0 (0, 0.49)	726154.11 (1.86, 29302165095235235840)
3530496760604694016 (2369.2, 2.30598975144898e+46)	1399475174.29 (3.23, 5.9939018340534e+28)	262565005.12 (1.04, 1.14580864802167e+28)	Ramelteon	1.68 (0, 2.537762446825 36e+22)	2820370881819990 (708.84, 1.40011297518918e+38)
1908224136959067392 (6513.91, 4.78574462687299e+44)	315249094.79 (6.28, 5.57357341478234e+27)	60693324.03 (2.04, 1.02050840516609e+27)	0.6 (0, 1.644563604803 97e+20)	Tasimelteon	1049978729351180 (1114.45, 7.78094188004315e+36)
241.53 (0, 7.61477832587447e+23)	0 (0, 5.2)	0 (0, 0.54)	0 (0, 0)	0 (0, 0)	Zolpidem

League table for tachycardia

Nitrazepam	5532.23 (0, 499406087029084)	1809.95 (0.39, 67532410803.45)	283334331.61 (21.6, 10698731677524185088)
0 (0, 603311.13)	Placebo	0.72 (0, 209571398.46)	28543.05 (0.63, 11013493827770.1)
0 (0, 2.59)	1.39 (0, 781784484.57)	Triazolam	34986.03 (0.81, 31619501356532.7)
0 (0, 0.05)	0 (0, 1.58)	0 (0, 1.23)	Zolpidem

Appendix 12 Results of inconsistency between direct and indirect comparisons

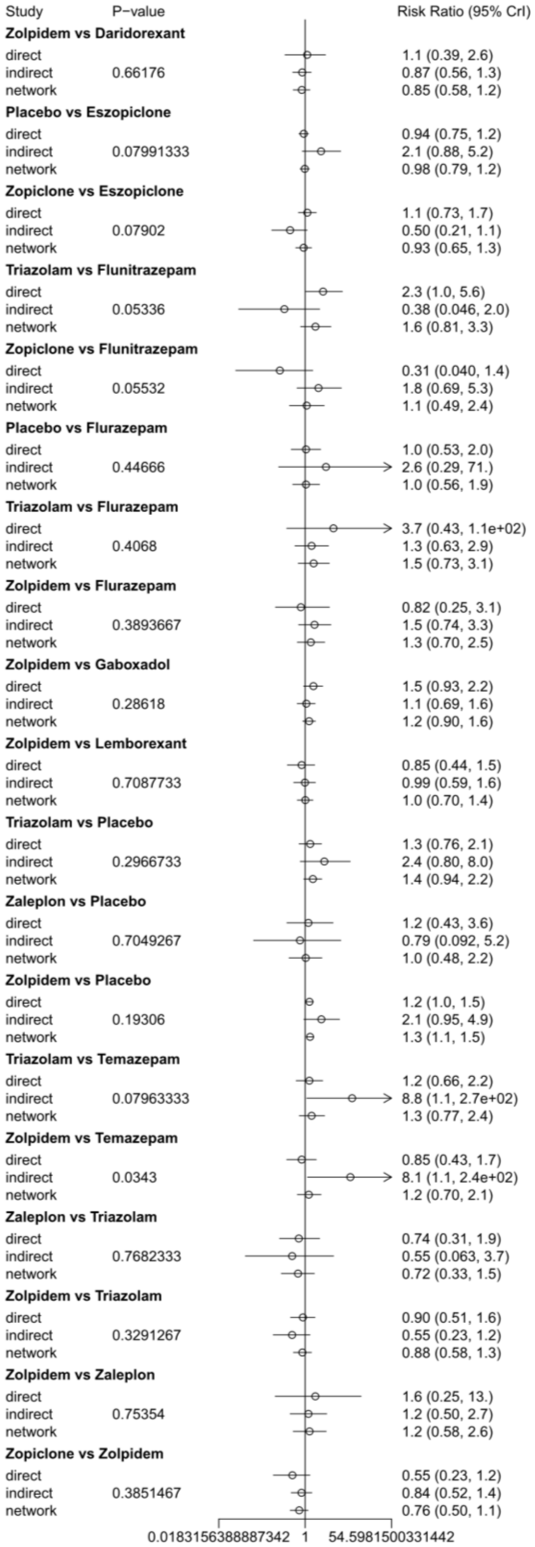

Node split plot for headache

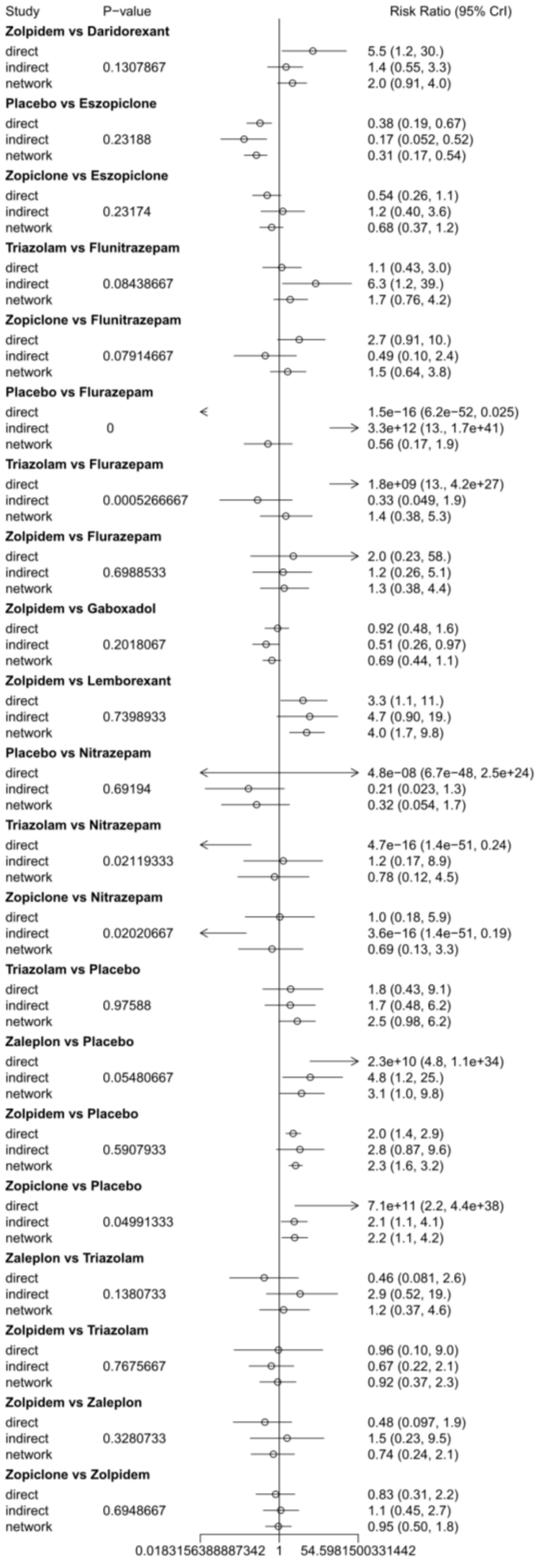

Node split plot for dizziness

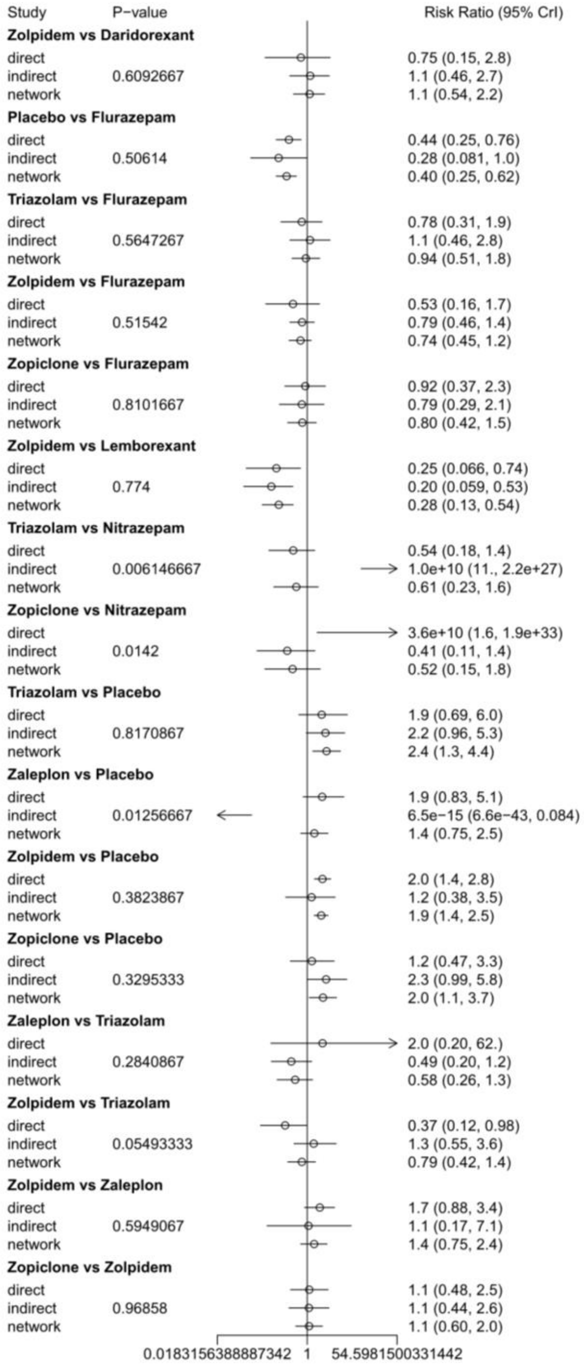

Node split plot for somnolence

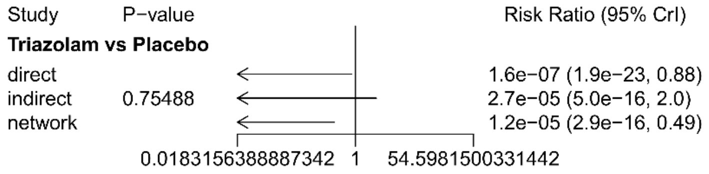

Node split plot for memory disorders

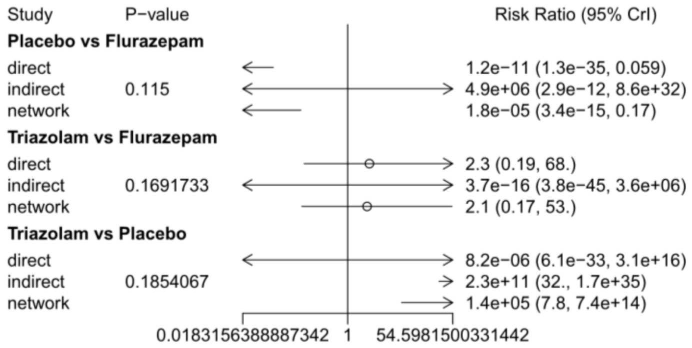

Node split plot for impaired coordination

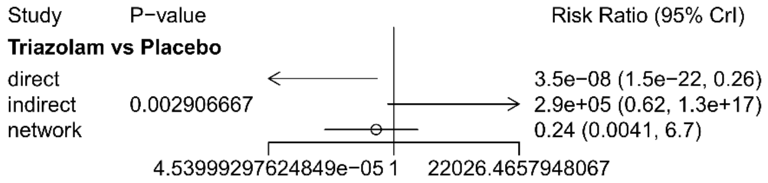

Node split plot for nightmare

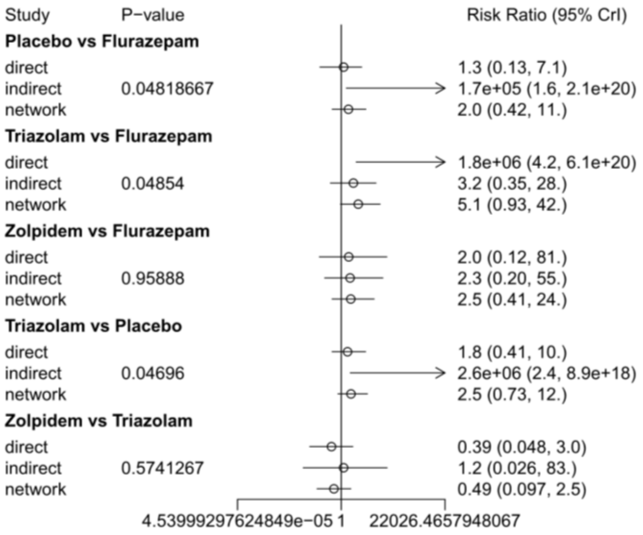

Node split plot for nervousness

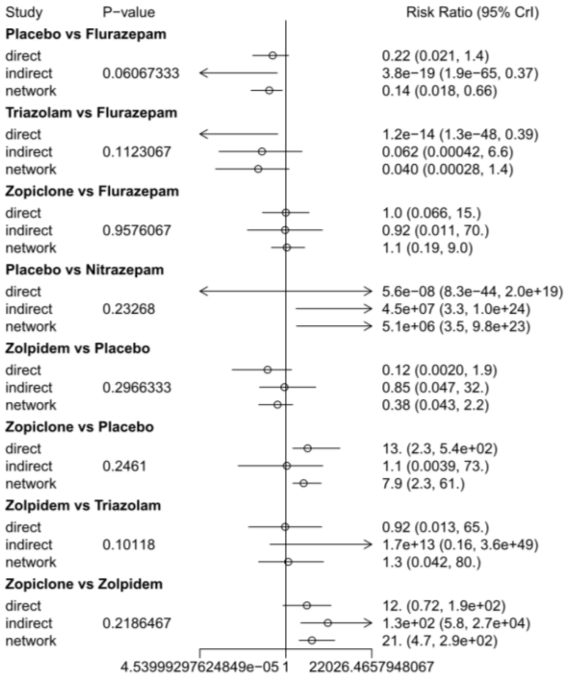

Node split plot for dysgeusia

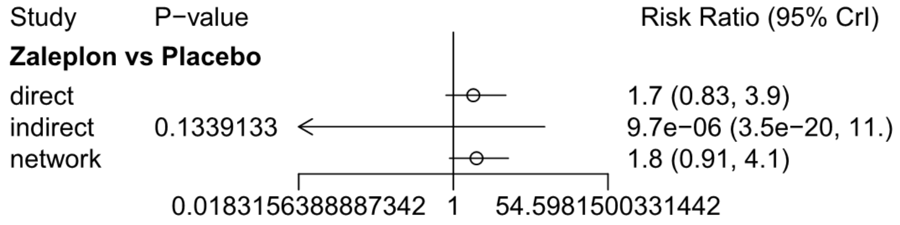

Node split plot for Abdominal pain

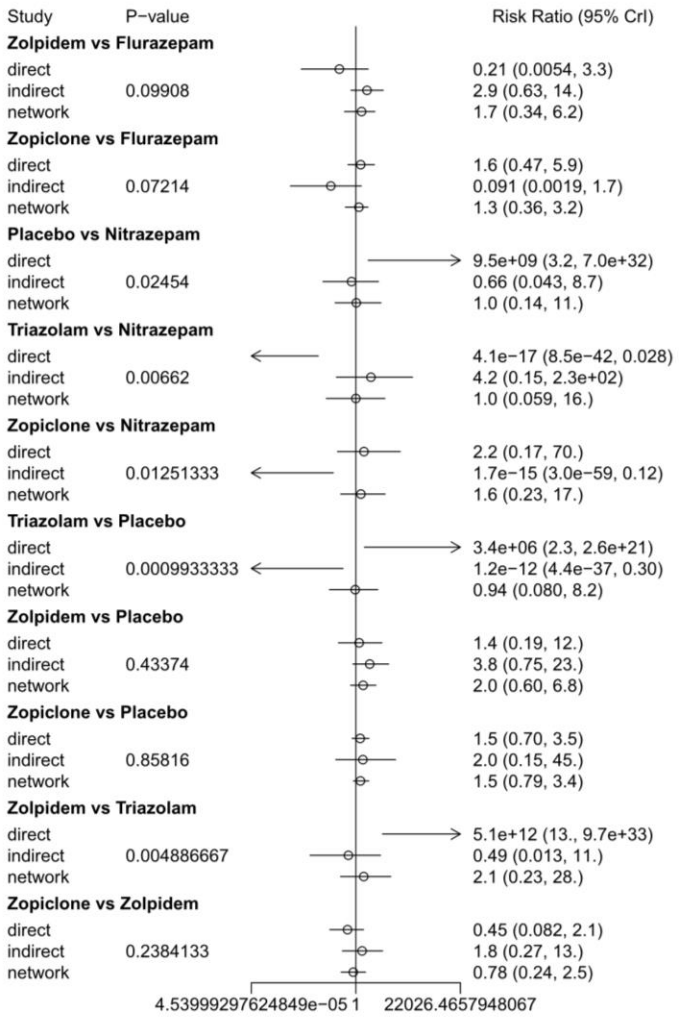

Node split plot for dry mouth

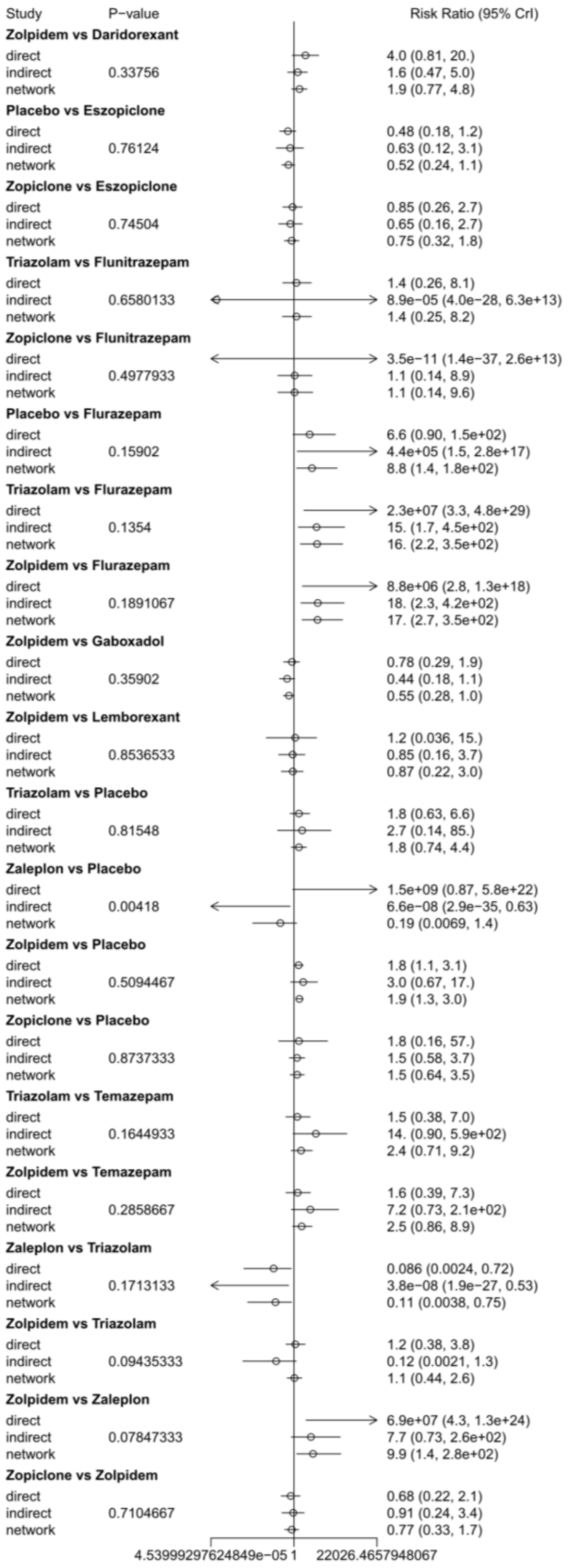

Node split plot for Nausea/vomiting

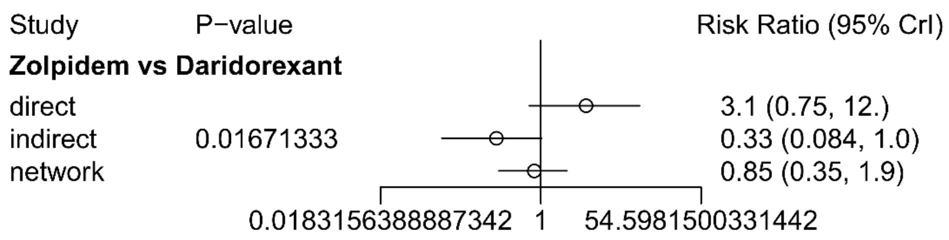

Node split plot for Fatigue

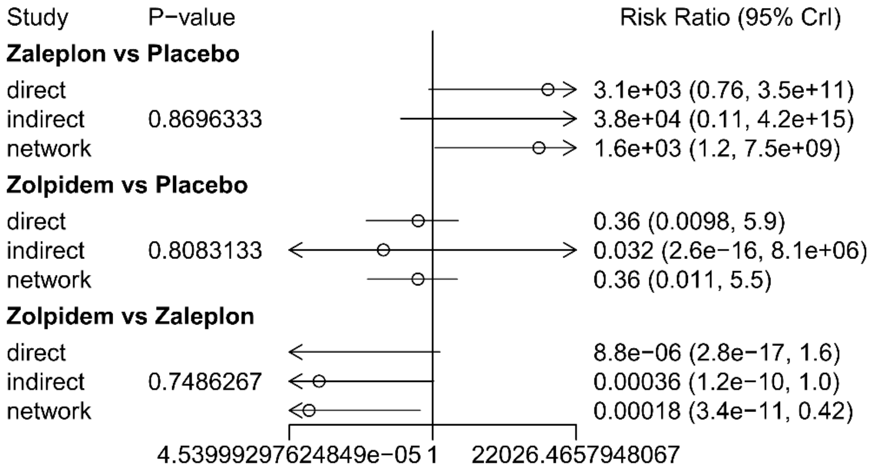

Node split plot for Pain

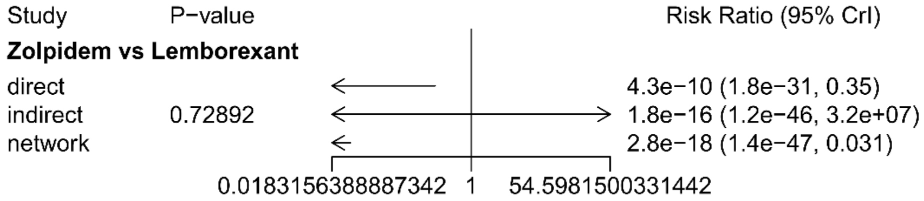

Node split plot for Sleep paralysis

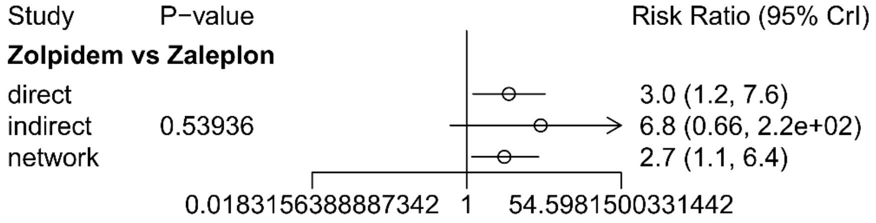

Node split plot for Nasopharyngitis

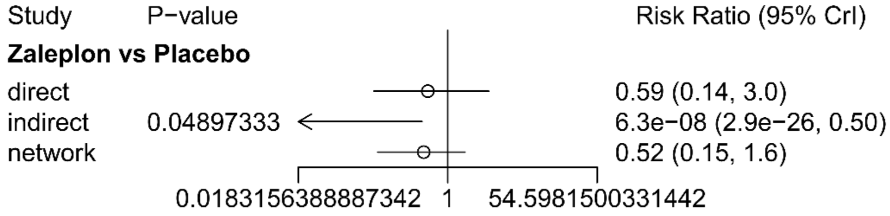

Node split plot for anxiety

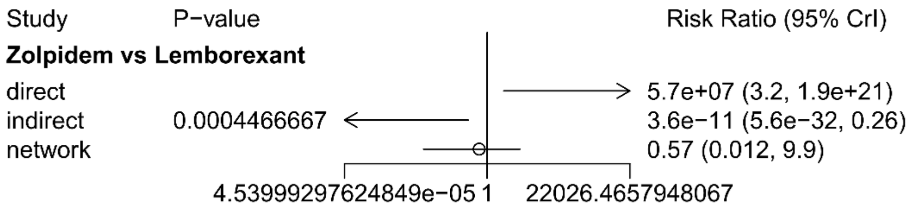

Node split plot for Myalgia

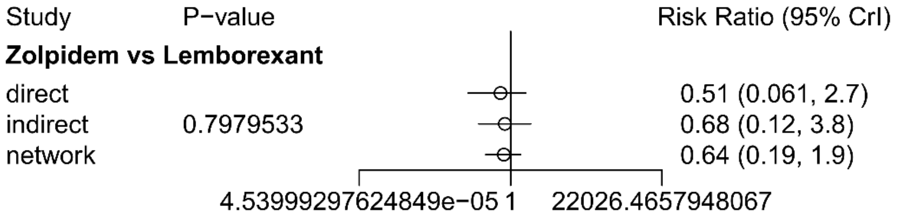

Node split plot for Upper repiratory tract infection

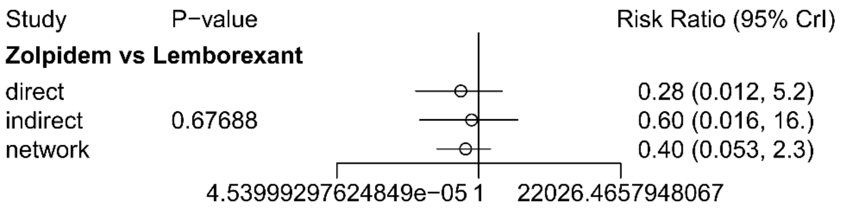

Node split plot for urinary tract infection

Appendix 13 Results of sensitivity analysis

Results of Mantel-Haenszel network meta-analyses

League table for Somnolence

Almorexant	.	.	.	.	.	.	.	.	.	.	.	.	.	.	1.36 (0.43 - 4.32)	.	.	.	.	.	.	.	.	1.28 (0.40 - 4.10)	.
1.16 (0.39 - 3.48)	Daridorexant	.	.	.	.	.	.	.	.	.	.	.	.	.	1.50 (0.80 - 2.81)	.	.	.	.	.	.	.	.	1.18 (0.33 - 4.25)	.
2.07 (0.67 - 6.41)	1.78 (0.75 - 4.20)	Doxepin	.	.	.	.	.	.	.	.	.	.	.	.	0.93 (0.49 - 1.75)	.	.	.	.	.	.	.	.	.	.
0.39 (0.12 - 1.23)	0.34 (0.14 - 0.81)	0.19 (0.08 - 0.47)	Esmirtazapine	.	.	.	.	.	.	.	.	.	.	.	4.92 (2.54 - 9.52)	.	.	.	.	.	.	.	.	.	.
0.63 (0.20 - 1.94)	0.54 (0.23 - 1.27)	0.30 (0.12 - 0.74)	1.61 (0.64 - 4.02)	Estazolam	.	.	.	0.68 (0.37 - 1.25)	.	.	.	.	.	.	3.04 (1.54 - 6.02)	.	.	.	.	.	.	.	.	.	.
0.88 (0.32 - 2.44)	0.75 (0.37 - 1.53)	0.42 (0.20 - 0.90)	2.25 (1.03 - 4.88)	1.40 (0.66 - 2.98)	Eszopiclone	.	.	.	.	.	.	.	.	.	2.19 (1.46 - 3.29)	.	.	.	.	.	.	.	.	.	.
0.54 (0.05 - 5.45)	0.47 (0.05 - 4.15)	0.26 (0.03 - 2.37)	1.39 (0.15 - 12.65)	0.86 (0.10 - 7.83)	0.62 (0.07 - 5.29)	EVT_201	.	.	.	.	.	.	.	.	3.55 (0.43 - 29.24)	.	.	.	.	.	.	.	.	.	.
0.46 (0.13 - 1.69)	0.40 (0.13 - 1.17)	0.22 (0.07 - 0.68)	1.18 (0.38 - 3.69)	0.74 (0.25 - 2.13)	0.53 (0.19 - 1.45)	0.85 (0.09 - 8.53)	Flunitrazepam	.	.	.	.	.	.	.	.	.	.	.	.	.	.	1.35 (0.72 - 2.50)	.	.	.
0.42 (0.14 - 1.23)	0.36 (0.16 - 0.80)	0.20 (0.09 - 0.47)	1.08 (0.46 - 2.55)	0.67 (0.37 - 1.22)	0.48 (0.24 - 0.95)	0.78 (0.09 - 6.89)	0.92 (0.35 - 2.40)	Flurazepam	.	.	.	.	.	.	4.72 (2.46 - 9.03)	.	.	.	.	.	.	1.44 (0.48 - 4.29)	.	2.13 (0.63 - 7.17)	.
0.94 (0.26 - 3.41)	0.80 (0.27 - 2.36)	0.45 (0.15 - 1.37)	2.40 (0.78 - 7.39)	1.49 (0.49 - 4.52)	1.07 (0.39 - 2.90)	1.73 (0.17 - 17.21)	2.03 (0.56 - 7.32)	2.22 (0.77 - 6.34)	Gaboxadol	.	.	.	.	.	1.27 (0.42 - 3.88)	.	.	.	.	.	.	.	.	1.81 (0.52 - 6.30)	.
0.55 (0.16 - 1.83)	0.47 (0.18 - 1.22)	0.26 (0.10 - 0.71)	1.40 (0.51 - 3.83)	0.87 (0.32 - 2.35)	0.62 (0.26 - 1.48)	1.01 (0.11 - 9.49)	1.18 (0.36 - 3.92)	1.29 (0.50 - 3.30)	0.58 (0.18 - 1.91)	Indiplon	.	.	.	.	3.52 (1.64 - 7.57)	.	.	.	.	.	.	.	.	.	.
0.29 (0.10 - 0.88)	0.25 (0.11 - 0.58)	0.14 (0.06 - 0.34)	0.75 (0.31 - 1.83)	0.47 (0.19 - 1.12)	0.33 (0.16 - 0.69)	0.54 (0.06 - 4.84)	0.63 (0.21 - 1.89)	0.69 (0.31 - 1.55)	0.31 (0.11 - 0.92)	0.54 (0.20 - 1.42)	Lemborexant	.	.	.	6.42 (3.24 - 12.74)	.	.	.	.	.	.	.	.	3.85 (1.34 - 11.06)	.
0.72 (0.23 - 2.31)	0.62 (0.25 - 1.56)	0.35 (0.13 - 0.91)	1.85 (0.70 - 4.93)	1.15 (0.47 - 2.81)	0.82 (0.36 - 1.89)	1.34 (0.14 - 12.41)	1.57 (0.81 - 3.02)	1.71 (0.79 - 3.69)	0.77 (0.25 - 2.42)	1.33 (0.46 - 3.80)	2.48 (0.98 - 6.26)	Loprazolam	.	.	.	.	.	.	.	.	.	0.86 (0.69 - 1.06)	.	.	.
0.32 (0.05 - 1.89)	0.27 (0.05 - 1.40)	0.15 (0.03 - 0.81)	0.81 (0.15 - 4.31)	0.50 (0.10 - 2.60)	0.36 (0.07 - 1.77)	0.58 (0.04 - 7.93)	0.68 (0.13 - 3.57)	0.75 (0.15 - 3.67)	0.34 (0.06 - 1.99)	0.58 (0.10 - 3.23)	1.08 (0.21 - 5.59)	0.44 (0.09 - 2.05)	Mldazolam	.	.	.	.	.	1.31 (0.38 - 4.46)	.	.	.	.	.	.
0.27 (0.05 - 1.36)	0.23 (0.05 - 0.99)	0.13 (0.03 - 0.57)	0.68 (0.15 - 3.07)	0.42 (0.10 - 1.81)	0.30 (0.07 - 1.25)	0.49 (0.04 - 6.01)	0.57 (0.15 - 2.16)	0.63 (0.16 - 2.50)	0.28 (0.06 - 1.43)	0.49 (0.10 - 2.31)	0.91 (0.21 - 3.98)	0.37 (0.11 - 1.20)	0.84 (0.12 - 5.78)	Nitrazepam	.	.	.	.	.	.	.	2.34 (0.73 - 7.55)	.	.	.
1.92 (0.75 - 4.91)	1.65 (0.92 - 2.95)	0.93 (0.49 - 1.75)	4.92 (2.54 - 9.52)	3.06 (1.62 - 5.80)	2.19 (1.46 - 3.29)	3.55 (0.43 - 29.24)	4.16 (1.65 - 10.51)	4.55 (2.63 - 7.85)	2.05 (0.82 - 5.10)	3.52 (1.64 - 7.57)	6.58 (3.59 - 12.06)	2.66 (1.29 - 5.46)	6.09 (1.31 - 28.37)	7.25 (1.86 - 28.18)	Placebo	0.47 (0.32 - 0.70)	0.57 (0.07 - 4.96)	0.28 (0.20 - 0.41)	0.27 (0.07 - 1.03)	0.98 (0.20 - 4.79)	0.27 (0.12 - 0.65)	0.51 (0.18 - 1.43)	0.51 (0.22 - 1.17)	0.46 (0.33 - 0.64)	0.27 (0.02 - 3.09)
0.91 (0.33 - 2.52)	0.78 (0.38 - 1.58)	0.44 (0.21 - 0.93)	2.32 (1.07 - 5.03)	1.45 (0.68 - 3.08)	1.03 (0.58 - 1.83)	1.68 (0.20 - 14.34)	1.97 (0.72 - 5.39)	2.15 (1.09 - 4.23)	0.97 (0.36 - 2.62)	1.67 (0.70 - 3.95)	3.11 (1.50 - 6.42)	1.25 (0.55 - 2.86)	2.88 (0.59 - 14.10)	3.42 (0.83 - 14.10)	0.47 (0.32 - 0.70)	Ramelteon	.	.	.	.	.	.	.	.	.
1.91 (0.40 - 9.17)	1.64 (0.40 - 6.68)	0.92 (0.22 - 3.88)	4.90 (1.15 - 20.80)	3.05 (0.73 - 12.71)	2.18 (0.57 - 8.41)	3.53 (0.30 - 41.79)	4.14 (0.87 - 19.74)	4.53 (1.14 - 18.04)	2.04 (0.43 - 9.67)	3.51 (0.79 - 15.68)	6.55 (1.61 - 26.72)	2.64 (0.62 - 11.26)	6.06 (0.83 - 44.12)	7.22 (1.13 - 45.89)	1.00 (0.27 - 3.61)	2.11 (0.55 - 8.11)	Seltorexant	.	.	.	.	.	.	0.41 (0.11 - 1.57)	.

0.55 (0.20 - 1.50)	0.47 (0.23 - 0.94)	0.26 (0.13 - 0.55)	1.40 (0.65 - 2.98)	0.87 (0.41 - 1.82)	0.62 (0.36 - 1.08)	1.01 (0.12 - 8.58)	1.18 (0.44 - 3.21)	1.29 (0.67 - 2.50)	0.58 (0.22 - 1.56)	1.00 (0.43 - 2.34)	1.87 (0.92 - 3.80)	0.75 (0.33 - 1.70)	1.73 (0.35 - 8.42)	2.06 (0.50 - 8.41)	0.28 (0.20 - 0.41)	0.60 (0.35 - 1.04)	0.29 (0.07 - 1.09)	Suvorexant	.	.	.	.	.	.	.
0.41 (0.11 - 1.51)	0.35 (0.12 - 1.05)	0.20 (0.06 - 0.61)	1.06 (0.34 - 3.31)	0.66 (0.22 - 1.96)	0.47 (0.17 - 1.30)	0.76 (0.08 - 7.64)	0.89 (0.29 - 2.71)	0.98 (0.35 - 2.70)	0.44 (0.12 - 1.59)	0.76 (0.23 - 2.52)	1.41 (0.47 - 4.23)	0.57 (0.22 - 1.47)	1.31 (0.38 - 4.46)	1.56 (0.35 - 6.90)	0.21 (0.08 - 0.54)	0.45 (0.17 - 1.25)	0.22 (0.05 - 1.03)	0.76 (0.28 - 2.06)	Temazepam	.	.	1.51 (0.54 - 4.16)	.	2.64 (0.79 - 8.77)	.
1.89 (0.30 - 11.91)	1.62 (0.30 - 8.77)	0.91 (0.17 - 5.03)	4.84 (0.87 - 26.90)	3.01 (0.55 - 16.62)	2.15 (0.42 - 11.05)	3.49 (0.25 - 48.77)	4.09 (0.65 - 25.63)	4.47 (0.84 - 23.88)	2.02 (0.32 - 12.54)	3.47 (0.60 - 20.12)	6.47 (1.19 - 35.25)	2.61 (0.46 - 14.88)	5.99 (0.66 - 54.47)	7.13 (0.88 - 57.40)	0.98 (0.20 - 4.79)	2.08 (0.41 - 10.66)	0.99 (0.13 - 7.60)	3.46 (0.68 - 17.64)	4.58 (0.73 - 28.73)	Tiagabine	.	.	.	.	.
0.55 (0.18 - 1.69)	0.47 (0.20 - 1.13)	0.27 (0.11 - 0.67)	1.41 (0.55 - 3.60)	0.88 (0.35 - 2.18)	0.63 (0.29 - 1.37)	1.01 (0.11 - 9.27)	1.19 (0.39 - 3.62)	1.30 (0.56 - 3.02)	0.59 (0.19 - 1.77)	1.01 (0.37 - 2.78)	1.88 (0.78 - 4.53)	0.76 (0.29 - 1.96)	1.74 (0.33 - 9.12)	2.07 (0.47 - 9.21)	0.29 (0.15 - 0.56)	0.61 (0.28 - 1.32)	0.29 (0.07 - 1.18)	1.01 (0.47 - 2.16)	1.33 (0.44 - 4.06)	0.29 (0.05 - 1.62)	Trazodone	.	.	1.61 (0.79 - 3.27)	.
0.62 (0.20 - 1.95)	0.53 (0.22 - 1.30)	0.30 (0.12 - 0.76)	1.59 (0.61 - 4.13)	0.99 (0.42 - 2.35)	0.71 (0.32 - 1.58)	1.15 (0.12 - 10.54)	1.35 (0.72 - 2.50)	1.47 (0.70 - 3.07)	0.66 (0.22 - 2.04)	1.14 (0.41 - 3.19)	2.13 (0.86 - 5.24)	0.86 (0.69 - 1.06)	1.97 (0.42 - 9.11)	2.34 (0.73 - 7.55)	0.32 (0.16 - 0.64)	0.68 (0.31 - 1.52)	0.32 (0.08 - 1.36)	1.14 (0.52 - 2.49)	1.50 (0.60 - 3.78)	0.33 (0.06 - 1.85)	1.13 (0.45 - 2.85)	Triazolam	0.71 (0.07 - 7.12)	2.07 (0.76 - 5.67)	.
1.38 (0.48 - 3.98)	1.19 (0.54 - 2.62)	0.67 (0.29 - 1.56)	3.53 (1.48 - 8.41)	2.20 (0.96 - 5.07)	1.57 (0.79 - 3.15)	2.55 (0.29 - 22.61)	2.99 (1.06 - 8.43)	3.27 (1.54 - 6.94)	1.47 (0.52 - 4.17)	2.53 (0.98 - 6.54)	4.72 (2.12 - 10.53)	1.91 (0.81 - 4.50)	4.37 (0.87 - 21.88)	5.21 (1.24 - 21.87)	0.72 (0.41 - 1.26)	1.52 (0.76 - 3.03)	0.72 (0.18 - 2.82)	2.53 (1.29 - 4.97)	3.34 (1.18 - 9.50)	0.73 (0.14 - 3.92)	2.51 (1.11 - 5.69)	2.22 (0.97 - 5.10)	Zaleplon	0.57 (0.32 - 1.02)	.
0.91 (0.35 - 2.31)	0.78 (0.41 - 1.47)	0.44 (0.22 - 0.89)	2.32 (1.11 - 4.82)	1.44 (0.72 - 2.87)	1.03 (0.61 - 1.73)	1.67 (0.20 - 14.10)	1.96 (0.77 - 4.96)	2.14 (1.19 - 3.85)	0.97 (0.39 - 2.42)	1.66 (0.72 - 3.80)	3.10 (1.63 - 5.87)	1.25 (0.61 - 2.58)	2.87 (0.62 - 13.33)	3.41 (0.88 - 13.28)	0.47 (0.34 - 0.65)	1.00 (0.60 - 1.66)	0.47 (0.13 - 1.67)	1.66 (1.02 - 2.71)	2.19 (0.87 - 5.54)	0.48 (0.10 - 2.41)	1.65 (0.86 - 3.14)	1.46 (0.73 - 2.91)	0.66 (0.39 - 1.10)	Zolpidem	0.91 (0.47 - 1.75)
0.80 (0.26 - 2.46)	0.68 (0.28 - 1.67)	0.38 (0.15 - 0.99)	2.03 (0.78 - 5.33)	1.27 (0.50 - 3.22)	0.91 (0.40 - 2.04)	1.47 (0.16 - 13.55)	1.72 (0.56 - 5.29)	1.88 (0.80 - 4.44)	0.85 (0.28 - 2.59)	1.46 (0.52 - 4.12)	2.72 (1.11 - 6.68)	1.10 (0.42 - 2.87)	2.52 (0.48 - 13.27)	3.00 (0.67 - 13.42)	0.41 (0.20 - 0.83)	0.88 (0.39 - 1.96)	0.42 (0.10 - 1.71)	1.46 (0.66 - 3.23)	1.93 (0.63 - 5.91)	0.42 (0.07 - 2.38)	1.45 (0.59 - 3.57)	1.28 (0.50 - 3.26)	0.58 (0.25 - 1.31)	0.88 (0.46 - 1.66)	Zopiclone

Leaguetable for Dizziness

Almorexant	.	.	.	.	.	.	.	.	.	.	.	.	.	.	0.67 (0.29 - 1.54)	.	.	.	.	.	.	2.21 (0.63 - 7.81)	.
0.24 (0.05 - 1.21)	Alprazolam	.	.	.	.	.	.	.	.	.	.	.	.	7.00 (1.38 - 35.48)	4.50 (1.09 - 18.50)	.	.	.	.	.	.	.	.
0.95 (0.33 - 2.70)	3.96 (0.81 - 19.22)	Daridorexant	.	.	.	.	.	.	.	.	.	.	.	.	1.41 (0.66 - 3.00)	.	.	.	.	.	.	0.18 (0.04 - 0.82)	.
0.89 (0.18 - 4.31)	3.69 (0.51 - 26.56)	0.93 (0.20 - 4.38)	Doxepin	.	.	.	.	.	.	.	.	.	.	.	1.22 (0.31 - 4.83)	.	.	.	.	.	.	.	.
0.40 (0.12 - 1.27)	1.65 (0.31 - 8.65)	0.42 (0.14 - 1.27)	0.45 (0.09 - 2.27)	Esmirtazapine	.	.	.	.	.	.	.	.	.	.	2.73 (1.15 - 6.48)	.	.	.	.	.	.	.	.
1.91 (0.10 - 36.80)	7.95 (0.33 - 194.06)	2.01 (0.11 - 38.11)	2.16 (0.09 - 51.74)	4.82 (0.24 - 96.16)	Estazolam	.	.	0.39 (0.07 - 2.10)	.	.	.	.	.	.	.	.	.	.	.	.	.	.	.
0.35 (0.14 - 0.91)	1.46 (0.32 - 6.68)	0.37 (0.15 - 0.90)	0.40 (0.09 - 1.75)	0.89 (0.32 - 2.48)	0.18 (0.01 - 3.37)	Eszopiclone	.	.	.	.	.	.	.	.	2.70 (1.45 - 5.05)	.	.	.	.	.	.	.	2.03 (0.94 - 4.36)
1.09 (0.28 - 4.15)	4.52 (0.75 - 27.24)	1.14 (0.31 - 4.21)	1.22 (0.21 - 7.17)	2.74 (0.67 - 11.18)	0.57 (0.03 - 11.97)	3.09 (1.00 - 9.58)	Flunitrazepam	.	.	.	.	.	.	.	.	.	.	.	.	0.88 (0.33 - 2.35)	.	.	0.33 (0.10 - 1.14)
0.76 (0.07 - 8.67)	3.14 (0.21 - 47.86)	0.79 (0.07 - 8.95)	0.85 (0.06 - 12.72)	1.91 (0.16 - 22.83)	0.39 (0.07 - 2.10)	2.15 (0.20 - 23.25)	0.70 (0.05 - 8.92)	Flurazepam	.	.	.	.	.	.	.	.	.	.	.	.	.	0.64 (0.06 - 6.36)	.
0.32 (0.13 - 0.75)	1.31 (0.30 - 5.74)	0.33 (0.15 - 0.75)	0.36 (0.08 - 1.50)	0.80 (0.30 - 2.08)	0.17 (0.01 - 2.94)	0.90 (0.45 - 1.78)	0.29 (0.09 - 0.93)	0.42 (0.04 - 4.36)	Gaboxadol	.	.	.	.	.	4.16 (2.55 - 6.77)	.	.	.	.	.	.	1.08 (0.58 - 2.03)	.
0.48 (0.18 - 1.30)	1.99 (0.42 - 9.36)	0.50 (0.20 - 1.30)	0.54 (0.12 - 2.45)	1.21 (0.41 - 3.52)	0.25 (0.01 - 4.70)	1.36 (0.59 - 3.15)	0.44 (0.12 - 1.58)	0.63 (0.06 - 7.06)	1.52 (0.71 - 3.22)	Indiplon	.	.	.	.	2.26 (1.21 - 4.24)	.	.	.	.	.	.	.	.
1.96 (0.62 - 6.18)	8.17 (1.56 - 42.71)	2.06 (0.69 - 6.22)	2.21 (0.44 - 11.21)	4.95 (1.46 - 16.76)	1.03 (0.05 - 20.05)	5.59 (2.03 - 15.36)	1.81 (0.45 - 7.20)	2.60 (0.22 - 30.39)	6.22 (2.45 - 15.81)	4.10 (1.42 - 11.88)	Lemborexant	.	.	.	0.48 (0.18 - 1.25)	.	.	.	.	.	.	0.30 (0.10 - 0.93)	.
2.90 (0.26 - 32.64)	12.08 (0.82 - 178.45)	3.05 (0.28 - 33.59)	3.27 (0.23 - 47.43)	7.32 (0.63 - 84.87)	1.52 (0.04 - 59.50)	8.26 (0.78 - 87.32)	2.67 (0.21 - 34.12)	3.84 (0.15 - 100.81)	9.19 (0.89 - 94.46)	6.06 (0.56 - 65.30)	1.48 (0.13 - 17.09)	Mldazolam	.	.	.	.	.	1.07 (0.26 - 4.44)	.	.	.	.	.
0.40 (0.05 - 3.07)	1.67 (0.16 - 17.68)	0.42 (0.06 - 3.15)	0.45 (0.04 - 4.69)	1.01 (0.13 - 8.09)	0.21 (0.01 - 6.37)	1.14 (0.18 - 7.42)	0.37 (0.05 - 2.84)	0.53 (0.03 - 10.42)	1.27 (0.19 - 8.68)	0.84 (0.11 - 6.14)	0.20 (0.03 - 1.61)	0.14 (0.01 - 2.70)	Nitrazepam	.	.	.	.	.	.	.	.	.	1.46 (0.25 - 8.50)
1.68 (0.22 - 12.69)	7.00 (1.38 - 35.48)	1.77 (0.24 - 13.01)	1.90 (0.19 - 19.28)	4.25 (0.54 - 33.19)	0.88 (0.03 - 26.86)	4.79 (0.68 - 33.53)	1.55 (0.18 - 13.58)	2.23 (0.11 - 44.00)	5.33 (0.79 - 36.05)	3.51 (0.49 - 25.17)	0.86 (0.11 - 6.68)	0.58 (0.03 - 11.14)	4.19 (0.29 - 59.49)	Paroxetine	0.64 (0.10 - 4.15)	.	.	.	.	.	.	.	.
1.08 (0.50 - 2.35)	4.50 (1.09 - 18.50)	1.14 (0.56 - 2.31)	1.22 (0.31 - 4.83)	2.73 (1.15 - 6.48)	0.57 (0.03 - 9.92)	3.08 (1.76 - 5.37)	1.00 (0.33 - 3.02)	1.43 (0.14 - 14.69)	3.43 (2.26 - 5.20)	2.26 (1.21 - 4.24)	0.55 (0.23 - 1.30)	0.37 (0.04 - 3.69)	2.69 (0.41 - 17.77)	0.64 (0.10 - 4.15)	Placebo	0.80 (0.51 - 1.23)	0.97 (0.58 - 1.64)	2.88 (0.48 - 17.45)	0.25 (0.06 - 1.07)	1.67 (0.22 - 12.60)	.	0.49 (0.34 - 0.72)	.
0.86 (0.35 - 2.10)	3.58 (0.81 - 15.73)	0.90 (0.39 - 2.08)	0.97 (0.23 - 4.11)	2.17 (0.82 - 5.73)	0.45 (0.02 - 8.16)	2.45 (1.20 - 4.97)	0.79 (0.24 - 2.61)	1.14 (0.11 - 12.17)	2.72 (1.49 - 4.99)	1.80 (0.83 - 3.87)	0.44 (0.17 - 1.15)	0.30 (0.03 - 3.06)	2.14 (0.31 - 14.86)	0.51 (0.08 - 3.47)	0.80 (0.51 - 1.23)	Ramelteon	.	.	.	.	.	.	.
1.05 (0.41 - 2.69)	4.38 (0.97 - 19.79)	1.11 (0.46 - 2.67)	1.19 (0.27 - 5.18)	2.66 (0.97 - 7.31)	0.55 (0.03 - 10.13)	2.99 (1.39 - 6.43)	0.97 (0.28 - 3.31)	1.39 (0.13 - 15.15)	3.33 (1.70 - 6.52)	2.20 (0.97 - 4.99)	0.54 (0.20 - 1.47)	0.36 (0.03 - 3.81)	2.62 (0.37 - 18.58)	0.63 (0.09 - 4.34)	0.97 (0.58 - 1.64)	1.22 (0.62 - 2.43)	Suvorexant	.	.	.	.	.	.
3.12 (0.44 - 22.16)	12.98 (1.32 - 128.06)	3.28 (0.47 - 22.69)	3.52 (0.36 - 33.92)	7.87 (1.07 - 58.02)	1.63 (0.06 - 48.08)	8.87 (1.35 - 58.40)	2.87 (0.35 - 23.82)	4.13 (0.22 - 78.36)	9.88 (1.56 - 62.71)	6.52 (0.97 - 43.87)	1.59 (0.22 - 11.67)	1.07 (0.26 - 4.44)	7.76 (0.57 - 105.40)	1.85 (0.14 - 24.78)	2.88 (0.48 - 17.45)	3.63 (0.57 - 23.14)	2.96 (0.45 - 19.33)	Temazepam	.	.	.	.	.
0.27 (0.05 - 1.41)	1.14 (0.15 - 8.60)	0.29 (0.06 - 1.44)	0.31 (0.04 - 2.27)	0.69 (0.13 - 3.72)	0.14 (0.01 - 3.54)	0.78 (0.16 - 3.66)	0.25 (0.04 - 1.56)	0.36 (0.02 - 5.61)	0.86 (0.19 - 3.90)	0.57 (0.12 - 2.77)	0.14 (0.03 - 0.75)	0.09 (0.01 - 1.42)	0.68 (0.06 - 7.33)	0.16 (0.02 - 1.72)	0.25 (0.06 - 1.07)	0.32 (0.07 - 1.44)	0.26 (0.06 - 1.21)	0.09 (0.01 - 0.88)	Tiagabine	.	.	.	.

0.71 (0.18 - 2.70)	2.93 (0.49 - 17.71)	0.74 (0.20 - 2.74)	0.80 (0.14 - 4.66)	1.78 (0.44 - 7.27)	0.37 (0.02 - 7.78)	2.01 (0.63 - 6.43)	0.65 (0.27 - 1.57)	0.93 (0.07 - 11.97)	2.23 (0.70 - 7.15)	1.47 (0.41 - 5.28)	0.36 (0.09 - 1.43)	0.24 (0.02 - 3.10)	1.76 (0.22 - 14.07)	0.42 (0.05 - 3.68)	0.65 (0.21 - 1.98)	0.82 (0.25 - 2.71)	0.67 (0.20 - 2.29)	0.23 (0.03 - 1.87)	2.59 (0.42 - 16.05)	Triazolam	2.29 (0.43 - 12.03)	2.00 (0.19 - 21.07)	.
0.53 (0.13 - 2.23)	2.22 (0.34 - 14.40)	0.56 (0.14 - 2.27)	0.60 (0.10 - 3.80)	1.34 (0.30 - 6.03)	0.28 (0.01 - 6.08)	1.52 (0.41 - 5.58)	0.49 (0.12 - 1.96)	0.71 (0.05 - 9.41)	1.69 (0.48 - 5.98)	1.11 (0.28 - 4.41)	0.27 (0.06 - 1.18)	0.18 (0.01 - 2.47)	1.33 (0.15 - 11.83)	0.32 (0.03 - 2.95)	0.49 (0.14 - 1.68)	0.62 (0.17 - 2.28)	0.51 (0.13 - 1.92)	0.17 (0.02 - 1.51)	1.95 (0.29 - 13.03)	0.76 (0.21 - 2.66)	Zaleplon	2.10 (0.49 - 8.96)	.
0.48 (0.21 - 1.09)	2.00 (0.47 - 8.62)	0.51 (0.24 - 1.09)	0.54 (0.13 - 2.25)	1.22 (0.48 - 3.10)	0.25 (0.01 - 4.32)	1.37 (0.74 - 2.55)	0.44 (0.15 - 1.34)	0.64 (0.06 - 6.36)	1.53 (0.97 - 2.41)	1.01 (0.49 - 2.08)	0.25 (0.10 - 0.59)	0.17 (0.02 - 1.69)	1.20 (0.18 - 7.91)	0.29 (0.04 - 1.91)	0.45 (0.31 - 0.64)	0.56 (0.32 - 0.99)	0.46 (0.24 - 0.86)	0.15 (0.02 - 0.97)	1.77 (0.40 - 7.86)	0.68 (0.23 - 2.06)	0.90 (0.27 - 2.98)	Zolpidem	1.21 (0.46 - 3.19)
0.59 (0.21 - 1.63)	2.45 (0.51 - 11.77)	0.62 (0.23 - 1.63)	0.66 (0.14 - 3.08)	1.48 (0.49 - 4.47)	0.31 (0.02 - 5.72)	1.67 (0.89 - 3.15)	0.54 (0.19 - 1.51)	0.78 (0.07 - 8.57)	1.86 (0.86 - 4.02)	1.23 (0.49 - 3.11)	0.30 (0.10 - 0.87)	0.20 (0.02 - 2.22)	1.46 (0.25 - 8.50)	0.35 (0.05 - 2.55)	0.54 (0.27 - 1.08)	0.68 (0.30 - 1.54)	0.56 (0.24 - 1.32)	0.19 (0.03 - 1.29)	2.16 (0.43 - 10.70)	0.83 (0.27 - 2.54)	1.10 (0.30 - 4.05)	1.22 (0.62 - 2.41)	Zopiclone

Leaguetable for Headache

Almorexant	.	.	.	.	.	.	.	.	.	.	.	.	.	.	1.03 (0.59 - 1.79)	.	.	.	.	.	.	.	.	.	.	.	0.66 (0.40 - 1.10)	.
0.65 (0.37 - 1.15)	Daridorexant	.	.	.	.	.	.	.	.	.	.	.	.	.	1.57 (1.07 - 2.31)	.	.	.	.	.	.	.	.	.	.	.	0.87 (0.33 - 2.25)	.
1.91 (0.90 - 4.07)	2.95 (1.46 - 5.98)	Doxepin	.	.	.	.	.	.	.	.	.	.	.	.	0.49 (0.27 - 0.89)	.	.	.	.	.	.	.	.	.	.	.	.	.
1.32 (0.68 - 2.57)	2.05 (1.12 - 3.75)	0.69 (0.32 - 1.51)	Esmirtazapine	.	.	.	.	.	.	.	.	.	.	.	0.70 (0.43 - 1.14)	.	.	.	.	.	.	.	.	.	.	.	.	.
1.40 (0.51 - 3.88)	2.17 (0.81 - 5.79)	0.73 (0.24 - 2.20)	1.06 (0.38 - 2.99)	Estazolam	.	.	0.72 (0.27 - 1.89)	.	.	.	.	.	.	.	0.62 (0.24 - 1.61)	.	.	.	.	.	.	.	.	.	.	.	.	.
0.90 (0.55 - 1.49)	1.40 (0.91 - 2.14)	0.47 (0.25 - 0.90)	0.68 (0.40 - 1.17)	0.64 (0.25 - 1.66)	Eszopiclone	.	.	.	.	.	.	.	.	.	1.08 (0.85 - 1.36)	.	.	.	.	.	.	.	.	.	.	.	.	0.85 (0.50 - 1.47)
1.03 (0.39 - 2.70)	1.59 (0.62 - 4.05)	0.54 (0.19 - 1.55)	0.77 (0.29 - 2.10)	0.73 (0.21 - 2.57)	1.14 (0.47 - 2.76)	Flunitrazepam	.	.	.	.	.	.	.	.	.	.	.	.	.	.	.	.	0.41 (0.17 - 0.98)	.	.	.	.	3.13 (0.60 - 16.31)
0.94 (0.41 - 2.12)	1.45 (0.66 - 3.15)	0.49 (0.20 - 1.23)	0.71 (0.30 - 1.65)	0.67 (0.26 - 1.68)	1.04 (0.50 - 2.14)	0.91 (0.31 - 2.71)	Flurazepam	.	.	.	.	.	.	.	0.96 (0.45 - 2.07)	.	.	.	.	.	.	.	0.32 (0.03 - 3.29)	.	.	.	1.33 (0.35 - 5.06)	.
0.89 (0.53 - 1.50)	1.38 (0.87 - 2.18)	0.47 (0.24 - 0.91)	0.67 (0.38 - 1.19)	0.64 (0.24 - 1.66)	0.99 (0.68 - 1.42)	0.87 (0.35 - 2.16)	0.95 (0.45 - 2.01)	Gaboxadol	.	.	.	.	.	.	1.14 (0.83 - 1.57)	.	.	.	.	.	.	.	.	.	.	.	0.63 (0.39 - 1.03)	.
0.56 (0.29 - 1.08)	0.87 (0.48 - 1.58)	0.29 (0.14 - 0.64)	0.43 (0.22 - 0.84)	0.40 (0.14 - 1.13)	0.62 (0.37 - 1.06)	0.55 (0.20 - 1.48)	0.60 (0.26 - 1.39)	0.63 (0.36 - 1.10)	Indiplon	.	.	.	.	.	1.65 (1.03 - 2.66)	.	.	.	.	.	.	.	.	.	.	.	.	.
0.74 (0.42 - 1.31)	1.15 (0.69 - 1.90)	0.39 (0.19 - 0.79)	0.56 (0.31 - 1.03)	0.53 (0.20 - 1.42)	0.82 (0.54 - 1.26)	0.72 (0.28 - 1.84)	0.79 (0.37 - 1.72)	0.83 (0.53 - 1.31)	1.32 (0.73 - 2.39)	Lemborexant	.	.	.	.	1.16 (0.79 - 1.71)	.	.	.	.	.	.	.	.	.	.	.	1.17 (0.61 - 2.22)	.
2.10 (0.17 - 26.38)	3.24 (0.26 - 40.18)	1.10 (0.08 - 14.25)	1.58 (0.12 - 20.06)	1.49 (0.10 - 21.28)	2.32 (0.19 - 28.35)	2.04 (0.15 - 28.60)	2.24 (0.17 - 29.75)	2.35 (0.19 - 28.89)	3.72 (0.29 - 47.09)	2.82 (0.23 - 34.97)	Lometazepam	.	.	.	0.44 (0.04 - 5.38)	.	.	.	.	.	.	.	.	0.19 (0.02 - 1.94)	.	.	.	.
0.99 (0.49 - 1.98)	1.53 (0.80 - 2.92)	0.52 (0.23 - 1.17)	0.75 (0.36 - 1.55)	0.70 (0.25 - 2.02)	1.09 (0.61 - 1.96)	0.96 (0.43 - 2.17)	1.06 (0.45 - 2.47)	1.11 (0.60 - 2.04)	1.75 (0.85 - 3.61)	1.33 (0.70 - 2.53)	0.47 (0.04 - 6.05)	Loprazolam	.	.	.	.	.	.	.	.	.	.	0.60 (0.47 - 0.75)	.	.	.	.	.
1.65 (0.63 - 4.32)	2.55 (1.01 - 6.43)	0.86 (0.30 - 2.46)	1.24 (0.47 - 3.33)	1.18 (0.34 - 4.11)	1.82 (0.75 - 4.42)	1.61 (0.48 - 5.43)	1.76 (0.59 - 5.28)	1.85 (0.75 - 4.55)	2.93 (1.10 - 7.78)	2.22 (0.88 - 5.59)	0.79 (0.06 - 10.97)	1.67 (0.61 - 4.59)	Melatonin	.	0.56 (0.24 - 1.33)	.	.	.	.	.	.	.	.	.	.	.	.	.
1.38 (0.32 - 6.02)	2.13 (0.50 - 9.12)	0.72 (0.16 - 3.35)	1.04 (0.24 - 4.63)	0.99 (0.18 - 5.27)	1.53 (0.37 - 6.36)	1.35 (0.27 - 6.72)	1.48 (0.31 - 7.03)	1.55 (0.37 - 6.50)	2.45 (0.56 - 10.85)	1.86 (0.44 - 7.92)	0.66 (0.04 - 11.56)	1.40 (0.33 - 5.94)	0.84 (0.16 - 4.35)	Midazolam	.	.	.	.	.	0.59 (0.17 - 2.10)	.	.	.	.	.	.	.	.
0.93 (0.59 - 1.46)	1.44 (1.00 - 2.06)	0.49 (0.27 - 0.89)	0.70 (0.43 - 1.14)	0.66 (0.27 - 1.66)	1.03 (0.82 - 1.29)	0.91 (0.38 - 2.16)	0.99 (0.50 - 1.98)	1.04 (0.78 - 1.39)	1.65 (1.03 - 2.66)	1.25 (0.88 - 1.79)	0.44 (0.04 - 5.38)	0.94 (0.55 - 1.62)	0.56 (0.24 - 1.33)	0.67 (0.16 - 2.75)	Placebo	1.04 (0.79 - 1.37)	1.40 (0.58 - 3.39)	0.80 (0.56 - 1.14)	0.68 (0.26 - 1.77)	1.12 (0.56 - 2.23)	0.76 (0.22 - 2.66)	0.57 (0.30 - 1.10)	0.75 (0.42 - 1.36)	0.44 (0.07 - 2.76)	1.17 (0.37 - 3.64)	0.88 (0.28 - 2.77)	0.79 (0.65 - 0.97)	.
0.97 (0.57 - 1.64)	1.50 (0.95 - 2.35)	0.51 (0.26 - 0.98)	0.73 (0.42 - 1.28)	0.69 (0.27 - 1.80)	1.07 (0.75 - 1.53)	0.94 (0.38 - 2.34)	1.04 (0.49 - 2.17)	1.09 (0.73 - 1.61)	1.72 (0.99 - 2.98)	1.30 (0.83 - 2.04)	0.46 (0.04 - 5.68)	0.98 (0.53	0.59 (0.24	0.70 (0.17	1.04 (0.79	Ramelteon	.	.	.	.	.	.	.	.	.	.	.	.

												- 1.80)	- 1.44)	- 2.94)	- 1.37)													
1.16 (0.50 - 2.68)	1.79 (0.80 - 4.01)	0.61 (0.24 - 1.56)	0.87 (0.37 - 2.09)	0.83 (0.26 - 2.65)	1.28 (0.60 - 2.73)	1.13 (0.37 - 3.47)	1.24 (0.46 - 3.35)	1.30 (0.60 - 2.81)	2.06 (0.87 - 4.89)	1.56 (0.70 - 3.47)	0.55 (0.04 - 7.42)	1.17 (0.48 - 2.88)	0.70 (0.23 - 2.15)	0.84 (0.17 - 4.06)	1.24 (0.60 - 2.56)	1.20 (0.55 - 2.59)	Seltorexant	.	.	.	.	.	.	.	.	.	0.69 (0.29 - 1.68)	.
0.74 (0.42 - 1.32)	1.15 (0.69 - 1.91)	0.39 (0.19 - 0.79)	0.56 (0.31 - 1.03)	0.53 (0.20 - 1.42)	0.82 (0.54 - 1.26)	0.72 (0.28 - 1.85)	0.79 (0.37 - 1.73)	0.83 (0.53 - 1.32)	1.32 (0.73 - 2.40)	1.00 (0.60 - 1.66)	0.36 (0.03 - 4.41)	0.75 (0.39 - 1.45)	0.45 (0.18 - 1.14)	0.54 (0.13 - 2.30)	0.80 (0.56 - 1.14)	0.77 (0.49 - 1.20)	0.64 (0.29 - 1.44)	Suvorexant	.	.	.	.	.	.	.	.	.	.
0.63 (0.22 - 1.82)	0.98 (0.35 - 2.72)	0.33 (0.11 - 1.03)	0.48 (0.16 - 1.40)	0.45 (0.12 - 1.70)	0.70 (0.26 - 1.87)	0.62 (0.17 - 2.24)	0.68 (0.21 - 2.20)	0.71 (0.26 - 1.93)	1.12 (0.39 - 3.27)	0.85 (0.31 - 2.36)	0.30 (0.02 - 4.36)	0.64 (0.21 - 1.92)	0.38 (0.11 - 1.38)	0.46 (0.08 - 2.51)	0.68 (0.26 - 1.77)	0.65 (0.24 - 1.76)	0.55 (0.16 - 1.81)	0.85 (0.31 - 2.36)	Tasimelteon	.	.	.	.	.	.	.	.	.
0.82 (0.39 - 1.73)	1.27 (0.62 - 2.58)	0.43 (0.18 - 1.02)	0.62 (0.28 - 1.36)	0.58 (0.19 - 1.75)	0.91 (0.47 - 1.74)	0.80 (0.30 - 2.15)	0.88 (0.35 - 2.18)	0.92 (0.47 - 1.80)	1.45 (0.67 - 3.17)	1.10 (0.54 - 2.23)	0.39 (0.03 - 5.10)	0.83 (0.41 - 1.67)	0.50 (0.17 - 1.42)	0.59 (0.17 - 2.10)	0.88 (0.48 - 1.63)	0.85 (0.43 - 1.66)	0.71 (0.28 - 1.81)	1.10 (0.54 - 2.25)	1.29 (0.42 - 4.03)	Temazepam	.	.	0.78 (0.38 - 1.59)	.	.	.	1.22 (0.57 - 2.62)	.
0.71 (0.19 - 2.68)	1.09 (0.29 - 4.03)	0.37 (0.09 - 1.49)	0.53 (0.14 - 2.05)	0.50 (0.11 - 2.38)	0.78 (0.22 - 2.80)	0.69 (0.15 - 3.17)	0.75 (0.18 - 3.16)	0.79 (0.22 - 2.87)	1.25 (0.33 - 4.81)	0.95 (0.26 - 3.51)	0.34 (0.02 - 5.49)	0.71 (0.18 - 2.81)	0.43 (0.09 - 1.96)	0.51 (0.08 - 3.37)	0.76 (0.22 - 2.66)	0.73 (0.20 - 2.64)	0.61 (0.14 - 2.60)	0.95 (0.26 - 3.51)	1.11 (0.23 - 5.40)	0.86 (0.21 - 3.49)	Tiagabine	.	.	.	.	.	.	.
0.54 (0.27 - 1.08)	0.83 (0.43 - 1.60)	0.28 (0.12 - 0.64)	0.40 (0.19 - 0.85)	0.38 (0.13 - 1.11)	0.59 (0.33 - 1.08)	0.52 (0.19 - 1.45)	0.57 (0.24 - 1.38)	0.60 (0.32 - 1.11)	0.95 (0.46 - 1.98)	0.72 (0.37 - 1.39)	0.26 (0.02 - 3.29)	0.54 (0.25 - 1.17)	0.33 (0.12 - 0.90)	0.39 (0.09 - 1.75)	0.58 (0.33 - 1.00)	0.55 (0.30 - 1.03)	0.46 (0.19 - 1.14)	0.72 (0.37 - 1.40)	0.85 (0.28 - 2.56)	0.65 (0.29 - 1.48)	0.76 (0.19 - 3.01)	Trazodone	.	.	.	.	1.33 (0.71 - 2.50)	.
0.59 (0.31 - 1.13)	0.91 (0.49 - 1.67)	0.31 (0.14 - 0.67)	0.44 (0.22 - 0.89)	0.42 (0.15 - 1.17)	0.65 (0.38 - 1.11)	0.57 (0.26 - 1.25)	0.63 (0.28 - 1.43)	0.66 (0.38 - 1.16)	1.04 (0.53 - 2.07)	0.79 (0.43 - 1.44)	0.28 (0.02 - 3.56)	0.60 (0.47 - 0.75)	0.36 (0.13 - 0.96)	0.43 (0.10 - 1.77)	0.63 (0.39 - 1.03)	0.61 (0.35 - 1.07)	0.51 (0.21 - 1.21)	0.79 (0.43 - 1.45)	0.93 (0.32 - 2.72)	0.72 (0.37 - 1.39)	0.83 (0.22 - 3.22)	1.10 (0.53 - 2.28)	Triazolam	.	.	1.48 (0.51 - 4.29)	1.14 (0.60 - 2.18)	.
0.41 (0.06 - 2.71)	0.63 (0.10 - 4.11)	0.21 (0.03 - 1.48)	0.31 (0.05 - 2.07)	0.29 (0.04 - 2.27)	0.45 (0.07 - 2.89)	0.40 (0.05 - 3.04)	0.44 (0.06 - 3.11)	0.46 (0.07 - 2.95)	0.72 (0.11 - 4.85)	0.55 (0.08 - 3.58)	0.19 (0.02 - 1.94)	0.41 (0.06 - 2.82)	0.25 (0.03 - 1.88)	0.29 (0.03 - 3.00)	0.44 (0.07 - 2.76)	0.42 (0.07 - 2.71)	0.35 (0.05 - 2.55)	0.55 (0.08 - 3.58)	0.64 (0.08 - 5.12)	0.50 (0.07 - 3.47)	0.58 (0.06 - 5.38)	0.76 (0.11 - 5.21)	0.69 (0.10 - 4.67)	Trimipramine	.	.	.	.
1.09 (0.32 - 3.69)	1.68 (0.51 - 5.53)	0.57 (0.16 - 2.06)	0.82 (0.24 - 2.83)	0.77 (0.18 - 3.34)	1.20 (0.38 - 3.83)	1.06 (0.25 - 4.43)	1.16 (0.31 - 4.39)	1.22 (0.38 - 3.94)	1.93 (0.56 - 6.62)	1.46 (0.44 - 4.81)	0.52 (0.03 - 8.03)	1.10 (0.31 - 3.88)	0.66 (0.16 - 2.73)	0.79 (0.13 - 4.80)	1.17 (0.37 - 3.64)	1.12 (0.35 - 3.61)	0.94 (0.24 - 3.61)	1.46 (0.44 - 4.81)	1.72 (0.39 - 7.57)	1.33 (0.36 - 4.83)	1.54 (0.28 - 8.40)	2.03 (0.57 - 7.19)	1.85 (0.54 - 6.38)	2.67 (0.31 - 23.25)	Vestipitant	.	.	.
0.89 (0.35 - 2.30)	1.38 (0.55 - 3.45)	0.47 (0.17 - 1.32)	0.68 (0.26 - 1.79)	0.64 (0.18 - 2.20)	0.99 (0.41 - 2.37)	0.87 (0.28 - 2.70)	0.96 (0.33 - 2.81)	1.00 (0.41 - 2.43)	1.59 (0.60 - 4.18)	1.20 (0.48 - 2.99)	0.43 (0.03 - 5.93)	0.91 (0.37 - 2.19)	0.54 (0.16 - 1.80)	0.65 (0.13 - 3.25)	0.96 (0.41 - 2.23)	0.92 (0.38 - 2.24)	0.77 (0.26 - 2.33)	1.20 (0.48 - 3.00)	1.41 (0.40 - 5.05)	1.09 (0.40 - 2.97)	1.27 (0.28 - 5.77)	1.67 (0.61 - 4.55)	1.52 (0.65 - 3.57)	2.20 (0.29 - 16.66)	0.82 (0.20 - 3.39)	Zaleplon	0.64 (0.10 - 4.02)	.
0.72 (0.46 - 1.11)	1.11 (0.74 - 1.64)	0.37 (0.20 - 0.71)	0.54 (0.32 - 0.91)	0.51 (0.20 - 1.29)	0.79 (0.59 - 1.06)	0.70 (0.29 - 1.67)	0.76 (0.38 - 1.55)	0.80 (0.59 - 1.10)	1.27 (0.76 - 2.12)	0.96 (0.66 - 1.41)	0.34 (0.03 - 4.16)	0.72 (0.42 - 1.25)	0.43 (0.18 - 1.04)	0.52 (0.13 - 2.12)	0.77 (0.64 - 0.93)	0.74 (0.53 - 1.03)	0.62 (0.30 - 1.27)	0.96 (0.64 - 1.44)	1.13 (0.43 - 2.99)	0.87 (0.47 - 1.62)	1.01 (0.28 - 3.62)	1.34 (0.77 - 2.33)	1.22 (0.74 - 2.00)	1.76 (0.28 - 11.20)	0.66 (0.21 - 2.09)	0.80 (0.34 - 1.87)	Zolpidem	1.84 (0.79 - 4.29)
0.99 (0.52 - 1.88)	1.52 (0.84 - 2.76)	0.52 (0.24 - 1.11)	0.74 (0.38 - 1.47)	0.70 (0.25 - 1.97)	1.09 (0.69 - 1.72)	0.96 (0.38 - 2.42)	1.05 (0.46 - 2.42)	1.11 (0.64 - 1.91)	1.75 (0.89 - 3.43)	1.33 (0.74 - 2.39)	0.47 (0.04 - 5.96)	1.00 (0.50 - 2.00)	0.60 (0.23 - 1.59)	0.71 (0.16 - 3.13)	1.06 (0.66 - 1.70)	1.02 (0.59 - 1.76)	0.85 (0.36 - 2.01)	1.33 (0.73 - 2.40)	1.56 (0.54 - 4.52)	1.20 (0.56 - 2.58)	1.40 (0.36 - 5.37)	1.84 (0.89 - 3.79)	1.68 (0.87 - 3.23)	2.42 (0.36 - 16.23)	0.91 (0.26 - 3.11)	1.10 (0.42 - 2.86)	1.38 (0.85 - 2.24)	Zopiclone

Leaguetable for Amnesia

Flurazepam	.	1.04 (0.06 - 17.43)	.	0.47 (0.05 - 4.38)
1.00 (0.07 - 13.97)	Placebo	.	0.78 (0.22 - 2.86)	0.47 (0.12 - 1.93)
1.04 (0.06 - 17.43)	1.04 (0.02 - 49.35)	Triazolam	.	.
0.78 (0.07 - 9.10)	0.78 (0.22 - 2.86)	0.76 (0.02 - 31.76)	Zaleplon	0.60 (0.22 - 1.66)
0.47 (0.05 - 4.38)	0.47 (0.12 - 1.93)	0.45 (0.01 - 16.57)	0.60 (0.22 - 1.66)	Zolpidem

Leaguetable for Difficulty concentrating

Flurazepam	0.47 (0.04 - 5.45)	.	.
0.47 (0.04 - 5.45)	Placebo	2.99 (0.73 - 12.25)	0.48 (0.23 - 1.01)
1.01 (0.06 - 15.95)	2.14 (0.60 - 7.67)	Seltorexant	0.33 (0.08 - 1.34)
0.24 (0.02 - 3.05)	0.50 (0.24 - 1.06)	0.23 (0.07 - 0.84)	Zolpidem

Leaguetable for Nightmare

Lemborexant	8.63 (1.11 - 66.89)	.
8.63 (1.11 - 66.89)	Placebo	4.57 (0.46 - 45.86)
39.47 (1.81 - 861.80)	4.57 (0.46 - 45.86)	Temazepam

Leaguetable for dysgeusia

Eszopiclone	.	10.21 (6.52 - 15.98)	.	.	.	.	.
1.02 (0.24 - 4.39)	Flurazepam	11.18 (2.62 - 47.63)	.	.	.	.	1.00 (0.12 - 8.56)
10.21 (6.52 - 15.98)	10.03 (2.50 - 40.32)	Placebo	0.65 (0.30 - 1.40)	.	0.77 (0.25 - 2.35)	3.97 (0.44 - 36.01)	0.10 (0.01 - 0.69)
6.59 (2.70 - 16.13)	6.48 (1.32 - 31.84)	0.65 (0.30 - 1.40)	Ramelteon	.	.	.	.
22.71 (0.92 - 558.72)	22.32 (0.79 - 631.06)	2.22 (0.09 - 53.04)	3.44 (0.13 - 90.10)	Triazolam	.	1.04 (0.06 - 17.04)	.
7.00 (2.19 - 22.40)	6.88 (1.25 - 37.98)	0.69 (0.23 - 2.01)	1.06 (0.28 - 3.99)	0.31 (0.01 - 7.62)	Zaleplon	5.19 (0.68 - 39.67)	.
23.73 (4.94 - 113.97)	23.32 (3.72 - 146.34)	2.32 (0.52 - 10.46)	3.60 (0.66 - 19.53)	1.04 (0.06 - 17.04)	3.39 (0.70 - 16.42)	Zolpidem	0.07 (0.03 - 0.16)
1.45 (0.31 - 6.71)	1.42 (0.24 - 8.36)	0.14 (0.03 - 0.61)	0.22 (0.04 - 1.15)	0.06 (0.00 - 1.17)	0.21 (0.04 - 1.02)	0.06 (0.03 - 0.14)	Zopiclone

Leaguetable for Asthenia

Doxepin	.	.	.	.	1.50 (0.23 - 9.92)	.	.	.
2.89 (0.32 - 26.24)	Estazolam	.	0.35 (0.12 - 1.04)	.	0.52 (0.17 - 1.62)	.	.	.
1.96 (0.26 - 14.78)	0.68 (0.18 - 2.61)	Eszopiclone	.	.	0.77 (0.37 - 1.58)	.	.	.
1.02 (0.13 - 8.34)	0.35 (0.12 - 1.04)	0.52 (0.16 - 1.68)	Flurazepam	.	1.47 (0.59 - 3.65)	.	.	.
3.38 (0.15 - 77.03)	1.17 (0.08 - 18.11)	1.73 (0.13 - 23.15)	3.30 (0.23 - 46.87)	Lometazepam	0.44 (0.04 - 5.38)	0.44 (0.04 - 5.38)	.	.
1.50 (0.23 - 9.92)	0.52 (0.17 - 1.62)	0.77 (0.37 - 1.58)	1.47 (0.59 - 3.65)	0.44 (0.04 - 5.38)	Placebo	1.00 (0.13 - 8.00)	0.51 (0.17 - 1.51)	0.63 (0.17 - 2.30)
1.50 (0.09 - 24.88)	0.52 (0.05 - 5.56)	0.77 (0.08 - 6.93)	1.47 (0.15 - 14.18)	0.44 (0.04 - 5.38)	1.00 (0.13 - 8.00)	Trimipramine	.	.
0.77 (0.09 - 6.77)	0.27 (0.05 - 1.28)	0.39 (0.11 - 1.45)	0.75 (0.18 - 3.09)	0.23 (0.01 - 3.45)	0.51 (0.17 - 1.51)	0.51 (0.05 - 5.33)	Zaleplon	1.24 (0.49 - 3.13)
0.95 (0.10 - 9.37)	0.33 (0.06 - 1.84)	0.49 (0.11 - 2.14)	0.93 (0.19 - 4.51)	0.28 (0.02 - 4.67)	0.63 (0.17 - 2.30)	0.63 (0.05 - 7.32)	1.24 (0.49 - 3.13)	Zolpidem

Leaguetable for nervousness

Doxepin	.	.	.	.	0.68 (0.13 - 3.43)	.	.	.	.
1.73 (0.21 - 14.51)	Estazolam	.	0.76 (0.16 - 3.51)	.	0.40 (0.10 - 1.61)	.	.	.	.
0.40 (0.05 - 2.93)	0.23 (0.04 - 1.40)	Eszopiclone	.	.	1.71 (0.53 - 5.51)	.	.	.	.
1.37 (0.19 - 10.01)	0.79 (0.18 - 3.52)	3.45 (0.67 - 17.85)	Flurazepam	.	0.53 (0.15 - 1.88)	.	.	.	0.64 (0.06 - 6.36)
1.42 (0.07 - 29.06)	0.82 (0.05 - 14.55)	3.58 (0.22 - 59.00)	1.04 (0.07 - 16.16)	Nitrazepam	.	.	0.31 (0.03 - 3.17)	.	.
0.68 (0.13 - 3.43)	0.39 (0.10 - 1.55)	1.71 (0.53 - 5.51)	0.50 (0.16 - 1.57)	0.48 (0.04 - 6.08)	Placebo	1.35 (0.29 - 6.23)	0.64 (0.22 - 1.85)	2.03 (0.27 - 15.09)	2.00 (0.36 - 11.23)
0.96 (0.11 - 8.38)	0.55 (0.08 - 3.95)	2.41 (0.38 - 15.47)	0.70 (0.12 - 4.11)	0.67 (0.05 - 9.92)	1.41 (0.33 - 5.97)	Temazepam	0.41 (0.10 - 1.65)	.	1.48 (0.24 - 9.10)
0.44 (0.06 - 3.01)	0.25 (0.05 - 1.38)	1.11 (0.23 - 5.29)	0.32 (0.07 - 1.38)	0.31 (0.03 - 3.17)	0.65 (0.23 - 1.82)	0.46 (0.12 - 1.79)	Triazolam	2.24 (0.30 - 16.70)	2.76 (0.70 - 10.88)
1.17 (0.10 - 13.23)	0.67 (0.07 - 6.46)	2.94 (0.34 - 25.34)	0.85 (0.10 - 7.05)	0.82 (0.04 - 15.61)	1.72 (0.28 - 10.49)	1.22 (0.14 - 10.66)	2.65 (0.43 - 16.18)	Zaleplon	.
1.07 (0.13 - 8.78)	0.62 (0.10 - 3.89)	2.71 (0.46 - 16.01)	0.78 (0.17 - 3.56)	0.75 (0.05 - 10.79)	1.58 (0.42 - 6.03)	1.12 (0.22 - 5.77)	2.44 (0.67 - 8.88)	0.92 (0.11 - 7.62)	Zolpidem

Leaguetable for Dyspepsia

Doxepin	.	0.29 (0.03 - 3.01)	.	.	.	.	.
0.26 (0.02 - 2.80)	Eszopiclone	1.12 (0.73 - 1.73)	.	.	.	.	0.68 (0.39 - 1.18)
0.29 (0.03 - 3.01)	1.12 (0.73 - 1.73)	Placebo	1.44 (0.44 - 4.72)	2.12 (0.61 - 7.35)	.	0.98 (0.35 - 2.75)	.
0.37 (0.03 - 5.01)	1.41 (0.41 - 4.91)	1.26 (0.39 - 4.06)	Temazepam	1.73 (0.40 - 7.48)	.	0.97 (0.27 - 3.50)	.
0.66 (0.05 - 9.35)	2.54 (0.68 - 9.54)	2.27 (0.65 - 7.92)	1.80 (0.45 - 7.16)	Triazolam	1.08 (0.09 - 12.42)	0.45 (0.13 - 1.58)	.
0.71 (0.02 - 26.20)	2.75 (0.17 - 44.15)	2.46 (0.16 - 38.10)	1.95 (0.12 - 32.12)	1.08 (0.09 - 12.42)	Zaleplon	.	.
0.29 (0.02 - 3.84)	1.13 (0.36 - 3.59)	1.01 (0.35 - 2.95)	0.80 (0.23 - 2.73)	0.44 (0.13 - 1.58)	0.41 (0.03 - 6.41)	Zolpidem	.
0.18 (0.02 - 2.03)	0.68 (0.39 - 1.18)	0.61 (0.30 - 1.23)	0.48 (0.12 - 1.88)	0.27 (0.06 - 1.13)	0.25 (0.01 - 4.19)	0.60 (0.17 - 2.17)	Zopiclone

Leaguetable for Diarrhea

Almorexant	.	.	.	.	3.47 (0.42 - 28.43)	.	.	.	.	1.64 (0.34 - 7.96)
3.58 (0.45 - 28.36)	Daridorexant	.	.	.	0.49 (0.09 - 2.76)	.	.	.	.	1.00 (0.11 - 9.15)
6.90 (0.96 - 49.35)	1.93 (0.27 - 13.84)	Doxepin	.	.	0.35 (0.10 - 1.15)	.	.	.	.	.
2.26 (0.42 - 12.09)	0.63 (0.12 - 3.39)	0.33 (0.08 - 1.27)	Eszopiclone	.	1.06 (0.57 - 1.98)	.	.	.	.	.
2.45 (0.10 - 60.68)	0.69 (0.03 - 16.99)	0.36 (0.02 - 7.51)	1.09 (0.06 - 19.22)	Melatonin	0.98 (0.06 - 16.13)	.	.	.	.	.
2.40 (0.50 - 11.39)	0.67 (0.14 - 3.20)	0.35 (0.10 - 1.15)	1.06 (0.57 - 1.98)	0.98 (0.06 - 16.13)	Placebo	0.71 (0.40 - 1.28)	8.96 (0.92 - 87.48)	0.86 (0.41 - 1.79)	0.93 (0.19 - 4.50)	0.83 (0.27 - 2.51)
1.71 (0.32 - 9.02)	0.48 (0.09 - 2.53)	0.25 (0.07 - 0.94)	0.76 (0.32 - 1.78)	0.70 (0.04 - 12.21)	0.71 (0.40 - 1.28)	Ramelteon	.	.	.	.
23.18 (1.88 - 285.29)	6.48 (0.48 - 87.89)	3.36 (0.27 - 41.34)	10.27 (1.04 - 101.42)	9.45 (0.27 - 334.43)	9.67 (1.07 - 87.63)	13.58 (1.39 - 132.80)	Seltorexant	.	.	0.08 (0.01 - 0.73)
2.06 (0.37 - 11.54)	0.58 (0.10 - 3.24)	0.30 (0.07 - 1.22)	0.91 (0.35 - 2.39)	0.84 (0.05 - 15.24)	0.86 (0.41 - 1.79)	1.21 (0.47 - 3.09)	0.09 (0.01 - 0.91)	Suvorexant	.	.
2.22 (0.24 - 20.45)	0.62 (0.07 - 5.73)	0.32 (0.04 - 2.34)	0.99 (0.18 - 5.38)	0.91 (0.04 - 22.65)	0.93 (0.19 - 4.50)	1.30 (0.24 - 7.02)	0.10 (0.01 - 1.44)	1.08 (0.19 - 6.16)	Temazepam	.
1.97 (0.48 - 8.11)	0.55 (0.10 - 3.00)	0.29 (0.05 - 1.50)	0.87 (0.24 - 3.21)	0.80 (0.04 - 16.60)	0.82 (0.26 - 2.58)	1.16 (0.32 - 4.17)	0.09 (0.01 - 0.74)	0.96 (0.25 - 3.72)	0.89 (0.13 - 6.22)	Zolpidem

Leaguetable for Dry mouth

Doxepin	.	.	.	.	.	1.59 (0.56 - 4.50)	.	.	.	.
1.18 (0.32 - 4.39)	Esmirtazapine	.	.	.	.	1.35 (0.60 - 3.01)	.	.	.	.
0.37 (0.10 - 1.37)	0.31 (0.10 - 0.97)	Eszopiclone	.	.	.	4.31 (1.94 - 9.58)	.	.	.	.
1.24 (0.22 - 7.09)	1.05 (0.21 - 5.28)	3.36 (0.67 - 16.83)	Flurazepam	.	.	0.70 (0.13 - 3.68)	.	.	4.06 (0.36 - 46.35)	0.13 (0.01 - 1.33)
0.41 (0.04 - 4.09)	0.35 (0.04 - 3.15)	1.12 (0.12 - 10.04)	0.33 (0.03 - 3.43)	Midazolam	.	.	.	.	.	1.86 (0.30 - 11.75)
1.68 (0.09 - 29.97)	1.43 (0.09 - 23.55)	4.56 (0.28 - 75.14)	1.36 (0.07 - 24.96)	4.07 (0.18 - 93.41)	Nitrazepam	.	.	.	.	0.46 (0.04 - 5.79)
1.59 (0.56 - 4.50)	1.35 (0.60 - 3.01)	4.31 (1.94 - 9.58)	1.28 (0.32 - 5.20)	3.85 (0.50 - 29.73)	0.95 (0.06 - 13.90)	Placebo	0.46 (0.25 - 0.84)	0.49 (0.04 - 5.49)	.	0.38 (0.16 - 0.95)
0.73 (0.22 - 2.43)	0.62 (0.23 - 1.69)	1.99 (0.74 - 5.39)	0.59 (0.13 - 2.71)	1.78 (0.21 - 14.95)	0.44 (0.03 - 6.85)	0.46 (0.25 - 0.84)	Suvorexant	.	.	.
0.78 (0.06 - 10.81)	0.66 (0.05 - 8.43)	2.10 (0.16 - 26.89)	0.63 (0.04 - 10.24)	1.88 (0.08 - 44.61)	0.46 (0.01 - 17.17)	0.49 (0.04 - 5.49)	1.06 (0.09 - 12.77)	Vestipitant	.	.
0.60 (0.11 - 3.35)	0.51 (0.10 - 2.49)	1.63 (0.33 - 7.94)	0.48 (0.10 - 2.35)	1.45 (0.17 - 12.57)	0.36 (0.02 - 5.73)	0.38 (0.10 - 1.48)	0.82 (0.18 - 3.64)	0.77 (0.05 - 12.50)	Zolpidem	2.18 (0.65 - 7.34)
0.77 (0.20 - 3.02)	0.65 (0.20 - 2.16)	2.09 (0.63 - 6.89)	0.62 (0.15 - 2.61)	1.86 (0.30 - 11.75)	0.46 (0.04 - 5.79)	0.48 (0.20 - 1.18)	1.05 (0.36 - 3.05)	0.99 (0.08 - 13.08)	1.28 (0.42 - 3.95)	Zopiclone

Leaguetable for Nausea/vomiting

Almorexant	.	.	.	.	.	.	.	.	.	.	.	.	.	0.69 (0.26 - 1.84)	.	.	.	.	.	.	1.16 (0.36 - 3.77)	.
0.70 (0.16 - 3.13)	Alprazolam	.	.	.	.	.	.	.	.	.	.	.	3.27 (0.77 - 13.83)	1.45 (0.44 - 4.86)	.	.	.	.	.	.	.	.
1.29 (0.39 - 4.20)	1.83 (0.43 - 7.84)	Daridorexant	.	.	.	.	.	.	.	.	.	.	.	1.08 (0.41 - 2.86)	.	.	.	.	.	.	0.24 (0.06 - 0.98)	.
0.68 (0.16 - 2.92)	0.97 (0.18 - 5.16)	0.53 (0.13 - 2.17)	Doxepin	.	.	.	.	.	.	.	.	.	.	1.50 (0.47 - 4.74)	.	.	.	.	.	.	.	.
1.10 (0.28 - 4.32)	1.57 (0.32 - 7.73)	0.85 (0.23 - 3.21)	1.61 (0.34 - 7.64)	Esmirtazapine	.	.	.	.	.	.	.	.	.	0.93 (0.33 - 2.64)	.	.	.	.	.	.	.	.
1.31 (0.26 - 6.59)	1.87 (0.30 - 11.43)	1.02 (0.21 - 4.93)	1.92 (0.32 - 11.35)	1.19 (0.22 - 6.59)	Estazolam	.	.	4.26 (0.47 - 38.96)	.	.	.	.	.	0.78 (0.20 - 3.01)	.	.	.	.	.	.	.	.
0.52 (0.18 - 1.49)	0.74 (0.19 - 2.84)	0.40 (0.15 - 1.09)	0.76 (0.21 - 2.78)	0.47 (0.14 - 1.57)	0.40 (0.09 - 1.73)	Eszopiclone	.	.	.	.	.	.	.	2.13 (1.10 - 4.12)	.	.	.	.	.	.	.	1.19 (0.44 - 3.21)
1.08 (0.15 - 7.85)	1.53 (0.18 - 13.35)	0.84 (0.12 - 5.93)	1.57 (0.19 - 13.33)	0.98 (0.12 - 7.83)	0.82 (0.09 - 7.78)	2.07 (0.31 - 13.66)	Flunitrazepam	.	.	.	.	.	.	.	.	.	.	.	0.75 (0.16 - 3.39)	.	.	.
5.59 (0.54 - 58.10)	7.95 (0.66 - 95.19)	4.34 (0.43 - 44.01)	8.18 (0.70 - 95.41)	5.08 (0.46 - 56.44)	4.26 (0.47 - 38.96)	10.76 (1.13 - 102.02)	5.19 (0.31 - 86.94)	Flurazepam	.	.	.	.	.	0.18 (0.02 - 1.60)	.	.	.	.	.	.	.	.
0.31 (0.12 - 0.83)	0.44 (0.12 - 1.63)	0.24 (0.10 - 0.61)	0.45 (0.13 - 1.59)	0.28 (0.09 - 0.90)	0.24 (0.06 - 1.00)	0.60 (0.28 - 1.27)	0.29 (0.05 - 1.83)	0.06 (0.01 - 0.51)	Gaboxadol	.	.	.	.	4.49 (2.49 - 8.08)	.	.	.	.	.	.	1.26 (0.61 - 2.59)	.

0.57 (0.14 - 2.39)	0.81 (0.16 - 4.27)	0.44 (0.11 - 1.78)	0.84 (0.17 - 4.23)	0.52 (0.11 - 2.44)	0.44 (0.07 - 2.55)	1.10 (0.31 - 3.96)	0.53 (0.06 - 4.43)	0.10 (0.01 - 1.19)	1.84 (0.54 - 6.29)	Lemborexant	.	.	.	2.05 (0.57 - 7.32)	.	.	.	.	.	.	0.61 (0.05 - 7.40)	.
0.11 (0.01 - 1.29)	0.16 (0.01 - 2.13)	0.09 (0.01 - 0.98)	0.16 (0.01 - 2.14)	0.10 (0.01 - 1.27)	0.08 (0.01 - 1.23)	0.21 (0.02 - 2.15)	0.10 (0.01 - 1.89)	0.02 (0.00 - 0.47)	0.36 (0.03 - 3.72)	0.19 (0.01 - 2.52)	Midazolam	.	.	.	.	.	.	.	.	.	.	6.71 (0.75 - 60.06)
1.62 (0.10 - 25.88)	2.30 (0.13 - 41.97)	1.26 (0.08 - 19.71)	2.37 (0.13 - 42.23)	1.47 (0.09 - 25.16)	1.23 (0.06 - 23.97)	3.11 (0.22 - 43.83)	1.50 (0.06 - 36.17)	0.29 (0.01 - 8.83)	5.20 (0.36 - 75.43)	2.83 (0.16 - 49.82)	14.65 (0.51 - 418.19)	Nitrazepam	.	.	.	.	.	.	.	.	.	0.46 (0.04 - 5.79)
2.30 (0.41 - 12.99)	3.27 (0.77 - 13.83)	1.79 (0.33 - 9.75)	3.36 (0.51 - 22.16)	2.09 (0.34 - 12.91)	1.75 (0.23 - 13.13)	4.43 (0.89 - 22.04)	2.14 (0.21 - 22.10)	0.41 (0.03 - 5.73)	7.40 (1.54 - 35.55)	4.02 (0.62 - 26.21)	20.83 (1.33 - 326.27)	1.42 (0.07 - 29.52)	Paroxetine	0.44 (0.10 - 1.97)	.	.	.	.	.	.	.	.
1.02 (0.42 - 2.47)	1.45 (0.44 - 4.86)	0.79 (0.35 - 1.79)	1.50 (0.47 - 4.74)	0.93 (0.33 - 2.64)	0.78 (0.20 - 3.01)	**1.97 (1.09 - 3.56)**	0.95 (0.16 - 5.74)	0.18 (0.02 - 1.60)	3.29 (2.02 - 5.37)	1.79 (0.57 - 5.57)	9.26 (0.92 - 93.48)	0.63 (0.05 - 8.87)	0.44 (0.10 - 1.97)	Placebo	0.83 (0.39 - 1.76)	1.95 (0.32 - 11.87)	0.90 (0.44 - 1.82)	1.85 (0.60 - 5.73)	1.01 (0.31 - 3.28)	.	0.68 (0.44 - 1.04)	0.64 (0.05 - 7.96)
0.85 (0.27 - 2.71)	1.21 (0.29 - 5.02)	0.66 (0.22 - 2.00)	1.25 (0.32 - 4.93)	0.77 (0.21 - 2.80)	0.65 (0.14 - 3.05)	1.64 (0.63 - 4.27)	0.79 (0.11 - 5.56)	0.15 (0.02 - 1.51)	2.74 (1.12 - 6.71)	1.49 (0.38 - 5.81)	7.72 (0.68 - 87.75)	0.53 (0.03 - 8.21)	0.37 (0.07 - 1.97)	0.83 (0.39 - 1.76)	Ramelteon	.	.	.	.	.	.	.
2.71 (0.53 - 13.84)	3.86 (0.61 - 24.48)	2.11 (0.43 - 10.40)	3.97 (0.65 - 24.32)	2.47 (0.43 - 14.13)	2.07 (0.30 - 14.48)	5.23 (1.16 - 23.58)	2.52 (0.27 - 24.01)	0.49 (0.04 - 6.42)	8.74 (2.04 - 37.37)	4.75 (0.79 - 28.42)	24.60 (1.69 - 358.59)	1.68 (0.09 - 32.67)	1.18 (0.15 - 9.11)	2.66 (0.66 - 10.75)	3.18 (0.65 - 15.55)	Seltorexant	.	.	.	.	0.19 (0.04 - 0.82)	.
0.92 (0.30 - 2.84)	1.30 (0.32 - 5.28)	0.71 (0.24 - 2.09)	1.34 (0.35 - 5.19)	0.83 (0.24 - 2.94)	0.70 (0.15 - 3.22)	1.76 (0.70 - 4.45)	0.85 (0.12 - 5.88)	0.16 (0.02 - 1.61)	2.95 (1.25 - 6.98)	1.60 (0.42 - 6.12)	8.30 (0.74 - 93.21)	0.57 (0.04 - 8.73)	0.40 (0.08 - 2.08)	0.90 (0.44 - 1.82)	1.07 (0.38 - 3.01)	0.34 (0.07 - 1.62)	Suvorexant	.	.	.	.	.
1.45 (0.37 - 5.64)	2.07 (0.42 - 10.25)	1.13 (0.30 - 4.20)	2.13 (0.45 - 10.13)	1.32 (0.30 - 5.82)	1.11 (0.20 - 6.15)	2.80 (0.84 - 9.28)	1.35 (0.19 - 9.44)	0.26 (0.02 - 2.90)	4.68 (1.50 - 14.61)	2.54 (0.54 - 11.86)	13.16 (1.05 - 164.60)	0.90 (0.05 - 15.23)	0.63 (0.10 - 3.92)	1.42 (0.50 - 4.07)	1.70 (0.47 - 6.20)	0.54 (0.10 - 3.00)	1.59 (0.45 - 5.64)	Temazepam	0.66 (0.18 - 2.42)	.	0.63 (0.17 - 2.33)	.
0.80 (0.22 - 2.91)	1.14 (0.24 - 5.37)	0.62 (0.18 - 2.16)	1.17 (0.26 - 5.30)	0.73 (0.17 - 3.03)	0.61 (0.12 - 3.23)	1.54 (0.50 - 4.76)	0.75 (0.16 - 3.39)	0.14 (0.01 - 1.55)	2.58 (0.90 - 7.43)	1.40 (0.32 - 6.18)	7.26 (0.60 - 87.55)	0.50 (0.03 - 8.13)	0.35 (0.06 - 2.07)	0.78 (0.30 - 2.07)	0.94 (0.28 - 3.20)	0.30 (0.06 - 1.57)	0.87 (0.26 - 2.91)	0.55 (0.16 - 1.87)	Triazolam	9.78 (1.04 - 91.53)	0.97 (0.33 - 2.88)	.

7.84 (0.59 - 103.56)	11.16 (0.73 - 169.38)	6.09 (0.47 - 78.74)	11.47 (0.77 - 170.16)	7.12 (0.50 - 101.10)	5.98 (0.37 - 97.13)	15.09 (1.23 - 184.57)	7.29 (0.49 - 108.48)	1.40 (0.05 - 36.69)	25.23 (2.13 - 299.55)	13.70 (0.94 - 200.68)	71.02 (2.50 - 2017.22)	4.85 (0.13 - 174.23)	3.41 (0.20 - 59.40)	7.67 (0.67 - 87.83)	9.20 (0.72 - 117.82)	2.89 (0.18 - 47.03)	8.55 (0.68 - 108.37)	5.40 (0.42 - 69.00)	9.78 (1.04 - 91.53)	Zaleplon	.	.
0.59 (0.24 - 1.46)	0.84 (0.24 - 2.99)	0.46 (0.20 - 1.07)	0.87 (0.26 - 2.92)	0.54 (0.18 - 1.64)	0.45 (0.11 - 1.84)	1.14 (0.58 - 2.23)	0.55 (0.09 - 3.31)	0.11 (0.01 - 0.96)	1.91 (1.13 - 3.21)	1.04 (0.32 - 3.35)	5.37 (0.53 - 53.93)	0.37 (0.03 - 5.12)	0.26 (0.06 - 1.20)	0.58 (0.39 - 0.85)	0.69 (0.30 - 1.61)	0.22 (0.06 - 0.86)	0.65 (0.29 - 1.45)	0.41 (0.14 - 1.18)	0.74 (0.28 - 1.94)	0.08 (0.01 - 0.86)	Zolpidem	1.49 (0.59 - 3.78)
0.74 (0.24 - 2.28)	1.05 (0.26 - 4.34)	0.58 (0.20 - 1.68)	1.08 (0.28 - 4.26)	0.67 (0.19 - 2.42)	0.57 (0.12 - 2.64)	1.43 (0.67 - 3.02)	0.69 (0.10 - 4.70)	0.13 (0.01 - 1.31)	2.39 (1.02 - 5.56)	1.30 (0.34 - 4.96)	6.71 (0.75 - 60.06)	0.46 (0.04 - 5.79)	0.32 (0.06 - 1.70)	0.73 (0.35 - 1.52)	0.87 (0.30 - 2.49)	0.27 (0.06 - 1.28)	0.81 (0.29 - 2.25)	0.51 (0.15 - 1.79)	0.92 (0.28 - 3.01)	0.09 (0.01 - 1.19)	1.25 (0.61 - 2.58)	Zopiclone

Leaguetable for Constipation

Doxepin	0.57 (0.14 - 2.35)	.
0.57 (0.14 - 2.35)	Placebo	0.94 (0.12 - 7.52)
0.53 (0.04 - 6.61)	0.94 (0.12 - 7.52)	Temazepam

Leaguetable for Abdominal pain

Daridorexant	.	0.50 (0.04 - 5.58)	.	.	0.12 (0.02 - 0.66)
0.16 (0.02 - 1.11)	Eszopiclone	1.47 (0.75 - 2.90)	.	.	.
0.24 (0.04 - 1.45)	1.47 (0.75 - 2.90)	Placebo	0.51 (0.09 - 2.77)	0.61 (0.28 - 1.32)	0.57 (0.25 - 1.33)
0.12 (0.01 - 1.44)	0.74 (0.12 - 4.64)	0.51 (0.09 - 2.77)	Ramelteon	.	.
0.13 (0.02 - 0.77)	0.81 (0.30 - 2.23)	0.55 (0.26 - 1.17)	1.09 (0.17 - 7.00)	Zaleplon	1.15 (0.58 - 2.29)
0.14 (0.03 - 0.76)	0.87 (0.29 - 2.60)	0.59 (0.25 - 1.40)	1.17 (0.17 - 7.88)	1.07 (0.55 - 2.10)	Zolpidem

Leaguetable for Increased appetite

Doxepin	.	1.25 (0.29 - 5.39)
0.10 (0.01 - 1.18)	Esmirtazapine	12.58 (1.70 - 93.00)
1.25 (0.29 - 5.39)	12.58 (1.70 - 93.00)	Placebo

Leaguetable for Fatigue

Almorexant	.	.	.	3.78 (0.86 - 16.73)	.	.	.	.	.	.	.	1.17 (0.44 - 3.06)
1.46 (0.44 - 4.92)	Daridorexant	.	.	2.36 (0.97 - 5.74)	.	.	.	.	.	.	.	0.30 (0.08 - 1.15)
2.56 (0.69 - 9.52)	1.75 (0.52 - 5.92)	Esmirtazapine	.	0.86 (0.35 - 2.11)	.	.	.	.	.	.	.	.
0.18 (0.02 - 1.68)	0.12 (0.01 - 1.08)	0.07 (0.01 - 0.64)	Lemborexant	12.09 (1.63 - 89.93)	.	.	.	.	.	.	.	.
2.19 (0.84 - 5.71)	1.50 (0.66 - 3.41)	0.86 (0.35 - 2.11)	12.09 (1.63 - 89.93)	Placebo	0.56 (0.28 - 1.15)	2.75 (1.02 - 7.40)	0.47 (0.28 - 0.77)	0.19 (0.02 - 1.67)	9.33 (0.82 - 105.73)	0.50 (0.04 - 5.62)	0.58 (0.13 - 2.50)	0.76 (0.46 - 1.26)
1.24 (0.37 - 4.08)	0.84 (0.28 - 2.51)	0.48 (0.15 - 1.52)	6.81 (0.81 - 57.33)	0.56 (0.28 - 1.15)	Ramelteon	.	.	.	.	.	.	.
4.02 (1.09 - 14.86)	2.75 (0.80 - 9.44)	1.57 (0.43 - 5.77)	22.16 (2.42 - 203.09)	1.83 (0.72 - 4.69)	3.25 (1.00 - 10.60)	Seltorexant	.	.	.	.	.	1.54 (0.33 - 7.31)
1.02 (0.35 - 3.02)	0.70 (0.27 - 1.84)	0.40 (0.14 - 1.12)	5.64 (0.71 - 44.69)	0.47 (0.28 - 0.77)	0.83 (0.35 - 1.99)	0.25 (0.09 - 0.74)	Suvorexant	.	.	.	.	.
0.34 (0.05 - 2.22)	0.23 (0.04 - 1.47)	0.13 (0.02 - 0.89)	1.87 (0.14 - 25.58)	0.15 (0.03 - 0.83)	0.27 (0.04 - 1.70)	0.08 (0.01 - 0.57)	0.33 (0.06 - 1.91)	Temazepam	.	2.63 (0.50 - 13.93)	.	5.13 (0.59 - 44.87)
20.47 (1.51 - 278.14)	13.99 (1.08 - 181.55)	8.00 (0.60 - 106.50)	112.83 (4.84 - 2631.48)	9.33 (0.82 - 105.73)	16.57 (1.32 - 208.17)	5.09 (0.38 - 68.71)	19.99 (1.68 - 238.46)	60.50 (3.16 - 1159.62)	Tiagabine	.	.	.
0.89 (0.10 - 7.75)	0.61 (0.07 - 5.16)	0.35 (0.04 - 3.10)	4.90 (0.29 - 82.98)	0.41 (0.06 - 2.98)	0.72 (0.09 - 5.99)	0.22 (0.02 - 1.98)	0.87 (0.11 - 6.79)	2.63 (0.50 - 13.93)	0.04 (0.00 - 1.00)	Triazolam	.	1.95 (0.17 - 21.95)
1.27 (0.22 - 7.29)	0.86 (0.16 - 4.65)	0.49 (0.09 - 2.76)	6.97 (0.58 - 83.72)	0.58 (0.13 - 2.50)	1.02 (0.20 - 5.24)	0.31 (0.06 - 1.79)	1.24 (0.26 - 5.82)	3.74 (0.40 - 34.83)	0.06 (0.00 - 1.05)	1.42 (0.12 - 16.93)	Vestipitant	.
1.41 (0.58 - 3.40)	0.96 (0.39 - 2.34)	0.55 (0.19 - 1.56)	7.75 (0.97 - 61.66)	0.64 (0.38 - 1.08)	1.14 (0.47 - 2.76)	0.35 (0.13 - 0.97)	1.37 (0.66 - 2.84)	4.15 (0.77 - 22.34)	0.07 (0.01 - 0.82)	1.58 (0.22 - 11.63)	1.11 (0.23 - 5.27)	Zolpidem

Leaguetable for Pain

Eszopiclone	1.23 (0.86 - 1.77)	.
1.23 (0.86 - 1.77)	Placebo	2.43 (0.20 - 29.66)
3.00 (0.24 - 37.54)	2.43 (0.20 - 29.66)	Zolpidem

Leaguetable for Nasopharyngitis

Daridorexant	.	.	.	.	.	.	0.36 (0.10 - 1.32)	.	.	.	.	.	0.28 (0.08 - 0.96)
0.34 (0.10 - 1.20)	Esmirtazapine	.	.	.	.	.	0.94 (0.53 - 1.67)	.	.	.	.	.	.
0.21 (0.06 - 0.68)	0.61 (0.31 - 1.20)	Eszopiclone	.	.	.	.	1.55 (1.08 - 2.23)	.	.	.	.	.	.
0.32 (0.02 - 4.58)	0.92 (0.07 - 12.16)	1.51 (0.12 - 19.24)	Flurazepam	.	.	.	.	.	.	.	.	.	0.97 (0.09 - 11.09)
0.28 (0.08 - 0.95)	0.82 (0.39 - 1.72)	1.35 (0.74 - 2.44)	0.89 (0.07 - 11.50)	Gaboxadol	.	.	1.15 (0.72 - 1.84)	.	.	.	.	.	.
0.22 (0.04 - 1.10)	0.64 (0.17 - 2.44)	1.05 (0.29 - 3.73)	0.69 (0.04 - 10.90)	0.78 (0.21 - 2.87)	Lemborexant	.	1.04 (0.27 - 3.98)	.	.	.	.	.	3.98 (0.50 - 32.03)
0.23 (0.04 - 1.29)	0.68 (0.16 - 2.80)	1.11 (0.29 - 4.28)	0.73 (0.04 - 12.46)	0.83 (0.21 - 3.29)	1.06 (0.18 - 6.31)	Melatonin	1.39 (0.38 - 5.11)	.	.	.	.	.	.
0.32 (0.11 - 0.99)	0.94 (0.53 - 1.67)	1.55 (1.08 - 2.23)	1.02 (0.08 - 12.67)	1.15 (0.72 - 1.84)	1.48 (0.44 - 5.02)	1.39 (0.38 - 5.11)	Placebo	1.07 (0.80 - 1.42)	1.10 (0.75 - 1.61)	1.21 (0.34 - 4.21)	0.43 (0.10 - 1.81)	1.91 (0.71 - 5.17)	0.91 (0.49 - 1.69)
0.35 (0.11 - 1.10)	1.01 (0.53 - 1.91)	1.65 (1.04 - 2.63)	1.09 (0.09 - 13.75)	1.23 (0.71 - 2.13)	1.58 (0.45 - 5.54)	1.49 (0.39 - 5.63)	1.07 (0.80 - 1.42)	Ramelteon	.	.	.	.	.
0.36 (0.11 - 1.16)	1.04 (0.52 - 2.06)	1.71 (1.01 - 2.89)	1.13 (0.09 - 14.36)	1.27 (0.69 - 2.32)	1.63 (0.46 - 5.85)	1.53 (0.40 - 5.94)	1.10 (0.75 - 1.61)	1.03 (0.64 - 1.66)	Suvorexant	.	.	.	.
0.39 (0.07 - 2.09)	1.14 (0.29 - 4.50)	1.87 (0.51 - 6.88)	1.23 (0.07 - 20.50)	1.39 (0.36 - 5.28)	1.79 (0.31 - 10.25)	1.68 (0.28 - 10.20)	1.21 (0.34 - 4.21)	1.13 (0.31 - 4.08)	1.10 (0.30 - 4.05)	Tasimelteon	.	.	.
0.11 (0.02 - 0.63)	0.33 (0.08 - 1.40)	0.54 (0.14 - 2.14)	0.36 (0.02 - 6.00)	0.40 (0.10 - 1.64)	0.52 (0.09 - 3.10)	0.48 (0.08 - 3.11)	0.35 (0.09 - 1.31)	0.33 (0.08 - 1.27)	0.32 (0.08 - 1.26)	0.29 (0.05 - 1.79)	Triazolam	14.25 (1.66 - 122.00)	.
0.85 (0.22 - 3.21)	2.46 (0.85 - 7.10)	4.03 (1.53 - 10.60)	2.66 (0.20 - 35.11)	2.99 (1.09 - 8.23)	3.86 (0.89 - 16.80)	3.63 (0.75 - 17.56)	2.60 (1.06 - 6.37)	2.44 (0.95 - 6.24)	2.36 (0.89 - 6.26)	2.16 (0.46 - 10.05)	7.49 (1.72 - 32.55)	Zaleplon	0.32 (0.12 - 0.82)
0.31 (0.10 - 0.92)	0.90 (0.38 - 2.10)	1.47 (0.71 - 3.05)	0.97 (0.09 - 11.09)	1.09 (0.50 - 2.40)	1.41 (0.38 - 5.15)	1.32 (0.31 - 5.61)	0.95 (0.50 - 1.79)	0.89 (0.44 - 1.78)	0.86 (0.41 - 1.81)	0.79 (0.19 - 3.20)	2.73 (0.65 - 11.43)	0.36 (0.16 - 0.85)	Zolpidem

Leaguetable for Respiratory problem

Placebo	0.94 (0.12 - 7.52)	0.50 (0.17 - 1.47)
0.94 (0.12 - 7.52)	Temazepam	.
0.50 (0.17 - 1.47)	0.53 (0.05 - 5.56)	Zolpidem

Leaguetable for Anxiety

Placebo	.	1.81 (0.43 - 7.69)	0.36 (0.20 - 0.68)
0.12 (0.01 - 1.26)	Triazolam	.	3.15 (0.31 - 31.62)
1.69 (0.53 - 5.45)	14.60 (1.13 - 188.16)	Zaleplon	0.22 (0.07 - 0.72)
0.37 (0.20 - 0.68)	3.15 (0.31 - 31.62)	0.22 (0.07 - 0.65)	Zolpidem

Leaguetable for Depression

Placebo	0.28 (0.08 - 0.95)	1.13 (0.07 - 19.74)
0.28 (0.08 - 0.95)	Ramelteon	.
1.13 (0.07 - 19.74)	4.03 (0.18 - 89.95)	Zolpidem

Leaguetable for Accidental injury

Daridorexant	.	1.68 (0.67 - 4.20)
1.66 (0.60 - 4.57)	Eszopiclone	1.01 (0.66 - 1.54)
1.68 (0.67 - 4.20)	1.01 (0.66 - 1.54)	Placebo

Leaguetable for Arthralgia

Esmirtazapine	.	.	2.05 (0.45 - 9.32)	.	.
0.72 (0.05 - 11.03)	Eszopiclone	.	2.86 (0.29 - 27.90)	.	.
2.14 (0.38 - 11.97)	2.99 (0.27 - 33.59)	Lemborexant	0.96 (0.42 - 2.17)	.	.
2.05 (0.45 - 9.32)	2.86 (0.29 - 27.90)	0.96 (0.42 - 2.17)	Placebo	0.59 (0.14 - 2.56)	0.72 (0.16 - 3.33)
1.22 (0.15 - 9.99)	1.70 (0.11 - 25.42)	0.57 (0.11 - 3.04)	0.59 (0.14 - 2.56)	Triazolam	1.22 (0.32 - 4.71)
1.48 (0.17 - 12.74)	2.07 (0.13 - 32.11)	0.69 (0.12 - 3.92)	0.72 (0.16 - 3.33)	1.22 (0.32 - 4.71)	Zolpidem

Leaguetable for Back pain

Esmirtazapine	.	.	0.78 (0.20 - 3.07)	.	.	.	.
0.61 (0.15 - 2.59)	Eszopiclone	.	1.27 (0.81 - 1.98)	.	.	.	.
0.51 (0.10 - 2.50)	0.83 (0.33 - 2.09)	Lemborexant	1.53 (0.68 - 3.45)	.	.	.	.
0.78 (0.20 - 3.07)	1.27 (0.81 - 1.98)	1.53 (0.68 - 3.45)	Placebo	0.25 (0.03 - 2.26)	1.86 (0.96 - 3.60)	0.65 (0.22 - 1.96)	0.41 (0.12 - 1.38)
0.20 (0.01 - 2.61)	0.32 (0.03 - 3.01)	0.39 (0.04 - 4.01)	0.25 (0.03 - 2.26)	Ramelteon	.	.	.
1.45 (0.32 - 6.63)	2.36 (1.07 - 5.24)	2.85 (1.00 - 8.12)	1.86 (0.96 - 3.60)	7.37 (0.75 - 72.86)	Suvorexant	.	.
0.51 (0.09 - 2.95)	0.83 (0.25 - 2.72)	1.00 (0.25 - 3.93)	0.65 (0.22 - 1.96)	2.58 (0.22 - 30.09)	0.35 (0.10 - 1.27)	Zaleplon	0.64 (0.28 - 1.46)
0.32 (0.05 - 2.00)	0.53 (0.15 - 1.90)	0.63 (0.15 - 2.72)	0.41 (0.12 - 1.38)	1.64 (0.13 - 20.04)	0.22 (0.06 - 0.88)	0.64 (0.28 - 1.46)	Zolpidem

Leaguetable for Myalgia

Eszopiclone	.	2.18 (0.99 - 4.78)	.	.	.	.	.	.
2.26 (0.19 - 26.41)	Flurazepam	0.97 (0.06 - 16.16)	.	.	.	.	.	0.97 (0.09 - 11.09)
2.18 (0.99 - 4.78)	0.97 (0.09 - 9.94)	Placebo	0.49 (0.22 - 1.09)	4.46 (0.73 - 27.22)	1.14 (0.42 - 3.11)	1.34 (0.47 - 3.77)	0.68 (0.29 - 1.58)	1.02 (0.56 - 1.87)
1.08 (0.35 - 3.29)	0.48 (0.04 - 5.60)	0.49 (0.22 - 1.09)	Ramelteon	.	.	.	.	.
11.77 (1.94 - 71.50)	5.22 (0.32 - 85.04)	5.40 (1.07 - 27.40)	10.95 (1.80 - 66.82)	Seltorexant	.	.	.	0.16 (0.03 - 0.90)
2.37 (0.70 - 8.03)	1.05 (0.09 - 12.35)	1.09 (0.43 - 2.76)	2.21 (0.65 - 7.52)	0.20 (0.03 - 1.24)	Temazepam	1.17 (0.41 - 3.39)	.	0.97 (0.35 - 2.73)
2.78 (0.80 - 9.68)	1.23 (0.10 - 14.69)	1.28 (0.49 - 3.36)	2.59 (0.74 - 9.06)	0.24 (0.04 - 1.48)	1.17 (0.41 - 3.39)	Triazolam	.	0.83 (0.29 - 2.40)
1.62 (0.56 - 4.71)	0.72 (0.07 - 7.81)	0.74 (0.36 - 1.53)	1.51 (0.52 - 4.42)	0.14 (0.02 - 0.76)	0.68 (0.23 - 2.04)	0.58 (0.19 - 1.79)	Zaleplon	1.25 (0.55 - 2.80)
2.20 (0.81 - 5.95)	0.97 (0.10 - 9.76)	1.01 (0.55 - 1.86)	2.04 (0.75 - 5.58)	0.19 (0.04 - 0.93)	0.93 (0.36 - 2.37)	0.79 (0.30 - 2.10)	1.36 (0.67 - 2.74)	Zolpidem

Leaguetable for Eye pain

Placebo	0.62 (0.24 - 1.55)	0.39 (0.12 - 1.33)	0.40 (0.10 - 1.60)
0.62 (0.24 - 1.55)	Ramelteon	.	.
0.39 (0.12 - 1.33)	0.64 (0.14 - 2.95)	Zaleplon	1.03 (0.43 - 2.47)
0.40 (0.10 - 1.60)	0.65 (0.12 - 3.44)	1.03 (0.43 - 2.47)	Zolpidem

Leaguetable for Infection

Eszopiclone	1.81 (1.28 - 2.54)	.	.
1.81 (1.28 - 2.54)	Placebo	0.92 (0.29 - 2.86)	0.41 (0.12 - 1.38)
1.65 (0.50 - 5.44)	0.92 (0.29 - 2.86)	Zaleplon	0.45 (0.19 - 1.09)
0.75 (0.21 - 2.62)	0.41 (0.12 - 1.38)	0.45 (0.19 - 1.09)	Zolpidem

Leaguetable for Upper respiratory tract infection

Daridorexant	.	.	.	.	0.55 (0.22 - 1.36)	.	.	.	.	.
0.52 (0.11 - 2.48)	Doxepin	.	.	.	1.06 (0.30 - 3.78)	.	.	.	.	.
0.69 (0.20 - 2.37)	1.32 (0.29 - 6.08)	Esmirtazapine	.	.	0.80 (0.35 - 1.86)	.	.	.	.	.
0.60 (0.22 - 1.61)	1.15 (0.30 - 4.36)	0.87 (0.34 - 2.20)	Gaboxadol	.	0.92 (0.62 - 1.39)	.	.	.	.	.
0.51 (0.17 - 1.52)	0.97 (0.24 - 4.02)	0.74 (0.26 - 2.10)	0.85 (0.40 - 1.79)	Lemborexant	1.06 (0.56 - 2.01)	.	.	.	.	1.73 (0.36 - 8.40)
0.55 (0.22 - 1.36)	1.06 (0.30 - 3.78)	0.80 (0.35 - 1.86)	0.92 (0.62 - 1.39)	1.09 (0.58 - 2.04)	Placebo	0.82 (0.50 - 1.37)	0.68 (0.28 - 1.64)	1.00 (0.33 - 2.99)	3.77 (0.76 - 18.72)	1.48 (0.58 - 3.78)
0.45 (0.16 - 1.28)	0.87 (0.22 - 3.43)	0.66 (0.25 - 1.76)	0.76 (0.40 - 1.45)	0.90 (0.40 - 2.00)	0.82 (0.50 - 1.37)	Ramelteon	.	.	.	.
0.37 (0.10 - 1.32)	0.72 (0.15 - 3.37)	0.54 (0.16 - 1.83)	0.62 (0.24 - 1.65)	0.74 (0.25 - 2.17)	0.68 (0.28 - 1.64)	0.82 (0.30 - 2.28)	Suvorexant	.	.	.
0.60 (0.15 - 2.39)	1.15 (0.22 - 5.98)	0.87 (0.23 - 3.34)	1.00 (0.33 - 3.09)	1.18 (0.36 - 3.87)	1.09 (0.38 - 3.10)	1.32 (0.41 - 4.23)	1.61 (0.41 - 6.34)	Temazepam	3.77 (0.76 - 18.72)	1.15 (0.37 - 3.58)
2.25 (0.37 - 13.83)	4.34 (0.57 - 32.77)	3.28 (0.55 - 19.50)	3.79 (0.75 - 19.19)	4.45 (0.84 - 23.60)	4.10 (0.85 - 19.73)	4.98 (0.95 - 25.94)	6.06 (1.00 - 36.80)	3.77 (0.76 - 18.72)	Triazolam	0.31 (0.06 - 1.56)
0.76 (0.21 - 2.75)	1.46 (0.30 - 7.00)	1.10 (0.32 - 3.83)	1.27 (0.47 - 3.47)	1.50 (0.53 - 4.19)	1.38 (0.55 - 3.45)	1.67 (0.59 - 4.77)	2.04 (0.57 - 7.29)	1.27 (0.43 - 3.72)	0.34 (0.07 - 1.65)	Zolpidem

Leaguetable for Urinary tract infection

Doxepin	.	.	0.28 (0.05 - 1.67)	.	.	.	.	.
0.24 (0.04 - 1.63)	Indiplon	.	1.17 (0.58 - 2.34)	.	.	.	.	.
0.20 (0.03 - 1.41)	0.84 (0.30 - 2.35)	Lemborexant	1.28 (0.59 - 2.78)	.	.	.	.	3.00 (0.67 - 13.50)
0.28 (0.05 - 1.67)	1.17 (0.58 - 2.34)	1.39 (0.65 - 2.96)	Placebo	0.97 (0.43 - 2.17)	6.03 (1.08 - 33.61)	2.09 (1.11 - 3.95)	0.34 (0.04 - 2.89)	0.78 (0.22 - 2.76)
0.27 (0.04 - 1.93)	1.13 (0.39 - 3.29)	1.34 (0.44 - 4.07)	0.97 (0.43 - 2.17)	Ramelteon	.	.	.	.
1.70 (0.14 - 20.20)	7.05 (1.10 - 45.00)	8.37 (1.28 - 54.74)	6.03 (1.08 - 33.61)	6.23 (0.93 - 41.65)	Seltorexant	.	.	.
0.59 (0.09 - 3.91)	2.44 (0.95 - 6.28)	2.90 (1.08 - 7.81)	2.09 (1.11 - 3.95)	2.16 (0.77 - 6.05)	0.35 (0.06 - 2.17)	Suvorexant	.	.
0.10 (0.01 - 1.55)	0.40 (0.04 - 3.77)	0.48 (0.05 - 4.57)	0.34 (0.04 - 2.89)	0.35 (0.04 - 3.46)	0.06 (0.00 - 0.88)	0.16 (0.02 - 1.52)	Tasimelteon	.
0.33 (0.04 - 2.75)	1.35 (0.34 - 5.29)	1.60 (0.49 - 5.24)	1.15 (0.36 - 3.74)	1.19 (0.29 - 4.98)	0.19 (0.02 - 1.54)	0.55 (0.14 - 2.10)	3.37 (0.30 - 38.36)	Zolpidem

Leaguetable for Sinusitis

Doxepin	.	.	1.54 (0.16 - 15.03)	.
0.51 (0.02 - 11.63)	Esmirtazapine	.	2.99 (0.36 - 25.08)	.
2.09 (0.19 - 22.87)	4.06 (0.43 - 38.45)	Eszopiclone	0.74 (0.36 - 1.53)	.
1.54 (0.16 - 15.03)	2.99 (0.36 - 25.08)	0.74 (0.36 - 1.53)	Placebo	0.25 (0.08 - 0.86)
0.39 (0.03 - 5.19)	0.76 (0.07 - 8.82)	0.19 (0.05 - 0.77)	0.25 (0.08 - 0.86)	Zolpidem

Appendix 14 Results of posterior mean of the overall residual deviance for all outcomes

Outcomes	Random effect model	Fixed effect model	Final model
**Primary outcomes**			
Somnolence	239.48656	240.33007	Random
Dizziness	249.20358	249.02159	Fixed
Headache	266.2841	266.2560	Fixed
Amnesia	16.498883	16.323694	Fixed
Dysgeusia	75.91858	76.19476	Random
Difficulty concentrating	19.671848	19.689505	Random
Impaired coordination	17.881270	17.873337	Fixed
Nervousness	52.57567	52.64294	Random
Nightmare	36.50582	36.60976	Random
Asthenia	36.98769	36.88850	Fixed
Dyspepsia	75.91858	76.19476	Random
Diarrhea	86.01477	85.94166	Fixed
Dry mouth	109.43152	109.73511	Random
Nausea/vomiting	172.37396	173.06546	Random
Constipation	23.80382	23.70062	Fixed
Abdominal pain	32.98517	32.90654	Fixed
Increased appetite	10.865606	10.856028	Fixed
Fatigue	100.49584	100.45484	Fixed
Pain	22.83986	22.88959	Random
Nasopharyngitis	118.59948	118.34202	Fixed
Respiratory problem	10.962317	10.908738	Fixed
Anxiety	35.94072	35.93834	Fixed
Confusional state	20.310012	20.318675	Random
Depression	23.57313	23.67626	Random
Emotional lability	19.982471	19.994643	Random
Accidental injury	20.63861	20.680305	Random
Arthralgia	18.942218	18.945547	Random
Back pain	51.35186	51.30161	Fixed
Myalgia	49.85065	49.91595	Random
Eye pain	13.536759	13.409594	Fixed
Infection	14.148974	14.082012	Fixed
Upper respiratory tract infection	74.86891	74.87883	Random
Urinary tract infection	57.21054	57.27741	Random
Sinusitis	19.306472	19.369732	Random
**Secondary outcomes**			
Abnormal dreams	31.51422	31.47516	Fixed
Abnormal vision	14.265046	14.281539	Random
Gait disturbance	6.771541	6.778507	Random
Hypnagogic hallucinations	6.796999	6.769555	Fixed
Tremor	4.678108	4.693885	Random
Paresthesia	10.494292	10.455490	Fixed
Gastroenteritis	22.64441	22.60179	Fixed
Decreased appetite	11.422219	11.370232	Fixed
Pain gastralgia	2.1948899	2.1688587	Fixed
Insomnia exacerbated	10.797107	10.851963	Random
Sleep paralysis	21.65390	21.63701	Fixed
Peripheral oedema	16.311686	16.314797	Random

Pruritis	7.074316	7.141428	Random
Sweating	6.526866	6.482475	Fixed
Skin diseases	19.210158	18.951884	Fixed
Influenza	22.87381	23.01572	Random
Common cold	8.329710	8.344070	Random
Malaise	8.656639	8.702773	Random
Cough	21.11418	21.04091	Fixed
Suicidal ideation	4.612472	4.529400	Fixed
Hallucinations*	/	/	Random
Irritability	7.038171	6.989740	Fixed
Falls	14.668736	14.710499	Random
Laceration	4.733728	4.645565	Fixed
Dry eyes*	/	/	Random
Alanine aminotransferase increased	7.247424	7.167859	Fixed
Blood creatine phosphokinase increased	10.850340	10.908432	Random
Weight increased	9.181849	9.166523	Fixed
γ -Glutamyl transferase increased	14.496699	14.511631	Random
Hyperglycaemia	6.816273	6.817032	Random
Dysmenorrhea	16.713205	16.663713	Fixed
Hypertension	13.614259	13.559236	Fixed
Tachycardia	5.107045	5.057809	Fixed
Haematuria*	/	/	Random
Memory disorders	16.605104	16.522636	Fixed

*There were only results of paired meta-analysis for this outcome

Appendix 15 Reference list of included studies

1. Lydiard RB, Lankford DA, Seiden DJ, Landin R, Farber R, Walsh JK. Efficacy and tolerability of modified-release indiplon in elderly patients with chronic insomnia: results of a 2-week double-blind, placebo-controlled trial. J Clin Sleep Med. 2006 Jul 15;2(3):309-15.
2. Murphy P, Moline M, Mayleben D, Rosenberg R, Zammit G, Pinner K, Dhadda S, Hong Q, Giorgi L, Satlin A. Lemborexant, A Dual Orexin Receptor Antagonist (DORA) for the Treatment of Insomnia Disorder: Results From a Bayesian, Adaptive, Randomized, Double-Blind, Placebo-Controlled Study. J Clin Sleep Med. 2017 Nov 15;13(11):1289-1299. doi: 10.5664/jcsm.6800.
3. Black J, Pillar G, Hedner J, Polo O, Berkani O, Mangialaio S, Hmissi A, Zammit G, Hajak G. Efficacy and safety of almorexant in adult chronic insomnia: a randomized placebo-controlled trial with an active reference. Sleep Med. 2017 Aug;36:86-94. doi: 10.1016/j.sleep.2017.05.009.
4. Pinto LR Jr, Bittencourt LR, Treptow EC, Braga LR, Tufik S. Eszopiclone versus zopiclone in the treatment of insomnia. Clinics (Sao Paulo). 2016 Jan;71(1):5-9. doi: 10.6061/clinics/2016(01)02.
5. Herring WJ, Connor KM, Ivgy-May N, Snyder E, Liu K, Snavely DB, Krystal AD, Walsh JK, Benca RM, Rosenberg R, Sangal RB, Budd K, Hutzelmann J, Leibensperger H, Froman S, Lines C, Roth T, Michelson D. Suvorexant in Patients With Insomnia: Results From Two 3-Month Randomized Controlled Clinical Trials. Biol Psychiatry. 2016 Jan 15;79(2):136-48. doi: 10.1016/j.biopsych.2014.10.003.
6. Michelson D, Snyder E, Paradis E, Chengan-Liu M, Snavely DB, Hutzelmann J, Walsh JK, Krystal AD, Benca RM, Cohn M, Lines C, Roth T, Herring WJ. Safety and efficacy of suvorexant during 1-year treatment of insomnia with subsequent abrupt treatment discontinuation: a phase 3 randomised, double-blind, placebo-controlled trial. Lancet Neurol. 2014 May;13(5):461-71. doi: 10.1016/S1474-4422(14)70053-5.
7. Ratti E, Carpenter DJ, Zamuner S, Fernandes S, Squassante L, Danker-Hopfe H, Archer G, Robertson J, Alexander R, Trist DG, Merlo-Pich E. Efficacy of vestipitant, a neurokinin-1 receptor antagonist, in primary insomnia. Sleep. 2013 Dec 1;36(12):1823-30. doi: 10.5665/sleep.3208.
8. Roth T, Krystal A, Steinberg FJ, Singh NN, Moline M. Novel sublingual low-dose zolpidem tablet reduces latency to sleep onset following spontaneous middle-of-the-night awakening in insomnia in a randomized, double-blind, placebo-controlled, outpatient study. Sleep. 2013 Feb 1;36(2):189-96. doi: 10.5665/sleep.2370.
9. Lankford A, Rogowski R, Essink B, Ludington E, Heith Durrence H, Roth T. Efficacy and safety of doxepin 6 mg in a four-week outpatient trial of elderly adults with chronic primary insomnia. Sleep Med. 2012 Feb;13(2):133-8. doi: 10.1016/j.sleep.2011.09.006.
10. Fan B, Kang J , He Y , et al. Efficacy and safety of suvorexant for the treatment of primary insomnia among Chinese: A 6-month randomized double-blind controlled study. 2017.
11. Roth AJ, McCall WV, Liguori A. Cognitive, psychomotor and polysomnographic effects of trazodone in primary insomniacs. J Sleep Res. 2011 Dec;20(4):552-8. doi: 10.1111/j.1365-2869.2011.00928.x. Epub 2011 May 30.

12. Huang YS, Hsu SC, Liu SI, Chen CK. A double-blind, randomized, comparative study to evaluate the efficacy and safety of zaleplon versus zolpidem in shortening sleep latency in primary insomnia. Chang Gung Med J. 2011 Jan-Feb;34(1):50-6.
13. Uchimura N, Ogawa A, Hamamura M, Hashimoto T, Nagata H, Uchiyama M. Efficacy and safety of ramelteon in Japanese adults with chronic insomnia: a randomized, double-blind, placebo-controlled study. Expert Rev Neurother. 2011 Feb;11(2):215-24. doi: 10.1586/ern.10.197.
14. Ivgy-May N, Hajak G, van Osta G, Braat S, Chang Q, Roth T. Efficacy and safety of esmirtazapine in adult outpatients with chronic primary insomnia: a randomized, double-blind placebo-controlled study and open-label extension. J Clin Sleep Med. 2020 Sep 15;16(9):1455-1467. doi: 10.5664/jcsm.8526.
15. Hajak G, Hedner J, Eglin M, Loft H, Stórustovu SI, Lütolf S, Lundahl J; Gaboxadol Study 99775 Group. A 2-week efficacy and safety study of gaboxadol and zolpidem using electronic diaries in primary insomnia outpatients. Sleep Med. 2009 Aug;10(7):705-12. doi: 10.1016/j.sleep.2008.09.010.
16. Uchiyama M, Hamamura M, Kuwano T, Nishiyama H, Nagata H, Uchimura N. Evaluation of subjective efficacy and safety of ramelteon in Japanese subjects with chronic insomnia. Sleep Med. 2011 Feb;12(2):119-26. doi: 10.1016/j.sleep.2010.08.010. Epub 2011 Jan 21.
17. Krystal AD, Durrence HH, Scharf M, Jochelson P, Rogowski R, Ludington E, Roth T. Efficacy and Safety of Doxepin 1 mg and 3 mg in a 12-week Sleep Laboratory and Outpatient Trial of Elderly Subjects with Chronic Primary Insomnia. Sleep. 2010 Nov;33(11):1553-61. doi: 10.1093/sleep/33.11.1553.
18. Ancoli-Israel S, Krystal AD, McCall WV, Schaefer K, Wilson A, Claus R, Rubens R, Roth T. A 12-week, randomized, double-blind, placebo-controlled study evaluating the effect of eszopiclone 2 mg on sleep/wake function in older adults with primary and comorbid insomnia. Sleep. 2010 Feb;33(2):225-34. doi: 10.1093/sleep/33.2.225.
19. Walsh JK, Salkeld L, Knowles LJ, Tasker T, Hunneyball IM. Treatment of elderly primary insomnia patients with EVT 201 improves sleep initiation, sleep maintenance, and daytime sleepiness. Sleep Med. 2010 Jan;11(1):23-30. doi: 10.1016/j.sleep.2009.07.012.
20. Luthringer R, Muzet M, Zisapel N, Staner L. The effect of prolonged-release melatonin on sleep measures and psychomotor performance in elderly patients with insomnia. Int Clin Psychopharmacol. 2009 Sep;24(5):239-49. doi: 10.1097/YIC.0b013e32832e9b08.
21. Mayer G, Wang-Weigand S, Roth-Schechter B, Lehmann R, Staner C, Partinen M. Efficacy and safety of 6-month nightly ramelteon administration in adults with chronic primary insomnia. Sleep. 2009 Mar;32(3):351-60. doi: 10.1093/sleep/32.3.351.
22. Krystal AD, Erman M, Zammit GK, Soubrane C, Roth T; ZOLONG Study Group. Long-term efficacy and safety of zolpidem extended-release 12.5 mg, administered 3 to 7 nights per week for 24 weeks, in patients with chronic primary insomnia: a 6-month, randomized, double-blind, placebo-controlled, parallel-group, multicenter study. Sleep. 2008 Jan;31(1):79-90. doi: 10.1093/sleep/31.1.79.
23. Walsh JK, Soubrane C, Roth T. Efficacy and safety of zolpidem extended release in elderly primary insomnia patients. Am J Geriatr Psychiatry. 2008 Jan;16(1):44-57. doi:

10.1097/JGP.0b013e3181256b01.
24. Lemoine P, Nir T, Laudon M, Zisapel N. Prolonged-release melatonin improves sleep quality and morning alertness in insomnia patients aged 55 years and older and has no withdrawal effects. J Sleep Res. 2007 Dec;16(4):372-80. doi: 10.1111/j.1365-2869.2007.00613.x.
25. Roth T, Seiden D, Sainati S, Wang-Weigand S, Zhang J, Zee P. Effects of ramelteon on patient-reported sleep latency in older adults with chronic insomnia. Sleep Med. 2006 Jun;7(4):312-8. doi: 10.1016/j.sleep.2006.01.003.
26. Ivgy-May N, Ruwe F, Krystal A, Roth T. Esmirtazapine in non-elderly adult patients with primary insomnia: efficacy and safety from a randomized, 6-week sleep laboratory trial. Sleep Med. 2015 Jul;16(7):838-44. doi: 10.1016/j.sleep.2015.04.001.
27. Ivgy-May N, Roth T, Ruwe F, Walsh J. Esmirtazapine in non-elderly adult patients with primary insomnia: efficacy and safety from a 2-week randomized outpatient trial. Sleep Med. 2015 Jul;16(7):831-7. doi: 10.1016/j.sleep.2015.03.005.
28. Wade AG, Ford I, Crawford G, McMahon AD, Nir T, Laudon M, Zisapel N. Efficacy of prolonged release melatonin in insomnia patients aged 55-80 years: quality of sleep and next-day alertness outcomes. Curr Med Res Opin. 2007 Oct;23(10):2597-605. doi: 10.1185/030079907X233098.
29. Walsh, James K , et al. "Efficacy and tolerability of indiplon in older adults with primary insomnia." Sleep Medicine 8.7-8(2007):753-759.
30. Zammit G, Erman M, Wang-Weigand S, Sainati S, Zhang J, Roth T. Evaluation of the efficacy and safety of ramelteon in subjects with chronic insomnia. J Clin Sleep Med. 2007 Aug 15;3(5):495-504. Erratum in: J Clin Sleep Med. 2007 Oct 15;3(6):table of contents. Erratum in: J Clin Sleep Med. 2008 Oct 15;4(5): table of contents.
31. Walsh JK, Krystal AD, Amato DA, Rubens R, Caron J, Wessel TC, Schaefer K, Roach J, Wallenstein G, Roth T. Nightly treatment of primary insomnia with eszopiclone for six months: effect on sleep, quality of life, and work limitations. Sleep. 2007 Aug;30(8):959-68. doi: 10.1093/sleep/30.8.959.
32. Scharf MB, Black J, Hull S, Landin R, Farber R. Long-term nightly treatment with indiplon in adults with primary insomnia: results of a double-blind, placebo-controlled, 3-month study. Sleep. 2007 Jun;30(6):743-52. doi: 10.1093/sleep/30.6.743.
33. Walsh JK, Perlis M, Rosenthal M, Krystal A, Jiang J, Roth T. Tiagabine increases slow-wave sleep in a dose-dependent fashion without affecting traditional efficacy measures in adults with primary insomnia. J Clin Sleep Med. 2006 Jan 15;2(1):35-41.
34. McCall WV, Erman M, Krystal AD, Rosenberg R, Scharf M, Zammit GK, Wessel T. A polysomnography study of eszopiclone in elderly patients with insomnia. Curr Med Res Opin. 2006 Sep;22(9):1633-42. doi: 10.1185/030079906X112741.
35. Roth T, Soubrane C, Titeux L, Walsh JK; Zoladult Study Group. Efficacy and safety of zolpidem-MR: a double-blind, placebo-controlled study in adults with primary insomnia. Sleep Med. 2006 Aug;7(5):397-406. doi: 10.1016/j.sleep.2006.04.008.
36. Scharf M, Erman M, Rosenberg R, Seiden D, McCall WV, Amato D, Wessel TC. A 2-week efficacy and safety study of eszopiclone in elderly patients with primary insomnia. Sleep. 2005 Jun;28(6):720-7. doi: 10.1093/sleep/28.6.720.

37. Zammit GK, McNabb LJ, Caron J, Amato DA, Roth T. Efficacy and safety of eszopiclone across 6-weeks of treatment for primary insomnia. Curr Med Res Opin. 2004 Dec;20(12):1979-91. doi: 10.1185/174234304x15174.
38. Allain, Herv , L. Arbus , and Stéphane Schück. "Efficacy and Safety of Zolpidem Administered As Needed in Primary Insomnia: Results of a Double-Blind, Placebo-Controlled Study." Clinical Drug Investigation 21.6(2001):391-400.
39. Krystal AD, Walsh JK, Laska E, Caron J, Amato DA, Wessel TC, Roth T. Sustained efficacy of eszopiclone over 6 months of nightly treatment: results of a randomized, double-blind, placebo-controlled study in adults with chronic insomnia. Sleep. 2003 Nov 1;26(7):793-9. doi: 10.1093/sleep/26.7.793.
40. Morin CM, Bastien CH, Brink D, Brown TR. Adverse effects of temazepam in older adults with chronic insomnia. Hum Psychopharmacol. 2003 Jan;18(1):75-82. doi: 10.1002/hup.454.
41. Riemann D, Voderholzer U, Cohrs S, Rodenbeck A, Hajak G, Rüther E, Wiegand MH, Laakmann G, Baghai T, Fischer W, Hoffmann M, Hohagen F, Mayer G, Berger M. Trimipramine in primary insomnia: results of a polysomnographic double-blind controlled study. Pharmacopsychiatry. 2002 Sep;35(5):165-74. doi: 10.1055/s-2002-34119.
42. Tsutsui S; Zolipidem Study Group. A double-blind comparative study of zolpidem versus zopiclone in the treatment of chronic primary insomnia. J Int Med Res. 2001 May-Jun;29(3):163-77. doi: 10.1177/147323000102900303.
43. Krystal AD, Huang H, Zummo J, Grinnell T, Marshall RD. A WASO sub-group analysis of a 6-month study of eszopiclone 3 mg. Sleep Med. 2012 Jun;13(6):691-6. doi: 10.1016/j.sleep.2012.01.010. Epub 2012 Mar 31.
44. Zhanghui, Shenyang, Liu Na, Xiao Weizhong. Efficacy and safety of zaleplon in the treatment of insomnia: a randomized controlled trial. Chinese journal of clinical rehabilitation. 2004: 8 (18): 3488-3490
45. Roth T, Lines C, Vandormael K, Ceesay P, Anderson D, Snavely D. Effect of gaboxadol on patient-reported measures of sleep and waking function in patients with Primary Insomnia: results from two randomized, controlled, 3-month studies. J Clin Sleep Med. 2010 Feb 15;6(1):30-9.
46. Fry J, Scharf M, Mangano R, Fujimori M. Zaleplon improves sleep without producing rebound effects in outpatients with insomnia. Zaleplon Clinical Study Group. Int Clin Psychopharmacol. 2000 May;15(3):141-52. doi: 10.1097/00004850-200015030-00003.
47. Elie R, Rüther E, Farr I, Emilien G, Salinas E. Sleep latency is shortened during 4 weeks of treatment with zaleplon, a novel nonbenzodiazepine hypnotic. Zaleplon Clinical Study Group. J Clin Psychiatry. 1999 Aug;60(8):536-44. doi: 10.4088/jcp.v60n0806.
48. Walsh, J. K. , Fry, J. , Erwin, C. W. , Scharf, M. , Roth, T. , & Vogel, G. W. Efficacy and tolerability of 14-day administration of zaleplon 5mg and 10mg for the treatment of primary insomnia. Clinical Drug Investigation, 1998; 16(5), 347-354.
49. Walsh, J. K, Erman M, Erwin CW, et al. "Subjective hypnotic efficacy of trazodone and zolpidem in DSMIII–R primary insomnia." Human Psychopharmacology Clinical & Experimental 13.3(1998):191–198.
50. Lahmeyer, H. , Wilcox, C. S. , Kann, J. , & Leppik, I. Subjective efficacy of zolpidem in

outpatients with chronic insomnia. Clinical Drug Investigation, 1997; 13(3), 134-144.
51. Fleming, D. J. , Moldofsky, H. , & JK Walsh. Comparison of the residual effects and efficacy of short-term zolpidem, flurazepam and placebo in patients with chronic insomnia. Clinical Drug Investigation,1995; 9(6), 303-313.
52. Roth T, Wright KP Jr, Walsh J. Effect of tiagabine on sleep in elderly subjects with primary insomnia: a randomized, double-blind, placebo-controlled study. Sleep. 2006 Mar;29(3):335-41. doi: 10.1093/sleep/29.3.335.
53. Allain, H, Coz, F. L. , Borderies, P. , Schuck, S. , & Gandon, J. M.. Use of zolpidem 10 mg as a benzodiazepine substitute in 84 patients with insomnia. Human Psychopharmacology Clinical and Experimental, 1998; 13(8), 551-559.
54. Leppik, G., Barbara, & Roth-Schechter, et al. Double-blind, placebo-controlled comparison of zolpidem, triazolam, and temazepam in elderly patients with insomnia. Drug Development Research. 1997
55. Dockhorn RJ, Dockhorn DW. Zolpidem in the treatment of short-term insomnia: a randomized, double-blind, placebo-controlled clinical trial. Clin Neuropharmacol. 1996 Aug;19(4):333-40. doi: 10.1097/00002826-199619040-00006.
56. Dehlin O, Rubin B, Rundgren A. Double-blind comparison of zopiclone and flunitrazepam in elderly insomniacs with special focus on residual effects. Curr Med Res Opin. 1995;13(6):317-24. doi: 10.1185/03007999509110492.
57. Rosenberg J, Ahlstrøm F. Randomized, double blind trial of zolpidem 10 mg versus triazolam 0.25 mg for treatment of insomnia in general practice. Scand J Prim Health Care. 1994 Jun;12(2):88-92. doi: 10.3109/02813439409003681.
58. Scharf MB, Roth PB, Dominguez RA, Ware JC. Estazolam and flurazepam: a multicenter, placebo-controlled comparative study in outpatients with insomnia. J Clin Pharmacol. 1990 May;30(5):461-7. doi: 10.1002/j.1552-4604.1990. tb03486.x.
59. Tamminen T, Hansen PP. Chronic administration of zopiclone and nitrazepam in the treatment of insomnia. Sleep. 1987;10 Suppl 1:63-72.
60. Klimm HD, Dreyfus JF, Delmotte M. Zopiclone versus nitrazepam: a double-blind comparative study of efficacy and tolerance in elderly patients with chronic insomnia. Sleep. 1987;10 Suppl 1:73-8. doi: 10.1093/sleep/10.suppl_1.73.
61. Jovanovic UJ, Dreyfus JF. Polygraphical sleep recordings in insomniac patients under zopiclone or nitrazepam. Pharmacology. 1983;27 Suppl 2:136-45. doi: 10.1159/000137920.
62. Zhou CL, Xie HJ, Wang LQ, Tang XF. Clinical observation of paroxetine in the treatment of chronic primary insomnia in the elderly. Chinese Journal of Gerontology. 2002; 3 (21): 185-187.
63. Kärppä M, Yardley J, Pinner K, Filippov G, Zammit G, Moline M, Perdomo C, Inoue Y, Ishikawa K, Kubota N. Long-term efficacy and tolerability of lemborexant compared with placebo in adults with insomnia disorder: results from the phase 3 randomized clinical trial SUNRISE 2. Sleep. 2020 Sep 14;43(9):zsaa123. doi: 10.1093/sleep/zsaa123.
64. Dauvilliers Y, Zammit G, Fietze I, Mayleben D, Seboek Kinter D, Pain S, Hedner J. Daridorexant, a New Dual Orexin Receptor Antagonist to Treat Insomnia Disorder. Ann Neurol. 2020 Mar;87(3):347-356. doi: 10.1002/ana.25680. Epub 2020 Feb 5. Erratum in: Ann Neurol.

2020 Sep;88(3):647-651.
65. Sivertsen B, Omvik S, Pallesen S, Bjorvatn B, Havik OE, Kvale G, Nielsen GH, Nordhus IH. Cognitive behavioral therapy vs zopiclone for treatment of chronic primary insomnia in older adults: a randomized controlled trial. JAMA. 2006 Jun 28;295(24):2851-8. doi: 10.1001/jama.295.24.2851
66. Herrmann WM, Kubicki ST, Boden S, Eich FX, Attali P, Coquelin JP. Pilot controlled double-blind study of the hypnotic effects of zolpidem in patients with chronic 'learned' insomnia: psychometric and polysomnographic evaluation. J Int Med Res. 1993 Nov-Dec;21(6):306-22. doi: 10.1177/030006059302100602. PMID: 8143886.
67. Takeda. 2007. NCT00492232. Facilitation of Zolpidem (≥10 mg) Discontinuation Through Use of Ramelteon in Subjects With Chronic Insomnia. https://clinicaltrials.gov/ct2/show/study/NCT00492232?rslt=With&type=Intr&cond=insomnia&age=12&draw=3
68. Katz, 2011. NCT01489969. Sleep Laboratory Study to Investigate the Safety and Efficacy of Neu-P11 in Primary Insomnia Patients. https://clinicaltrials.gov/ct2/show/study/NCT01489969?rslt=With&type=Intr&cond=insomnia&age=12&draw=3
69. NCT00548340. VEC-162 Study in Adult Patients With Primary Insomnia. https://clinicaltrials.gov/ct2/show/NCT00548340?rslt=With&type=Intr&cond=insomnia&age=12&draw=7&rank=59
70. NCT01463098. A 2-Part Single Dose Study to Assess the Safety, Tolerability, Pharmacokinetics, and Pharmacodynamics of E2006. https://clinicaltrials.gov/ct2/show/results/NCT01463098?rslt=With&type=Intr&cond=insomnia&age=12&draw=11&rank=77
71. Scharf MB, Roth T, Vogel GW, Walsh JK. A multicenter, placebo-controlled study evaluating zolpidem in the treatment of chronic insomnia. J Clin Psychiatry. 1994;55(5):192-9.
72. Soares CN, Joffe H, Rubens R, Caron J, Roth T, Cohen L. Eszopiclone in patients with insomnia during perimenopause and early postmenopause: a randomized controlled trial. Obstet Gynecol. 2006;108(6):1402-10.
73. Roth T, Zammit GK, Scharf MB, Farber R. Efficacy and safety of as-needed, post bedtime dosing with indiplon in insomnia patients with chronic difficulty maintaining sleep. Sleep. 2007 Dec;30(12):1731-8. doi: 10.1093/sleep/30.12.1731.
74. Cordingley GJ, Dean BC, Harris RI. A double-blind comparison of two benzodiazepine hypnotics, flunitrazepam and triazolam, in general practice. Curr Med Res Opin. 1984;8(10):714-9. doi: 10.1185/03007998409110122. PMID: 6144456.
75. Dahl LE, Dencker SJ, Lundin L, Kullingsjö H. Comparison of nitrazepam with triazolam in insomniac outpatients. Acta Psychiatr Scand. 1982 Feb;65(2):86-92. doi: 10.1111/j.1600-0447.1982.tb00826.x. PMID: 6138924.
76. Fabre LF Jr, Gross L, Pasigajen V, Metzler C. Multiclinic double-blind comparison of triazolam and flurazepam for seven nights in outpatients with insomnia. J Clin Pharmacol. 1977 Jul;17(7):402-9. doi: 10.1002/j.1552-4604.1977.tb04623.x. PMID: 18492.
77. Allen RP, Mendels J, Nevins DB, Chernik DA, Hoddes E. Efficacy without tolerance or rebound

insomnia for midazolam and temazepam after use for one to three months. J Clin Pharmacol. 1987 Oct;27(10):768-75. doi: 10.1002/j.1552-4604.1987.tb02994.x. PMID: 2892863.

78. Anderson AA. Zopiclone and nitrazepam: a multicenter placebo controlled comparative study of efficacy and tolerance in insomniac patients in general practice. Sleep. 1987;10 Suppl 1:54-62. doi: 10.1093/sleep/10.suppl_1.54. PMID: 3326116.
79. Bayer AJ, Pathy MS. Clinical and psychometric evaluation of two doses of loprazolam and placebo in geriatric patients. Curr Med Res Opin. 1986;10(1):17-24. doi: 10.1185/03007998609111088. PMID: 2870876.
80. Dominguez RA, Goldstein BJ, Jacobson AF, Steinbook RM. Hypnotic efficacy of a modified triazolodiazepine, brotizolam. Clin Pharmacol Ther. 1985 Jun;37(6):674-9. doi: 10.1038/clpt.1985.110. PMID: 3891191.
81. F. Goldenberg,I. Hindmarch,C. R. B. Joyce,M. Le Gal,M. Partinen,C. Pilate. Zopiclone, sleep and health-related quality of life. Human psychopharacology clinical &experimental.
82. Mignot E, Mayleben D, Fietze I, Leger D, Zammit G, Bassetti CLA, Pain S, Kinter DS, Roth T; investigators. Safety and efficacy of daridorexant in patients with insomnia disorder: results from two multicentre, randomised, double-blind, placebo-controlled, phase 3 trials. Lancet Neurol. 2022 Feb;21(2):125-139. doi: 10.1016/S1474-4422(21)00436-1. Erratum in: Lancet Neurol. 2022 Jan 20;: Erratum in: Lancet Neurol. 2022 Jun;21(6):e6. PMID: 35065036.
83. Monti JM, Attali P, Monti D, Zipfel A, de la Giclais B, Morselli PL. Zolpidem and rebound insomnia--a double-blind, controlled polysomnographic study in chronic insomniac patients. Pharmacopsychiatry. 1994 Jul;27(4):166-75. doi: 10.1055/s-2007-1014298. PMID: 7972349.
84. Richardson G, Wang-Weigand S. Effects of long-term exposure to ramelteon, a melatonin receptor agonist, on endocrine function in adults with chronic insomnia. Hum Psychopharmacol. 2009 Mar;24(2):103-11. doi: 10.1002/hup.993. PMID: 19090503.
85. Wang-Weigand S, Watissée M, Roth T. Use of a post-sleep questionnaire-interactive voice response system (PSQ-IVRS) to evaluate the subjective sleep effects of ramelteon in adults with chronic insomnia. Sleep Med. 2011 Oct;12(9):920-3. doi: 10.1016/j.sleep.2011.06.008. Epub 2011 Sep 16. PMID: 21925941.
86. Heidrich H, Ott H, Beach RC. Lormetazepam - a benzodiazepine derivative without hangover effect? A double-blind study with chronic insomniacs in a general practice setting. International Journal of Clinical Pharmacology, Therapy and Toxicology 1981;191:11-7.
87. Begg EJ, Robson RA, Frampton CM, Campbell JE. A comparison of efficacy and tolerance of the short acting sedatives midazolam and zopiclone. New Zealand Medical Journal 1992;105(944):428-9.
88. Moon CA, Ankier SI, Hayes G. Early morning insomnia and daytime anxiety—a multicentre general practice study comparing loprazolam and triazolam. British Journal of Clinical Practice 1985;399:352-8.
89. Roger M, Attali P, Coquelin JP. Multicenter, double-blind, controlled comparison of zolpidem and triazolam in elderly patients with insomnia. Clinical Therapeutics 1993;151:127-36
90. NCT00156533. Long Term Treatment With Zolpidem: Nightly and Intermittent Dosing. clinicaltrials.gov/ct2/show/NCT00156533
91. NCT00383643. Xyrem Sodium Oxybate and Ambien Zolpidem Tartrate in the Treatment of

Chronic Insomnia. clinicaltrials.gov/ct2/show/NCT00383643

92. NCT00755495. Safety and Efficacy of Ramelteon and Doxepin in Subjects With Chronic Insomnia. clinicaltrials.gov/ct2/show/NCT00755495
93. NCT00756002. Safety and Efficacy Study of Ramelteon in Subjects With Chronic Insomnia. clinicaltrials.gov/ct2/show/NCT00756002
94. NCT03375203. A Study to Evaluate the Efficacy, Safety, and Tolerability of JNJ-42847922 in Participants With Insomnia Disorder. https://clinicaltrials.gov/ct2/show/NCT03375203
95. NCT03545191. Study to Assess the Efficacy and Safety of ACT-541468 in Adult and Elderly Subjects With Insomnia Disorder. https://clinicaltrials.gov/ct2/show/NCT03545191
96. NCT03575104. Study to Assess the Efficacy and Safety of ACT-541468 in Adult and Elderly Subjects Suffering From Difficulties to Sleep. https://clinicaltrials.gov/ct2/show/NCT03575104
97. Hajak G, Rodenbeck A, Voderholzer U, Riemann D, Cohrs S, Hohagen F, Berger M, Rüther E. Doxepin in the treatment of primary insomnia: a placebo-controlled, double-blind, polysomnographic study. J Clin Psychiatry. 2001 Jun;62(6):453-63. doi: 10.4088/jcp.v62n0609. PMID: 11465523.
98. Elie R, Lavoie G, Bourgouin J, Le Morvan P. Zopiclone versus flurazepam in insomnia: prolonged administration and withdrawal. Int Clin Psychopharmacol. 1990 Oct;5(4):279-86. doi: 10.1097/00004850-199010000-00005. PMID: 2081899.
99. NCT00177216. Characteristics of Sleep Patterns in Young Adults With and Without Insomnia. https://clinicaltrials.gov/ct2/show/NCT00177216
100. Rosenberg R, Murphy P, Zammit G, Mayleben D, Kumar D, Dhadda S, Filippov G, LoPresti A, Moline M. Comparison of Lemborexant With Placebo and Zolpidem Tartrate Extended Release for the Treatment of Older Adults With Insomnia Disorder: A Phase 3 Randomized Clinical Trial. JAMA Netw Open. 2019 Dec 2;2(12):e1918254. doi: 10.1001/jamanetworkopen.2019.18254. Erratum in: JAMA Netw Open. 2020 Apr 1;3(4):e206497. Erratum in: JAMA Netw Open. 2021 Aug 2;4(8):e2127643. PMID: 31880796; PMCID: PMC6991236.

Appendix 16 The results of small-study effects bias

zolpidem vs placebo in somnolence

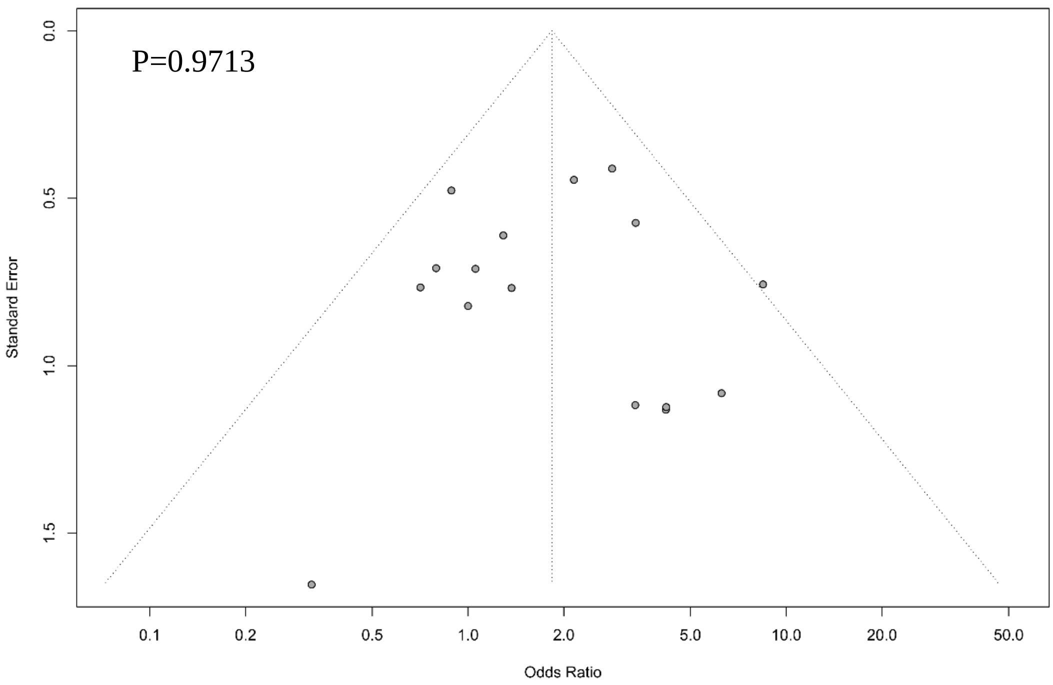

zolpidem vs placebo in headache

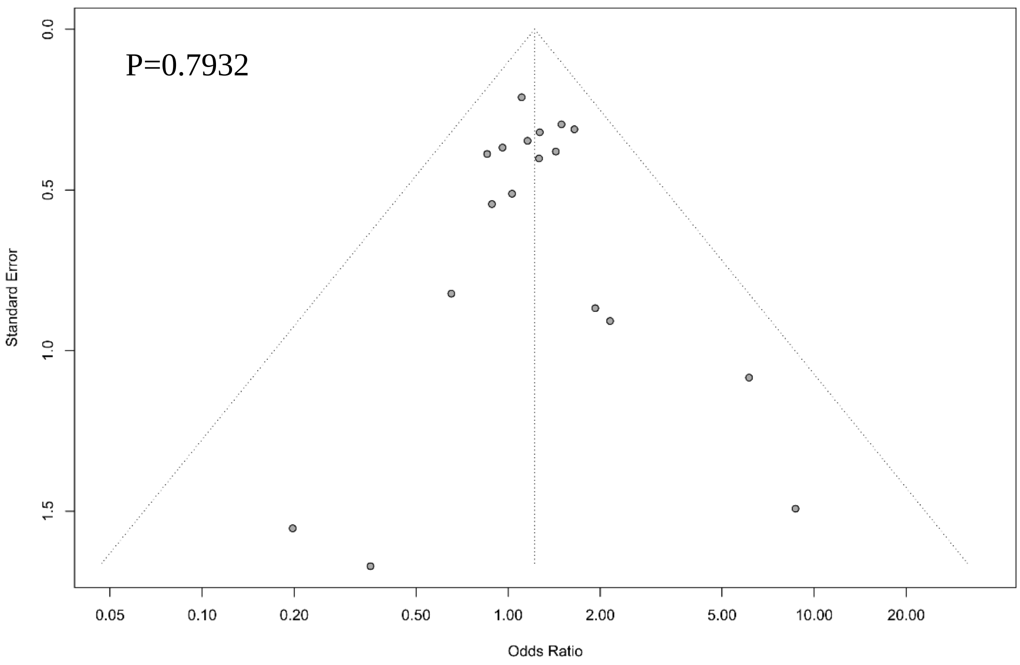

zolpidem vs placebo in dizziness

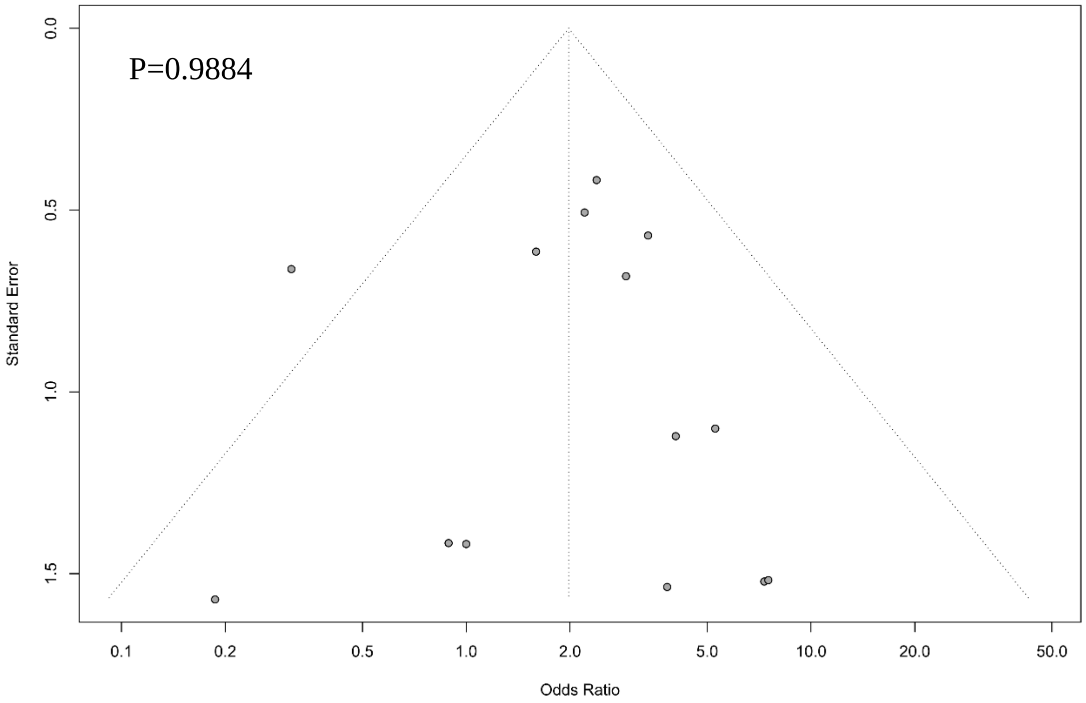

Supplement: Supplementary file 1 [file DataSheet1.pdf]
